# A ReaxFF Molecular Dynamics Study of Hydrogen Diffusion in Ruthenium–The Role of Grain Boundaries

**DOI:** 10.1021/acs.jpcc.1c08776

**Published:** 2022-03-23

**Authors:** Chidozie Onwudinanti, Mike Pols, Geert Brocks, Vianney Koelman, Adri C. T. van Duin, Thomas Morgan, Shuxia Tao

**Affiliations:** † Dutch Institute for Fundamental Energy Research, P. O. Box 6336, 5600 HH Eindhoven, The Netherlands; ^‡^Materials Simulation and Modelling, Department of Applied Physics and ^∥^Department of Applied Physics, Eindhoven University of Technology, 5600 MB Eindhoven, The Netherlands; ¶ Center for Computational Energy Research, P. O. Box 6336, 5600 HH Eindhoven, The Netherlands; § Computational Materials Science, Faculty of Science and Technology, MESA+ Institute for Nanotechnology, University of Twente, P. O. Box 217, 7500 AE Enschede, The Netherlands; ⊥ Department of Mechanical Engineering, The Pennsylvania State University, University Park, Pennsylvania 16802, United States

## Abstract

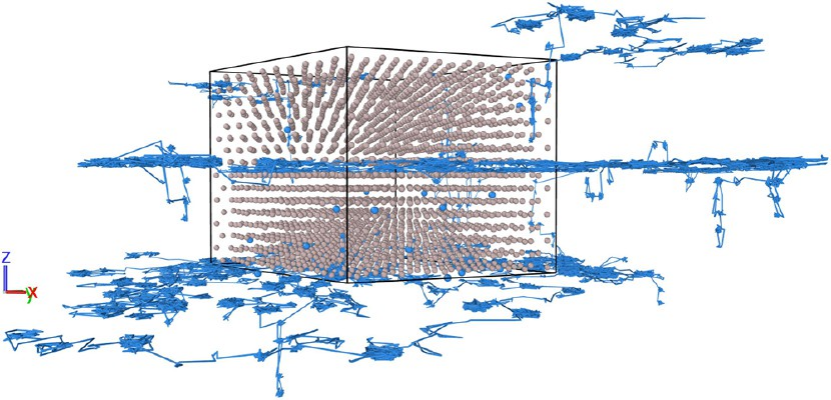

Ruthenium (Ru) thin films are used as protective caps for the multilayer mirrors in extreme ultraviolet lithography machines. When these mirrors are exposed to atomic hydrogen (H), it can permeate through Ru, leading to the formation of hydrogen-filled blisters on the mirrors. H has been shown to exhibit low solubility in bulk Ru, but the nature of H diffusion through Ru and its contribution to the mechanisms of blistering remain unknown. This work makes use of reactive molecular dynamics simulations to study the influence of imperfections in a Ru film on the behavior of H. For the Ru/H system, a ReaxFF force field which reproduces structures and energies obtained from quantum-mechanical calculations was parametrized. Molecular dynamics simulations have been performed with the newly developed force field to study the effect of tilt and twist grain boundaries on the overall diffusion behavior of H in Ru. Our simulations show that the tilt and twist grain boundaries provide energetically favorable sites for hydrogen atoms and act as sinks and highways for H. They therefore block H transport across their planes and favor diffusion along their planes. This results in the accumulation of hydrogen at the grain boundaries. The strong effect of the grain boundaries on hydrogen diffusion suggests tailoring the morphology of ruthenium thin films as a means to curb the rate of hydrogen permeation.

## Introduction

Hydrogen inclusion is often detrimental to the mechanical response and associated desirable properties of materials. Such hydrogen-induced damage^1^ poses problems in many fields, including hydrogen (H) transport and storage,^2,3^ nuclear fusion,^4^ and extreme ultraviolet (EUV) lithography.^5^ The latter application employs multilayer reflective optics which are susceptible to hydrogen-induced damage. Ruthenium (Ru) can serve as a capping layer in these mirror; therefore, the transport of H through the metal is an important factor in determining the operational lifetime of the optical elements.

Although the interaction of H with ruthenium surfaces is well-represented in the literature,^6−8^ research on H in the Ru bulk is sparse and limited to H solubility in Ru and the thermodynamics of hydride formation.^9,10^ Our previous study using Density Functional Theory (DFT) gave positive formation energies of +0.34/+0.85 eV for an H atom occupying one octahedral/tetrahedral site in Ru, indicating the low solubility of H in Ru.^11^ In contrast to numerous other metals,^12^ the diffusion of H in Ru has received little attention. This is in part due to the technical challenges in detecting the hydrogen in the Ru bulk because of its low concentration. One indirect measurement of the diffusion rate of H through Ru thin films has been reported,^13^ using optical changes in an yttrium hydride substrate.

H diffusion in the bulk of a metal typically occurs via hopping of H atoms through the interstitial sites in the lattice. However, in real samples, this is altered by the interaction of H with defects in the crystal lattice, such as vacancies, voids, phase boundaries, and grain boundaries (GBs).^12^ These defects provide microstructural trap sites for H—sites at which the energy of inclusion of the solute atom is significantly lower, and the residence time longer, than at the usual interstitial sites. The number and nature of these traps is therefore a determining factor in the overall diffusion of the hydrogen in the metal. For example, in nickel GBs are reported to retard diffusion.^14^ In aluminum, GBs have been shown to enhance or suppress H diffusion depending on the size of the grains^15^ and to block H diffusion across the boundary plane while enhancing diffusion along it.^16^ In hydrogen storage, the rate of hydrogenation of magnesium was shown to increase with grain size.^17^ Notably, the hydrogen diffusion model in Ru in ref (13) assumes GB transport to be dominant. An atomistic view of the role of GBs in H diffusion in Ru is, however, lacking.

Computer simulations, such as molecular dynamics (MD), are an efficient way to study H diffusion in diverse metals and are in particular suited to studying the effect of GBs. In nickel, certain grain boundaries have been shown to enhance diffusion along their plane and to hinder diffusion across it; others appear to have no significant effect on diffusivity.^18^ In α-iron, GBs have been shown to slow diffusion by trapping H atoms.^19^ These MD studies illustrate that the GB effects—enhancement or retardation of diffusion—depend strongly on the specific grain boundary in consideration; in tungsten, certain GBs provide deep traps which hold H atoms in place,^20^ while others provide paths of low resistance.^21^ These paths of low resistance allow for a more rapid transport of H, resulting in so-called short-circuit diffusion.

In an earlier work, we calculated the energy barrier to H jumps within the perfect Ru lattice using ab initio methods.^11^ However, the computational cost of dynamic simulations with quantum-mechanical methods is prohibitive. Moreover, grain boundaries disrupt the periodicity of a crystal lattice, so large unit cells are required to model them accurately. They also come in a large variety of possible configurations, and due to the complexity of the interfaces, the potential energy surface may be quite complicated. Therefore, a technique which balances accuracy with reasonable computational load is required for a simulation of these structures. With its dynamic bond breaking and bond formation, the bond-order-based ReaxFF method is well-suited to the study of such features as GBs and can sample the potential energy surface to an extent which is not accessible to ab initio methods.

Here we develop a ReaxFF force field for the Ru/H system which reproduces the energies and properties obtained with quantum-mechanical methods. The force field is used for molecular dynamics simulations of the diffusion of H through the intact Ru crystal lattice and through structures with different grain boundaries. We show the effect of these GBs on the rate and pattern of H transport through Ru and report diffusion coefficients for the simulated structures. We find that GBs have a profound effect on the H diffusion dynamics in Ru at all simulated temperatures. We discuss the implications of these findings for H transport through Ru bulk and thin films.

## Computational Methods

The critical component of a molecular dynamics simulation is the force field. This work employs ReaxFF,^22−24^ a bond-order-based force field method which allows the formation and breaking of bonds in a dynamic simulation. We have developed a set of force field parameters for the Ru/H system using a Monte Carlo global optimization algorithm,^25^ which minimizes an objective function of the form
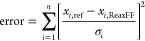
1where *x*_*i*,ref_ and *x*_*i*,ReaxFF_ are the reference value of the property and the value computed with ReaxFF, respectively, σ_*i*_ represents the weighting of the property, and the sum is over all the entries in the training set.

The force field parameters are optimized to match results obtained from first-principles calculations. The training set includes Ru equations of state for multiple crystal structures, surface formation energies, H adsorption energies on Ru surfaces, hydride formation energies, and bond length scans. The general ReaxFF parameters are as published by Kim et al.,^26^ and the parameters for H are taken from the set developed by Senftle et al.^27^ The Ru and Ru–H parameters were newly generated for this study.

The Vienna Ab Initio Simulation Package (VASP)^28−30^ is used for all periodic DFT calculations, which are performed with the generalized gradient approach as proposed by Perdew, Burke, and Ernzerhof (PBE).^31^ The convergence parameters are as follows: energy cutoff of 400 eV; residual force criterion of 1 × 10^–2^ eV/Å; energy convergence criterion of 1 × 10^–5^ eV. Slab calculations are performed with a (9 × 9 × 1) Γ-centered *k*-points grid, while bulk calculations are done with a (9 × 9 × 9) grid; all atoms are allowed to relax in the optimization process.

Charges from atom clusters and molecules are also included in the training set. For clusters and molecules, we have used the AMS2020 software package (version 2020.101) under license from SCM.^32^ The ADF molecular DFT in AMS2020 was run with the PBE exchange-correlation, the triple-ζ (TZ2P) basis set, and the zeroth order regular approximation (ZORA) relativistic scalar correction. Charges are extracted from the Mulliken population analysis. More information on the force field parameters and training set can be found in the Supporting Information.

The ReaxFF MD calculations are performed in AMS2020. All the MD simulations employ periodic boundary conditions in three directions and are carried out with a velocity Verlet integrator with a time step of 0.25 fs. The atom locations as a function of time are tracked at intervals of 250 fs, i.e., every 1000 time steps. Initial velocities of the atoms are set according to a Maxwell–Boltzmann distribution at the target temperature. The system is then brought to equilibrium in a preparatory simulation of at least 0.1 ns duration, in an NpT ensemble with a Berendsen barostat set to 1 atm and a Nosé–Hoover chain (NHC) thermostat with a chain length of 10 and a damping constant of 25 fs. The main diffusion simulation is performed in an NVT ensemble with the NHC thermostat. We extract from the NVT simulation trajectory the mean-squared displacement (MSD) of the H atoms, using the MDAnalysis package.^33−37^ Diffusion coefficients are calculated from the slope of the MSDs according to the Einstein–Smoluchowski relation:
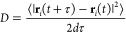
2where **r** is the position of the H atom, τ is the elapsed time, and *d* is the dimensionality of the system. The average is taken over time steps and all H atoms. The temperature-dependent diffusion coefficients are fitted to the Arrhenius expression *D* = *D*_0_ exp(−*E*_a_/*k*_B_*T*), which yields a pre-exponential factor *D*_0_ and activation energy *E*_a_, where *k*_B_ is the Boltzmann constant and *T* is the temperature. Hydrogen diffusion data are generated from MD simulations and analyzed for three representative structures: the pristine Ru crystal and the two different types of GBs described below. The simulated structures are summarized in Table 1.

**Table 1:** Simulated Ru Structures

structure	box dimensions (Å)	no. of Ru atoms	no. of H atoms
pristine	40.6 × 37.5 × 34.3	3840	40
Σ7 tilt GB (tilt)	27.0 × 46.8 × 42.8	3690	40
Σ7 twist GB (twist)	36.1 × 36.1 × 34.8	2800	28

**Figure 1:**
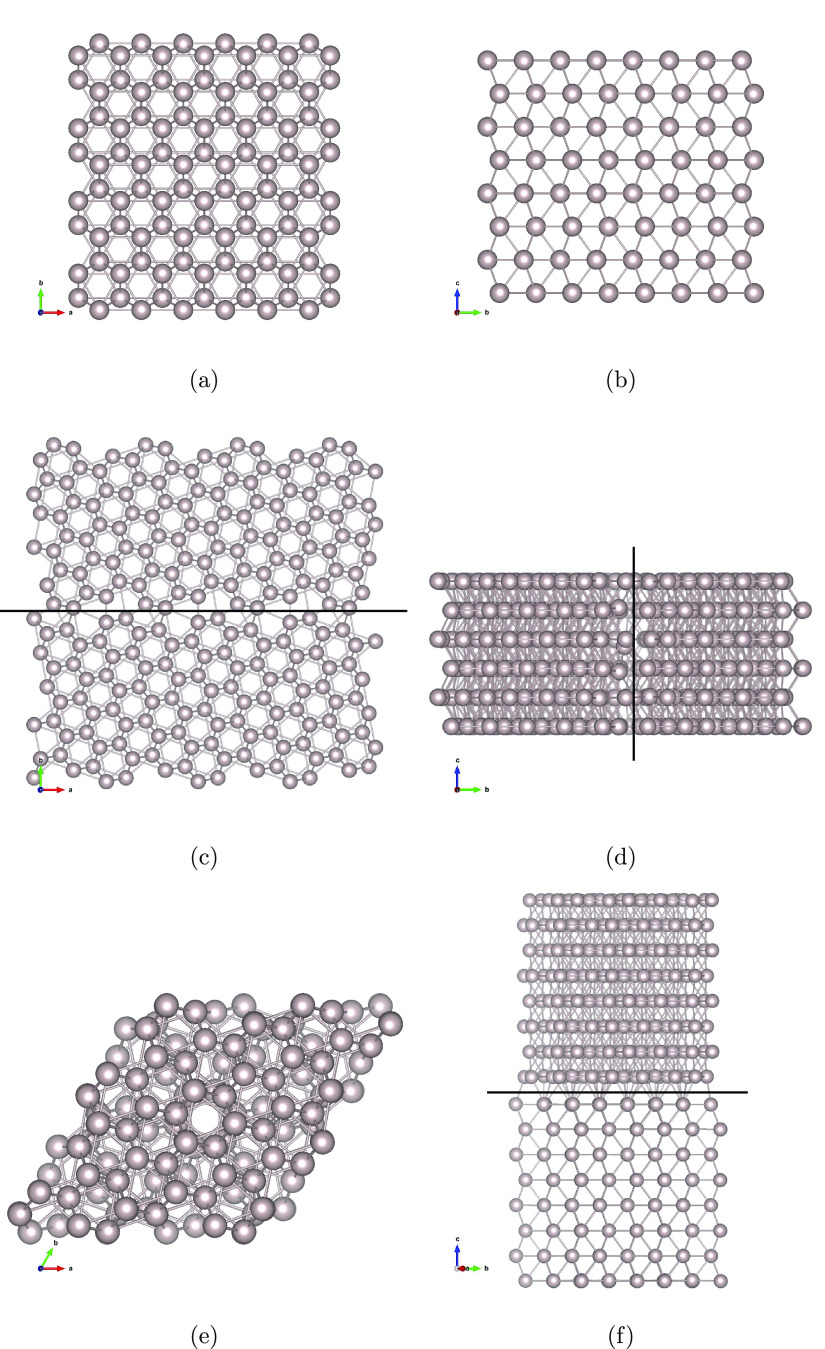
(a) Top view and (b) side view of pristine hcp Ru; (c) top view and (d) side view of the Σ7 symmetric tilt GB; (e) top view and (f) side view of the Σ7 twist GB. The black line marks the GB plane.

The crystallography of a GB can be described completely in terms of five parameters: three to describe the misorientation of the two grains and two to describe the inclination of the boundary relative to the axes of either of the crystals. The misorientation is determined by the rotation axis, e.g., [0001] and 38.21° for the tilt GB and [0001] and 21.79° for the twist GB. The inclination of the boundary is defined by the GB plane, (011̅0) for the tilt GB and (0001) for the twist GB. The coincidence site lattice (CSL) concept is a convenient way to denote special misorientations, rotation angles at which the superposition of two crystals results in a number of lattice points coinciding and forming a sublattice of the two crystal lattices. The CSL is characterized by its Σ value, the ratio of the CSL’s unit cell volume to the volume of the generating bulk lattice cell. Because the number of feasible GBs is very large, a thorough exploration of all types is impractical. Therefore, a selection of representative structures is necessary. The first requirement is low formation energy, which implies a high likelihood of occurrence. Another factor is the difference between the selected structures; the more dissimilar the systems simulated, the more information can be extracted. We have selected the Σ7 symmetric tilt GB and the Σ7 twist GB, with rotation about [0001]; they are generated with the free and open-source Atomsk software^38^ and illustrated in Figure 1. For a more thorough discussion of the chosen GBs and their properties, see Bruggeman et al.^39^ and Zheng et al.^40^

## Results and Discussion

### Force Field Validation

We have performed a series of calculations to assess the accuracy of the Ru/H force field. The test cases include evaluation of the lattice parameters for the hexagonal close-packed (hcp) Ru crystal and mechanical properties. A comparison of the Ru bulk properties from ReaxFF and DFT is shown in Table 2.

**Table 2:** HCP Ru Properties from ReaxFF and DFT

method	*a* (Å)	*c*/*a*	*V*_0_ (Å)^3^	*B* (GPa)
ReaxFF	2.73	1.60	14.2	332
DFT	2.72	1.58	13.7	312

**Figure 2:**
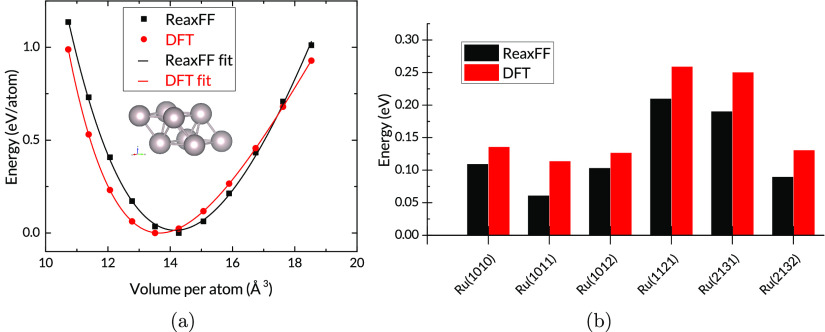
(a) Equations of state for hcp Ru from the ReaxFF force field and DFT and (b) energy per atom of various Ru surfaces relative to Ru(0001).

The ReaxFF-computed lattice parameters *a* (2.73 Å) and *c*/*a* (1.60) for hexagonal close-packed (hcp) ruthenium are in good agreement with the DFT-computed values and with the experimental data, 2.71 Å and 1.58, respectively.^41^ The equilibrium volume per atom *V*_0_ is overestimated slightly, by 4%. Mechanical properties from the reference DFT calculations are also reproduced to a good standard as shown in Figure 2a. Bulk moduli calculated from Birch–Murnaghan equations of state are 313 and 333 GPa for ReaxFF and DFT, respectively.

The force field also shows a good qualitative reproduction of relative energies for a number of Ru slabs with different exposed facets. As shown in Figure 2b, although the absolute values are almost uniformly underestimated, the trend is quite well-matched. The largest mismatch does not exceed 0.06 eV. It should be noted that these slabs were not included in the training set for the parametrization, so they serve as a validation of the force field’s performance outside the training space.

**Figure 3:**
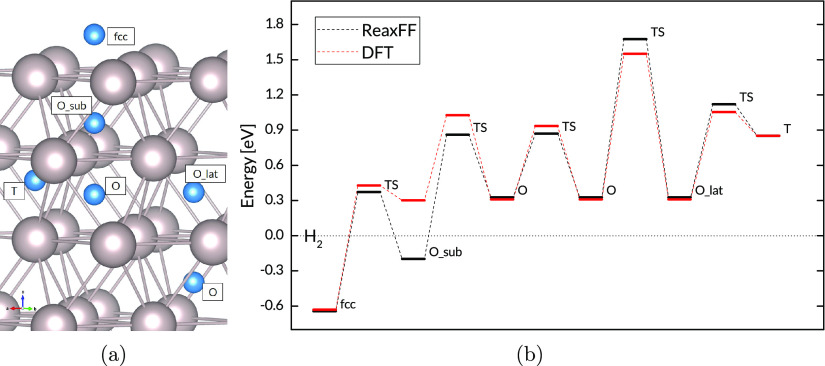
A comparison of adsorption energies and hydride formation energies (hydrogen at interstitial sites) on and in Ru and energies of the transition states along the diffusion paths, obtained with the ReaxFF force field and the DFT reference.^11^

For the diffusion simulations, it is especially important that the force field reproduces the energies of H in the interstitial states within the Ru bulk. Figure 3 shows a comparison of ReaxFF and DFT energies for key sites of H in Ru. The interstitial hydride formation energies are calculated as
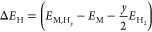
3where *y* is the number of H atoms, while , *E*_M_, and  stand respectively for the total energy of the metal hydride, the energy of the host metal structure, and the energy of a H_2_ molecule.

**Figure 4:**
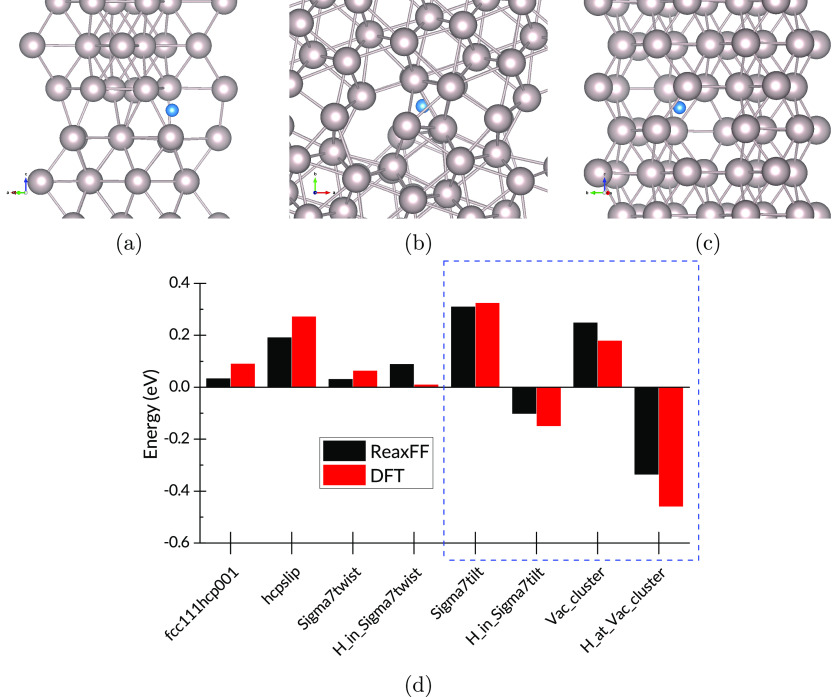
(a) H in a twist GB, (b) H in a tilt GB, (c) H at a vacancy cluster, and (d) energy per atom for Ru stacking faults, GBs, vacancy clusters, and H sites at defects.

**Figure 5:**
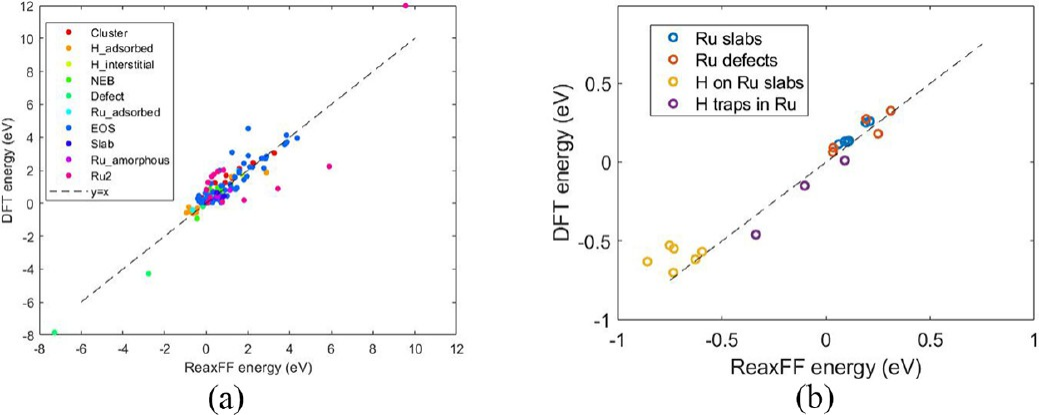
(a) Training set fit and (b) validation set for ReaxFF parameter fitting.

The agreement is good, within 0.15 eV for all but the octahedral site in the near-surface region, which is lower in energy than the H_2_ reference. The main deviation is found near the Ru(0001) surface. However, the discrepancy is unlikely to have a large impact on the simulated bulk diffusion. This conclusion is further strengthened by the force field’s reproduction of GBs and defect formation energies. Figure 4d shows the energies of GBs and stacking faults, as well as the energies of H sites at the two GBs and at a vacancy cluster. The structures and energies within the blue rectangle are outside the training set. Figure 5 shows parity plots for the training set and the validation set, which demonstrate the successful reproduction of DFT energies by the ReaxFF force field.

### H Diffusion in Pristine Ru

**Figure 6:**
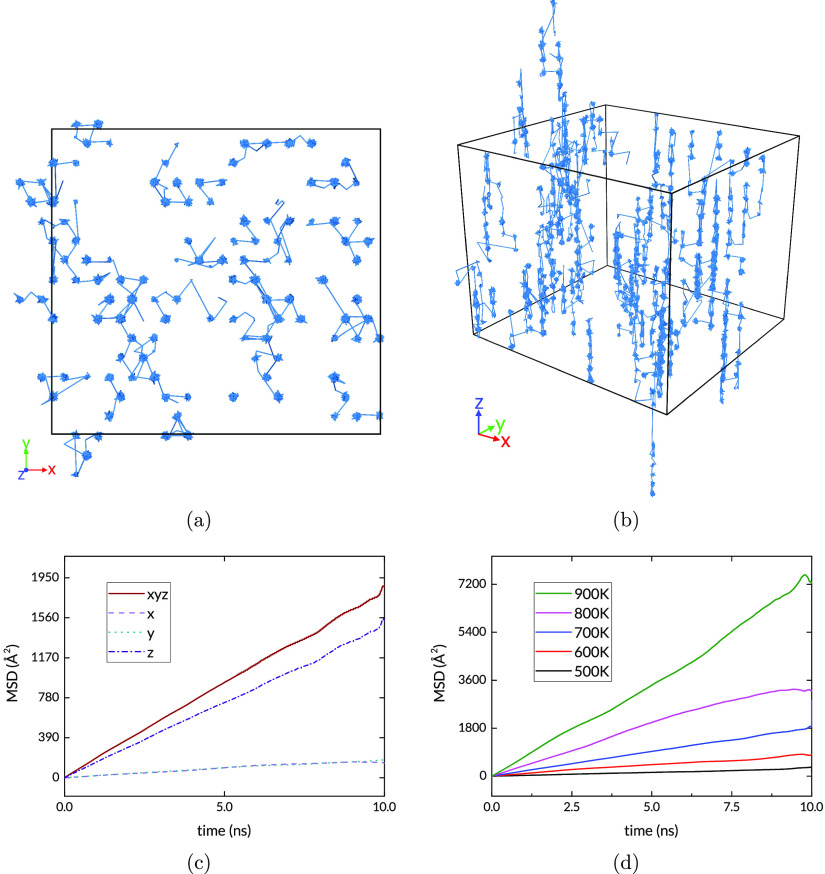
(a) and (b) H trajectories in pristine hcp Ru simulation at 700 K. (c) MSD at 700 K; (d) MSD for all simulated temperatures.

H transport in Ru proceeds via a series of jumps between interstitial sites, over the energy barriers shown in Figure 3. The barriers indicate a preference for octahedral-to-octahedral jumps in the *c* direction of the hcp lattice, which is always the *Z* direction in our coordinate system. This can be seen from the trajectories and the MSD plot in Figure 6. First we observe that the H paths include nodes situated at the octahedral sites, confirming that the solute atoms indeed spend many time steps at these sites between successful hops. It is also apparent that more successful jumps occur in the *Z* direction. The trajectories are unwrapped from the periodic translation into the simulation box, so the extent of the map in Figure 6b shows the disparity in vertical and horizontal displacement. Figure 6c shows the MSD for the 700 K NVT simulation. In the diffusive regime, a three-dimensional random walk through the interstitial sites, the MSD has a linear dependence on the elapsed time. The diffusive regime is reached quickly, as the H atoms quickly reach an equilibrium distribution in the intact lattice. The fluctuation at the long time scales is due to the progressively smaller number of data points with the large time lags. The MSD contribution of each of the spatial dimensions is also plotted; the full MSD is the sum of the MSDs in each spatial dimension. In keeping with the observed difference in trajectories, the MSD contribution of the *X* and *Y* directions is much smaller than that of the *Z* component. As expected, there is a monotonic increase in MSD as the temperature is increased, as shown in Figure 6d.

### H Diffusion in Ru with a Tilt GB

**Figure 7:**
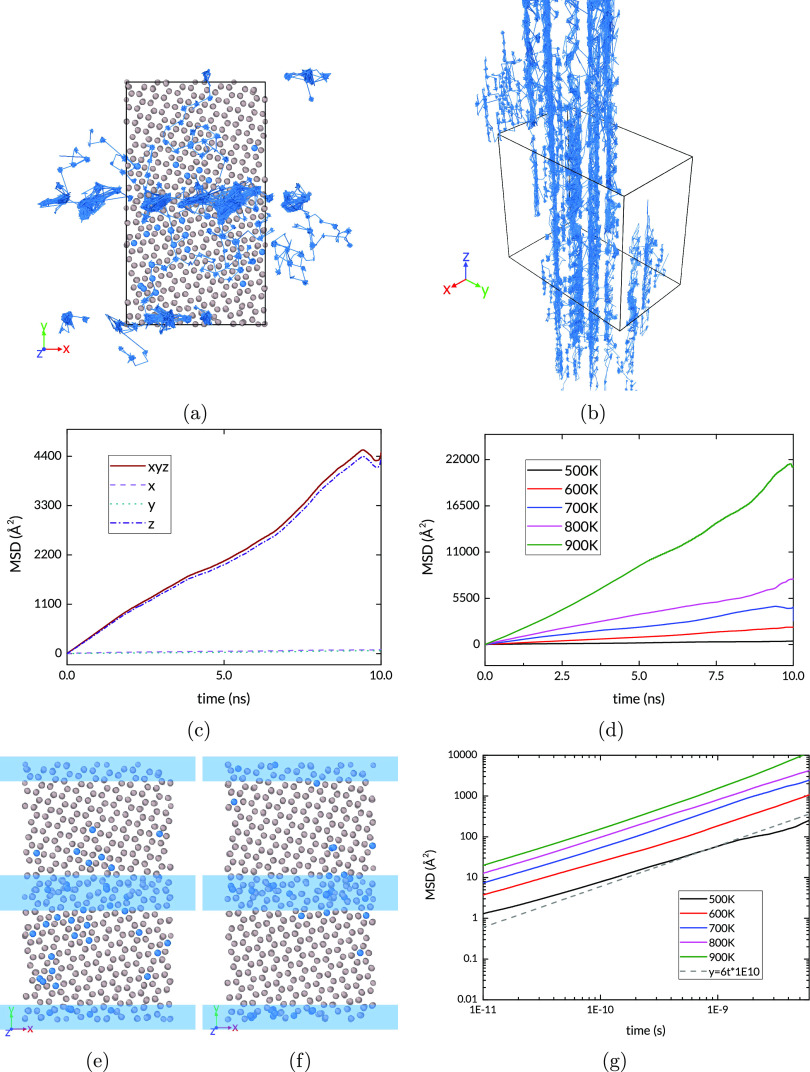
(a) and (b) H trajectories in the tilt GB simulation at 700 K; (c) MSD at 700 K; (d) MSD for all simulated temperatures. Snapshots of the (e) start and (f) end of the tilt GB NVT simulation at 700 K; blue bands show GBs. (g) Log–log MSD plot for all simulated temperatures of the tilt GB. The dashed line has a slope of 1, which indicates a diffusive regime.

The introduction of a tilt grain boundary has a marked effect on the rate and direction of H transport in Ru. As the trajectories in Figure 7 show, here too the extent of the unwrapped trajectories in the *XY* plane is much smaller than in the *Z* direction. In each of the grains, the predominance of jumps along the *Z* direction remains and is overall enhanced within the GB. Here the atoms end up in channels, within which they remain, traveling mainly along the *Z* direction. The MSD over the same duration as the pristine structure is doubled. Furthermore, it can be seen that there is a much smaller, essentially negligible contribution from diffusion in the *XY* plane.

**Figure 8:**
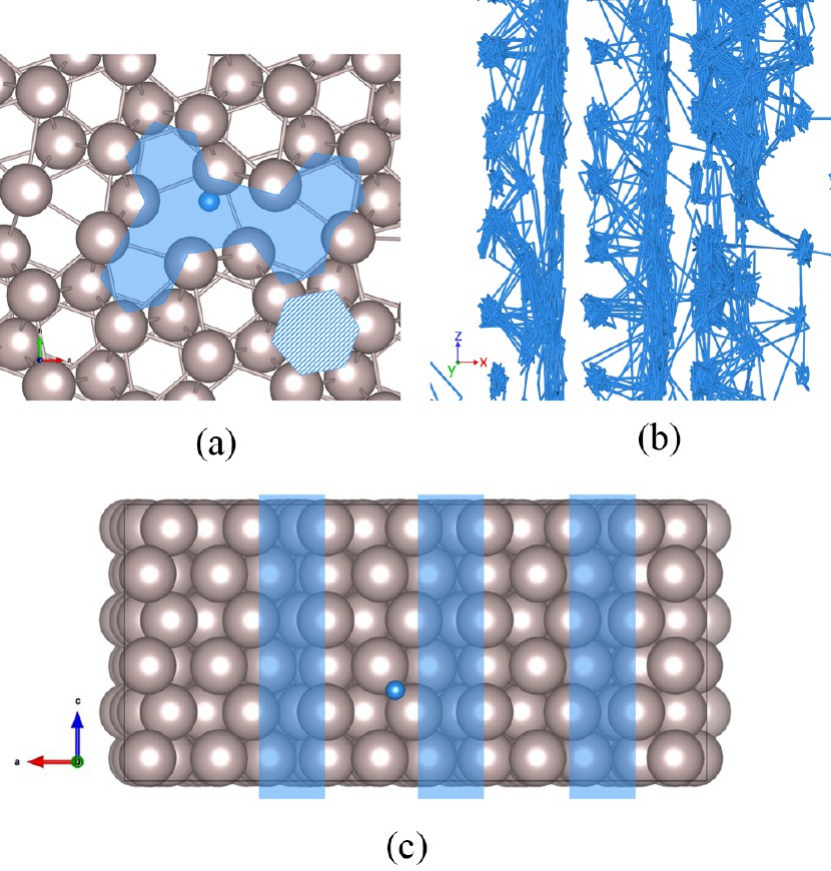
(a) Top view of the Ru-depleted zone at the tilt GB plane: the shaded hexagon shows the span of the octahedral site, while the semitransparent irregular polygon shows the Ru-depleted GB channel. (b) Enlarged side view of the tilt GB trajectory and (c) a section through the tilt GB plane, with channels highlighted.

The low energy of the GB sites (Figure 4) suggests that the H atoms will tend to be trapped at these sites, and this is reflected in the trajectory maps of Figure 7. There is little transport across the plane of the grain boundary from one grain to the other. It follows that the equilibrium diffusive regime in this structure is reached only when the population of these GB sites stabilizes. Figures 7e and 7f show the distribution of H atoms in the structure at the beginning and end of the NVT simulation. It can be seen that the interstitial sites in the grains are depleted, with the H population at the boundary rising accordingly. Figure 7g is a log–log plot of the MSD for all the simulated temperatures. It shows that the diffusive regime is reached quickly at the higher temperatures, with all the plots reaching a slope of unity by 500 ps; the exception is the 500 K simulation which shows a rough match. This is likely because at such relatively low temperature (a) the H distribution is still out of equilibrium, and/or (b) the number of diffusion events being averaged is small. A closer view is shown in Figure 8 in which the region with the greatest density of trajectory lines can be seen. The tilt GB has channels with larger volume than the native hcp lattice allows, which explains their accommodation of the solute atoms. The image suggests a significant difference in the energy barriers for hops between sites within the channels and the barrier to exit, with the latter being higher. Figure 8b shows a close-up of the side view of the tilt GB, in which three separate channels can be demarcated. The section through the GB plane shown in Figure 8c highlights the short-circuit paths formed at the GB which dominate the diffusion through this structure.

### H Diffusion in Ru with a Twist GB

**Figure 9:**
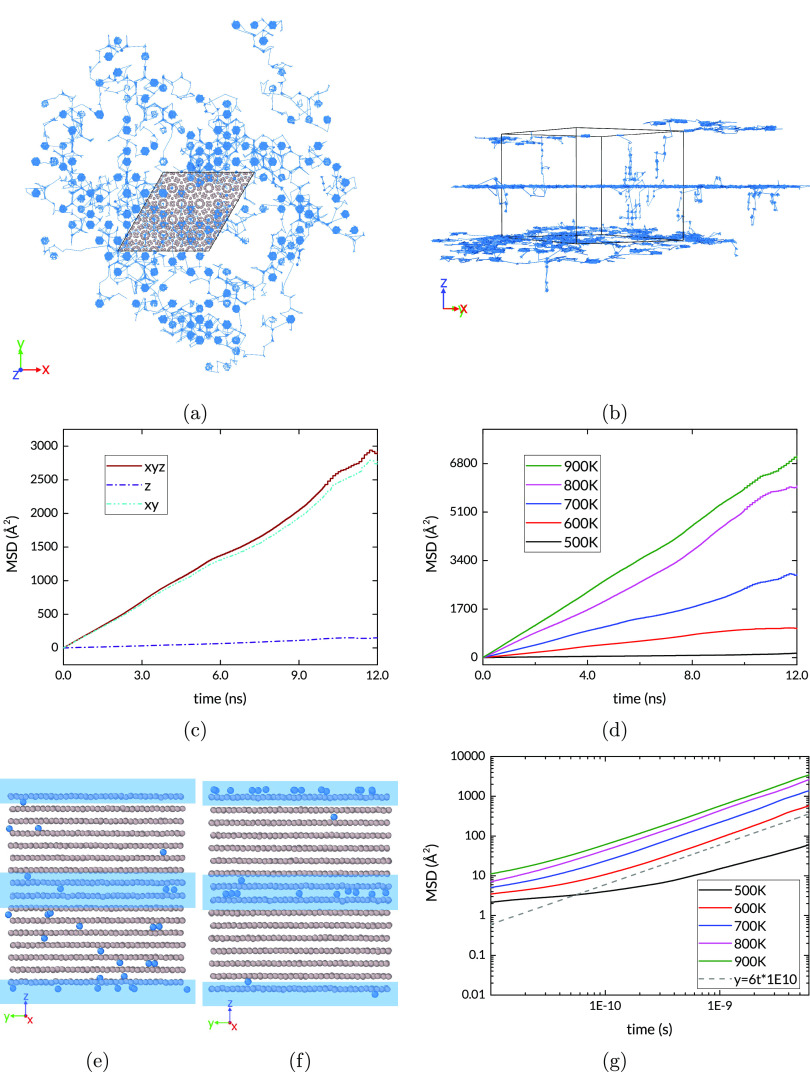
(a) and (b) H trajectories in the twist GB simulation at 700 K; (c) MSD at 700 K; (d) MSD for all simulated temperatures. Snapshots of the (e) start and (f) end of the tilt GB NVT simulation at 700 K; blue bands show GBs. (g) Log–log MSD plot for all simulated temperatures of the twist GB. The dashed line has a slope of 1, which indicates a diffusive regime.

The second grain boundary structure also influences the diffusion process significantly. The plane of the boundary is horizontal, and the sites in the boundary put H atoms lower in energy than the octahedral site. Figure 9 shows the H trajectories, which lie predominantly in the plane of the boundary. The movement of H atoms at the twist GB sites contrasts strongly with the tightly bound vibrations at the octahedral site; the nodes of the latter are quite small compared to the broadly smeared loci at the former. Most importantly for the overall transport, this structure obstructs the otherwise dominant diffusion along the *Z* direction, with the horizontal reach of the trajectory now greater. The MSD plot reflects this, with the *Z* contribution practically zero, while the overall MSD magnitude is halfway between the values for the pristine structure and the tilt GB. The diffusion in the grains ultimately leads the solute atoms to accumulate at the boundary (Figure 9f). This proceeds at a temperature-dependent rate. The NVT simulation at 500 K does not reach the slope of a random-walk diffusive regime within the 12 ns duration of the run; all the higher-temperature simulations reach this by the 1 ns mark (Figure 9g).

**Figure 10:**
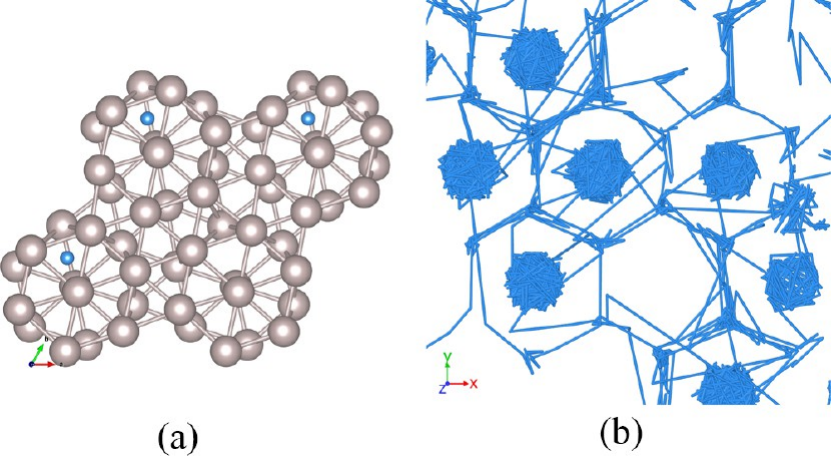
(a) H sites at the twist GB plane: the wheels are formed by atoms from the interfacing grains; (b) enlarged top view of the twist GB trajectory.

Figure 10 offers a closer look at the behavior of H in the twist GB plane during the MD simulation. The trajectory lines show that H atoms have a strong affinity for the sites in the GB plane. The twist GB has formed hexagonal “wheels” between the top and bottom grains, within which the H atom moves, jumping intermittently to an identical neighboring region. These traps show up in the trajectory lines of Figure 9a as the large nodes. The hexagonal symmetry of the interfacing grains is reflected in the paths around and between these traps.

### Diffusion Coefficients

**Figure 11:**
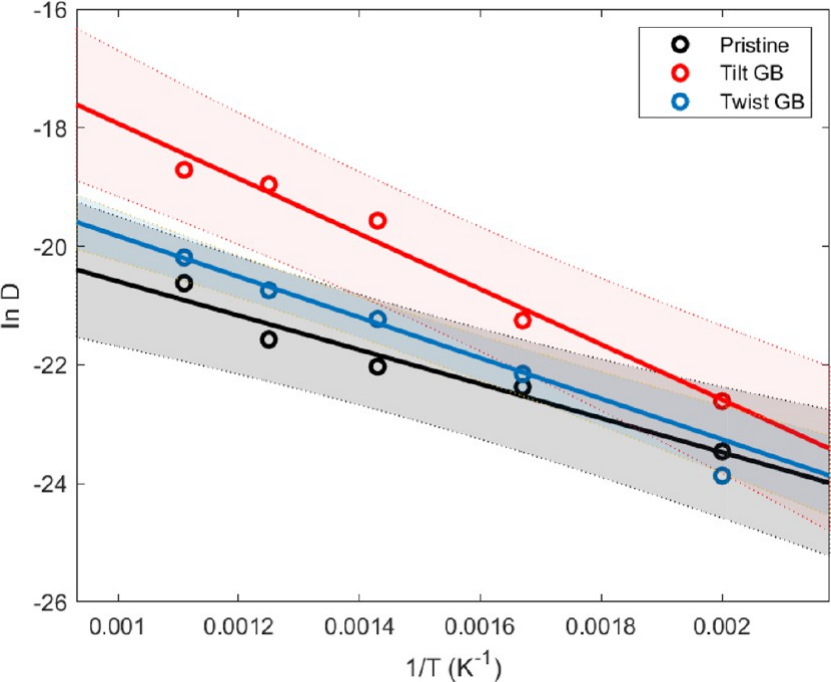
Diffusion coefficients for all simulated structures and temperatures, with confidence bands for the fit to the Arrhenius expression; the twist GB point at 500 K (0.002 K^–1^) is an outlier and excluded from the fit, as it does not represent a diffusive regime.

The MSD for each of the diffusion simulations yields the diffusion coefficient at each temperature, according to eq 2. H diffusion in the intact Ru lattice is three-dimensional. However, in both GBs, the diffusion is mostly within the GB, such that the random-walk dimensionality is reduced. The *z* direction accounts for most of the displacement occurring in the tilt GB, making it 1D diffusion; the twist GB has H trajectories across the GB plane, i.e., 2D diffusion. Figure 6d shows a linear dependence of MSD on time for the entire duration of the simulation of the pristine Ru. However, the inhomogeneous GB structures do not reach a diffusive regime as rapidly. They also exhibit greater variation in the slope. Therefore, the MSD slope is taken only after 40% of the simulation time has elapsed, and the slope of the MSD has matched that of a random walk. Furthermore, to account for the noise, the diffusion coefficient is averaged from 10 overlapping intervals between the 40% mark and the end of the simulation. The diffusion coefficients are plotted in Figure 11. We fit the temperature-dependent diffusion coefficients to the Arrhenius expression. As shown in Figure 9g, the 500 K twist GB simulation does not reach the diffusive regime; the diffusion coefficient is therefore not included in the fit. We obtain a pre-exponential factor *D*_0_ and an activation energy *E*_a_ for temperature-dependent H diffusion in each of the simulated structures. These are, in m^2^ s^–1^ and eV, respectively: 2.0 × 10^–8^ and 0.25 for the pristine hcp Ru; 1.73 × 10^–6^ and 0.40 for the tilt GB structure; and 7.6 × 10^–8^ and 0.29 for the twist GB structure.

## Conclusion

We have developed a ReaxFF force field for the Ru/H system which reproduces DFT energies with high accuracy. We have applied the force field to the study of H diffusion in Ru, a topic previously underrepresented in the literature. We performed simulations of H diffusion though a perfect Ru crystal and through tilt and twist GBs, which have yielded diffusion coefficients for H in the hcp Ru crystal and in GBs. While they do not cover all possible GBs and defects which can influence H transport through polycrystalline Ru, the diffusion coefficients and the trajectory maps indicate that the character of H diffusion in Ru depends largely on the number and nature of GBs present.

Both the static calculations and the dynamic simulations show the presence of energetically favorable sites for H atoms in the boundary region. Also important is the fact that diffusion across the GBs is inhibited. These findings are similar to the results obtained for H in Al GBs.^16^ However, Pedersen and Jónsson^16^ observed diffusion through H hopping from the GB site out into the grain, whereas we find that the main trajectories lie within the GB. The diffusion coefficients we have extracted imply that at 300 K, the diffusion rates in the tilt and twist GBs are slightly lower than that of the perfect crystal. We can surmise that in moving through polycrystalline Ru, H atoms will hop between interstitial sites until they reach a GB, within which their residence time exceeds that of the interstitial sites. If the sites at the GB are occupied, an arriving H atom is repelled, as we observed minimal transport across GBs. For thin films, transport through the Ru will depend greatly on the morphology of the film. Since the Ru capping layers are mostly of (0001) orientation,^42^ the accumulation of H and preferential transport along the plane of the tilt GB suggest that this type of GB will dominate H diffusion through the films, enabling short-circuit diffusion through the film.

These results point to film morphology control as an important tool in preventing the permeation of hydrogen into multilayer mirrors. The results also give insight into the trapping and diffusion of H and other impurities in metal grain boundaries. The developed force field can be applied to the study of other phenomena, including surface interactions, while the results of the diffusion study will be of interest both for research and for technological applications.

## Supporting Information Available

ReaxFF training set (PDF)
ReaxFF training set geometries (PDF)
Training set description; force field parameters; mean-squared displacement plots of molecular dynamics simulations; images of hydrogen trajectories in the tilt grain boundary (PDF)
