# Supplementary material for: A ReaxFF Molecular Dynamics Study of Hydrogen Diffusion in Ruthenium–The Role of Grain Boundaries
Source: J Phys Chem C Nanomater Interfaces. 2022 Mar 23;126(13):5950–9. doi: 10.1021/acs.jpcc.1c08776 (PMC8996245; doi:10.1021/acs.jpcc.1c08776)
Supplement: Supplementary file 1 — jp1c08776_si_001.pdf [file jp1c08776_si_001.pdf]

```
CHARGE
RuH4_length-1 0.1 1 0.9719390081781158
RuH4_length-1 0.1 2 -0.235324780705597
RuH4_length-1 0.1 3 -0.266720769955017
RuH4_length-1 0.1 4 -0.234570544863259
RuH4_length-1 0.1 5 -0.2353229126542452
Ru2H6 0.1 1 0.397
Ru2H6 0.1 2 0.397
Ru2H6 0.1 3 -0.132
Ru2H6 0.1 4 -0.132
Ru2H6 0.1 5 -0.132
Ru2H6 0.1 6 -0.132
Ru2H6 0.1 7 -0.132
Ru2H6 0.1 8 -0.132
Ru2H3 0.1 1 0.461
Ru2H3 0.1 2 0.002
Ru2H3 0.1 3 -0.173
Ru2H3 0.1 4 -0.173
Ru2H3 0.1 5 -0.117
ENDCHARGE
ENERGY
# 1.0 + RuH4_length-1/1 - RuH4_length-2/1 0.02213473757910613
# 1.0 + RuH4_length-3/1 - RuH4_length-2/1 7.223719911892302
# 1.0 + RuH4_length-4/1 - RuH4_length-2/1 17.37569440615634
# 1.0 + RuH4_length-5/1 - RuH4_length-2/1 28.23103918898292
# 1.0 + RuH4_length-6/1 - RuH4_length-2/1 38.68349407667594
# 1.0 + RuH4_length-7/1 - RuH4_length-2/1 48.22325201192551
# 1.0 + RuH4_length-8/1 - RuH4_length-2/1 56.67128482581084
# 1.0 + RuH4_length-9/1 - RuH4_length-2/1 63.98287532611067
# 1.0 + RuH4_length-10/1 - RuH4_length-2/1 70.24579242036296
 1.0 + RuH2_angle-1/1 - RuH2_angle-6/1 3.736854993975479
 1.0 + RuH2_angle-2/1 - RuH2_angle-6/1 2.387170586876507
 1.0 + RuH2_angle-3/1 - RuH2_angle-6/1 1.337408565042445
 1.0 + RuH2_angle-4/1 - RuH2_angle-6/1 0.5772059904278281
 1.0 + RuH2_angle-5/1 - RuH2_angle-6/1 0.1136046417558479
 1.0 + RuH2_angle-7/1 - RuH2_angle-6/1 0.281531515314867
 1.0 + RuH2_angle-8/1 - RuH2_angle-6/1 1.000830902222158
 1.0 + RuH2_angle-9/1 - RuH2_angle-6/1 2.213204331843717
 1.0 + RuH2_angle-10/1 - RuH2_angle-6/1 3.994070839752721
 1.0 + RuH_length-1/1 - RuH_length-3/1 8.108852804413829
 1.0 + RuH_length-2/1 - RuH_length-3/1 1.477764576266893
 1.0 + RuH_length-4/1 - RuH_length-3/1 1.83294136897821
 1.0 + RuH_length-5/1 - RuH_length-3/1 5.729795303241538
 1.0 + RuH_length-6/1 - RuH_length-3/1 10.84804954847658
 1.0 + RuH_length-7/1 - RuH_length-3/1 16.62096187682322
 1.0 + RuH_length-8/1 - RuH_length-3/1 22.66639344730559
 1.0 + RuH_length-9/1 - RuH_length-3/1 28.73391709621108
 1.0 + RuH_length-10/1 - RuH_length-3/1 34.65886603037643
 1.0 + RuH2_angle-1/1 - RuH2_angle-6/1 3.817629456919503
 1.0 + RuH2_angle-2/1 - RuH2_angle-6/1 2.464460898399252
 1.0 + RuH2_angle-3/1 - RuH2_angle-6/1 1.383930246472127
 1.0 + RuH2_angle-4/1 - RuH2_angle-6/1 0.5949888412675222
 1.0 + RuH2_angle-5/1 - RuH2_angle-6/1 0.1213738790923478
 1.0 + RuH2_angle-7/1 - RuH2_angle-6/1 0.2699999374791499
 1.0 + RuH2_angle-8/1 - RuH2_angle-6/1 0.9835677925028026
 1.0 + RuH2_angle-9/1 - RuH2_angle-6/1 2.198885347260324
 1.0 + RuH2_angle-10/1 - RuH2_angle-6/1 3.980050855828154
 1.0 + RuH4_length-2/1 - RuH4_length-1/1 0.2471180967279425
```

```
 1.0 + RuH4_length-3/1 - RuH4_length-1/1 7.560696761920075
 1.0 + RuH4_length-4/1 - RuH4_length-1/1 17.7562463964683
 1.0 + RuH4_length-5/1 - RuH4_length-1/1 28.66019701472828
 1.0 + RuH4_length-6/1 - RuH4_length-1/1 39.16920760789474
 1.0 + RuH4_length-7/1 - RuH4_length-1/1 48.77693345661104
 1.0 + RuH4_length-8/1 - RuH4_length-1/1 57.27545311546375
 1.0 + RuH4_length-9/1 - RuH4_length-1/1 64.65004163474234
 1.0 + RuH4_length-10/1 - RuH4_length-1/1 70.95859558476671
 0.01 + Ruads1Hfcc/1 - trainingRuslab0001/1 - H2mol/2 -14.65116279
 0.01 + Ruads1Hhcp/1 - Ruads1Hfcc/1 1.7771704651167965
 0.1 + Ruads1Htop/1 - Ruads1Hfcc/1 10.630305813952873
 0.1 + Ruadsfccscand1/1 - Ruadsfccscand3/1 37.70131953488453
 0.1 + Ruadsfccscand2/1 - Ruadsfccscand3/1 13.054361162790883
 0.1 + Ruadsfccscand4/1 - Ruadsfccscand3/1 17.67512837209324
 0.1 + Ruadsfccscand5/1 - Ruadsfccscand3/1 44.10221674418608
 0.1 + Ruadshcpscand1/1 - Ruadshcpscand3/1 38.233154883721
 0.1 + Ruadshcpscand2/1 - Ruadshcpscand3/1 12.822658139533814
 0.1 + Ruadshcpscand4/1 - Ruadshcpscand3/1 17.15568604651162
 0.1 + Ruadshcpscand5/1 - Ruadshcpscand3/1 42.94676953488488
 0.01 + RuHbulk1Hbulk/1 - RuHbulk0Hbulk/1 - H2mol/2 7.261851512
 0.1 + RuHbulk2Hbulk/1 - RuHbulk0Hbulk/1 - H2mol/1 17.91448
 0.1 + RuHbulk3Hbulk/1 - RuHbulk0Hbulk/1 - H2mol/2 - H2mol/1 27.04361919
 0.1 + RuHbulk4Hbulk/1 - RuHbulk0Hbulk/1 - H2mol/1 - H2mol/1 39.17865674
 0.01 + tRuHbulkocta/1 - RuHbulk0Hbulk/1 - H2mol/2 7.2093023
 0.01 + tRuHbulktetra/1 - tRuHbulkocta/1 12.58919581
 0.1 + NEBocta2octa01/1 - NEBocta2octa00/1 7.256248837208659
 0.1 + NEBocta2octa02/1 - NEBocta2octa00/1 15.57243697674403
 0.1 + NEBocta2octa03/1 - NEBocta2octa00/1 7.151629999999841
 0.1 + NEBocta2octa04/1 - NEBocta2octa00/1 0.012434651162493537
 0.1 + NEBocta2octaPREbegin/1 - NEBocta2octa00/1 0.0
 0.1 + NEBocta2octaPREend/1 - NEBocta2octa00/1 0.012434651162493537
 0.1 + NEBfcc2octa01/1 - NEBfcc2octa00/1 3.6904681395344596
 0.1 + NEBfcc2octa02/1 - NEBfcc2octa00/1 16.68267348837162
 0.1 + NEBfcc2octa03/1 - NEBfcc2octa00/1 23.124015581394815
 0.1 + NEBfcc2octa04/1 - NEBfcc2octa00/1 21.535884883720428
 0.1 + NEBfcc2octa05/1 - NEBfcc2octa00/1 20.13027581395363
 0.1 + NEBfcc2octa06/1 - NEBfcc2octa00/1 21.696268604650868
# 0.01 + NEBfcc2octaPREbegin/1 - NEBfcc2octa00/1 0.0006555813952218159
# 0.01 + NEBfcc2octaPREend/1 - NEBfcc2octa00/1 21.696857674418425
 0.01 + NEBfcc2octaPREbegin/1 - NEBfcc2octaPREend/1 -21.6962
 0.1 + NEBocta2octaPREbegin/1 - NEBocta2octaPREend/1 0.0
 0.1 + ingaultsRu111hcp001H/1 - ingaultsRuc111hcp001/1 - H2mol/2 7.869654
 0.1 + tackfaultsRuhcpslipH/1 - stackfaultsRuhcpslip/1 - H2mol/2 -99.4654
 0.01 + ingaultsRugma7twistH/1 - ingaultsRuigma7twist/1 - H2mol/2
0.256843
 0.1 + RuadsmultiH4H/4 - trainingRuslab0001/4 - H2mol/2 -13.57685459
 0.1 + RuadsmultiH6H/6 - trainingRuslab0001/6 - H2mol/2 -12.95607547
 0.1 + RuadsmultiH8H/8 - trainingRuslab0001/8 - H2mol/2 -7.290397093
 0.1 + RuadsmultiH12H/12 - trainingRuslab0001/12 - H2mol/2 -3.96865157
 0.1 + laboddsurfHadsbcc100/1 - Ruslaboddsurfbcc100/1 - H2mol/2 -
13.40682593
 0.1 + laboddsurfHadsbcc110/1 - Ruslaboddsurfbcc110/1 - H2mol/2 -
11.53695128
 0.1 + laboddsurfHadsfcc100/1 - Ruslaboddsurffcc100/1 - H2mol/2 -
13.55227012
 0.1 + laboddsurfHadsfcc111/1 - Ruslaboddsurffcc111/1 - H2mol/2 -
5.493086163
```

```
 0.1 + Ruadsrect142rect010/1 - Ruslabrect142rect010/1 - H2mol/2 -
10.91716291
 0.1 + Ruadsrect412rect100/1 - Ruslabrect412rect100/1 - H2mol/2 -
9.402210581
 1.0 + uadsrectRu142rect010/1 - Ruslabrect142rect010/1 -
eosRuhcpRu270scana/4 9.766623663
 1.0 + uadsrectRu412rect100/1 - Ruslabrect412rect100/1 -
eosRuhcpRu270scana/4 0.84764064
 1.0 + eosRufccRufinal/4 - eosRuhcpRufinal/4 2.6583809883720733
 1.0 + eosRubccRufinal/2 - eosRuhcpRufinal/4 2.786376569767441
 1.0 + eosRufccRu380scan/4 - eosRuhcpRu270scana/4 2.718895
 1.0 + eosRubccRu300scan/2 - eosRuhcpRu270scana/4 15.13407
 1.0 + eosRuscRu250scan/1 - eosRuhcpRu270scana/4 26.03916
 1.0 + eosRuhcpRu270scana/4 - trainingRuvac01Ru/1 -182.6470753
# 1.0 + eosRufccRu380scan/4 - trainingRuvac01Ru/1 -179.9281803
# 1.0 + eosRubccRu300scan/2 - trainingRuvac01Ru/1 -167.5130047
# 1.0 + eosRuscRu250scan/1 - trainingRuvac01Ru/1 -156.6079147
 1.0 + iningdefectsRu1vac32/31 - iningdefectsRu0vac32/32 2.074844096
 1.0 + iningdefectsRu2vac32/30 - iningdefectsRu0vac32/32 3.842740891
 1.0 + iningdefectsRu3vac32/29 - iningdefectsRu0vac32/32 5.876383311
 1.0 + iningdefectsRu4vac32/28 - iningdefectsRu0vac32/32 8.157860406
 1.0 + stackfaultsRuhcpslip/16 - ingaultsRuigma7twist/112 4.80559101744
 1.0 + ingaultsRuc111hcp001/16 - ingaultsRuigma7twist/112 0.631328444767
 1.0 + ingaultsRuc111hcp001/16 - stackfaultsRuhcpslip/16 -4.17426266
 1.0 + ingaultsRuc111hcp001/16 - eosRuhcpRu270scana/4 2.110332398
 1.0 + ingaultsRuigma7twist/112 - eosRuhcpRu270scana/4 1.484458768
 1.0 + stackfaultsRuhcpslip/16 - eosRuhcpRu270scana/4 6.29005
 1.0 + Ruads1Rufcc/1 - trainingRuslab0001/1 - eosRuhcpRu270scana/4
46.12943459
 1.0 + Ruads1Rufcc/1 - Ruads1Ruhcp/1 12.129995581393814
 1.0 + Ruslabrect412rect100/32 - Ruslabrect142rect010/32 6.14267148256
 1.0 + trainingRuslab0001/42 - Ruslabrect142rect010/32 0.687977805233
 1.0 + Ruslaboddsurfbcc100/8 - Ruslaboddsurffcc100/8 9.626206104651175
 1.0 + Ruslaboddsurfbcc110/6 - Ruslaboddsurffcc100/8 10.208522945736433
 1.0 + Ruslaboddsurffcc111/9 - Ruslaboddsurffcc100/8 14.571537480620151
 1.0 + aboddsurfRuadsbcc100/9 - aboddsurfRuadsfcc100/9 8.27605506459949
 1.0 + aboddsurfRuadsbcc110/7 - aboddsurfRuadsfcc100/9 7.277257224067938
 1.0 + aboddsurfRuadsfcc111/10 - aboddsurfRuadsfcc100/9 9.71300276227393
 1.0 + md_Ru1/288 - eosRuhcpRu270scana/4 18.34075591
 1.0 + md_Ru1/288 - md_Ru2/288 9.34297933059
 1.0 + md_Ru3/288 - md_Ru2/288 1.4306061959
# 1.0 + Ru_cluster_02/2 - Ru_cluster_01/1 -85.3165108233
# 1.0 + Ru_cluster_03/3 - Ru_cluster_01/1 -103.793842986
# 1.0 + Ru_cluster_04/4 - Ru_cluster_01/1 -120.479635411
# 1.0 + Ru_cluster_05/5 - Ru_cluster_01/1 -129.174093843
# 1.0 + Ru_cluster_06/6 - Ru_cluster_01/1 -131.652965697
# 1.0 + Ru_cluster_07/7 - Ru_cluster_01/1 -136.266475598
# 1.0 + Ru_cluster_08/8 - Ru_cluster_01/1 -138.945907718
# 1.0 + Ru_cluster_07/7 - Ru_cluster_08/8 2.67943212
 20.0 + trainingdimerRu01/2 - trainingdimerRurelax/2 1362.93294837
 10.0 + trainingdimerRu02/2 - trainingdimerRurelax/2 278.682016163
 5.0 + trainingdimerRu03/2 - trainingdimerRurelax/2 52.1206319767
 1.0 + trainingdimerRu04/2 - trainingdimerRurelax/2 20.3505268605
 1.0 + trainingdimerRu05/2 - trainingdimerRurelax/2 4.35728616279
 1.0 + trainingdimerRu06/2 - trainingdimerRurelax/2 1.91922662791
 1.0 + trainingdimerRu07/2 - trainingdimerRurelax/2 8.26291104651
 1.0 + trainingdimerRu08/2 - trainingdimerRurelax/2 18.8666703488
 1.0 + trainingdimerRu09/2 - trainingdimerRurelax/2 29.1484690698
```

```
 1.0 + trainingdimerRu10/2 - trainingdimerRurelax/2 37.6317688372
 1.0 + trainingdimerRu11/2 - trainingdimerRurelax/2 40.4065289535
 1.0 + trainingdimerRu12/2 - trainingdimerRurelax/2 45.0538926744
 1.0 + trainingdimerRu13/2 - trainingdimerRurelax/2 46.8271061628
 1.0 + eosRubccRu270scan/1 - eosRubccRu305scan/1 105.19249930232553
 1.0 + eosRubccRu275scan/1 - eosRubccRu305scan/1 71.86677581395344
 1.0 + eosRubccRu280scan/1 - eosRubccRu305scan/1 46.29332395348837
 1.0 + eosRubccRu285scan/1 - eosRubccRu305scan/1 27.349629767441797
 1.0 + eosRubccRu290scan/1 - eosRubccRu305scan/1 14.048084418604617
 1.0 + eosRubccRu295scan/1 - eosRubccRu305scan/1 5.516796511627888
 1.0 + eosRubccRu300scan/1 - eosRubccRu305scan/1 0.9945039534883335
 1.0 + eosRubccRu310scan/1 - eosRubccRu305scan/1 1.4556974418604227
 1.0 + eosRubccRu315scan/1 - eosRubccRu305scan/1 5.289838837209288
 1.0 + eosRubccRu320scan/1 - eosRubccRu305scan/1 11.175882558139506
 1.0 + eosRubccRu325scan/1 - eosRubccRu305scan/1 18.348562558139463
 1.0 + eosRubccRu330scan/1 - eosRubccRu305scan/1 26.590350930232546
 1.0 + eosRubccRu335scan/1 - eosRubccRu305scan/1 35.870203488372056
 1.0 + eosRubccRu340scan/1 - eosRubccRu305scan/1 45.831606279069774
 1.0 + eosRubccRu345scan/1 - eosRubccRu305scan/1 56.282170465116224
 1.0 + eosRubccRu350scan/1 - eosRubccRu305scan/1 67.06342232558137
 1.0 + eosRufccRu350scan/1 - eosRufccRu380scan/1 95.8345574418604
 1.0 + eosRufccRu355scan/1 - eosRufccRu380scan/1 62.72908395348816
 1.0 + eosRufccRu360scan/1 - eosRufccRu380scan/1 38.03336627906958
 1.0 + eosRufccRu365scan/1 - eosRufccRu380scan/1 20.428952558139372
 1.0 + eosRufccRu370scan/1 - eosRufccRu380scan/1 8.724676976744036
 1.0 + eosRufccRu375scan/1 - eosRufccRu380scan/1 2.487710930232538
 1.0 + eosRufccRu385scan/1 - eosRufccRu380scan/1 2.265985581395171
 1.0 + eosRufccRu390scan/1 - eosRufccRu380scan/1 7.295871860465013
 1.0 + eosRufccRu395scan/1 - eosRufccRu380scan/1 15.56799372093019
 1.0 + eosRufccRu400scan/1 - eosRufccRu380scan/1 24.871130465116153
 1.0 + eosRufccRu405scan/1 - eosRufccRu380scan/1 37.16342837209288
 1.0 + eosRufccRu410scan/1 - eosRufccRu380scan/1 51.30396837209287
 1.0 + eosRufccRu415scan/1 - eosRufccRu380scan/1 66.14974697674404
 1.0 + eosRufccRu420scan/1 - eosRufccRu380scan/1 82.44308209302324
 1.0 + eosRuhcpRu250scana/1 - eosRuhcpRu270scana/1 91.99105441860445
 1.0 + eosRuhcpRu255scana/1 - eosRuhcpRu270scana/1 49.41721906976727
 1.0 + eosRuhcpRu260scana/1 - eosRuhcpRu270scana/1 21.54645069767423
 1.0 + eosRuhcpRu265scana/1 - eosRuhcpRu270scana/1 5.80954441860456
 1.0 + eosRuhcpRu275scana/1 - eosRuhcpRu270scana/1 2.234301395348666
 1.0 + eosRuhcpRu280scana/1 - eosRuhcpRu270scana/1 10.917967674418492
 1.0 + eosRuhcpRu285scana/1 - eosRuhcpRu270scana/1 24.707149302325547
 1.0 + eosRuhcpRu290scana/1 - eosRuhcpRu270scana/1 42.47813953488355
 1.0 + eosRuhcpRu295scana/1 - eosRuhcpRu270scana/1 63.29461465116265
 1.0 + eosRuhcpRu300scana/1 - eosRuhcpRu270scana/1 86.37593116279061
 1.0 + eosRuscRu220scan/1 - eosRuscRu250scan/1 51.11358558139534
 1.0 + eosRuscRu225scan/1 - eosRuscRu250scan/1 32.946869767441854
 1.0 + eosRuscRu230scan/1 - eosRuscRu250scan/1 19.56939953488373
 1.0 + eosRuscRu235scan/1 - eosRuscRu250scan/1 10.222901860465129
 1.0 + eosRuscRu240scan/1 - eosRuscRu250scan/1 4.225388139534886
 1.0 + eosRuscRu245scan/1 - eosRuscRu250scan/1 0.9868655813953353
 1.0 + eosRuscRu255scan/1 - eosRuscRu250scan/1 0.8305065116279025
 1.0 + eosRuscRu260scan/1 - eosRuscRu250scan/1 3.108178837209323
 1.0 + eosRuscRu265scan/1 - eosRuscRu250scan/1 6.524789069767422
 1.0 + eosRuscRu270scan/1 - eosRuscRu250scan/1 10.82002837209302
ENDENERGY
GEOMETRY
RuH2_angle-1 5.0 2 1 3  75.0
RuH2_angle-2 5.0 2 1 3  80.0
```

```
RuH2_angle-3 5.0 2 1 3  85.0
RuH2_angle-4 5.0 2 1 3  90.0
RuH2_angle-5 5.0 2 1 3  95.0
RuH2_angle-6 5.0 2 1 3 100.0
RuH2_angle-7 5.0 2 1 3 105.0
RuH2_angle-8 5.0 2 1 3 110.0
RuH2_angle-9 5.0 2 1 3 115.0
RuH2_angle-10 5.0 2 1 3 120.0
RuH4_length-1 0.1 1 3 1.499996255261992
RuH4_length-1 0.1 1 2 1.577289635609136
RuH4_length-1 0.1 1 5 1.577344169957844
RuH4_length-1 0.1 1 4 1.579054000374908
RuH4_length-2 0.1 1 5 1.577649421037513
RuH4_length-2 0.1 1 2 1.577706067967034
RuH4_length-2 0.1 1 4 1.577764988393392
RuH4_length-2 0.1 1 3 1.666668862551887
RuH4_length-3 0.1 1 5 1.577319255223875
RuH4_length-3 0.1 1 2 1.577389786292532
RuH4_length-3 0.1 1 4 1.57755613408842
RuH4_length-3 0.1 1 3 1.833335513374461
RuH4_length-4 0.1 1 5 1.575740680283403
RuH4_length-4 0.1 1 2 1.575745187046434
RuH4_length-4 0.1 1 4 1.575898583316833
RuH4_length-4 0.1 1 3 2.000006791288469
RuH4_length-5 0.1 1 5 1.575428174465596
RuH4_length-5 0.1 1 2 1.575459845854536
RuH4_length-5 0.1 1 4 1.575660422235705
RuH4_length-6 0.1 1 5 1.574211959711906
RuH4_length-6 0.1 1 2 1.574236144484048
RuH4_length-6 0.1 1 4 1.574501789710002
RuH4_length-7 0.1 1 2 1.573632322653548
RuH4_length-7 0.1 1 4 1.575092779806955
RuH4_length-7 0.1 1 5 1.573461574363988
RuH4_length-8 0.1 1 4 1.573058950802544
RuH4_length-8 0.1 1 2 1.574489775228788
RuH4_length-8 0.1 1 5 1.574710792304415
RuH4_length-9 0.1 1 4 1.574011061015773
RuH4_length-9 0.1 1 2 1.573734640147442
RuH4_length-9 0.1 1 5 1.57377077269849
RuH4_length-10 0.1 1 5 1.573685276032028
RuH4_length-10 0.1 1 2 1.573714957957762
RuH4_length-10 0.1 1 4 1.573891230453998
RuH4_length-1 0.1 1 3 1.5
RuH4_length-1 0.1 1 2 1.573668565997983
RuH4_length-1 0.1 1 5 1.573785913606552
RuH4_length-1 0.1 1 4 1.575086288264765
RuH4_length-2 0.1 1 2 1.573162533651702
RuH4_length-2 0.1 1 5 1.573190842474049
RuH4_length-2 0.1 1 4 1.573353705692703
RuH4_length-2 0.1 1 3 1.666666666666667
RuH4_length-3 0.1 1 2 1.572487863639681
RuH4_length-3 0.1 1 5 1.572505624308302
RuH4_length-3 0.1 1 4 1.572707260279844
RuH4_length-3 0.1 1 3 1.833333333333333
RuH4_length-4 0.1 1 2 1.570896098662439
RuH4_length-4 0.1 1 5 1.570935213071105
RuH4_length-4 0.1 1 4 1.570995233694877
RuH4_length-4 0.1 1 3 2.000000000000685
```

```
RuH4_length-5 0.1 1 2 1.570505846871478
RuH4_length-5 0.1 1 5 1.57054641308867
RuH4_length-5 0.1 1 4 1.570676916200793
RuH4_length-6 0.1 1 5 1.570520763077304
RuH4_length-6 0.1 1 4 1.57064157893765
RuH4_length-6 0.1 1 2 1.570651308647034
RuH4_length-7 0.1 1 4 1.568675096953889
RuH4_length-7 0.1 1 2 1.569058265469121
RuH4_length-7 0.1 1 5 1.570331601802776
RuH4_length-8 0.1 1 4 1.56942714277854
RuH4_length-8 0.1 1 2 1.569498480474761
RuH4_length-8 0.1 1 5 1.569273363559393
RuH4_length-9 0.1 1 2 1.569502632471784
RuH4_length-9 0.1 1 4 1.569727132848001
RuH4_length-9 0.1 1 5 1.568918765333422
RuH4_length-10 0.1 1 4 1.568920442668794
RuH4_length-10 0.1 1 5 1.569180136221553
RuH4_length-10 0.1 1 2 1.569286504160621
RuH_length-1 0.1 1 2 1.4
RuH_length-2 0.1 1 2 1.5
RuH_length-3 0.1 1 2 1.6
RuH_length-4 0.1 1 2 1.7
RuH_length-5 0.1 1 2 1.7999999999999999
RuH_length-6 0.1 1 2 1.9
RuH_length-7 0.1 1 2 2.0
ENDGEOMETRY
```
