# Supplementary material for: A ReaxFF Molecular Dynamics Study of Hydrogen Diffusion in Ruthenium–The Role of Grain Boundaries
Source: J Phys Chem C Nanomater Interfaces. 2022 Mar 23;126(13):5950–9. doi: 10.1021/acs.jpcc.1c08776 (PMC8996245; doi:10.1021/acs.jpcc.1c08776)
Supplement: Supplementary file 2 — jp1c08776_si_002.pdf [file jp1c08776_si_002.pdf]

```
XTLGRF 200
DESCRP eosRubccRu270scan
RUTYPE NORMAL RUN
#refdata /ortho_train/Ru/eos_Ru/bcc_Ru/2.70_scan
REMARK Created by geo_energy_extract.py EO = -12.72985906 eV; -
296.0432339534884 kcal/mol
CRYSTX     2.70000    2.70000    2.70000   90.00000   90.00000   90.00000
HETATM     1 Ru                  0.00000   0.00000   0.00000   Ru1  1 1
0.00000
HETATM     2 Ru                  1.35000   1.35000   1.35000   Ru1  1 1
0.00000
UNIT ENERGY   kcal
ENERGY        -296.0432339534884
END

XTLGRF 200
DESCRP eosRubccRu275scan
RUTYPE NORMAL RUN
#refdata /ortho_train/Ru/eos_Ru/bcc_Ru/2.75_scan
REMARK Created by geo_energy_extract.py EO = -14.16286517 eV; -
329.3689574418605 kcal/mol
CRYSTX     2.75000    2.75000    2.75000   90.00000   90.00000   90.00000
HETATM     1 Ru                  0.00000   0.00000   0.00000   Ru1  1 1
0.00000
HETATM     2 Ru                  1.37500   1.37500   1.37500   Ru1  1 1
0.00000
UNIT ENERGY   kcal
ENERGY        -329.3689574418605
END

XTLGRF 200
DESCRP eosRubccRu280scan
RUTYPE NORMAL RUN
#refdata /ortho_train/Ru/eos_Ru/bcc_Ru/2.80_scan
REMARK Created by geo_energy_extract.py EO = -15.26252360 eV; -
354.9424093023256 kcal/mol
CRYSTX     2.80000    2.80000    2.80000   90.00000   90.00000   90.00000
HETATM     1 Ru                  0.00000   0.00000   0.00000   Ru1  1 1
0.00000
HETATM     2 Ru                  1.40000   1.40000   1.40000   Ru1  1 1
0.00000
UNIT ENERGY   kcal
ENERGY        -354.9424093023256
END

XTLGRF 200
DESCRP eosRubccRu285scan
RUTYPE NORMAL RUN
#refdata /ortho_train/Ru/eos_Ru/bcc_Ru/2.85_scan
REMARK Created by geo_energy_extract.py EO = -16.07710245 eV; -
373.88610348837216 kcal/mol
CRYSTX     2.85000    2.85000    2.85000   90.00000   90.00000   90.00000
HETATM     1 Ru                  0.00000   0.00000   0.00000   Ru1  1 1
0.00000
HETATM     2 Ru                  1.42500   1.42500   1.42500   Ru1  1 1
0.00000
UNIT ENERGY   kcal
ENERGY        -373.88610348837216
```

```
END

XTLGRF 200
DESCRP eosRubccRu290scan
RUTYPE NORMAL RUN
#refdata /ortho_train/Ru/eos_Ru/bcc_Ru/2.90_scan
REMARK Created by geo_energy_extract.py EO = -16.64906890 eV; -
387.18764883720934 kcal/mol
CRYSTX     2.90000    2.90000    2.90000   90.00000   90.00000   90.00000
HETATM     1 Ru                  0.00000   0.00000   0.00000   Ru1  1 1
0.00000
HETATM     2 Ru                  1.45000   1.45000   1.45000   Ru1  1 1
0.00000
UNIT ENERGY   kcal
ENERGY        -387.18764883720934
END

XTLGRF 200
DESCRP eosRubccRu295scan
RUTYPE NORMAL RUN
#refdata /ortho_train/Ru/eos_Ru/bcc_Ru/2.95_scan
REMARK Created by geo_energy_extract.py EO = -17.01591428 eV; -
395.71893674418607 kcal/mol
CRYSTX     2.95000    2.95000    2.95000   90.00000   90.00000   90.00000
HETATM     1 Ru                  0.00000   0.00000   0.00000   Ru1  1 1
0.00000
HETATM     2 Ru                  1.47500   1.47500   1.47500   Ru1  1 1
0.00000
UNIT ENERGY   kcal
ENERGY        -395.71893674418607
END

XTLGRF 200
DESCRP eosRubccRu300scan
RUTYPE NORMAL RUN
#refdata /ortho_train/Ru/eos_Ru/bcc_Ru/3.00_scan
REMARK Created by geo_energy_extract.py EO = -17.21037286 eV; -
400.2412293023256 kcal/mol
CRYSTX     3.00000    3.00000    3.00000   90.00000   90.00000   90.00000
HETATM     1 Ru                  0.00000   0.00000   0.00000   Ru1  1 1
0.00000
HETATM     2 Ru                  1.50000   1.50000   1.50000   Ru1  1 1
0.00000
UNIT ENERGY   kcal
ENERGY        -400.2412293023256
END

XTLGRF 200
DESCRP eosRubccRu305scan
RUTYPE NORMAL RUN
#refdata /ortho_train/Ru/eos_Ru/bcc_Ru/3.05_scan
REMARK Created by geo_energy_extract.py EO = -17.25313653 eV; -
401.23573325581395 kcal/mol
CRYSTX     3.05000    3.05000    3.05000   90.00000   90.00000   90.00000
HETATM     1 Ru                  0.00000   0.00000   0.00000   Ru1  1 1
0.00000
HETATM     2 Ru                  1.52500   1.52500   1.52500   Ru1  1 1
0.00000
```

```
UNIT ENERGY   kcal
ENERGY        -401.23573325581395
END

XTLGRF 200
DESCRP eosRubccRu310scan
RUTYPE NORMAL RUN
#refdata /ortho_train/Ru/eos_Ru/bcc_Ru/3.10_scan
REMARK Created by geo_energy_extract.py EO = -17.19054154 eV; -
399.78003581395353 kcal/mol
CRYSTX     3.10000    3.10000    3.10000   90.00000   90.00000   90.00000
HETATM     1 Ru                  0.00000   0.00000   0.00000   Ru1  1 1
0.00000
HETATM     2 Ru                  1.55000   1.55000   1.55000   Ru1  1 1
0.00000
UNIT ENERGY   kcal
ENERGY        -399.78003581395353
END

XTLGRF 200
DESCRP eosRubccRu315scan
RUTYPE NORMAL RUN
#refdata /ortho_train/Ru/eos_Ru/bcc_Ru/3.15_scan
REMARK Created by geo_energy_extract.py EO = -17.02567346 eV; -
395.94589441860467 kcal/mol
CRYSTX     3.15000    3.15000    3.15000   90.00000   90.00000   90.00000
HETATM     1 Ru                  0.00000   0.00000   0.00000   Ru1  1 1
0.00000
HETATM     2 Ru                  1.57500   1.57500   1.57500   Ru1  1 1
0.00000
UNIT ENERGY   kcal
ENERGY        -395.94589441860467
END

XTLGRF 200
DESCRP eosRubccRu320scan
RUTYPE NORMAL RUN
#refdata /ortho_train/Ru/eos_Ru/bcc_Ru/3.20_scan
REMARK Created by geo_energy_extract.py EO = -16.77257358 eV; -
390.05985069767445 kcal/mol
CRYSTX     3.20000    3.20000    3.20000   90.00000   90.00000   90.00000
HETATM     1 Ru                  0.00000   0.00000   0.00000   Ru1  1 1
0.00000
HETATM     2 Ru                  1.60000   1.60000   1.60000   Ru1  1 1
0.00000
UNIT ENERGY   kcal
ENERGY        -390.05985069767445
END

XTLGRF 200
DESCRP eosRubccRu325scan
RUTYPE NORMAL RUN
#refdata /ortho_train/Ru/eos_Ru/bcc_Ru/3.25_scan
REMARK Created by geo_energy_extract.py EO = -16.46414834 eV; -
382.8871706976745 kcal/mol
CRYSTX     3.25000    3.25000    3.25000   90.00000   90.00000   90.00000
HETATM     1 Ru                  0.00000   0.00000   0.00000   Ru1  1 1
0.00000
```

```
HETATM     2 Ru                 1.62500   1.62500   1.62500   Ru1  1 1
0.00000
UNIT ENERGY   kcal
ENERGY        -382.8871706976745
END

XTLGRF 200
DESCRP eosRubccRu330scan
RUTYPE NORMAL RUN
#refdata /ortho_train/Ru/eos_Ru/bcc_Ru/3.30_scan
REMARK Created by geo_energy_extract.py EO = -16.10975144 eV; -
374.6453823255814 kcal/mol
CRYSTX     3.30000    3.30000    3.30000   90.00000   90.00000   90.00000
HETATM     1 Ru                 0.00000   0.00000   0.00000   Ru1  1 1
0.00000
HETATM     2 Ru                 1.65000   1.65000   1.65000   Ru1  1 1
0.00000
UNIT ENERGY   kcal
ENERGY        -374.6453823255814
END

XTLGRF 200
DESCRP eosRubccRu335scan
RUTYPE NORMAL RUN
#refdata /ortho_train/Ru/eos_Ru/bcc_Ru/3.35_scan
REMARK Created by geo_energy_extract.py EO = -15.71071778 eV; -
365.3655297674419 kcal/mol
CRYSTX     3.35000    3.35000    3.35000   90.00000   90.00000   90.00000
HETATM     1 Ru                 0.00000   0.00000   0.00000   Ru1  1 1
0.00000
HETATM     2 Ru                 1.67500   1.67500   1.67500   Ru1  1 1
0.00000
UNIT ENERGY   kcal
ENERGY        -365.3655297674419
END

XTLGRF 200
DESCRP eosRubccRu340scan
RUTYPE NORMAL RUN
#refdata /ortho_train/Ru/eos_Ru/bcc_Ru/3.40_scan
REMARK Created by geo_energy_extract.py EO = -15.28237746 eV; -
355.4041269767442 kcal/mol
CRYSTX     3.40000    3.40000    3.40000   90.00000   90.00000   90.00000
HETATM     1 Ru                 0.00000   0.00000   0.00000   Ru1  1 1
0.00000
HETATM     2 Ru                 1.70000   1.70000   1.70000   Ru1  1 1
0.00000
UNIT ENERGY   kcal
ENERGY        -355.4041269767442
END

XTLGRF 200
DESCRP eosRubccRu345scan
RUTYPE NORMAL RUN
#refdata /ortho_train/Ru/eos_Ru/bcc_Ru/3.45_scan
REMARK Created by geo_energy_extract.py EO = -14.83300320 eV; -
344.95356279069773 kcal/mol
CRYSTX     3.45000    3.45000    3.45000   90.00000   90.00000   90.00000
```

```
HETATM     1 Ru                    0.00000   0.00000   0.00000   Ru1  1 1
0.00000
HETATM     2 Ru                    1.72500   1.72500   1.72500   Ru1  1 1
0.00000
UNIT ENERGY   kcal
ENERGY        -344.95356279069773
END

XTLGRF 200
DESCRP eosRubccRu350scan
RUTYPE NORMAL RUN
#refdata /ortho_train/Ru/eos_Ru/bcc_Ru/3.50_scan
REMARK Created by geo_energy_extract.py EO = -14.36940937 eV; -
334.1723109302326 kcal/mol
CRYSTX     3.50000    3.50000    3.50000   90.00000   90.00000   90.00000
HETATM     1 Ru                    0.00000   0.00000   0.00000   Ru1  1 1
0.00000
HETATM     2 Ru                    1.75000   1.75000   1.75000   Ru1  1 1
0.00000
UNIT ENERGY   kcal
ENERGY        -334.1723109302326
END

XTLGRF 200
DESCRP eosRufccRu350scan
RUTYPE NORMAL RUN
#refdata /ortho_train/Ru/eos_Ru/fcc_Ru/3.50_scan
REMARK Created by geo_energy_extract.py EO = -32.43526997 eV; -
754.3086039534884 kcal/mol
CRYSTX     3.50000    3.50000    3.50000   90.00000   90.00000   90.00000
HETATM     1 Ru                    2.62500   2.62500   0.87500   Ru1  1 1
0.00000
HETATM     2 Ru                    2.62500   0.87500   2.62500   Ru1  1 1
0.00000
HETATM     3 Ru                    0.87500   2.62500   2.62500   Ru1  1 1
0.00000
HETATM     4 Ru                    0.87500   0.87500   0.87500   Ru1  1 1
0.00000
UNIT ENERGY   kcal
ENERGY        -754.3086039534884
END

XTLGRF 200
DESCRP eosRufccRu355scan
RUTYPE NORMAL RUN
#refdata /ortho_train/Ru/eos_Ru/fcc_Ru/3.55_scan
REMARK Created by geo_energy_extract.py EO = -33.85880533 eV; -
787.4140774418606 kcal/mol
CRYSTX     3.55000    3.55000    3.55000   90.00000   90.00000   90.00000
HETATM     1 Ru                    2.66250   2.66250   0.88750   Ru1  1 1
0.00000
HETATM     2 Ru                    2.66250   0.88750   2.66250   Ru1  1 1
0.00000
HETATM     3 Ru                    0.88750   2.66250   2.66250   Ru1  1 1
0.00000
HETATM     4 Ru                    0.88750   0.88750   0.88750   Ru1  1 1
0.00000
UNIT ENERGY   kcal
```

```
ENERGY        -787.4140774418606
END

XTLGRF 200
DESCRP eosRufccRu360scan
RUTYPE NORMAL RUN
#refdata /ortho_train/Ru/eos_Ru/fcc_Ru/3.60_scan
REMARK Created by geo_energy_extract.py EO = -34.92072119 eV; -
812.1097951162792 kcal/mol
CRYSTX     3.60000    3.60000    3.60000   90.00000   90.00000   90.00000
HETATM     1 Ru                  2.70000   2.70000   0.90000   Ru1  1 1
0.00000
HETATM     2 Ru                  2.70000   0.90000   2.70000   Ru1  1 1
0.00000
HETATM     3 Ru                  0.90000   2.70000   2.70000   Ru1  1 1
0.00000
HETATM     4 Ru                  0.90000   0.90000   0.90000   Ru1  1 1
0.00000
UNIT ENERGY   kcal
ENERGY        -812.1097951162792
END

XTLGRF 200
DESCRP eosRufccRu365scan
RUTYPE NORMAL RUN
#refdata /ortho_train/Ru/eos_Ru/fcc_Ru/3.65_scan
REMARK Created by geo_energy_extract.py EO = -35.67771098 eV; -
829.7142088372094 kcal/mol
CRYSTX     3.65000    3.65000    3.65000   90.00000   90.00000   90.00000
HETATM     1 Ru                  2.73750   2.73750   0.91250   Ru1  1 1
0.00000
HETATM     2 Ru                  2.73750   0.91250   2.73750   Ru1  1 1
0.00000
HETATM     3 Ru                  0.91250   2.73750   2.73750   Ru1  1 1
0.00000
HETATM     4 Ru                  0.91250   0.91250   0.91250   Ru1  1 1
0.00000
UNIT ENERGY   kcal
ENERGY        -829.7142088372094
END

XTLGRF 200
DESCRP eosRufccRu370scan
RUTYPE NORMAL RUN
#refdata /ortho_train/Ru/eos_Ru/fcc_Ru/3.70_scan
REMARK Created by geo_energy_extract.py EO = -36.18099483 eV; -
841.4184844186047 kcal/mol
CRYSTX     3.70000    3.70000    3.70000   90.00000   90.00000   90.00000
HETATM     1 Ru                  2.77500   2.77500   0.92500   Ru1  1 1
0.00000
HETATM     2 Ru                  2.77500   0.92500   2.77500   Ru1  1 1
0.00000
HETATM     3 Ru                  0.92500   2.77500   2.77500   Ru1  1 1
0.00000
HETATM     4 Ru                  0.92500   0.92500   0.92500   Ru1  1 1
0.00000
UNIT ENERGY   kcal
ENERGY        -841.4184844186047
```

END

XTLGRF 200
DESCRP eosRufccRu375scan
RUTYPE NORMAL RUN
#refdata /ortho_train/Ru/eos_Ru/fcc_Ru/3.75_scan
REMARK Created by geo_energy_extract.py EO = -36.44918437 eV; -
847.6554504651162 kcal/mol
CRYSTX 3.75000 3.75000 3.75000 90.00000 90.00000 90.00000
HETATM 1 Ru 2.81250 2.81250 0.93750 Ru1 1 1
0.00000
HETATM 2 Ru 2.81250 0.93750 2.81250 Ru1 1 1
0.00000
HETATM 3 Ru 0.93750 2.81250 2.81250 Ru1 1 1
0.00000
HETATM 4 Ru 0.93750 0.93750 0.93750 Ru1 1 1
0.00000
UNIT ENERGY kcal
ENERGY -847.6554504651162
END

XTLGRF 200
DESCRP eosRufccRu380scan
RUTYPE NORMAL RUN
#refdata /ortho_train/Ru/eos_Ru/fcc_Ru/3.80_scan
REMARK Created by geo_energy_extract.py EO = -36.55615594 eV; -
850.1431613953488 kcal/mol
CRYSTX 3.80000 3.80000 3.80000 90.00000 90.00000 90.00000
HETATM 1 Ru 2.85000 2.85000 0.95000 Ru1 1 1
0.00000
HETATM 2 Ru 2.85000 0.95000 2.85000 Ru1 1 1
0.00000
HETATM 3 Ru 0.95000 2.85000 2.85000 Ru1 1 1
0.00000
HETATM 4 Ru 0.95000 0.95000 0.95000 Ru1 1 1
0.00000
UNIT ENERGY kcal
ENERGY -850.1431613953488
END

XTLGRF 200
DESCRP eosRufccRu385scan
RUTYPE NORMAL RUN
#refdata /ortho_train/Ru/eos_Ru/fcc_Ru/3.85_scan
REMARK Created by geo_energy_extract.py EO = -36.45871856 eV; -
847.8771758139536 kcal/mol
CRYSTX 3.85000 3.85000 3.85000 90.00000 90.00000 90.00000
HETATM 1 Ru 2.88750 2.88750 0.96250 Ru1 1 1
0.00000
HETATM 2 Ru 2.88750 0.96250 2.88750 Ru1 1 1
0.00000
HETATM 3 Ru 0.96250 2.88750 2.88750 Ru1 1 1
0.00000
HETATM 4 Ru 0.96250 0.96250 0.96250 Ru1 1 1
0.00000
UNIT ENERGY kcal
ENERGY -847.8771758139536
END

```
XTLGRF 200
DESCRP eosRufccRu390scan
RUTYPE NORMAL RUN
#refdata /ortho_train/Ru/eos_Ru/fcc_Ru/3.90_scan
REMARK Created by geo_energy_extract.py EO = -36.24243345 eV; -
842.8472895348838 kcal/mol
CRYSTX     3.90000    3.90000    3.90000   90.00000   90.00000   90.00000
HETATM     1 Ru                  2.92500   2.92500   0.97500   Ru1  1 1
0.00000
HETATM     2 Ru                  2.92500   0.97500   2.92500   Ru1  1 1
0.00000
HETATM     3 Ru                  0.97500   2.92500   2.92500   Ru1  1 1
0.00000
HETATM     4 Ru                  0.97500   0.97500   0.97500   Ru1  1 1
0.00000
UNIT ENERGY   kcal
ENERGY        -842.8472895348838
END

XTLGRF 200
DESCRP eosRufccRu395scan
RUTYPE NORMAL RUN
#refdata /ortho_train/Ru/eos_Ru/fcc_Ru/3.95_scan
REMARK Created by geo_energy_extract.py EO = -35.88673221 eV; -
834.5751676744186 kcal/mol
CRYSTX     3.95000    3.95000    3.95000   90.00000   90.00000   90.00000
HETATM     1 Ru                  2.96250   2.96250   0.98750   Ru1  1 1
0.00000
HETATM     2 Ru                  2.96250   0.98750   2.96250   Ru1  1 1
0.00000
HETATM     3 Ru                  0.98750   2.96250   2.96250   Ru1  1 1
0.00000
HETATM     4 Ru                  0.98750   0.98750   0.98750   Ru1  1 1
0.00000
UNIT ENERGY   kcal
ENERGY        -834.5751676744186
END

XTLGRF 200
DESCRP eosRufccRu400scan
RUTYPE NORMAL RUN
#refdata /ortho_train/Ru/eos_Ru/fcc_Ru/4.00_scan
REMARK Created by geo_energy_extract.py EO = -35.48669733 eV; -
825.2720309302326 kcal/mol
CRYSTX     4.00000    4.00000    4.00000   90.00000   90.00000   90.00000
HETATM     1 Ru                  3.00000   3.00000   1.00000   Ru1  1 1
0.00000
HETATM     2 Ru                  3.00000   1.00000   3.00000   Ru1  1 1
0.00000
HETATM     3 Ru                  1.00000   3.00000   3.00000   Ru1  1 1
0.00000
HETATM     4 Ru                  1.00000   1.00000   1.00000   Ru1  1 1
0.00000
UNIT ENERGY   kcal
ENERGY        -825.2720309302326
END
```

```
XTLGRF 200
DESCRP eosRufccRu405scan
RUTYPE NORMAL RUN
#refdata /ortho_train/Ru/eos_Ru/fcc_Ru/4.05_scan
REMARK Created by geo_energy_extract.py EO = -34.95812852 eV; -
812.9797330232559 kcal/mol
CRYSTX     4.05000    4.05000    4.05000   90.00000   90.00000   90.00000
HETATM     1 Ru                  3.03750   3.03750   1.01250   Ru1  1 1
0.00000
HETATM     2 Ru                  3.03750   1.01250   3.03750   Ru1  1 1
0.00000
HETATM     3 Ru                  1.01250   3.03750   3.03750   Ru1  1 1
0.00000
HETATM     4 Ru                  1.01250   1.01250   1.01250   Ru1  1 1
0.00000
UNIT ENERGY   kcal
ENERGY        -812.9797330232559
END

XTLGRF 200
DESCRP eosRufccRu410scan
RUTYPE NORMAL RUN
#refdata /ortho_train/Ru/eos_Ru/fcc_Ru/4.10_scan
REMARK Created by geo_energy_extract.py EO = -34.35008530 eV; -
798.8391930232559 kcal/mol
CRYSTX     4.10000    4.10000    4.10000   90.00000   90.00000   90.00000
HETATM     1 Ru                  3.07500   3.07500   1.02500   Ru1  1 1
0.00000
HETATM     2 Ru                  3.07500   1.02500   3.07500   Ru1  1 1
0.00000
HETATM     3 Ru                  1.02500   3.07500   3.07500   Ru1  1 1
0.00000
HETATM     4 Ru                  1.02500   1.02500   1.02500   Ru1  1 1
0.00000
UNIT ENERGY   kcal
ENERGY        -798.8391930232559
END

XTLGRF 200
DESCRP eosRufccRu415scan
RUTYPE NORMAL RUN
#refdata /ortho_train/Ru/eos_Ru/fcc_Ru/4.15_scan
REMARK Created by geo_energy_extract.py EO = -33.71171682 eV; -
783.9934144186047 kcal/mol
CRYSTX     4.15000    4.15000    4.15000   90.00000   90.00000   90.00000
HETATM     1 Ru                  3.11250   3.11250   1.03750   Ru1  1 1
0.00000
HETATM     2 Ru                  3.11250   1.03750   3.11250   Ru1  1 1
0.00000
HETATM     3 Ru                  1.03750   3.11250   3.11250   Ru1  1 1
0.00000
HETATM     4 Ru                  1.03750   1.03750   1.03750   Ru1  1 1
0.00000
UNIT ENERGY   kcal
ENERGY        -783.9934144186047
END

XTLGRF 200
```

```
DESCRP eosRufccRu420scan
RUTYPE NORMAL RUN
#refdata /ortho_train/Ru/eos_Ru/fcc_Ru/4.20_scan
REMARK Created by geo_energy_extract.py EO = -33.01110341 eV; -
767.7000793023255 kcal/mol
CRYSTX     4.20000    4.20000    4.20000   90.00000   90.00000   90.00000
HETATM     1 Ru                  3.15000   3.15000   1.05000   Ru1  1 1
0.00000
HETATM     2 Ru                  3.15000   1.05000   3.15000   Ru1  1 1
0.00000
HETATM     3 Ru                  1.05000   3.15000   3.15000   Ru1  1 1
0.00000
HETATM     4 Ru                  1.05000   1.05000   1.05000   Ru1  1 1
0.00000
UNIT ENERGY   kcal
ENERGY        -767.7000793023255
END

XTLGRF 200
DESCRP eosRuhcpRu250scana
RUTYPE NORMAL RUN
#refdata /ortho_train/Ru/eos_Ru/hcp_Ru/2.50_scan_a
REMARK Created by geo_energy_extract.py EO = -33.06819053 eV; -
769.0276867441862 kcal/mol
CRYSTX     2.50000    4.33012    3.96297   90.00000   90.00000   90.00000
HETATM     1 Ru                  0.00000   1.44132   0.99074   Ru1  1 1
0.00000
HETATM     2 Ru                  1.25000   3.60639   0.99074   Ru1  1 1
0.00000
HETATM     3 Ru                  1.25000   0.72373   2.97223   Ru1  1 1
0.00000
HETATM     4 Ru                  0.00000   2.88880   2.97223   Ru1  1 1
0.00000
UNIT ENERGY   kcal
ENERGY        -769.0276867441862
END

XTLGRF 200
DESCRP eosRuhcpRu255scana
RUTYPE NORMAL RUN
#refdata /ortho_train/Ru/eos_Ru/hcp_Ru/2.55_scan_a
REMARK Created by geo_energy_extract.py EO = -34.89886545 eV; -
811.6015220930234 kcal/mol
CRYSTX     2.55000    4.41673    4.04223   90.00000   90.00000   90.00000
HETATM     1 Ru                  0.00000   1.46989   1.01056   Ru1  1 1
0.00000
HETATM     2 Ru                  1.27500   3.67826   1.01056   Ru1  1 1
0.00000
HETATM     3 Ru                  1.27500   0.73846   3.03167   Ru1  1 1
0.00000
HETATM     4 Ru                  0.00000   2.94683   3.03167   Ru1  1 1
0.00000
UNIT ENERGY   kcal
ENERGY        -811.6015220930234
END

XTLGRF 200
DESCRP eosRuhcpRu260scana
```

```
RUTYPE NORMAL RUN
#refdata /ortho_train/Ru/eos_Ru/hcp_Ru/2.60_scan_a
REMARK Created by geo_energy_extract.py EO = -36.09730849 eV; -
839.4722904651164 kcal/mol
CRYSTX     2.60000    4.50333    4.12149   90.00000   90.00000   90.00000
HETATM     1 Ru                  0.00000   1.49903   1.03037   Ru1  1 1
0.00000
HETATM     2 Ru                  1.30000   3.75070   1.03037   Ru1  1 1
0.00000
HETATM     3 Ru                  1.30000   0.75263   3.09112   Ru1  1 1
0.00000
HETATM     4 Ru                  0.00000   3.00431   3.09112   Ru1  1 1
0.00000
UNIT ENERGY   kcal
ENERGY        -839.4722904651164
END

XTLGRF 200
DESCRP eosRuhcpRu265scana
RUTYPE NORMAL RUN
#refdata /ortho_train/Ru/eos_Ru/hcp_Ru/2.65_scan_a
REMARK Created by geo_energy_extract.py EO = -36.77399546 eV; -
855.2091967441861 kcal/mol
CRYSTX     2.65000    4.58993    4.20075   90.00000   90.00000   90.00000
HETATM     1 Ru                  0.00000   1.52810   1.05019   Ru1  1 1
0.00000
HETATM     2 Ru                  1.32500   3.82308   1.05019   Ru1  1 1
0.00000
HETATM     3 Ru                  1.32500   0.76686   3.15056   Ru1  1 1
0.00000
HETATM     4 Ru                  0.00000   3.06182   3.15056   Ru1  1 1
0.00000
UNIT ENERGY   kcal
ENERGY        -855.2091967441861
END

XTLGRF 200
DESCRP eosRuhcpRu270scana
RUTYPE NORMAL RUN
#refdata /ortho_train/Ru/eos_Ru/hcp_Ru/2.70_scan_a
REMARK Created by geo_energy_extract.py EO = -37.02380587 eV; -
861.0187411627907 kcal/mol
CRYSTX     2.70000    4.67654    4.28001   90.00000   90.00000   90.00000
HETATM     1 Ru                  0.00000   1.55718   1.07000   Ru1  1 1
0.00000
HETATM     2 Ru                  1.35000   3.89546   1.07000   Ru1  1 1
0.00000
HETATM     3 Ru                  1.35000   0.78109   3.21001   Ru1  1 1
0.00000
HETATM     4 Ru                  0.00000   3.11935   3.21001   Ru1  1 1
0.00000
UNIT ENERGY   kcal
ENERGY        -861.0187411627907
END

XTLGRF 200
DESCRP eosRuhcpRu275scana
RUTYPE NORMAL RUN
```

```
#refdata /ortho_train/Ru/eos_Ru/hcp_Ru/2.75_scan_a
REMARK Created by geo_energy_extract.py EO = -36.92773091 eV; -
858.784439767442 kcal/mol
CRYSTX     2.75000    4.76314    4.35927   90.00000   90.00000   90.00000
HETATM     1 Ru                  0.00000   1.58627   1.08982   Ru1  1 1
0.00000
HETATM     2 Ru                  1.37500   3.96785   1.08982   Ru1  1 1
0.00000
HETATM     3 Ru                  1.37500   0.79530   3.26945   Ru1  1 1
0.00000
HETATM     4 Ru                  0.00000   3.17686   3.26945   Ru1  1 1
0.00000
UNIT ENERGY   kcal
ENERGY        -858.784439767442
END

XTLGRF 200
DESCRP eosRuhcpRu280scana
RUTYPE NORMAL RUN
#refdata /ortho_train/Ru/eos_Ru/hcp_Ru/2.80_scan_a
REMARK Created by geo_energy_extract.py EO = -36.55433326 eV; -
850.1007734883722 kcal/mol
CRYSTX     2.80000    4.84974    4.43853   90.00000   90.00000   90.00000
HETATM     1 Ru                  0.00000   1.61536   1.10963   Ru1  1 1
0.00000
HETATM     2 Ru                  1.40000   4.04023   1.10963   Ru1  1 1
0.00000
HETATM     3 Ru                  1.40000   0.80951   3.32890   Ru1  1 1
0.00000
HETATM     4 Ru                  0.00000   3.23438   3.32890   Ru1  1 1
0.00000
UNIT ENERGY   kcal
ENERGY        -850.1007734883722
END

XTLGRF 200
DESCRP eosRuhcpRu285scana
RUTYPE NORMAL RUN
#refdata /ortho_train/Ru/eos_Ru/hcp_Ru/2.85_scan_a
REMARK Created by geo_energy_extract.py EO = -35.96139845 eV; -
836.3115918604651 kcal/mol
CRYSTX     2.85000    4.93634    4.51779   90.00000   90.00000   90.00000
HETATM     1 Ru                  0.00000   1.64442   1.12945   Ru1  1 1
0.00000
HETATM     2 Ru                  1.42500   4.11259   1.12945   Ru1  1 1
0.00000
HETATM     3 Ru                  1.42500   0.82375   3.38834   Ru1  1 1
0.00000
HETATM     4 Ru                  0.00000   3.29192   3.38834   Ru1  1 1
0.00000
UNIT ENERGY   kcal
ENERGY        -836.3115918604651
END

XTLGRF 200
DESCRP eosRuhcpRu290scana
RUTYPE NORMAL RUN
#refdata /ortho_train/Ru/eos_Ru/hcp_Ru/2.90_scan_a
```

```
REMARK Created by geo_energy_extract.py EO = -35.19724587 eV; -
818.5406016279071 kcal/mol
CRYSTX     2.90000    5.02294    4.59705   90.00000   90.00000   90.00000
HETATM     1 Ru                  0.00000   1.67346   1.14926   Ru1  1 1
0.00000
HETATM     2 Ru                  1.45000   4.18494   1.14926   Ru1  1 1
0.00000
HETATM     3 Ru                  1.45000   0.83801   3.44779   Ru1  1 1
0.00000
HETATM     4 Ru                  0.00000   3.34948   3.44779   Ru1  1 1
0.00000
UNIT ENERGY   kcal
ENERGY        -818.5406016279071
END

XTLGRF 200
DESCRP eosRuhcpRu295scana
RUTYPE NORMAL RUN
#refdata /ortho_train/Ru/eos_Ru/hcp_Ru/2.95_scan_a
REMARK Created by geo_energy_extract.py EO = -34.30213744 eV; -
797.724126511628 kcal/mol
CRYSTX     2.95000    5.10955    4.67631   90.00000   90.00000   90.00000
HETATM     1 Ru                  0.00000   1.70248   1.16908   Ru1  1 1
0.00000
HETATM     2 Ru                  1.47500   4.25726   1.16908   Ru1  1 1
0.00000
HETATM     3 Ru                  1.47500   0.85229   3.50723   Ru1  1 1
0.00000
HETATM     4 Ru                  0.00000   3.40706   3.50723   Ru1  1 1
0.00000
UNIT ENERGY   kcal
ENERGY        -797.724126511628
END

XTLGRF 200
DESCRP eosRuhcpRu300scana
RUTYPE NORMAL RUN
#refdata /ortho_train/Ru/eos_Ru/hcp_Ru/3.00_scan_a
REMARK Created by geo_energy_extract.py EO = -33.30964083 eV; -774.64281
kcal/mol
CRYSTX     3.00000    5.19615    4.75557   90.00000   90.00000   90.00000
HETATM     1 Ru                  0.00000   1.73182   1.18889   Ru1  1 1
0.00000
HETATM     2 Ru                  1.50000   4.32990   1.18889   Ru1  1 1
0.00000
HETATM     3 Ru                  1.50000   0.86625   3.56668   Ru1  1 1
0.00000
HETATM     4 Ru                  0.00000   3.46432   3.56668   Ru1  1 1
0.00000
UNIT ENERGY   kcal
ENERGY        -774.64281
END

XTLGRF 200
DESCRP eosRuscRu220scan
RUTYPE NORMAL RUN
#refdata /ortho_train/Ru/eos_Ru/sc_Ru/2.20_scan
```

```
REMARK Created by geo_energy_extract.py EO = -5.93838338 eV; -
138.10193906976747 kcal/mol
CRYSTX     2.20000    2.20000    2.20000   90.00000   90.00000   90.00000
HETATM     1 Ru                  0.00000   0.00000   0.00000   Ru1  1 1
0.00000
UNIT ENERGY   kcal
ENERGY        -138.10193906976747
END

XTLGRF 200
DESCRP eosRuscRu225scan
RUTYPE NORMAL RUN
#refdata /ortho_train/Ru/eos_Ru/sc_Ru/2.25_scan
REMARK Created by geo_energy_extract.py EO = -6.71955216 eV; -
156.26865488372096 kcal/mol
CRYSTX     2.25000    2.25000    2.25000   90.00000   90.00000   90.00000
HETATM     1 Ru                  0.00000   0.00000   0.00000   Ru1  1 1
0.00000
UNIT ENERGY   kcal
ENERGY        -156.26865488372096
END

XTLGRF 200
DESCRP eosRuscRu230scan
RUTYPE NORMAL RUN
#refdata /ortho_train/Ru/eos_Ru/sc_Ru/2.30_scan
REMARK Created by geo_energy_extract.py EO = -7.29478338 eV; -
169.64612511627908 kcal/mol
CRYSTX     2.30000    2.30000    2.30000   90.00000   90.00000   90.00000
HETATM     1 Ru                  0.00000   0.00000   0.00000   Ru1  1 1
0.00000
UNIT ENERGY   kcal
ENERGY        -169.64612511627908
END

XTLGRF 200
DESCRP eosRuscRu235scan
RUTYPE NORMAL RUN
#refdata /ortho_train/Ru/eos_Ru/sc_Ru/2.35_scan
REMARK Created by geo_energy_extract.py EO = -7.69668278 eV; -
178.99262279069768 kcal/mol
CRYSTX     2.35000    2.35000    2.35000   90.00000   90.00000   90.00000
HETATM     1 Ru                  0.00000   0.00000   0.00000   Ru1  1 1
0.00000
UNIT ENERGY   kcal
ENERGY        -178.99262279069768
END

XTLGRF 200
DESCRP eosRuscRu240scan
RUTYPE NORMAL RUN
#refdata /ortho_train/Ru/eos_Ru/sc_Ru/2.40_scan
REMARK Created by geo_energy_extract.py EO = -7.95457587 eV; -
184.99013651162792 kcal/mol
CRYSTX     2.40000    2.40000    2.40000   90.00000   90.00000   90.00000
HETATM     1 Ru                  0.00000   0.00000   0.00000   Ru1  1 1
0.00000
UNIT ENERGY   kcal
```

```
ENERGY        -184.99013651162792
END

XTLGRF 200
DESCRP eosRuscRu245scan
RUTYPE NORMAL RUN
#refdata /ortho_train/Ru/eos_Ru/sc_Ru/2.45_scan
REMARK Created by geo_energy_extract.py EO = -8.09383234 eV; -
188.22865906976747 kcal/mol
CRYSTX     2.45000    2.45000    2.45000   90.00000   90.00000   90.00000
HETATM     1 Ru                  0.00000   0.00000   0.00000   Ru1  1 1
0.00000
UNIT ENERGY   kcal
ENERGY        -188.22865906976747
END

XTLGRF 200
DESCRP eosRuscRu250scan
RUTYPE NORMAL RUN
#refdata /ortho_train/Ru/eos_Ru/sc_Ru/2.50_scan
REMARK Created by geo_energy_extract.py EO = -8.13626756 eV; -
189.2155246511628 kcal/mol
CRYSTX     2.50000    2.50000    2.50000   90.00000   90.00000   90.00000
HETATM     1 Ru                  0.00000   0.00000   0.00000   Ru1  1 1
0.00000
UNIT ENERGY   kcal
ENERGY        -189.2155246511628
END

XTLGRF 200
DESCRP eosRuscRu255scan
RUTYPE NORMAL RUN
#refdata /ortho_train/Ru/eos_Ru/sc_Ru/2.55_scan
REMARK Created by geo_energy_extract.py EO = -8.10055578 eV; -
188.3850181395349 kcal/mol
CRYSTX     2.55000    2.55000    2.55000   90.00000   90.00000   90.00000
HETATM     1 Ru                  0.00000   0.00000   0.00000   Ru1  1 1
0.00000
UNIT ENERGY   kcal
ENERGY        -188.3850181395349
END

XTLGRF 200
DESCRP eosRuscRu260scan
RUTYPE NORMAL RUN
#refdata /ortho_train/Ru/eos_Ru/sc_Ru/2.60_scan
REMARK Created by geo_energy_extract.py EO = -8.00261587 eV; -
186.1073458139535 kcal/mol
CRYSTX     2.60000    2.60000    2.60000   90.00000   90.00000   90.00000
HETATM     1 Ru                  0.00000   0.00000   0.00000   Ru1  1 1
0.00000
UNIT ENERGY   kcal
ENERGY        -186.1073458139535
END

XTLGRF 200
DESCRP eosRuscRu265scan
RUTYPE NORMAL RUN
```

```
#refdata /ortho_train/Ru/eos_Ru/sc_Ru/2.65_scan
REMARK Created by geo_energy_extract.py EO = -7.85570163 eV; -
182.6907355813954 kcal/mol
CRYSTX     2.65000    2.65000    2.65000   90.00000   90.00000   90.00000
HETATM     1 Ru                  0.00000   0.00000   0.00000   Ru1  1 1
0.00000
UNIT ENERGY   kcal
ENERGY        -182.6907355813954
END

XTLGRF 200
DESCRP eosRuscRu270scan
RUTYPE NORMAL RUN
#refdata /ortho_train/Ru/eos_Ru/sc_Ru/2.70_scan
REMARK Created by geo_energy_extract.py EO = -7.67100634 eV; -
178.3954962790698 kcal/mol
CRYSTX     2.70000    2.70000    2.70000   90.00000   90.00000   90.00000
HETATM     1 Ru                  0.00000   0.00000   0.00000   Ru1  1 1
0.00000
UNIT ENERGY   kcal
ENERGY        -178.3954962790698
END

XTLGRF 200
DESCRP trainingRuvac01Ru
RUTYPE NORMAL RUN
#refdata /rf3/training/Ru_vac/01Ru
REMARK Created by geo_energy_extract.py EO = -1.15357673 eV; -
26.827365813953488 kcal/mol
CRYSTX    10.00000   10.00000   10.00000   90.00000   90.00000   90.00000
HETATM     1 Ru                  5.00049   5.00056   5.00027   Ru1  1 1
0.00000
UNIT ENERGY   kcal
ENERGY        -26.827365813953488
END

XTLGRF 200
DESCRP iningdefectsRu1vac54
RUTYPE NORMAL RUN
#refdata /rf3/training/defects_Ru/1_vac54
REMARK Created by geo_energy_extract.py EO = -487.81031694 eV; -
11344.425975348839 kcal/mol
CRYSTX     8.11167    8.11167   12.84504   90.00000   90.00000  120.00000
HETATM     1 Ru                  0.78054   1.35194   5.35024   Ru1  1 1
0.00000
HETATM     2 Ru                  0.78054   1.35194   9.63558   Ru1  1 1
0.00000
HETATM     3 Ru                  0.78193   4.06789   1.07045   Ru1  1 1
0.00000
HETATM     4 Ru                  0.78475   4.05282   5.34372   Ru1  1 1
0.00000
HETATM     5 Ru                  0.78475   4.05282   9.64215   Ru1  1 1
0.00000
HETATM     6 Ru                  0.78193   6.74768   1.07044   Ru1  1 1
0.00000
HETATM     7 Ru                  0.78475   6.76274   5.34372   Ru1  1 1
0.00000
```

HETATM 8 Ru 0.78475 6.76274 9.64214 Ru1 1 1
0.00000
HETATM 9 Ru 3.13191 -0.00723 1.07044 Ru1 1 1
0.00000
HETATM 10 Ru 3.11746 -0.00214 5.34372 Ru1 1 1
0.00000
HETATM 11 Ru 3.11746 -0.00214 9.64215 Ru1 1 1
0.00000
HETATM 12 Ru 3.13193 2.71112 1.07044 Ru1 1 1
0.00000
HETATM 13 Ru 3.11747 2.70602 5.34372 Ru1 1 1
0.00000
HETATM 14 Ru 3.11747 2.70602 9.64215 Ru1 1 1
0.00000
HETATM 15 Ru 3.12218 5.40777 1.07045 Ru1 1 1
0.00000
HETATM 16 Ru 3.12218 5.40778 5.34286 Ru1 1 1
0.00000
HETATM 17 Ru 3.12218 5.40778 9.64300 Ru1 1 1
0.00000
HETATM 18 Ru 5.45270 -1.34713 1.07044 Ru1 1 1
0.00000
HETATM 19 Ru 5.46434 -1.35709 5.34372 Ru1 1 1
0.00000
HETATM 20 Ru 5.46433 -1.35710 9.64215 Ru1 1 1
0.00000
HETATM 21 Ru 5.46383 1.35195 1.07044 Ru1 1 1
0.00000
HETATM 22 Ru 5.46383 1.35195 5.34250 Ru1 1 1
0.00000
HETATM 23 Ru 5.46383 1.35195 9.64336 Ru1 1 1
0.00000
HETATM 24 Ru 5.45270 4.05101 1.07045 Ru1 1 1
0.00000
HETATM 25 Ru 5.46433 4.06098 5.34372 Ru1 1 1
0.00000
HETATM 26 Ru 5.46433 4.06098 9.64214 Ru1 1 1
0.00000
HETATM 27 Ru 1.54634 0.02554 3.20248 Ru1 1 1
0.00000
HETATM 28 Ru 1.56254 -0.00252 7.49292 Ru1 1 1
0.00000
HETATM 29 Ru 1.54634 0.02553 11.78341 Ru1 1 1
0.00000
HETATM 30 Ru 1.54634 2.67834 3.20248 Ru1 1 1
0.00000
HETATM 31 Ru 1.56254 2.70641 7.49292 Ru1 1 1
0.00000
HETATM 32 Ru 1.54634 2.67835 11.78341 Ru1 1 1
0.00000
HETATM 33 Ru 1.56223 5.40779 3.21357 Ru1 1 1
0.00000
HETATM 34 Ru 1.55890 5.40779 7.49293 Ru1 1 1
0.00000
HETATM 35 Ru 1.56223 5.40779 11.77234 Ru1 1 1
0.00000
HETATM 36 Ru 3.90216 -1.35294 3.21357 Ru1 1 1
0.00000

```
HETATM    37 Ru                    3.90383  -1.35005   7.49293   Ru1  1 1
0.00000
HETATM    38 Ru                    3.90216  -1.35294  11.77234   Ru1  1 1
0.00000
HETATM    39 Ru                    3.89781   1.35194   3.20619   Ru1  1 1
0.00000
HETATM    40 Ru                    3.90253   1.35194   7.49291   Ru1  1 1
0.00000
HETATM    41 Ru                    3.89781   1.35194  11.77970   Ru1  1 1
0.00000
HETATM    42 Ru                    3.90217   4.05683   3.21357   Ru1  1 1
0.00000
HETATM    43 Ru                    3.90383   4.05394   7.49293   Ru1  1 1
0.00000
HETATM    44 Ru                    3.90217   4.05683  11.77234   Ru1  1 1
0.00000
HETATM    45 Ru                    6.27386  -2.70389   3.20248   Ru1  1 1
0.00000
HETATM    46 Ru                    6.24146  -2.70389   7.49292   Ru1  1 1
0.00000
HETATM    47 Ru                    6.27385  -2.70389  11.78341   Ru1  1 1
0.00000
HETATM    48 Ru                    6.24682  -0.00426   3.20619   Ru1  1 1
0.00000
HETATM    49 Ru                    6.24446  -0.00017   7.49291   Ru1  1 1
0.00000
HETATM    50 Ru                    6.24682  -0.00426  11.77970   Ru1  1 1
0.00000
HETATM    51 Ru                    6.24684   2.70816   3.20619   Ru1  1 1
0.00000
HETATM    52 Ru                    6.24448   2.70407   7.49291   Ru1  1 1
0.00000
HETATM    53 Ru                    6.24683   2.70815  11.77971   Ru1  1 1
0.00000
UNIT ENERGY   kcal
ENERGY        -11344.425975348839
END

XTLGRF 200
DESCRP iningdefectsRu2vac54
RUTYPE NORMAL RUN
#refdata /rf3/training/defects_Ru/2_vac54
REMARK Created by geo_energy_extract.py EO = -475.95478087 eV; -
11068.715834186047 kcal/mol
CRYSTX     8.11167    8.11167   12.84504   90.00000   90.00000  120.00000
HETATM     1 Ru                    0.77240   1.35266   5.34124   Ru1  1 1
0.00000
HETATM     2 Ru                    0.78173   1.35129   9.63698   Ru1  1 1
0.00000
HETATM     3 Ru                    0.77937   4.07147   1.07687   Ru1  1 1
0.00000
HETATM     4 Ru                    0.78641   4.04915   5.34260   Ru1  1 1
0.00000
HETATM     5 Ru                    0.78209   4.05702   9.62782   Ru1  1 1
0.00000
HETATM     6 Ru                    0.77514   6.76375   1.07939   Ru1  1 1
0.00000
```

HETATM 7 Ru 0.78673 6.75705 5.34312 Ru1 1 1
0.00000
HETATM 8 Ru 0.78717 6.77120 9.63378 Ru1 1 1
0.00000
HETATM 9 Ru 3.12688 -0.00670 1.08128 Ru1 1 1
0.00000
HETATM 10 Ru 3.12103 0.00070 9.63059 Ru1 1 1
0.00000
HETATM 11 Ru 3.12964 2.70956 1.07939 Ru1 1 1
0.00000
HETATM 12 Ru 3.12336 2.71319 5.34313 Ru1 1 1
0.00000
HETATM 13 Ru 3.12211 2.70378 9.63378 Ru1 1 1
0.00000
HETATM 14 Ru 3.12518 5.41427 1.08166 Ru1 1 1
0.00000
HETATM 15 Ru 3.12526 5.38864 5.34617 Ru1 1 1
0.00000
HETATM 16 Ru 3.12455 5.41336 9.63071 Ru1 1 1
0.00000
HETATM 17 Ru 5.44363 -1.34469 1.08688 Ru1 1 1
0.00000
HETATM 18 Ru 5.48020 -1.36580 5.33565 Ru1 1 1
0.00000
HETATM 19 Ru 5.46068 -1.35741 9.63378 Ru1 1 1
0.00000
HETATM 20 Ru 5.45510 1.35369 1.07992 Ru1 1 1
0.00000
HETATM 21 Ru 5.47795 1.35875 5.34564 Ru1 1 1
0.00000
HETATM 22 Ru 5.46408 1.35389 9.63975 Ru1 1 1
0.00000
HETATM 23 Ru 5.44571 4.04893 1.07635 Ru1 1 1
0.00000
HETATM 24 Ru 5.46795 4.06168 5.34085 Ru1 1 1
0.00000
HETATM 25 Ru 5.46747 4.06068 9.63685 Ru1 1 1
0.00000
HETATM 26 Ru 1.55522 -0.01015 3.21125 Ru1 1 1
0.00000
HETATM 27 Ru 1.59399 -0.00266 7.48127 Ru1 1 1
0.00000
HETATM 28 Ru 1.54233 0.02716 11.78630 Ru1 1 1
0.00000
HETATM 29 Ru 1.55143 2.67110 3.19577 Ru1 1 1
0.00000
HETATM 30 Ru 1.56576 2.69924 7.48854 Ru1 1 1
0.00000
HETATM 31 Ru 1.55526 2.68339 11.77033 Ru1 1 1
0.00000
HETATM 32 Ru 1.56337 5.40609 3.21607 Ru1 1 1
0.00000
HETATM 33 Ru 1.56053 5.41100 7.48861 Ru1 1 1
0.00000
HETATM 34 Ru 1.56315 5.40922 11.77379 Ru1 1 1
0.00000
HETATM 35 Ru 3.87402 -1.31864 3.23609 Ru1 1 1
0.00000

```
HETATM    36 Ru                  3.89284  -1.32898   7.48585   Ru1  1 1
0.00000
HETATM    37 Ru                  3.90135  -1.35212  11.77754   Ru1  1 1
0.00000
HETATM    38 Ru                  3.87915   1.32719   3.22674   Ru1  1 1
0.00000
HETATM    39 Ru                  3.88789   1.33665   7.49725   Ru1  1 1
0.00000
HETATM    40 Ru                  3.89637   1.35367  11.77902   Ru1  1 1
0.00000
HETATM    41 Ru                  3.90402   4.05806   3.21126   Ru1  1 1
0.00000
HETATM    42 Ru                  3.90895   4.05322   7.49600   Ru1  1 1
0.00000
HETATM    43 Ru                  3.89736   4.05991  11.77155   Ru1  1 1
0.00000
HETATM    44 Ru                  6.28757  -2.71210   3.18643   Ru1  1 1
0.00000
HETATM    45 Ru                  6.24491  -2.70518   7.49002   Ru1  1 1
0.00000
HETATM    46 Ru                  6.26920  -2.70097  11.78171   Ru1  1 1
0.00000
HETATM    47 Ru                  6.24386  -0.00088   3.21126   Ru1  1 1
0.00000
HETATM    48 Ru                  6.23917  -0.00046   7.48683   Ru1  1 1
0.00000
HETATM    49 Ru                  6.24656  -0.00472  11.78073   Ru1  1 1
0.00000
HETATM    50 Ru                  6.24176   2.70502   3.20644   Ru1  1 1
0.00000
HETATM    51 Ru                  6.24458   2.70640   7.49377   Ru1  1 1
0.00000
HETATM    52 Ru                  6.24743   2.70503  11.77895   Ru1  1 1
0.00000
UNIT ENERGY   kcal
ENERGY        -11068.715834186047
END

XTLGRF 200
DESCRP iningdefectsRu3vac54
RUTYPE NORMAL RUN
#refdata /rf3/training/defects_Ru/3_vac54
REMARK Created by geo_energy_extract.py EO = -464.12084770 eV; -
10793.508086046513 kcal/mol
CRYSTX     8.11167    8.11167   12.84504   90.00000   90.00000  120.00000
HETATM     1 Ru                  0.76818   1.35406   5.35117   Ru1  1 1
0.00000
HETATM     2 Ru                  0.78856   1.34229   9.63472   Ru1  1 1
0.00000
HETATM     3 Ru                  0.78549   4.06799   1.07021   Ru1  1 1
0.00000
HETATM     4 Ru                  0.77891   4.05298   5.35211   Ru1  1 1
0.00000
HETATM     5 Ru                  0.78859   4.06619   9.63397   Ru1  1 1
0.00000
HETATM     6 Ru                  0.78046   6.75966   1.07480   Ru1  1 1
0.00000
```

HETATM 7 Ru 0.78052 6.75962 5.34773 Ru1 1 1
0.00000
HETATM 8 Ru 0.78952 6.77529 9.63377 Ru1 1 1
0.00000
HETATM 9 Ru 3.13022 -0.00966 1.07136 Ru1 1 1
0.00000
HETATM 10 Ru 3.10978 0.00212 9.63281 Ru1 1 1
0.00000
HETATM 11 Ru 3.13024 2.71425 1.07063 Ru1 1 1
0.00000
HETATM 12 Ru 3.12713 2.71603 5.35191 Ru1 1 1
0.00000
HETATM 13 Ru 3.12053 2.70104 9.63377 Ru1 1 1
0.00000
HETATM 14 Ru 3.13116 5.42333 1.07042 Ru1 1 1
0.00000
HETATM 15 Ru 3.12210 5.40769 5.35649 Ru1 1 1
0.00000
HETATM 16 Ru 3.12215 5.40766 9.62939 Ru1 1 1
0.00000
HETATM 17 Ru 5.45143 -1.34982 1.06947 Ru1 1 1
0.00000
HETATM 18 Ru 5.47185 -1.36162 5.35307 Ru1 1 1
0.00000
HETATM 19 Ru 5.46217 1.34910 1.07042 Ru1 1 1
0.00000
HETATM 20 Ru 5.47187 1.36231 5.35232 Ru1 1 1
0.00000
HETATM 21 Ru 5.46877 1.36409 9.63356 Ru1 1 1
0.00000
HETATM 22 Ru 5.46380 4.05572 1.06604 Ru1 1 1
0.00000
HETATM 23 Ru 5.47281 4.07139 5.35211 Ru1 1 1
0.00000
HETATM 24 Ru 5.46374 4.05577 9.63815 Ru1 1 1
0.00000
HETATM 25 Ru 1.55650 -0.00794 3.21127 Ru1 1 1
0.00000
HETATM 26 Ru 1.60246 -0.00771 7.47390 Ru1 1 1
0.00000
HETATM 27 Ru 1.53373 0.03196 11.79362 Ru1 1 1
0.00000
HETATM 28 Ru 1.55116 2.67943 3.21620 Ru1 1 1
0.00000
HETATM 29 Ru 1.56080 2.70339 7.49294 Ru1 1 1
0.00000
HETATM 30 Ru 1.54487 2.68305 11.76968 Ru1 1 1
0.00000
HETATM 31 Ru 1.56642 5.40388 3.21674 Ru1 1 1
0.00000
HETATM 32 Ru 1.55504 5.41045 7.48747 Ru1 1 1
0.00000
HETATM 33 Ru 1.56295 5.41103 11.77461 Ru1 1 1
0.00000
HETATM 34 Ru 3.87537 -1.31999 3.23030 Ru1 1 1
0.00000
HETATM 35 Ru 3.89815 -1.35989 7.49294 Ru1 1 1
0.00000

```
HETATM    36 Ru                    3.94409  -1.35967  11.75558   Ru1  1 1
0.00000
HETATM    37 Ru                    3.88650   1.33111   3.20635   Ru1  1 1
0.00000
HETATM    38 Ru                    3.89279   1.32749   7.49785   Ru1  1 1
0.00000
HETATM    39 Ru                    3.90244   1.35145  11.77461   Ru1  1 1
0.00000
HETATM    40 Ru                    3.90462   4.05907   3.21128   Ru1  1 1
0.00000
HETATM    41 Ru                    3.90807   4.05193   7.49841   Ru1  1 1
0.00000
HETATM    42 Ru                    3.89670   4.05850  11.76914   Ru1  1 1
0.00000
HETATM    43 Ru                    6.28572  -2.71161   3.19226   Ru1  1 1
0.00000
HETATM    44 Ru                    6.21701  -2.67192   7.51197   Ru1  1 1
0.00000
HETATM    45 Ru                    6.23979  -2.71183  11.77461   Ru1  1 1
0.00000
HETATM    46 Ru                    6.24408  -0.00050   3.21127   Ru1  1 1
0.00000
HETATM    47 Ru                    6.22815  -0.02083   7.48803   Ru1  1 1
0.00000
HETATM    48 Ru                    6.23443  -0.02447  11.77954   Ru1  1 1
0.00000
HETATM    49 Ru                    6.23833   2.70656   3.20581   Ru1  1 1
0.00000
HETATM    50 Ru                    6.24625   2.70713   7.49294   Ru1  1 1
0.00000
HETATM    51 Ru                    6.24970   2.69999  11.78007   Ru1  1 1
0.00000
UNIT ENERGY   kcal
ENERGY        -10793.508086046513
END

XTLGRF 200
DESCRP iningdefectsRu4vac54
RUTYPE NORMAL RUN
#refdata /rf3/training/defects_Ru/4_vac54
REMARK Created by geo_energy_extract.py E0 = -452.60685243 eV; -
10525.740754186047 kcal/mol
CRYSTX     8.11167    8.11167   12.84504   90.00000   90.00000  120.00000
HETATM     1 Ru                    0.76146   1.35062   5.36012   Ru1  1 1
0.00000
HETATM     2 Ru                    0.80219   1.28956   9.69601   Ru1  1 1
0.00000
HETATM     3 Ru                    0.78653   4.08439   1.08089   Ru1  1 1
0.00000
HETATM     4 Ru                    0.78338   4.05477   5.35134   Ru1  1 1
0.00000
HETATM     5 Ru                    0.77713   4.05262   9.63227   Ru1  1 1
0.00000
HETATM     6 Ru                    0.79779   6.79009   1.07349   Ru1  1 1
0.00000
HETATM     7 Ru                    0.78225   6.76262   5.35096   Ru1  1 1
0.00000
```

```
HETATM     8 Ru                      0.78999   6.77076   9.62376   Ru1  1 1
0.00000
HETATM     9 Ru                      3.09972  -0.00335   1.06318   Ru1  1 1
0.00000
HETATM    10 Ru                      3.09852  -0.01700   9.65641   Ru1  1 1
0.00000
HETATM    11 Ru                      3.12901   2.72721   1.06983   Ru1  1 1
0.00000
HETATM    12 Ru                      3.12399   2.71307   5.36449   Ru1  1 1
0.00000
HETATM    13 Ru                      3.12166   2.67310   9.63844   Ru1  1 1
0.00000
HETATM    14 Ru                      3.13712   5.43474   1.05853   Ru1  1 1
0.00000
HETATM    15 Ru                      3.11797   5.40529   5.35533   Ru1  1 1
0.00000
HETATM    16 Ru                      3.11535   5.38932   9.61960   Ru1  1 1
0.00000
HETATM    17 Ru                      5.44508  -1.33030   1.07320   Ru1  1 1
0.00000
HETATM    18 Ru                      5.46656  -1.36422   5.35721   Ru1  1 1
0.00000
HETATM    19 Ru                      5.46538   1.35909   1.07942   Ru1  1 1
0.00000
HETATM    20 Ru                      5.46947   1.36693   5.34487   Ru1  1 1
0.00000
HETATM    21 Ru                      5.47352   1.34762   9.62335   Ru1  1 1
0.00000
HETATM    22 Ru                      5.47044   4.07556   1.05294   Ru1  1 1
0.00000
HETATM    23 Ru                      5.47108   4.07218   5.35973   Ru1  1 1
0.00000
HETATM    24 Ru                      5.46154   4.02668   9.63226   Ru1  1 1
0.00000
HETATM    25 Ru                      1.55042   0.00846   3.19573   Ru1  1 1
0.00000
HETATM    26 Ru                      1.59737  -0.01471   7.49773   Ru1  1 1
0.00000
HETATM    27 Ru                      1.54628   2.68704   3.22045   Ru1  1 1
0.00000
HETATM    28 Ru                      1.55054   2.68421   7.50860   Ru1  1 1
0.00000
HETATM    29 Ru                      1.55040   2.70990  11.77081   Ru1  1 1
0.00000
HETATM    30 Ru                      1.56439   5.42274   3.21164   Ru1  1 1
0.00000
HETATM    31 Ru                      1.54983   5.40589   7.49062   Ru1  1 1
0.00000
HETATM    32 Ru                      1.56641   5.39176  11.76965   Ru1  1 1
0.00000
HETATM    33 Ru                      3.87118  -1.30224   3.21175   Ru1  1 1
0.00000
HETATM    34 Ru                      3.90019  -1.35979   7.50150   Ru1  1 1
0.00000
HETATM    35 Ru                      3.96929  -1.36782  11.70788   Ru1  1 1
0.00000
HETATM    36 Ru                      3.88317   1.33896   3.19576   Ru1  1 1
0.00000
```

```
HETATM    37 Ru                       3.89210   1.30030   7.49592   Ru1  1 1
0.00000
HETATM    38 Ru                       3.93093   1.35401  11.78043   Ru1  1 1
0.00000
HETATM    39 Ru                       3.90418   4.06613   3.20958   Ru1  1 1
0.00000
HETATM    40 Ru                       3.90416   4.04050   7.50659   Ru1  1 1
0.00000
HETATM    41 Ru                       3.89969   4.05662  11.76627   Ru1  1 1
0.00000
HETATM    42 Ru                       6.27987  -2.69517   3.19390   Ru1  1 1
0.00000
HETATM    43 Ru                       6.21954  -2.67112   7.53444   Ru1  1 1
0.00000
HETATM    44 Ru                       6.20849  -2.68145  11.73723   Ru1  1 1
0.00000
HETATM    45 Ru                       6.24208   0.00813   3.21371   Ru1  1 1
0.00000
HETATM    46 Ru                       6.23510  -0.02489   7.48445   Ru1  1 1
0.00000
HETATM    47 Ru                       6.24426  -0.00055  11.80336   Ru1  1 1
0.00000
HETATM    48 Ru                       6.24156   2.72479   3.19562   Ru1  1 1
0.00000
HETATM    49 Ru                       6.24695   2.70206   7.48492   Ru1  1 1
0.00000
HETATM    50 Ru                       6.24946   2.69246  11.77903   Ru1  1 1
0.00000
UNIT ENERGY   kcal
ENERGY        -10525.740754186047
END

XTLGRF 200
DESCRP iningdefectsRu0vac32
RUTYPE NORMAL RUN
#refdata /rf3/training/defects_Ru/0_vac32
REMARK Created by geo_energy_extract.py EO = -295.94644609 eV; -
6882.475490465116 kcal/mol
CRYSTX     5.40782    9.36655    8.56336   90.00000   90.00000   90.00000
HETATM     1 Ru                       2.70391   1.52984   1.07041   Ru1  1 1
0.00000
HETATM     2 Ru                       2.70391   1.52984   5.35208   Ru1  1 1
0.00000
HETATM     3 Ru                       2.70391   6.21312   1.07041   Ru1  1 1
0.00000
HETATM     4 Ru                       2.70391   6.21312   5.35208   Ru1  1 1
0.00000
HETATM     5 Ru                       0.00000   1.52984   1.07040   Ru1  1 1
0.00000
HETATM     6 Ru                       0.00000   1.52984   5.35208   Ru1  1 1
0.00000
HETATM     7 Ru                       0.00000   6.21312   1.07041   Ru1  1 1
0.00000
HETATM     8 Ru                       0.00000   6.21312   5.35208   Ru1  1 1
0.00000
HETATM     9 Ru                       1.35189   3.87149   1.07040   Ru1  1 1
0.00000
```

```
HETATM    10 Ru                    1.35189   3.87149   5.35209   Ru1  1 1
0.00000
HETATM    11 Ru                    1.35189   8.55477   1.07041   Ru1  1 1
0.00000
HETATM    12 Ru                    1.35189   8.55476   5.35209   Ru1  1 1
0.00000
HETATM    13 Ru                    4.05580   3.87149   1.07041   Ru1  1 1
0.00000
HETATM    14 Ru                    4.05580   3.87149   5.35209   Ru1  1 1
0.00000
HETATM    15 Ru                    4.05580   8.55476   1.07041   Ru1  1 1
0.00000
HETATM    16 Ru                    4.05580   8.55477   5.35208   Ru1  1 1
0.00000
HETATM    17 Ru                    1.35198   0.75075   3.21127   Ru1  1 1
0.00000
HETATM    18 Ru                    1.35198   0.75075   7.49295   Ru1  1 1
0.00000
HETATM    19 Ru                    1.35198   5.43402   3.21127   Ru1  1 1
0.00000
HETATM    20 Ru                    1.35198   5.43402   7.49295   Ru1  1 1
0.00000
HETATM    21 Ru                    4.05589   0.75075   3.21127   Ru1  1 1
0.00000
HETATM    22 Ru                    4.05589   0.75075   7.49295   Ru1  1 1
0.00000
HETATM    23 Ru                    4.05589   5.43402   3.21127   Ru1  1 1
0.00000
HETATM    24 Ru                    4.05589   5.43402   7.49295   Ru1  1 1
0.00000
HETATM    25 Ru                    2.70388   3.09235   3.21128   Ru1  1 1
0.00000
HETATM    26 Ru                    2.70388   3.09235   7.49296   Ru1  1 1
0.00000
HETATM    27 Ru                    2.70388   7.77563   3.21128   Ru1  1 1
0.00000
HETATM    28 Ru                    2.70388   7.77563   7.49296   Ru1  1 1
0.00000
HETATM    29 Ru                   -0.00003   3.09235   3.21128   Ru1  1 1
0.00000
HETATM    30 Ru                   -0.00003   3.09235   7.49296   Ru1  1 1
0.00000
HETATM    31 Ru                   -0.00003   7.77563   3.21128   Ru1  1 1
0.00000
HETATM    32 Ru                   -0.00003   7.77563   7.49296   Ru1  1 1
0.00000
UNIT ENERGY   kcal
ENERGY        -6882.475490465116
END

XTLGRF 200
DESCRP iningdefectsRu1vac32
RUTYPE NORMAL RUN
#refdata /rf3/training/defects_Ru/1_vac32
REMARK Created by geo_energy_extract.py EO = -283.93235247 eV; -
6603.077964418605 kcal/mol
CRYSTX     5.40782    9.36655    8.56336   90.00000   90.00000   90.00000
```

```
HETATM     1 Ru                     2.70389   1.53539   1.06687   Ru1  1 1
0.00000
HETATM     2 Ru                     2.70389   1.53538   5.35564   Ru1  1 1
0.00000
HETATM     3 Ru                     2.70389   6.22031   1.06671   Ru1  1 1
0.00000
HETATM     4 Ru                     2.70389   6.22031   5.35579   Ru1  1 1
0.00000
HETATM     5 Ru                    -0.00002   1.51916   1.07427   Ru1  1 1
0.00000
HETATM     6 Ru                    -0.00002   1.51916   5.34824   Ru1  1 1
0.00000
HETATM     7 Ru                    -0.00002   6.24509   1.06663   Ru1  1 1
0.00000
HETATM     8 Ru                    -0.00002   6.24507   5.35583   Ru1  1 1
0.00000
HETATM     9 Ru                     1.35489   3.86724   1.07330   Ru1  1 1
0.00000
HETATM    10 Ru                     1.35489   3.86725   5.34920   Ru1  1 1
0.00000
HETATM    11 Ru                     1.30716   8.53322   1.04386   Ru1  1 1
0.00000
HETATM    12 Ru                     1.30718   8.53323   5.37864   Ru1  1 1
0.00000
HETATM    13 Ru                     4.05290   3.86724   1.07331   Ru1  1 1
0.00000
HETATM    14 Ru                     4.05290   3.86724   5.34920   Ru1  1 1
0.00000
HETATM    15 Ru                     4.10062   8.53322   1.04385   Ru1  1 1
0.00000
HETATM    16 Ru                     4.10060   8.53323   5.37864   Ru1  1 1
0.00000
HETATM    17 Ru                     1.35781   0.73002   3.21127   Ru1  1 1
0.00000
HETATM    18 Ru                     1.33880   0.74743   7.49295   Ru1  1 1
0.00000
HETATM    19 Ru                     1.34285   5.44309   3.21126   Ru1  1 1
0.00000
HETATM    20 Ru                     1.34164   5.45319   7.49294   Ru1  1 1
0.00000
HETATM    21 Ru                     4.04997   0.73003   3.21127   Ru1  1 1
0.00000
HETATM    22 Ru                     4.06899   0.74743   7.49295   Ru1  1 1
0.00000
HETATM    23 Ru                     4.06494   5.44309   3.21126   Ru1  1 1
0.00000
HETATM    24 Ru                     4.06615   5.45319   7.49294   Ru1  1 1
0.00000
HETATM    25 Ru                     2.70389   3.09217   3.21128   Ru1  1 1
0.00000
HETATM    26 Ru                     2.70390   3.10089   7.49296   Ru1  1 1
0.00000
HETATM    27 Ru                     2.70389   7.78787   3.21129   Ru1  1 1
0.00000
HETATM    28 Ru                     2.70389   7.79294   7.49297   Ru1  1 1
0.00000
HETATM    29 Ru                    -0.00002   3.08746   3.21128   Ru1  1 1
0.00000
```

```
HETATM    30 Ru                 -0.00001   3.09014   7.49297   Ru1  1 1
0.00000
HETATM    31 Ru                 -0.00002   7.77278   3.21127   Ru1  1 1
0.00000
UNIT ENERGY   kcal
ENERGY        -6603.077964418605
END

XTLGRF 200
DESCRP iningdefectsRu2vac32
RUTYPE NORMAL RUN
#refdata /rf3/training/defects_Ru/2_vac32
REMARK Created by geo_energy_extract.py EO = -272.49265746 eV; -
6337.038545581395 kcal/mol
CRYSTX     5.40782    9.36655    8.56336   90.00000   90.00000   90.00000
HETATM     1 Ru                  2.70390   1.53727   1.07041   Ru1  1 1
0.00000
HETATM     2 Ru                  2.70390   1.53727   5.35210   Ru1  1 1
0.00000
HETATM     3 Ru                  2.70390   6.21597   1.07041   Ru1  1 1
0.00000
HETATM     4 Ru                  2.70390   6.21597   5.35210   Ru1  1 1
0.00000
HETATM     5 Ru                 -0.00001   1.51609   1.07041   Ru1  1 1
0.00000
HETATM     6 Ru                 -0.00001   1.51609   5.35209   Ru1  1 1
0.00000
HETATM     7 Ru                 -0.00002   6.32508   1.07039   Ru1  1 1
0.00000
HETATM     8 Ru                 -0.00002   6.32508   5.35207   Ru1  1 1
0.00000
HETATM     9 Ru                  1.34729   3.86597   1.07042   Ru1  1 1
0.00000
HETATM    10 Ru                  1.34729   3.86597   5.35210   Ru1  1 1
0.00000
HETATM    11 Ru                  1.23903   8.49974   1.07039   Ru1  1 1
0.00000
HETATM    12 Ru                  1.23903   8.49974   5.35207   Ru1  1 1
0.00000
HETATM    13 Ru                  4.06049   3.86598   1.07041   Ru1  1 1
0.00000
HETATM    14 Ru                  4.06049   3.86598   5.35210   Ru1  1 1
0.00000
HETATM    15 Ru                  4.16873   8.49974   1.07039   Ru1  1 1
0.00000
HETATM    16 Ru                  4.16873   8.49974   5.35207   Ru1  1 1
0.00000
HETATM    17 Ru                  1.34146   0.73035   3.21127   Ru1  1 1
0.00000
HETATM    18 Ru                  1.34146   0.73035   7.49295   Ru1  1 1
0.00000
HETATM    19 Ru                  1.34080   5.46075   3.21127   Ru1  1 1
0.00000
HETATM    20 Ru                  1.34080   5.46075   7.49295   Ru1  1 1
0.00000
HETATM    21 Ru                  4.06634   0.73035   3.21127   Ru1  1 1
0.00000
```

```
HETATM    22 Ru                    4.06634   0.73035   7.49295   Ru1  1 1
0.00000
HETATM    23 Ru                    4.06699   5.46075   3.21127   Ru1  1 1
0.00000
HETATM    24 Ru                    4.06699   5.46075   7.49295   Ru1  1 1
0.00000
HETATM    25 Ru                    2.70389   3.09734   3.21128   Ru1  1 1
0.00000
HETATM    26 Ru                    2.70389   3.09734   7.49296   Ru1  1 1
0.00000
HETATM    27 Ru                    2.70387   7.78579   3.21130   Ru1  1 1
0.00000
HETATM    28 Ru                    2.70387   7.78579   7.49298   Ru1  1 1
0.00000
HETATM    29 Ru                   -0.00002   3.07837   3.21128   Ru1  1 1
0.00000
HETATM    30 Ru                   -0.00002   3.07837   7.49296   Ru1  1 1
0.00000
UNIT ENERGY   kcal
ENERGY        -6337.038545581395
END

XTLGRF 200
DESCRP iningdefectsRu3vac32
RUTYPE NORMAL RUN
#refdata /rf3/training/defects_Ru/3_vac32
REMARK Created by geo_energy_extract.py EO = -260.87361678 eV; -
6066.828297209303 kcal/mol
CRYSTX     5.40782    9.36655    8.56336   90.00000   90.00000   90.00000
HETATM     1 Ru                    2.70967   1.52176   1.07042   Ru1  1 1
0.00000
HETATM     2 Ru                    2.71367   1.52522   5.35209   Ru1  1 1
0.00000
HETATM     3 Ru                    2.71839   6.23202   1.07041   Ru1  1 1
0.00000
HETATM     4 Ru                    2.74049   6.21398   5.35210   Ru1  1 1
0.00000
HETATM     5 Ru                   -0.00157   1.50122   1.07042   Ru1  1 1
0.00000
HETATM     6 Ru                   -0.01686   1.50475   5.35210   Ru1  1 1
0.00000
HETATM     7 Ru                    0.02703   6.31722   1.07040   Ru1  1 1
0.00000
HETATM     8 Ru                   -0.00629   6.42335   5.35206   Ru1  1 1
0.00000
HETATM     9 Ru                    1.36010   3.86407   1.07042   Ru1  1 1
0.00000
HETATM    10 Ru                    1.34903   3.84657   5.35210   Ru1  1 1
0.00000
HETATM    11 Ru                    1.26869   8.48322   1.07039   Ru1  1 1
0.00000
HETATM    12 Ru                    1.16073   8.44295   5.35204   Ru1  1 1
0.00000
HETATM    13 Ru                    4.06435   3.86804   1.07042   Ru1  1 1
0.00000
HETATM    14 Ru                    4.06078   3.86300   5.35210   Ru1  1 1
0.00000
```

```
HETATM    15 Ru                   4.19068   8.49226   1.07041   Ru1  1 1
0.00000
HETATM    16 Ru                   1.32630   0.72793   3.22250   Ru1  1 1
0.00000
HETATM    17 Ru                   1.32631   0.72792   7.48173   Ru1  1 1
0.00000
HETATM    18 Ru                   1.34712   5.45235   3.22892   Ru1  1 1
0.00000
HETATM    19 Ru                   1.34712   5.45235   7.47530   Ru1  1 1
0.00000
HETATM    20 Ru                   4.05595   0.67860   3.19941   Ru1  1 1
0.00000
HETATM    21 Ru                   4.05595   0.67861   7.50482   Ru1  1 1
0.00000
HETATM    22 Ru                   4.07404   5.47163   3.21519   Ru1  1 1
0.00000
HETATM    23 Ru                   4.07404   5.47162   7.48902   Ru1  1 1
0.00000
HETATM    24 Ru                   2.70846   3.10161   3.20323   Ru1  1 1
0.00000
HETATM    25 Ru                   2.70846   3.10161   7.50099   Ru1  1 1
0.00000
HETATM    26 Ru                   2.73641   7.81977   3.23111   Ru1  1 1
0.00000
HETATM    27 Ru                   2.73640   7.81977   7.47314   Ru1  1 1
0.00000
HETATM    28 Ru                   0.00105   3.08987   3.20527   Ru1  1 1
0.00000
HETATM    29 Ru                   0.00105   3.08987   7.49896   Ru1  1 1
0.00000
UNIT ENERGY   kcal
ENERGY        -6066.828297209303
END

XTLGRF 200
DESCRP iningdefectsRu4vac32
RUTYPE NORMAL RUN
#refdata /rf3/training/defects_Ru/4_vac32
REMARK Created by geo_energy_extract.py EO = -249.13107640 eV; -
5793.745962790698 kcal/mol
CRYSTX     5.40782    9.36655    8.56336   90.00000   90.00000   90.00000
HETATM     1 Ru                   2.71289   1.52726   5.35210   Ru1  1 1
0.00000
HETATM     2 Ru                   2.72996   6.24595   1.07042   Ru1  1 1
0.00000
HETATM     3 Ru                   2.75032   6.19590   5.35210   Ru1  1 1
0.00000
HETATM     4 Ru                  -0.00267   1.53185   1.07043   Ru1  1 1
0.00000
HETATM     5 Ru                  -0.04303   1.42491   5.35210   Ru1  1 1
0.00000
HETATM     6 Ru                   0.04183   6.31228   1.07040   Ru1  1 1
0.00000
HETATM     7 Ru                   0.00715   6.41866   5.35205   Ru1  1 1
0.00000
HETATM     8 Ru                   1.37454   3.86325   1.07041   Ru1  1 1
0.00000
```

```
HETATM     9 Ru                 1.34883   3.82383   5.35209   Ru1  1 1
0.00000
HETATM    10 Ru                 1.29339   8.48782   1.07040   Ru1  1 1
0.00000
HETATM    11 Ru                 1.20324   8.42989   5.35205   Ru1  1 1
0.00000
HETATM    12 Ru                 4.06429   3.86843   1.07041   Ru1  1 1
0.00000
HETATM    13 Ru                 4.03878   3.83880   5.35209   Ru1  1 1
0.00000
HETATM    14 Ru                 4.17926   8.51518   1.07040   Ru1  1 1
0.00000
HETATM    15 Ru                 1.35509   0.72471   3.21195   Ru1  1 1
0.00000
HETATM    16 Ru                 1.35509   0.72471   7.49228   Ru1  1 1
0.00000
HETATM    17 Ru                 1.34728   5.46674   3.23459   Ru1  1 1
0.00000
HETATM    18 Ru                 1.34728   5.46674   7.46961   Ru1  1 1
0.00000
HETATM    19 Ru                 3.98998   0.79281   3.09837   Ru1  1 1
0.00000
HETATM    20 Ru                 3.98996   0.79284   7.60590   Ru1  1 1
0.00000
HETATM    21 Ru                 4.08027   5.46858   3.21540   Ru1  1 1
0.00000
HETATM    22 Ru                 4.08028   5.46859   7.48879   Ru1  1 1
0.00000
HETATM    23 Ru                 2.71919   3.05479   3.19100   Ru1  1 1
0.00000
HETATM    24 Ru                 2.71918   3.05480   7.51321   Ru1  1 1
0.00000
HETATM    25 Ru                 2.72646   7.80514   3.22627   Ru1  1 1
0.00000
HETATM    26 Ru                 2.72642   7.80514   7.47798   Ru1  1 1
0.00000
HETATM    27 Ru                -0.00081   3.07125   3.20790   Ru1  1 1
0.00000
HETATM    28 Ru                -0.00081   3.07125   7.49632   Ru1  1 1
0.00000
UNIT ENERGY   kcal
ENERGY       -5793.745962790698
END

XTLGRF 200
DESCRP ingaultsRuc111hcp001
RUTYPE NORMAL RUN
#refdata /rf3/training/stack_faults_Ru/fcc111hcp001
REMARK Created by geo_energy_extract.py EO = -146.63956168 eV; -
3410.2223646511634 kcal/mol
CRYSTX     2.70391    4.68327   17.12640   90.00000   90.00000   90.00000
HETATM     1 Ru                 2.02794   2.83389   1.04140   Ru1  1 1
0.00000
HETATM     2 Ru                 0.67605   2.04895   3.21112   Ru1  1 1
0.00000
HETATM     3 Ru                 2.02791   4.39059   3.21115   Ru1  1 1
0.00000
```

```
HETATM     4 Ru                     0.67594   3.60571   5.38081   Ru1  1 1
0.00000
HETATM     5 Ru                     2.02795   1.26400   5.38094   Ru1  1 1
0.00000
HETATM     6 Ru                     0.67596   0.47474   7.49300   Ru1  1 1
0.00000
HETATM     7 Ru                     2.02793   2.81599   7.49306   Ru1  1 1
0.00000
HETATM     8 Ru                     0.67605   3.60564   9.60465   Ru1  1 1
0.00000
HETATM     9 Ru                     2.02784   1.26402   9.60461   Ru1  1 1
0.00000
HETATM    10 Ru                     0.67598   2.04890  11.77449   Ru1  1 1
0.00000
HETATM    11 Ru                     2.02797   4.39064  11.77445   Ru1  1 1
0.00000
HETATM    12 Ru                     0.67599   0.49220  13.94398   Ru1  1 1
0.00000
HETATM    13 Ru                     2.02791   2.83378  13.94394   Ru1  1 1
0.00000
HETATM    14 Ru                     0.67598   3.62325  16.05616   Ru1  1 1
0.00000
HETATM    15 Ru                     2.02793   1.28164  16.05601   Ru1  1 1
0.00000
HETATM    16 Ru                     0.67594   0.49221   1.04145   Ru1  1 1
0.00000
UNIT ENERGY   kcal
ENERGY         -3410.2223646511634
END

XTLGRF 200
DESCRP trainingRuslab0001
RUTYPE NORMAL RUN
#refdata /rf3/training/Ru_slab/0001
REMARK Created by geo_energy_extract.py EO = -376.25821947 eV; -
8750.191150465116 kcal/mol
CRYSTX     8.11167    4.68327   28.85554   90.00000   90.00000   90.00000
HETATM     1 Ru                     8.11166   1.53106   1.12291   Ru1  1 1
0.00000
HETATM     2 Ru                     5.40778   1.53091   1.12267   Ru1  1 1
0.00000
HETATM     3 Ru                     2.70393   1.53091   1.12267   Ru1  1 1
0.00000
HETATM     4 Ru                     6.75986   3.87257   1.12287   Ru1  1 1
0.00000
HETATM     5 Ru                     4.05586   3.87230   1.12261   Ru1  1 1
0.00000
HETATM     6 Ru                     1.35185   3.87255   1.12288   Ru1  1 1
0.00000
HETATM     7 Ru                     1.35192   0.74952   3.18243   Ru1  1 1
0.00000
HETATM     8 Ru                     6.75978   0.74952   3.18243   Ru1  1 1
0.00000
HETATM     9 Ru                     4.05585   0.74939   3.18210   Ru1  1 1
0.00000
HETATM    10 Ru                     0.00002   3.09120   3.18252   Ru1  1 1
0.00000
```

```
HETATM    11 Ru                    5.40777   3.09107   3.18225   Ru1  1 1
0.00000
HETATM    12 Ru                    2.70394   3.09107   3.18226   Ru1  1 1
0.00000
HETATM    13 Ru                    0.00001   1.52967   5.32798   Ru1  1 1
0.00000
HETATM    14 Ru                    5.40769   1.52956   5.32781   Ru1  1 1
0.00000
HETATM    15 Ru                    2.70400   1.52956   5.32781   Ru1  1 1
0.00000
HETATM    16 Ru                    6.75973   3.87129   5.32795   Ru1  1 1
0.00000
HETATM    17 Ru                    4.05585   3.87120   5.32775   Ru1  1 1
0.00000
HETATM    18 Ru                    1.35195   3.87129   5.32797   Ru1  1 1
0.00000
HETATM    19 Ru                    1.35203   0.74752   7.48333   Ru1  1 1
0.00000
HETATM    20 Ru                    6.75964   0.74751   7.48332   Ru1  1 1
0.00000
HETATM    21 Ru                    4.05584   0.74733   7.48336   Ru1  1 1
0.00000
HETATM    22 Ru                    8.11165   3.08913   7.48328   Ru1  1 1
0.00000
HETATM    23 Ru                    5.40759   3.08902   7.48332   Ru1  1 1
0.00000
HETATM    24 Ru                    2.70407   3.08902   7.48332   Ru1  1 1
0.00000
HETATM    25 Ru                    0.00000   1.52691   9.63844   Ru1  1 1
0.00000
HETATM    26 Ru                    5.40756   1.52694   9.63881   Ru1  1 1
0.00000
HETATM    27 Ru                    2.70412   1.52694   9.63881   Ru1  1 1
0.00000
HETATM    28 Ru                    6.75961   3.86849   9.63854   Ru1  1 1
0.00000
HETATM    29 Ru                    4.05584   3.86858   9.63900   Ru1  1 1
0.00000
HETATM    30 Ru                    1.35206   3.86849   9.63853   Ru1  1 1
0.00000
HETATM    31 Ru                    1.35199   0.74462  11.78359   Ru1  1 1
0.00000
HETATM    32 Ru                    6.75968   0.74462  11.78359   Ru1  1 1
0.00000
HETATM    33 Ru                    4.05584   0.74458  11.78454   Ru1  1 1
0.00000
HETATM    34 Ru                    0.00000   3.08635  11.78345   Ru1  1 1
0.00000
HETATM    35 Ru                    5.40768   3.08624  11.78426   Ru1  1 1
0.00000
HETATM    36 Ru                    2.70400   3.08624  11.78426   Ru1  1 1
0.00000
HETATM    37 Ru                    8.11167   1.52544  13.84290   Ru1  1 1
0.00000
HETATM    38 Ru                    5.40809   1.52514  13.84382   Ru1  1 1
0.00000
HETATM    39 Ru                    2.70357   1.52515  13.84382   Ru1  1 1
0.00000
```

```
HETATM    40 Ru                    6.75987   3.86717  13.84327   Ru1  1 1
0.00000
HETATM    41 Ru                    4.05584   3.86702  13.84427   Ru1  1 1
0.00000
HETATM    42 Ru                    1.35180   3.86717  13.84327   Ru1  1 1
0.00000
UNIT ENERGY   kcal
ENERGY        -8750.191150465116
END

XTLGRF 200
DESCRP Ruads1Rufcc
RUTYPE NORMAL RUN
#refdata /rf3/training/Ru_ads/1Ru/fcc
REMARK Created by geo_energy_extract.py EO = -383.53060525 eV; -
8919.316401162792 kcal/mol
CRYSTX     8.11167    4.68327   28.85554   90.00000   90.00000   90.00000
HETATM     1 Ru                   -0.00014   1.53792   1.14611   Ru1  1 1
0.00000
HETATM     2 Ru                    5.40892   1.53203   1.14742   Ru1  1 1
0.00000
HETATM     3 Ru                    2.70247   1.53203   1.14742   Ru1  1 1
0.00000
HETATM     4 Ru                    6.75938   3.87342   1.14847   Ru1  1 1
0.00000
HETATM     5 Ru                    4.05569   3.87022   1.14970   Ru1  1 1
0.00000
HETATM     6 Ru                    1.35201   3.87342   1.14847   Ru1  1 1
0.00000
HETATM     7 Ru                    1.35152   0.75035   3.21202   Ru1  1 1
0.00000
HETATM     8 Ru                    6.75986   0.75035   3.21202   Ru1  1 1
0.00000
HETATM     9 Ru                    4.05569   0.75084   3.20373   Ru1  1 1
0.00000
HETATM    10 Ru                    8.11152   3.09207   3.21011   Ru1  1 1
0.00000
HETATM    11 Ru                    5.40756   3.09139   3.20327   Ru1  1 1
0.00000
HETATM    12 Ru                    2.70381   3.09139   3.20327   Ru1  1 1
0.00000
HETATM    13 Ru                    8.11151   1.53160   5.34977   Ru1  1 1
0.00000
HETATM    14 Ru                    5.40754   1.53009   5.35288   Ru1  1 1
0.00000
HETATM    15 Ru                    2.70381   1.53009   5.35288   Ru1  1 1
0.00000
HETATM    16 Ru                    6.75684   3.86991   5.35091   Ru1  1 1
0.00000
HETATM    17 Ru                    4.05567   3.87193   5.35342   Ru1  1 1
0.00000
HETATM    18 Ru                    1.35450   3.86991   5.35091   Ru1  1 1
0.00000
HETATM    19 Ru                    1.35584   0.74728   7.50704   Ru1  1 1
0.00000
HETATM    20 Ru                    6.75545   0.74728   7.50704   Ru1  1 1
0.00000
```

```
HETATM    21 Ru                 4.05564   0.75114   7.51360   Ru1  1 1
0.00000
HETATM    22 Ru                 8.11148   3.08716   7.50659   Ru1  1 1
0.00000
HETATM    23 Ru                 5.40330   3.09009   7.51177   Ru1  1 1
0.00000
HETATM    24 Ru                 2.70799   3.09009   7.51177   Ru1  1 1
0.00000
HETATM    25 Ru                 8.11145   1.53429   9.64101   Ru1  1 1
0.00000
HETATM    26 Ru                 5.41061   1.53528   9.66392   Ru1  1 1
0.00000
HETATM    27 Ru                 2.70062   1.53528   9.66392   Ru1  1 1
0.00000
HETATM    28 Ru                 6.75567   3.86147   9.65855   Ru1  1 1
0.00000
HETATM    29 Ru                 4.05562   3.87222   9.68390   Ru1  1 1
0.00000
HETATM    30 Ru                 1.35557   3.86147   9.65855   Ru1  1 1
0.00000
HETATM    31 Ru                 1.34188   0.74147  11.76727   Ru1  1 1
0.00000
HETATM    32 Ru                 6.76932   0.74147  11.76727   Ru1  1 1
0.00000
HETATM    33 Ru                 4.05560   0.77486  11.76780   Ru1  1 1
0.00000
HETATM    34 Ru                 8.11143   3.08390  11.81338   Ru1  1 1
0.00000
HETATM    35 Ru                 5.40312   3.09324  11.82378   Ru1  1 1
0.00000
HETATM    36 Ru                 2.70808   3.09324  11.82378   Ru1  1 1
0.00000
HETATM    37 Ru                 8.11142   1.49521  13.84880   Ru1  1 1
0.00000
HETATM    38 Ru                 5.49970   1.46448  13.95319   Ru1  1 1
0.00000
HETATM    39 Ru                 2.61146   1.46447  13.95318   Ru1  1 1
0.00000
HETATM    40 Ru                 6.79110   3.91006  13.87138   Ru1  1 1
0.00000
HETATM    41 Ru                 4.05558   4.02788  14.03058   Ru1  1 1
0.00000
HETATM    42 Ru                 1.32007   3.91005  13.87138   Ru1  1 1
0.00000
HETATM    43 Ru                 4.05556   2.32169  15.63068   Ru1  1 1
0.00000
UNIT ENERGY   kcal
ENERGY        -8919.316401162792
END

XTLGRF 200
DESCRP Ruads1Ruhcp
RUTYPE NORMAL RUN
#refdata /rf3/training/Ru_ads/1Ru/hcp
REMARK Created by geo_energy_extract.py EO = -384.05219506 eV; -
8931.446396744186 kcal/mol
CRYSTX     8.11167    4.68327   28.85554   90.00000   90.00000   90.00000
```

```
HETATM     1 Ru                   -0.00101   1.53469   1.14798   Ru1  1 1
0.00000
HETATM     2 Ru                    5.40842   1.53470   1.14798   Ru1  1 1
0.00000
HETATM     3 Ru                    2.70380   1.53212   1.14884   Ru1  1 1
0.00000
HETATM     4 Ru                    6.75963   3.87252   1.15037   Ru1  1 1
0.00000
HETATM     5 Ru                    4.05550   3.86813   1.15027   Ru1  1 1
0.00000
HETATM     6 Ru                    1.35192   3.86812   1.15028   Ru1  1 1
0.00000
HETATM     7 Ru                    1.35215   0.74959   3.21186   Ru1  1 1
0.00000
HETATM     8 Ru                    6.75959   0.74989   3.21495   Ru1  1 1
0.00000
HETATM     9 Ru                    4.05528   0.74957   3.21191   Ru1  1 1
0.00000
HETATM    10 Ru                    0.00015   3.09279   3.20953   Ru1  1 1
0.00000
HETATM    11 Ru                    5.40729   3.09281   3.20948   Ru1  1 1
0.00000
HETATM    12 Ru                    2.70377   3.09153   3.19945   Ru1  1 1
0.00000
HETATM    13 Ru                   -0.00018   1.53273   5.34896   Ru1  1 1
0.00000
HETATM    14 Ru                    5.40758   1.53273   5.34896   Ru1  1 1
0.00000
HETATM    15 Ru                    2.70375   1.53359   5.35823   Ru1  1 1
0.00000
HETATM    16 Ru                    6.75958   3.87512   5.35443   Ru1  1 1
0.00000
HETATM    17 Ru                    4.05269   3.87244   5.35913   Ru1  1 1
0.00000
HETATM    18 Ru                    1.35470   3.87244   5.35912   Ru1  1 1
0.00000
HETATM    19 Ru                    1.35371   0.75166   7.52018   Ru1  1 1
0.00000
HETATM    20 Ru                    6.75957   0.75164   7.49960   Ru1  1 1
0.00000
HETATM    21 Ru                    4.05363   0.75164   7.52020   Ru1  1 1
0.00000
HETATM    22 Ru                    0.00128   3.09646   7.51127   Ru1  1 1
0.00000
HETATM    23 Ru                    5.40609   3.09646   7.51128   Ru1  1 1
0.00000
HETATM    24 Ru                    2.70373   3.09686   7.49795   Ru1  1 1
0.00000
HETATM    25 Ru                    0.00351   1.53444   9.64570   Ru1  1 1
0.00000
HETATM    26 Ru                    5.40386   1.53441   9.64570   Ru1  1 1
0.00000
HETATM    27 Ru                    2.70371   1.52822   9.66814   Ru1  1 1
0.00000
HETATM    28 Ru                    6.75956   3.87383   9.66503   Ru1  1 1
0.00000
HETATM    29 Ru                    4.05669   3.87909   9.68001   Ru1  1 1
0.00000
```

```
HETATM    30 Ru                    1.35065   3.87911   9.68002   Ru1  1 1
0.00000
HETATM    31 Ru                    1.34716   0.75897  11.80298   Ru1  1 1
0.00000
HETATM    32 Ru                    6.75953   0.75532  11.80052   Ru1  1 1
0.00000
HETATM    33 Ru                    4.06016   0.75896  11.80290   Ru1  1 1
0.00000
HETATM    34 Ru                    0.00029   3.09172  11.79853   Ru1  1 1
0.00000
HETATM    35 Ru                    5.40707   3.09172  11.79852   Ru1  1 1
0.00000
HETATM    36 Ru                    2.70370   3.09073  11.81426   Ru1  1 1
0.00000
HETATM    37 Ru                    8.07771   1.51068  13.87220   Ru1  1 1
0.00000
HETATM    38 Ru                    5.44117   1.51068  13.87217   Ru1  1 1
0.00000
HETATM    39 Ru                    2.70369   1.39750  13.96383   Ru1  1 1
0.00000
HETATM    40 Ru                    6.75957   3.90280  13.87179   Ru1  1 1
0.00000
HETATM    41 Ru                    4.17285   3.94131  13.93826   Ru1  1 1
0.00000
HETATM    42 Ru                    1.23452   3.94131  13.93832   Ru1  1 1
0.00000
HETATM    43 Ru                    2.70385   3.11207  15.59180   Ru1  1 1
0.00000
UNIT ENERGY   kcal
ENERGY        -8931.446396744186
END

XTLGRF 200
DESCRP Ruads1Hfcc
RUTYPE NORMAL RUN
#refdata /rf3/training/Ru_ads/1H/fcc
REMARK Created by geo_energy_extract.py EO = -380.25369421 eV; -
8843.10916767442 kcal/mol
CRYSTX     8.11167    4.68327   28.85554   90.00000   90.00000   90.00000
HETATM     1 Ru                   -0.00008   1.53225   1.16704   Ru1  1 1
0.00000
HETATM     2 Ru                    5.40806   1.53113   1.16828   Ru1  1 1
0.00000
HETATM     3 Ru                    2.70344   1.53113   1.16828   Ru1  1 1
0.00000
HETATM     4 Ru                    6.75937   3.87188   1.16699   Ru1  1 1
0.00000
HETATM     5 Ru                    4.05575   3.87183   1.16814   Ru1  1 1
0.00000
HETATM     6 Ru                    1.35213   3.87188   1.16699   Ru1  1 1
0.00000
HETATM     7 Ru                    1.35224   0.75007   3.22834   Ru1  1 1
0.00000
HETATM     8 Ru                    6.75922   0.75007   3.22834   Ru1  1 1
0.00000
HETATM     9 Ru                    4.05572   0.74977   3.22796   Ru1  1 1
0.00000
```

HETATM 10 Ru 8.11156 3.09129 3.22684 Ru1 1 1
0.00000
HETATM 11 Ru 5.40811 3.09244 3.22650 Ru1 1 1
0.00000
HETATM 12 Ru 2.70334 3.09244 3.22649 Ru1 1 1
0.00000
HETATM 13 Ru 8.11152 1.53268 5.37280 Ru1 1 1
0.00000
HETATM 14 Ru 5.40723 1.53270 5.37339 Ru1 1 1
0.00000
HETATM 15 Ru 2.70413 1.53270 5.37339 Ru1 1 1
0.00000
HETATM 16 Ru 6.76091 3.87326 5.37203 Ru1 1 1
0.00000
HETATM 17 Ru 4.05568 3.87049 5.37267 Ru1 1 1
0.00000
HETATM 18 Ru 1.35045 3.87326 5.37203 Ru1 1 1
0.00000
HETATM 19 Ru 1.35365 0.75124 7.52558 Ru1 1 1
0.00000
HETATM 20 Ru 6.75763 0.75124 7.52558 Ru1 1 1
0.00000
HETATM 21 Ru 4.05564 0.75114 7.52854 Ru1 1 1
0.00000
HETATM 22 Ru 8.11147 3.09331 7.52654 Ru1 1 1
0.00000
HETATM 23 Ru 5.40574 3.09341 7.52958 Ru1 1 1
0.00000
HETATM 24 Ru 2.70554 3.09341 7.52958 Ru1 1 1
0.00000
HETATM 25 Ru 8.11143 1.53469 9.67561 Ru1 1 1
0.00000
HETATM 26 Ru 5.40881 1.53251 9.68617 Ru1 1 1
0.00000
HETATM 27 Ru 2.70239 1.53251 9.68616 Ru1 1 1
0.00000
HETATM 28 Ru 6.75847 3.87393 9.68332 Ru1 1 1
0.00000
HETATM 29 Ru 4.05560 3.87475 9.69373 Ru1 1 1
0.00000
HETATM 30 Ru 1.35273 3.87393 9.68332 Ru1 1 1
0.00000
HETATM 31 Ru 1.34520 0.75225 11.81937 Ru1 1 1
0.00000
HETATM 32 Ru 6.76593 0.75226 11.81937 Ru1 1 1
0.00000
HETATM 33 Ru 4.05556 0.75645 11.82648 Ru1 1 1
0.00000
HETATM 34 Ru 8.11140 3.09557 11.82719 Ru1 1 1
0.00000
HETATM 35 Ru 5.40922 3.09197 11.83512 Ru1 1 1
0.00000
HETATM 36 Ru 2.70191 3.09197 11.83511 Ru1 1 1
0.00000
HETATM 37 Ru 8.11138 1.54594 13.88377 Ru1 1 1
0.00000
HETATM 38 Ru 5.42436 1.52514 13.90623 Ru1 1 1
0.00000

```
HETATM    39 Ru                 2.68672   1.52514  13.90622   Ru1  1 1
0.00000
HETATM    40 Ru                 6.76581   3.87793  13.88407   Ru1  1 1
0.00000
HETATM    41 Ru                 4.05554   3.87542  13.90595   Ru1  1 1
0.00000
HETATM    42 Ru                 1.34528   3.87793  13.88407   Ru1  1 1
0.00000
HETATM    43 H                  4.05551   2.30871  14.97971    H1  1 1
0.00000
UNIT ENERGY   kcal
ENERGY        -8843.10916767442
END

XTLGRF 200
DESCRP Ruads1Hhcp
RUTYPE NORMAL RUN
#refdata /rf3/training/Ru_ads/1H/hcp
REMARK Created by geo_energy_extract.py EO = -380.17727588 eV; -
8841.331997209303 kcal/mol
CRYSTX     8.11167    4.68327   28.85554   90.00000   90.00000   90.00000
HETATM     1 Ru                -0.00024   1.53286   1.16876   Ru1  1 1
0.00000
HETATM     2 Ru                 5.40775   1.53287   1.16879   Ru1  1 1
0.00000
HETATM     3 Ru                 2.70381   1.53207   1.16922   Ru1  1 1
0.00000
HETATM     4 Ru                 6.75965   3.87233   1.16876   Ru1  1 1
0.00000
HETATM     5 Ru                 4.05514   3.87054   1.16911   Ru1  1 1
0.00000
HETATM     6 Ru                 1.35236   3.87054   1.16914   Ru1  1 1
0.00000
HETATM     7 Ru                 1.35165   0.75015   3.22793   Ru1  1 1
0.00000
HETATM     8 Ru                 6.75961   0.74946   3.23118   Ru1  1 1
0.00000
HETATM     9 Ru                 4.05583   0.75014   3.22794   Ru1  1 1
0.00000
HETATM    10 Ru                -0.00023   3.09206   3.22783   Ru1  1 1
0.00000
HETATM    11 Ru                 5.40771   3.09207   3.22782   Ru1  1 1
0.00000
HETATM    12 Ru                 2.70377   3.09290   3.22723   Ru1  1 1
0.00000
HETATM    13 Ru                 0.00018   1.53230   5.37167   Ru1  1 1
0.00000
HETATM    14 Ru                 5.40724   1.53230   5.37166   Ru1  1 1
0.00000
HETATM    15 Ru                 2.70374   1.53146   5.37053   Ru1  1 1
0.00000
HETATM    16 Ru                 6.75958   3.87349   5.37385   Ru1  1 1
0.00000
HETATM    17 Ru                 4.05548   3.87315   5.37276   Ru1  1 1
0.00000
HETATM    18 Ru                 1.35194   3.87314   5.37276   Ru1  1 1
0.00000
```

```
HETATM    19 Ru                    1.35363   0.75129   7.52884   Ru1  1 1
0.00000
HETATM    20 Ru                    6.75956   0.75119   7.52733   Ru1  1 1
0.00000
HETATM    21 Ru                    4.05371   0.75127   7.52880   Ru1  1 1
0.00000
HETATM    22 Ru                    0.00184   3.09336   7.52645   Ru1  1 1
0.00000
HETATM    23 Ru                    5.40553   3.09338   7.52649   Ru1  1 1
0.00000
HETATM    24 Ru                    2.70374   3.09341   7.53196   Ru1  1 1
0.00000
HETATM    25 Ru                    0.00059   1.53264   9.68106   Ru1  1 1
0.00000
HETATM    26 Ru                    5.40673   1.53263   9.68105   Ru1  1 1
0.00000
HETATM    27 Ru                    2.70369   1.53296   9.68858   Ru1  1 1
0.00000
HETATM    28 Ru                    6.75953   3.87480   9.68191   Ru1  1 1
0.00000
HETATM    29 Ru                    4.05489   3.87509   9.68957   Ru1  1 1
0.00000
HETATM    30 Ru                    1.35244   3.87509   9.68958   Ru1  1 1
0.00000
HETATM    31 Ru                    1.34829   0.75298  11.82287   Ru1  1 1
0.00000
HETATM    32 Ru                    6.75954   0.75468  11.82883   Ru1  1 1
0.00000
HETATM    33 Ru                    4.05899   0.75293  11.82286   Ru1  1 1
0.00000
HETATM    34 Ru                    8.10828   3.09486  11.82105   Ru1  1 1
0.00000
HETATM    35 Ru                    5.41066   3.09488  11.82108   Ru1  1 1
0.00000
HETATM    36 Ru                    2.70372   3.09215  11.83442   Ru1  1 1
0.00000
HETATM    37 Ru                    8.10294   1.52474  13.88134   Ru1  1 1
0.00000
HETATM    38 Ru                    5.41594   1.52471  13.88134   Ru1  1 1
0.00000
HETATM    39 Ru                    2.70366   1.52456  13.90949   Ru1  1 1
0.00000
HETATM    40 Ru                    6.75956   3.87601  13.88166   Ru1  1 1
0.00000
HETATM    41 Ru                    4.07614   3.89289  13.91116   Ru1  1 1
0.00000
HETATM    42 Ru                    1.33129   3.89294  13.91113   Ru1  1 1
0.00000
HETATM    43 H                     2.70369   3.10383  14.96712    H1  1 1
0.00000
UNIT ENERGY   kcal
ENERGY        -8841.331997209303
END

XTLGRF 200
DESCRP Ruads1Htop
RUTYPE NORMAL RUN
#refdata /rf3/training/Ru_ads/1H/top
```

REMARK Created by geo_energy_extract.py EO = -379.79659106 eV; -8832.478861860467 kcal/mol
CRYSTX 8.11167 4.68327 28.85554 90.00000 90.00000 90.00000
HETATM 1 Ru -0.00003 1.53123 1.16463 Ru1 1 1 0.00000
HETATM 2 Ru 5.40758 1.53124 1.16465 Ru1 1 1 0.00000
HETATM 3 Ru 2.70383 1.53007 1.16665 Ru1 1 1 0.00000
HETATM 4 Ru 6.75966 3.87323 1.16588 Ru1 1 1 0.00000
HETATM 5 Ru 4.05559 3.87220 1.16555 Ru1 1 1 0.00000
HETATM 6 Ru 1.35194 3.87220 1.16558 Ru1 1 1 0.00000
HETATM 7 Ru 1.35128 0.75083 3.22629 Ru1 1 1 0.00000
HETATM 8 Ru 6.75963 0.74971 3.22629 Ru1 1 1 0.00000
HETATM 9 Ru 4.05622 0.75082 3.22630 Ru1 1 1 0.00000
HETATM 10 Ru 8.11100 3.09127 3.22352 Ru1 1 1 0.00000
HETATM 11 Ru 5.40818 3.09129 3.22349 Ru1 1 1 0.00000
HETATM 12 Ru 2.70379 3.09196 3.22351 Ru1 1 1 0.00000
HETATM 13 Ru 8.11058 1.53288 5.36995 Ru1 1 1 0.00000
HETATM 14 Ru 5.40852 1.53288 5.36994 Ru1 1 1 0.00000
HETATM 15 Ru 2.70375 1.53138 5.37126 Ru1 1 1 0.00000
HETATM 16 Ru 6.75959 3.87346 5.37217 Ru1 1 1 0.00000
HETATM 17 Ru 4.05660 3.87203 5.36909 Ru1 1 1 0.00000
HETATM 18 Ru 1.35083 3.87203 5.36909 Ru1 1 1 0.00000
HETATM 19 Ru 1.35327 0.75355 7.52217 Ru1 1 1 0.00000
HETATM 20 Ru 6.75956 0.74993 7.52281 Ru1 1 1 0.00000
HETATM 21 Ru 4.05407 0.75354 7.52213 Ru1 1 1 0.00000
HETATM 22 Ru 8.11009 3.09300 7.52387 Ru1 1 1 0.00000
HETATM 23 Ru 5.40893 3.09302 7.52390 Ru1 1 1 0.00000
HETATM 24 Ru 2.70372 3.09140 7.52355 Ru1 1 1 0.00000
HETATM 25 Ru -0.00016 1.53299 9.67535 Ru1 1 1 0.00000
HETATM 26 Ru 5.40747 1.53298 9.67532 Ru1 1 1 0.00000
HETATM 27 Ru 2.70369 1.53185 9.68452 Ru1 1 1 0.00000

```
HETATM    28 Ru                     6.75953   3.87624   9.68131   Ru1  1 1
0.00000
HETATM    29 Ru                     4.05624   3.87460   9.68195   Ru1  1 1
0.00000
HETATM    30 Ru                     1.35106   3.87460   9.68195   Ru1  1 1
0.00000
HETATM    31 Ru                     1.34279   0.75024  11.81888   Ru1  1 1
0.00000
HETATM    32 Ru                     6.75955   0.75990  11.84140   Ru1  1 1
0.00000
HETATM    33 Ru                     4.06454   0.75020  11.81883   Ru1  1 1
0.00000
HETATM    34 Ru                     0.00245   3.09019  11.83057   Ru1  1 1
0.00000
HETATM    35 Ru                     5.40484   3.09023  11.83056   Ru1  1 1
0.00000
HETATM    36 Ru                     2.70371   3.10225  11.80715   Ru1  1 1
0.00000
HETATM    37 Ru                     8.10164   1.52942  13.88955   Ru1  1 1
0.00000
HETATM    38 Ru                     5.41722   1.52942  13.88952   Ru1  1 1
0.00000
HETATM    39 Ru                     2.70367   1.53596  13.89834   Ru1  1 1
0.00000
HETATM    40 Ru                     6.75952   3.87350  13.88629   Ru1  1 1
0.00000
HETATM    41 Ru                     4.06636   3.87854  13.87824   Ru1  1 1
0.00000
HETATM    42 Ru                     1.34095   3.87855  13.87825   Ru1  1 1
0.00000
HETATM    43 H                      2.70348   1.52801  15.54030    H1  1 1
0.00000
UNIT ENERGY   kcal
ENERGY        -8832.478861860467
END

XTLGRF 200
DESCRP Ruadsfccscand1
RUTYPE SINGLE POINT
#refdata /rf3/training/Ru_ads/fcc_scan/d1
REMARK Created by geo_energy_extract.py EO = -378.52521852 eV; -
8802.912058604652 kcal/mol
CRYSTX     8.11167    4.68327   28.85554   90.00000   90.00000   90.00000
HETATM     1 Ru                     0.00001   1.53078   1.16765   Ru1  1 1
0.00000
HETATM     2 Ru                     5.40780   1.53063   1.16776   Ru1  1 1
0.00000
HETATM     3 Ru                     2.70377   1.53065   1.16778   Ru1  1 1
0.00000
HETATM     4 Ru                     6.75965   3.87238   1.16767   Ru1  1 1
0.00000
HETATM     5 Ru                     4.05585   3.87230   1.16770   Ru1  1 1
0.00000
HETATM     6 Ru                     1.35193   3.87240   1.16769   Ru1  1 1
0.00000
HETATM     7 Ru                     1.35188   0.74928   3.22561   Ru1  1 1
0.00000
```

```
HETATM    8 Ru                  6.75970   0.74925   3.22561   Ru1  1 1
0.00000
HETATM    9 Ru                  4.05578   0.74927   3.22565   Ru1  1 1
0.00000
HETATM   10 Ru                  8.11158   3.09079   3.22563   Ru1  1 1
0.00000
HETATM   11 Ru                  5.40774   3.09088   3.22561   Ru1  1 1
0.00000
HETATM   12 Ru                  2.70386   3.09086   3.22562   Ru1  1 1
0.00000
HETATM   13 Ru                  8.11157   1.53090   5.37021   Ru1  1 1
0.00000
HETATM   14 Ru                  5.40770   1.53100   5.37022   Ru1  1 1
0.00000
HETATM   15 Ru                  2.70374   1.53096   5.37025   Ru1  1 1
0.00000
HETATM   16 Ru                  6.75960   3.87259   5.37017   Ru1  1 1
0.00000
HETATM   17 Ru                  4.05577   3.87276   5.37033   Ru1  1 1
0.00000
HETATM   18 Ru                  1.35188   3.87253   5.37021   Ru1  1 1
0.00000
HETATM   19 Ru                  1.35179   0.75177   7.52423   Ru1  1 1
0.00000
HETATM   20 Ru                  6.75945   0.75172   7.52425   Ru1  1 1
0.00000
HETATM   21 Ru                  4.05563   0.75194   7.52422   Ru1  1 1
0.00000
HETATM   22 Ru                  8.11145   3.09335   7.52406   Ru1  1 1
0.00000
HETATM   23 Ru                  5.40761   3.09354   7.52428   Ru1  1 1
0.00000
HETATM   24 Ru                  2.70362   3.09353   7.52431   Ru1  1 1
0.00000
HETATM   25 Ru                  8.11133   1.53310   9.67959   Ru1  1 1
0.00000
HETATM   26 Ru                  5.40756   1.53303   9.67970   Ru1  1 1
0.00000
HETATM   27 Ru                  2.70349   1.53295   9.67962   Ru1  1 1
0.00000
HETATM   28 Ru                  6.75949   3.87462   9.67967   Ru1  1 1
0.00000
HETATM   29 Ru                  4.05549   3.87464   9.67956   Ru1  1 1
0.00000
HETATM   30 Ru                  1.35147   3.87460   9.67959   Ru1  1 1
0.00000
HETATM   31 Ru                  1.35144   0.75297  11.80872   Ru1  1 1
0.00000
HETATM   32 Ru                  6.75937   0.75299  11.80875   Ru1  1 1
0.00000
HETATM   33 Ru                  4.05542   0.75288  11.80858   Ru1  1 1
0.00000
HETATM   34 Ru                  8.11131   3.09448  11.80877   Ru1  1 1
0.00000
HETATM   35 Ru                  5.40736   3.09454  11.80863   Ru1  1 1
0.00000
HETATM   36 Ru                  2.70345   3.09458  11.80861   Ru1  1 1
0.00000
```

```
HETATM    37 Ru                    8.11140   1.53440  13.92754   Ru1  1 1
0.00000
HETATM    38 Ru                    5.40759   1.53396  13.92717   Ru1  1 1
0.00000
HETATM    39 Ru                    2.70370   1.53388  13.92722   Ru1  1 1
0.00000
HETATM    40 Ru                    6.75960   3.87584  13.92742   Ru1  1 1
0.00000
HETATM    41 Ru                    4.05571   3.87548  13.92716   Ru1  1 1
0.00000
HETATM    42 Ru                    1.35176   3.87588  13.92751   Ru1  1 1
0.00000
HETATM    43 H                     4.05536   2.31309  13.93355    H1  1 1
0.00000
UNIT ENERGY   kcal
ENERGY        -8802.912058604652
END

XTLGRF 200
DESCRP Ruadsfccscand2
RUTYPE SINGLE POINT
#refdata /rf3/training/Ru_ads/fcc_scan/d2
REMARK Created by geo_energy_extract.py EO = -379.58503773 eV; -
8827.559016976746 kcal/mol
CRYSTX     8.11167    4.68327   28.85554   90.00000   90.00000   90.00000
HETATM     1 Ru                    0.00001   1.53078   1.16765   Ru1  1 1
0.00000
HETATM     2 Ru                    5.40780   1.53063   1.16776   Ru1  1 1
0.00000
HETATM     3 Ru                    2.70377   1.53065   1.16778   Ru1  1 1
0.00000
HETATM     4 Ru                    6.75965   3.87238   1.16767   Ru1  1 1
0.00000
HETATM     5 Ru                    4.05585   3.87230   1.16770   Ru1  1 1
0.00000
HETATM     6 Ru                    1.35193   3.87240   1.16769   Ru1  1 1
0.00000
HETATM     7 Ru                    1.35188   0.74928   3.22561   Ru1  1 1
0.00000
HETATM     8 Ru                    6.75970   0.74925   3.22561   Ru1  1 1
0.00000
HETATM     9 Ru                    4.05578   0.74927   3.22565   Ru1  1 1
0.00000
HETATM    10 Ru                    8.11158   3.09079   3.22563   Ru1  1 1
0.00000
HETATM    11 Ru                    5.40774   3.09088   3.22561   Ru1  1 1
0.00000
HETATM    12 Ru                    2.70386   3.09086   3.22562   Ru1  1 1
0.00000
HETATM    13 Ru                    8.11157   1.53090   5.37021   Ru1  1 1
0.00000
HETATM    14 Ru                    5.40770   1.53100   5.37022   Ru1  1 1
0.00000
HETATM    15 Ru                    2.70374   1.53096   5.37025   Ru1  1 1
0.00000
HETATM    16 Ru                    6.75960   3.87259   5.37017   Ru1  1 1
0.00000
```

```
HETATM    17 Ru                    4.05577   3.87276   5.37033   Ru1  1 1
0.00000
HETATM    18 Ru                    1.35188   3.87253   5.37021   Ru1  1 1
0.00000
HETATM    19 Ru                    1.35179   0.75177   7.52423   Ru1  1 1
0.00000
HETATM    20 Ru                    6.75945   0.75172   7.52425   Ru1  1 1
0.00000
HETATM    21 Ru                    4.05563   0.75194   7.52422   Ru1  1 1
0.00000
HETATM    22 Ru                    8.11145   3.09335   7.52406   Ru1  1 1
0.00000
HETATM    23 Ru                    5.40761   3.09354   7.52428   Ru1  1 1
0.00000
HETATM    24 Ru                    2.70362   3.09353   7.52431   Ru1  1 1
0.00000
HETATM    25 Ru                    8.11133   1.53310   9.67959   Ru1  1 1
0.00000
HETATM    26 Ru                    5.40756   1.53303   9.67970   Ru1  1 1
0.00000
HETATM    27 Ru                    2.70349   1.53295   9.67962   Ru1  1 1
0.00000
HETATM    28 Ru                    6.75949   3.87462   9.67967   Ru1  1 1
0.00000
HETATM    29 Ru                    4.05549   3.87464   9.67956   Ru1  1 1
0.00000
HETATM    30 Ru                    1.35147   3.87460   9.67959   Ru1  1 1
0.00000
HETATM    31 Ru                    1.35144   0.75297  11.80872   Ru1  1 1
0.00000
HETATM    32 Ru                    6.75937   0.75299  11.80875   Ru1  1 1
0.00000
HETATM    33 Ru                    4.05542   0.75288  11.80858   Ru1  1 1
0.00000
HETATM    34 Ru                    8.11131   3.09448  11.80877   Ru1  1 1
0.00000
HETATM    35 Ru                    5.40736   3.09454  11.80863   Ru1  1 1
0.00000
HETATM    36 Ru                    2.70345   3.09458  11.80861   Ru1  1 1
0.00000
HETATM    37 Ru                    8.11140   1.53440  13.92754   Ru1  1 1
0.00000
HETATM    38 Ru                    5.40759   1.53396  13.92717   Ru1  1 1
0.00000
HETATM    39 Ru                    2.70370   1.53388  13.92722   Ru1  1 1
0.00000
HETATM    40 Ru                    6.75960   3.87584  13.92742   Ru1  1 1
0.00000
HETATM    41 Ru                    4.05571   3.87548  13.92716   Ru1  1 1
0.00000
HETATM    42 Ru                    1.35176   3.87588  13.92751   Ru1  1 1
0.00000
HETATM    43 H                     4.05536   2.31309  14.51066    H1  1 1
0.00000
UNIT ENERGY   kcal
ENERGY        -8827.559016976746
END
```

```
XTLGRF 200
DESCRP Ruadsfccscand3
RUTYPE SINGLE POINT
#refdata /rf3/training/Ru_ads/fcc_scan/d3
REMARK Created by geo_energy_extract.py EO = -380.14637526 eV; -
8840.613378139537 kcal/mol
CRYSTX     8.11167    4.68327   28.85554   90.00000   90.00000   90.00000
HETATM     1 Ru                  0.00001   1.53078   1.16765   Ru1  1 1
0.00000
HETATM     2 Ru                  5.40780   1.53063   1.16776   Ru1  1 1
0.00000
HETATM     3 Ru                  2.70377   1.53065   1.16778   Ru1  1 1
0.00000
HETATM     4 Ru                  6.75965   3.87238   1.16767   Ru1  1 1
0.00000
HETATM     5 Ru                  4.05585   3.87230   1.16770   Ru1  1 1
0.00000
HETATM     6 Ru                  1.35193   3.87240   1.16769   Ru1  1 1
0.00000
HETATM     7 Ru                  1.35188   0.74928   3.22561   Ru1  1 1
0.00000
HETATM     8 Ru                  6.75970   0.74925   3.22561   Ru1  1 1
0.00000
HETATM     9 Ru                  4.05578   0.74927   3.22565   Ru1  1 1
0.00000
HETATM    10 Ru                  8.11158   3.09079   3.22563   Ru1  1 1
0.00000
HETATM    11 Ru                  5.40774   3.09088   3.22561   Ru1  1 1
0.00000
HETATM    12 Ru                  2.70386   3.09086   3.22562   Ru1  1 1
0.00000
HETATM    13 Ru                  8.11157   1.53090   5.37021   Ru1  1 1
0.00000
HETATM    14 Ru                  5.40770   1.53100   5.37022   Ru1  1 1
0.00000
HETATM    15 Ru                  2.70374   1.53096   5.37025   Ru1  1 1
0.00000
HETATM    16 Ru                  6.75960   3.87259   5.37017   Ru1  1 1
0.00000
HETATM    17 Ru                  4.05577   3.87276   5.37033   Ru1  1 1
0.00000
HETATM    18 Ru                  1.35188   3.87253   5.37021   Ru1  1 1
0.00000
HETATM    19 Ru                  1.35179   0.75177   7.52423   Ru1  1 1
0.00000
HETATM    20 Ru                  6.75945   0.75172   7.52425   Ru1  1 1
0.00000
HETATM    21 Ru                  4.05563   0.75194   7.52422   Ru1  1 1
0.00000
HETATM    22 Ru                  8.11145   3.09335   7.52406   Ru1  1 1
0.00000
HETATM    23 Ru                  5.40761   3.09354   7.52428   Ru1  1 1
0.00000
HETATM    24 Ru                  2.70362   3.09353   7.52431   Ru1  1 1
0.00000
HETATM    25 Ru                  8.11133   1.53310   9.67959   Ru1  1 1
0.00000
```

```
HETATM    26 Ru                   5.40756   1.53303   9.67970   Ru1  1 1
0.00000
HETATM    27 Ru                   2.70349   1.53295   9.67962   Ru1  1 1
0.00000
HETATM    28 Ru                   6.75949   3.87462   9.67967   Ru1  1 1
0.00000
HETATM    29 Ru                   4.05549   3.87464   9.67956   Ru1  1 1
0.00000
HETATM    30 Ru                   1.35147   3.87460   9.67959   Ru1  1 1
0.00000
HETATM    31 Ru                   1.35144   0.75297  11.80872   Ru1  1 1
0.00000
HETATM    32 Ru                   6.75937   0.75299  11.80875   Ru1  1 1
0.00000
HETATM    33 Ru                   4.05542   0.75288  11.80858   Ru1  1 1
0.00000
HETATM    34 Ru                   8.11131   3.09448  11.80877   Ru1  1 1
0.00000
HETATM    35 Ru                   5.40736   3.09454  11.80863   Ru1  1 1
0.00000
HETATM    36 Ru                   2.70345   3.09458  11.80861   Ru1  1 1
0.00000
HETATM    37 Ru                   8.11140   1.53440  13.92754   Ru1  1 1
0.00000
HETATM    38 Ru                   5.40759   1.53396  13.92717   Ru1  1 1
0.00000
HETATM    39 Ru                   2.70370   1.53388  13.92722   Ru1  1 1
0.00000
HETATM    40 Ru                   6.75960   3.87584  13.92742   Ru1  1 1
0.00000
HETATM    41 Ru                   4.05571   3.87548  13.92716   Ru1  1 1
0.00000
HETATM    42 Ru                   1.35176   3.87588  13.92751   Ru1  1 1
0.00000
HETATM    43 H                    4.05536   2.31309  15.08777    H1  1 1
0.00000
UNIT ENERGY   kcal
ENERGY        -8840.613378139537
END

XTLGRF 200
DESCRP Ruadsfccscand4
RUTYPE SINGLE POINT
#refdata /rf3/training/Ru_ads/fcc_scan/d4
REMARK Created by geo_energy_extract.py EO = -379.38634474 eV; -
8822.938249767443 kcal/mol
CRYSTX     8.11167    4.68327   28.85554   90.00000   90.00000   90.00000
HETATM     1 Ru                   0.00001   1.53078   1.16765   Ru1  1 1
0.00000
HETATM     2 Ru                   5.40780   1.53063   1.16776   Ru1  1 1
0.00000
HETATM     3 Ru                   2.70377   1.53065   1.16778   Ru1  1 1
0.00000
HETATM     4 Ru                   6.75965   3.87238   1.16767   Ru1  1 1
0.00000
HETATM     5 Ru                   4.05585   3.87230   1.16770   Ru1  1 1
0.00000
```

```
HETATM     6 Ru                    1.35193   3.87240   1.16769   Ru1  1 1
0.00000
HETATM     7 Ru                    1.35188   0.74928   3.22561   Ru1  1 1
0.00000
HETATM     8 Ru                    6.75970   0.74925   3.22561   Ru1  1 1
0.00000
HETATM     9 Ru                    4.05578   0.74927   3.22565   Ru1  1 1
0.00000
HETATM    10 Ru                    8.11158   3.09079   3.22563   Ru1  1 1
0.00000
HETATM    11 Ru                    5.40774   3.09088   3.22561   Ru1  1 1
0.00000
HETATM    12 Ru                    2.70386   3.09086   3.22562   Ru1  1 1
0.00000
HETATM    13 Ru                    8.11157   1.53090   5.37021   Ru1  1 1
0.00000
HETATM    14 Ru                    5.40770   1.53100   5.37022   Ru1  1 1
0.00000
HETATM    15 Ru                    2.70374   1.53096   5.37025   Ru1  1 1
0.00000
HETATM    16 Ru                    6.75960   3.87259   5.37017   Ru1  1 1
0.00000
HETATM    17 Ru                    4.05577   3.87276   5.37033   Ru1  1 1
0.00000
HETATM    18 Ru                    1.35188   3.87253   5.37021   Ru1  1 1
0.00000
HETATM    19 Ru                    1.35179   0.75177   7.52423   Ru1  1 1
0.00000
HETATM    20 Ru                    6.75945   0.75172   7.52425   Ru1  1 1
0.00000
HETATM    21 Ru                    4.05563   0.75194   7.52422   Ru1  1 1
0.00000
HETATM    22 Ru                    8.11145   3.09335   7.52406   Ru1  1 1
0.00000
HETATM    23 Ru                    5.40761   3.09354   7.52428   Ru1  1 1
0.00000
HETATM    24 Ru                    2.70362   3.09353   7.52431   Ru1  1 1
0.00000
HETATM    25 Ru                    8.11133   1.53310   9.67959   Ru1  1 1
0.00000
HETATM    26 Ru                    5.40756   1.53303   9.67970   Ru1  1 1
0.00000
HETATM    27 Ru                    2.70349   1.53295   9.67962   Ru1  1 1
0.00000
HETATM    28 Ru                    6.75949   3.87462   9.67967   Ru1  1 1
0.00000
HETATM    29 Ru                    4.05549   3.87464   9.67956   Ru1  1 1
0.00000
HETATM    30 Ru                    1.35147   3.87460   9.67959   Ru1  1 1
0.00000
HETATM    31 Ru                    1.35144   0.75297  11.80872   Ru1  1 1
0.00000
HETATM    32 Ru                    6.75937   0.75299  11.80875   Ru1  1 1
0.00000
HETATM    33 Ru                    4.05542   0.75288  11.80858   Ru1  1 1
0.00000
HETATM    34 Ru                    8.11131   3.09448  11.80877   Ru1  1 1
0.00000
```

```
HETATM    35 Ru                   5.40736   3.09454  11.80863   Ru1  1 1
0.00000
HETATM    36 Ru                   2.70345   3.09458  11.80861   Ru1  1 1
0.00000
HETATM    37 Ru                   8.11140   1.53440  13.92754   Ru1  1 1
0.00000
HETATM    38 Ru                   5.40759   1.53396  13.92717   Ru1  1 1
0.00000
HETATM    39 Ru                   2.70370   1.53388  13.92722   Ru1  1 1
0.00000
HETATM    40 Ru                   6.75960   3.87584  13.92742   Ru1  1 1
0.00000
HETATM    41 Ru                   4.05571   3.87548  13.92716   Ru1  1 1
0.00000
HETATM    42 Ru                   1.35176   3.87588  13.92751   Ru1  1 1
0.00000
HETATM    43 H                    4.05536   2.31309  15.66488    H1  1 1
0.00000
UNIT ENERGY   kcal
ENERGY        -8822.938249767443
END

XTLGRF 200
DESCRP Ruadsfccscand5
RUTYPE SINGLE POINT
#refdata /rf3/training/Ru_ads/fcc_scan/d5
REMARK Created by geo_energy_extract.py EO = -378.24997994 eV; -
8796.51116139535 kcal/mol
CRYSTX     8.11167    4.68327   28.85554   90.00000   90.00000   90.00000
HETATM     1 Ru                   0.00001   1.53078   1.16765   Ru1  1 1
0.00000
HETATM     2 Ru                   5.40780   1.53063   1.16776   Ru1  1 1
0.00000
HETATM     3 Ru                   2.70377   1.53065   1.16778   Ru1  1 1
0.00000
HETATM     4 Ru                   6.75965   3.87238   1.16767   Ru1  1 1
0.00000
HETATM     5 Ru                   4.05585   3.87230   1.16770   Ru1  1 1
0.00000
HETATM     6 Ru                   1.35193   3.87240   1.16769   Ru1  1 1
0.00000
HETATM     7 Ru                   1.35188   0.74928   3.22561   Ru1  1 1
0.00000
HETATM     8 Ru                   6.75970   0.74925   3.22561   Ru1  1 1
0.00000
HETATM     9 Ru                   4.05578   0.74927   3.22565   Ru1  1 1
0.00000
HETATM    10 Ru                   8.11158   3.09079   3.22563   Ru1  1 1
0.00000
HETATM    11 Ru                   5.40774   3.09088   3.22561   Ru1  1 1
0.00000
HETATM    12 Ru                   2.70386   3.09086   3.22562   Ru1  1 1
0.00000
HETATM    13 Ru                   8.11157   1.53090   5.37021   Ru1  1 1
0.00000
HETATM    14 Ru                   5.40770   1.53100   5.37022   Ru1  1 1
0.00000
```

```
HETATM    15 Ru                   2.70374   1.53096   5.37025   Ru1  1 1
0.00000
HETATM    16 Ru                   6.75960   3.87259   5.37017   Ru1  1 1
0.00000
HETATM    17 Ru                   4.05577   3.87276   5.37033   Ru1  1 1
0.00000
HETATM    18 Ru                   1.35188   3.87253   5.37021   Ru1  1 1
0.00000
HETATM    19 Ru                   1.35179   0.75177   7.52423   Ru1  1 1
0.00000
HETATM    20 Ru                   6.75945   0.75172   7.52425   Ru1  1 1
0.00000
HETATM    21 Ru                   4.05563   0.75194   7.52422   Ru1  1 1
0.00000
HETATM    22 Ru                   8.11145   3.09335   7.52406   Ru1  1 1
0.00000
HETATM    23 Ru                   5.40761   3.09354   7.52428   Ru1  1 1
0.00000
HETATM    24 Ru                   2.70362   3.09353   7.52431   Ru1  1 1
0.00000
HETATM    25 Ru                   8.11133   1.53310   9.67959   Ru1  1 1
0.00000
HETATM    26 Ru                   5.40756   1.53303   9.67970   Ru1  1 1
0.00000
HETATM    27 Ru                   2.70349   1.53295   9.67962   Ru1  1 1
0.00000
HETATM    28 Ru                   6.75949   3.87462   9.67967   Ru1  1 1
0.00000
HETATM    29 Ru                   4.05549   3.87464   9.67956   Ru1  1 1
0.00000
HETATM    30 Ru                   1.35147   3.87460   9.67959   Ru1  1 1
0.00000
HETATM    31 Ru                   1.35144   0.75297  11.80872   Ru1  1 1
0.00000
HETATM    32 Ru                   6.75937   0.75299  11.80875   Ru1  1 1
0.00000
HETATM    33 Ru                   4.05542   0.75288  11.80858   Ru1  1 1
0.00000
HETATM    34 Ru                   8.11131   3.09448  11.80877   Ru1  1 1
0.00000
HETATM    35 Ru                   5.40736   3.09454  11.80863   Ru1  1 1
0.00000
HETATM    36 Ru                   2.70345   3.09458  11.80861   Ru1  1 1
0.00000
HETATM    37 Ru                   8.11140   1.53440  13.92754   Ru1  1 1
0.00000
HETATM    38 Ru                   5.40759   1.53396  13.92717   Ru1  1 1
0.00000
HETATM    39 Ru                   2.70370   1.53388  13.92722   Ru1  1 1
0.00000
HETATM    40 Ru                   6.75960   3.87584  13.92742   Ru1  1 1
0.00000
HETATM    41 Ru                   4.05571   3.87548  13.92716   Ru1  1 1
0.00000
HETATM    42 Ru                   1.35176   3.87588  13.92751   Ru1  1 1
0.00000
HETATM    43 H                    4.05536   2.31309  16.24199    H1  1 1
0.00000
```

```
UNIT ENERGY   kcal
ENERGY        -8796.51116139535
END

XTLGRF 200
DESCRP Ruadshcpscand1
RUTYPE SINGLE POINT
#refdata /rf3/training/Ru_ads/hcp_scan/d1
REMARK Created by geo_energy_extract.py EO = -378.42763171 eV; -
8800.642597906977 kcal/mol
CRYSTX     8.11167    4.68327   28.85554   90.00000   90.00000   90.00000
HETATM     1 Ru                  0.00001   1.53078   1.16765   Ru1  1 1
0.00000
HETATM     2 Ru                  5.40780   1.53063   1.16776   Ru1  1 1
0.00000
HETATM     3 Ru                  2.70377   1.53065   1.16778   Ru1  1 1
0.00000
HETATM     4 Ru                  6.75965   3.87238   1.16767   Ru1  1 1
0.00000
HETATM     5 Ru                  4.05585   3.87230   1.16770   Ru1  1 1
0.00000
HETATM     6 Ru                  1.35193   3.87240   1.16769   Ru1  1 1
0.00000
HETATM     7 Ru                  1.35188   0.74928   3.22561   Ru1  1 1
0.00000
HETATM     8 Ru                  6.75970   0.74925   3.22561   Ru1  1 1
0.00000
HETATM     9 Ru                  4.05578   0.74927   3.22565   Ru1  1 1
0.00000
HETATM    10 Ru                  8.11158   3.09079   3.22563   Ru1  1 1
0.00000
HETATM    11 Ru                  5.40774   3.09088   3.22561   Ru1  1 1
0.00000
HETATM    12 Ru                  2.70386   3.09086   3.22562   Ru1  1 1
0.00000
HETATM    13 Ru                  8.11157   1.53090   5.37021   Ru1  1 1
0.00000
HETATM    14 Ru                  5.40770   1.53100   5.37022   Ru1  1 1
0.00000
HETATM    15 Ru                  2.70374   1.53096   5.37025   Ru1  1 1
0.00000
HETATM    16 Ru                  6.75960   3.87259   5.37017   Ru1  1 1
0.00000
HETATM    17 Ru                  4.05577   3.87276   5.37033   Ru1  1 1
0.00000
HETATM    18 Ru                  1.35188   3.87253   5.37021   Ru1  1 1
0.00000
HETATM    19 Ru                  1.35179   0.75177   7.52423   Ru1  1 1
0.00000
HETATM    20 Ru                  6.75945   0.75172   7.52425   Ru1  1 1
0.00000
HETATM    21 Ru                  4.05563   0.75194   7.52422   Ru1  1 1
0.00000
HETATM    22 Ru                  8.11145   3.09335   7.52406   Ru1  1 1
0.00000
HETATM    23 Ru                  5.40761   3.09354   7.52428   Ru1  1 1
0.00000
```

```
HETATM    24 Ru                    2.70362   3.09353   7.52431   Ru1  1 1
0.00000
HETATM    25 Ru                    8.11133   1.53310   9.67959   Ru1  1 1
0.00000
HETATM    26 Ru                    5.40756   1.53303   9.67970   Ru1  1 1
0.00000
HETATM    27 Ru                    2.70349   1.53295   9.67962   Ru1  1 1
0.00000
HETATM    28 Ru                    6.75949   3.87462   9.67967   Ru1  1 1
0.00000
HETATM    29 Ru                    4.05549   3.87464   9.67956   Ru1  1 1
0.00000
HETATM    30 Ru                    1.35147   3.87460   9.67959   Ru1  1 1
0.00000
HETATM    31 Ru                    1.35144   0.75297  11.80872   Ru1  1 1
0.00000
HETATM    32 Ru                    6.75937   0.75299  11.80875   Ru1  1 1
0.00000
HETATM    33 Ru                    4.05542   0.75288  11.80858   Ru1  1 1
0.00000
HETATM    34 Ru                    8.11131   3.09448  11.80877   Ru1  1 1
0.00000
HETATM    35 Ru                    5.40736   3.09454  11.80863   Ru1  1 1
0.00000
HETATM    36 Ru                    2.70345   3.09458  11.80861   Ru1  1 1
0.00000
HETATM    37 Ru                    8.11140   1.53440  13.92754   Ru1  1 1
0.00000
HETATM    38 Ru                    5.40759   1.53396  13.92717   Ru1  1 1
0.00000
HETATM    39 Ru                    2.70370   1.53388  13.92722   Ru1  1 1
0.00000
HETATM    40 Ru                    6.75960   3.87584  13.92742   Ru1  1 1
0.00000
HETATM    41 Ru                    4.05571   3.87548  13.92716   Ru1  1 1
0.00000
HETATM    42 Ru                    1.35176   3.87588  13.92751   Ru1  1 1
0.00000
HETATM    43 H                     2.70389   3.12218  13.93355    H1  1 1
0.00000
UNIT ENERGY   kcal
ENERGY        -8800.642597906977
END

XTLGRF 200
DESCRP Ruadshcpscand2
RUTYPE SINGLE POINT
#refdata /rf3/training/Ru_ads/hcp_scan/d2
REMARK Created by geo_energy_extract.py EO = -379.52028307 eV; -
8826.053094651164 kcal/mol
CRYSTX     8.11167    4.68327   28.85554   90.00000   90.00000   90.00000
HETATM     1 Ru                    0.00001   1.53078   1.16765   Ru1  1 1
0.00000
HETATM     2 Ru                    5.40780   1.53063   1.16776   Ru1  1 1
0.00000
HETATM     3 Ru                    2.70377   1.53065   1.16778   Ru1  1 1
0.00000
```

```
HETATM    4 Ru                      6.75965   3.87238   1.16767   Ru1  1 1
0.00000
HETATM    5 Ru                      4.05585   3.87230   1.16770   Ru1  1 1
0.00000
HETATM    6 Ru                      1.35193   3.87240   1.16769   Ru1  1 1
0.00000
HETATM    7 Ru                      1.35188   0.74928   3.22561   Ru1  1 1
0.00000
HETATM    8 Ru                      6.75970   0.74925   3.22561   Ru1  1 1
0.00000
HETATM    9 Ru                      4.05578   0.74927   3.22565   Ru1  1 1
0.00000
HETATM   10 Ru                      8.11158   3.09079   3.22563   Ru1  1 1
0.00000
HETATM   11 Ru                      5.40774   3.09088   3.22561   Ru1  1 1
0.00000
HETATM   12 Ru                      2.70386   3.09086   3.22562   Ru1  1 1
0.00000
HETATM   13 Ru                      8.11157   1.53090   5.37021   Ru1  1 1
0.00000
HETATM   14 Ru                      5.40770   1.53100   5.37022   Ru1  1 1
0.00000
HETATM   15 Ru                      2.70374   1.53096   5.37025   Ru1  1 1
0.00000
HETATM   16 Ru                      6.75960   3.87259   5.37017   Ru1  1 1
0.00000
HETATM   17 Ru                      4.05577   3.87276   5.37033   Ru1  1 1
0.00000
HETATM   18 Ru                      1.35188   3.87253   5.37021   Ru1  1 1
0.00000
HETATM   19 Ru                      1.35179   0.75177   7.52423   Ru1  1 1
0.00000
HETATM   20 Ru                      6.75945   0.75172   7.52425   Ru1  1 1
0.00000
HETATM   21 Ru                      4.05563   0.75194   7.52422   Ru1  1 1
0.00000
HETATM   22 Ru                      8.11145   3.09335   7.52406   Ru1  1 1
0.00000
HETATM   23 Ru                      5.40761   3.09354   7.52428   Ru1  1 1
0.00000
HETATM   24 Ru                      2.70362   3.09353   7.52431   Ru1  1 1
0.00000
HETATM   25 Ru                      8.11133   1.53310   9.67959   Ru1  1 1
0.00000
HETATM   26 Ru                      5.40756   1.53303   9.67970   Ru1  1 1
0.00000
HETATM   27 Ru                      2.70349   1.53295   9.67962   Ru1  1 1
0.00000
HETATM   28 Ru                      6.75949   3.87462   9.67967   Ru1  1 1
0.00000
HETATM   29 Ru                      4.05549   3.87464   9.67956   Ru1  1 1
0.00000
HETATM   30 Ru                      1.35147   3.87460   9.67959   Ru1  1 1
0.00000
HETATM   31 Ru                      1.35144   0.75297  11.80872   Ru1  1 1
0.00000
HETATM   32 Ru                      6.75937   0.75299  11.80875   Ru1  1 1
0.00000
```

```
HETATM    33 Ru                  4.05542   0.75288  11.80858   Ru1  1 1
0.00000
HETATM    34 Ru                  8.11131   3.09448  11.80877   Ru1  1 1
0.00000
HETATM    35 Ru                  5.40736   3.09454  11.80863   Ru1  1 1
0.00000
HETATM    36 Ru                  2.70345   3.09458  11.80861   Ru1  1 1
0.00000
HETATM    37 Ru                  8.11140   1.53440  13.92754   Ru1  1 1
0.00000
HETATM    38 Ru                  5.40759   1.53396  13.92717   Ru1  1 1
0.00000
HETATM    39 Ru                  2.70370   1.53388  13.92722   Ru1  1 1
0.00000
HETATM    40 Ru                  6.75960   3.87584  13.92742   Ru1  1 1
0.00000
HETATM    41 Ru                  4.05571   3.87548  13.92716   Ru1  1 1
0.00000
HETATM    42 Ru                  1.35176   3.87588  13.92751   Ru1  1 1
0.00000
HETATM    43 H                   2.70389   3.12218  14.51066    H1  1 1
0.00000
UNIT ENERGY   kcal
ENERGY        -8826.053094651164
END

XTLGRF 200
DESCRP Ruadshcpscand3
RUTYPE SINGLE POINT
#refdata /rf3/training/Ru_ads/hcp_scan/d3
REMARK Created by geo_energy_extract.py EO = -380.07165737 eV; -
8838.875752790698 kcal/mol
CRYSTX     8.11167    4.68327   28.85554   90.00000   90.00000   90.00000
HETATM     1 Ru                  0.00001   1.53078   1.16765   Ru1  1 1
0.00000
HETATM     2 Ru                  5.40780   1.53063   1.16776   Ru1  1 1
0.00000
HETATM     3 Ru                  2.70377   1.53065   1.16778   Ru1  1 1
0.00000
HETATM     4 Ru                  6.75965   3.87238   1.16767   Ru1  1 1
0.00000
HETATM     5 Ru                  4.05585   3.87230   1.16770   Ru1  1 1
0.00000
HETATM     6 Ru                  1.35193   3.87240   1.16769   Ru1  1 1
0.00000
HETATM     7 Ru                  1.35188   0.74928   3.22561   Ru1  1 1
0.00000
HETATM     8 Ru                  6.75970   0.74925   3.22561   Ru1  1 1
0.00000
HETATM     9 Ru                  4.05578   0.74927   3.22565   Ru1  1 1
0.00000
HETATM    10 Ru                  8.11158   3.09079   3.22563   Ru1  1 1
0.00000
HETATM    11 Ru                  5.40774   3.09088   3.22561   Ru1  1 1
0.00000
HETATM    12 Ru                  2.70386   3.09086   3.22562   Ru1  1 1
0.00000
```

HETATM 13 Ru 8.11157 1.53090 5.37021 Ru1 1 1
0.00000
HETATM 14 Ru 5.40770 1.53100 5.37022 Ru1 1 1
0.00000
HETATM 15 Ru 2.70374 1.53096 5.37025 Ru1 1 1
0.00000
HETATM 16 Ru 6.75960 3.87259 5.37017 Ru1 1 1
0.00000
HETATM 17 Ru 4.05577 3.87276 5.37033 Ru1 1 1
0.00000
HETATM 18 Ru 1.35188 3.87253 5.37021 Ru1 1 1
0.00000
HETATM 19 Ru 1.35179 0.75177 7.52423 Ru1 1 1
0.00000
HETATM 20 Ru 6.75945 0.75172 7.52425 Ru1 1 1
0.00000
HETATM 21 Ru 4.05563 0.75194 7.52422 Ru1 1 1
0.00000
HETATM 22 Ru 8.11145 3.09335 7.52406 Ru1 1 1
0.00000
HETATM 23 Ru 5.40761 3.09354 7.52428 Ru1 1 1
0.00000
HETATM 24 Ru 2.70362 3.09353 7.52431 Ru1 1 1
0.00000
HETATM 25 Ru 8.11133 1.53310 9.67959 Ru1 1 1
0.00000
HETATM 26 Ru 5.40756 1.53303 9.67970 Ru1 1 1
0.00000
HETATM 27 Ru 2.70349 1.53295 9.67962 Ru1 1 1
0.00000
HETATM 28 Ru 6.75949 3.87462 9.67967 Ru1 1 1
0.00000
HETATM 29 Ru 4.05549 3.87464 9.67956 Ru1 1 1
0.00000
HETATM 30 Ru 1.35147 3.87460 9.67959 Ru1 1 1
0.00000
HETATM 31 Ru 1.35144 0.75297 11.80872 Ru1 1 1
0.00000
HETATM 32 Ru 6.75937 0.75299 11.80875 Ru1 1 1
0.00000
HETATM 33 Ru 4.05542 0.75288 11.80858 Ru1 1 1
0.00000
HETATM 34 Ru 8.11131 3.09448 11.80877 Ru1 1 1
0.00000
HETATM 35 Ru 5.40736 3.09454 11.80863 Ru1 1 1
0.00000
HETATM 36 Ru 2.70345 3.09458 11.80861 Ru1 1 1
0.00000
HETATM 37 Ru 8.11140 1.53440 13.92754 Ru1 1 1
0.00000
HETATM 38 Ru 5.40759 1.53396 13.92717 Ru1 1 1
0.00000
HETATM 39 Ru 2.70370 1.53388 13.92722 Ru1 1 1
0.00000
HETATM 40 Ru 6.75960 3.87584 13.92742 Ru1 1 1
0.00000
HETATM 41 Ru 4.05571 3.87548 13.92716 Ru1 1 1
0.00000

```
HETATM    42 Ru                 1.35176   3.87588  13.92751   Ru1  1 1
0.00000
HETATM    43 H                  2.70389   3.12218  15.08777    H1  1 1
0.00000
UNIT ENERGY   kcal
ENERGY        -8838.875752790698
END

XTLGRF 200
DESCRP Ruadshcpscand4
RUTYPE SINGLE POINT
#refdata /rf3/training/Ru_ads/hcp_scan/d4
REMARK Created by geo_energy_extract.py EO = -379.33396287 eV; -
8821.720066744187 kcal/mol
CRYSTX     8.11167    4.68327   28.85554   90.00000   90.00000   90.00000
HETATM     1 Ru                 0.00001   1.53078   1.16765   Ru1  1 1
0.00000
HETATM     2 Ru                 5.40780   1.53063   1.16776   Ru1  1 1
0.00000
HETATM     3 Ru                 2.70377   1.53065   1.16778   Ru1  1 1
0.00000
HETATM     4 Ru                 6.75965   3.87238   1.16767   Ru1  1 1
0.00000
HETATM     5 Ru                 4.05585   3.87230   1.16770   Ru1  1 1
0.00000
HETATM     6 Ru                 1.35193   3.87240   1.16769   Ru1  1 1
0.00000
HETATM     7 Ru                 1.35188   0.74928   3.22561   Ru1  1 1
0.00000
HETATM     8 Ru                 6.75970   0.74925   3.22561   Ru1  1 1
0.00000
HETATM     9 Ru                 4.05578   0.74927   3.22565   Ru1  1 1
0.00000
HETATM    10 Ru                 8.11158   3.09079   3.22563   Ru1  1 1
0.00000
HETATM    11 Ru                 5.40774   3.09088   3.22561   Ru1  1 1
0.00000
HETATM    12 Ru                 2.70386   3.09086   3.22562   Ru1  1 1
0.00000
HETATM    13 Ru                 8.11157   1.53090   5.37021   Ru1  1 1
0.00000
HETATM    14 Ru                 5.40770   1.53100   5.37022   Ru1  1 1
0.00000
HETATM    15 Ru                 2.70374   1.53096   5.37025   Ru1  1 1
0.00000
HETATM    16 Ru                 6.75960   3.87259   5.37017   Ru1  1 1
0.00000
HETATM    17 Ru                 4.05577   3.87276   5.37033   Ru1  1 1
0.00000
HETATM    18 Ru                 1.35188   3.87253   5.37021   Ru1  1 1
0.00000
HETATM    19 Ru                 1.35179   0.75177   7.52423   Ru1  1 1
0.00000
HETATM    20 Ru                 6.75945   0.75172   7.52425   Ru1  1 1
0.00000
HETATM    21 Ru                 4.05563   0.75194   7.52422   Ru1  1 1
0.00000
```

```
HETATM    22 Ru                    8.11145   3.09335   7.52406   Ru1  1 1
0.00000
HETATM    23 Ru                    5.40761   3.09354   7.52428   Ru1  1 1
0.00000
HETATM    24 Ru                    2.70362   3.09353   7.52431   Ru1  1 1
0.00000
HETATM    25 Ru                    8.11133   1.53310   9.67959   Ru1  1 1
0.00000
HETATM    26 Ru                    5.40756   1.53303   9.67970   Ru1  1 1
0.00000
HETATM    27 Ru                    2.70349   1.53295   9.67962   Ru1  1 1
0.00000
HETATM    28 Ru                    6.75949   3.87462   9.67967   Ru1  1 1
0.00000
HETATM    29 Ru                    4.05549   3.87464   9.67956   Ru1  1 1
0.00000
HETATM    30 Ru                    1.35147   3.87460   9.67959   Ru1  1 1
0.00000
HETATM    31 Ru                    1.35144   0.75297  11.80872   Ru1  1 1
0.00000
HETATM    32 Ru                    6.75937   0.75299  11.80875   Ru1  1 1
0.00000
HETATM    33 Ru                    4.05542   0.75288  11.80858   Ru1  1 1
0.00000
HETATM    34 Ru                    8.11131   3.09448  11.80877   Ru1  1 1
0.00000
HETATM    35 Ru                    5.40736   3.09454  11.80863   Ru1  1 1
0.00000
HETATM    36 Ru                    2.70345   3.09458  11.80861   Ru1  1 1
0.00000
HETATM    37 Ru                    8.11140   1.53440  13.92754   Ru1  1 1
0.00000
HETATM    38 Ru                    5.40759   1.53396  13.92717   Ru1  1 1
0.00000
HETATM    39 Ru                    2.70370   1.53388  13.92722   Ru1  1 1
0.00000
HETATM    40 Ru                    6.75960   3.87584  13.92742   Ru1  1 1
0.00000
HETATM    41 Ru                    4.05571   3.87548  13.92716   Ru1  1 1
0.00000
HETATM    42 Ru                    1.35176   3.87588  13.92751   Ru1  1 1
0.00000
HETATM    43 H                     2.70389   3.12218  15.66488    H1  1 1
0.00000
UNIT ENERGY   kcal
ENERGY        -8821.720066744187
END

XTLGRF 200
DESCRP Ruadshcpscand5
RUTYPE SINGLE POINT
#refdata /rf3/training/Ru_ads/hcp_scan/d5
REMARK Created by geo_energy_extract.py EO = -378.22494628 eV; -
8795.928983255813 kcal/mol
CRYSTX     8.11167    4.68327   28.85554   90.00000   90.00000   90.00000
HETATM     1 Ru                    0.00001   1.53078   1.16765   Ru1  1 1
0.00000
```

```
HETATM    2 Ru                     5.40780   1.53063   1.16776   Ru1  1 1
0.00000
HETATM    3 Ru                     2.70377   1.53065   1.16778   Ru1  1 1
0.00000
HETATM    4 Ru                     6.75965   3.87238   1.16767   Ru1  1 1
0.00000
HETATM    5 Ru                     4.05585   3.87230   1.16770   Ru1  1 1
0.00000
HETATM    6 Ru                     1.35193   3.87240   1.16769   Ru1  1 1
0.00000
HETATM    7 Ru                     1.35188   0.74928   3.22561   Ru1  1 1
0.00000
HETATM    8 Ru                     6.75970   0.74925   3.22561   Ru1  1 1
0.00000
HETATM    9 Ru                     4.05578   0.74927   3.22565   Ru1  1 1
0.00000
HETATM   10 Ru                     8.11158   3.09079   3.22563   Ru1  1 1
0.00000
HETATM   11 Ru                     5.40774   3.09088   3.22561   Ru1  1 1
0.00000
HETATM   12 Ru                     2.70386   3.09086   3.22562   Ru1  1 1
0.00000
HETATM   13 Ru                     8.11157   1.53090   5.37021   Ru1  1 1
0.00000
HETATM   14 Ru                     5.40770   1.53100   5.37022   Ru1  1 1
0.00000
HETATM   15 Ru                     2.70374   1.53096   5.37025   Ru1  1 1
0.00000
HETATM   16 Ru                     6.75960   3.87259   5.37017   Ru1  1 1
0.00000
HETATM   17 Ru                     4.05577   3.87276   5.37033   Ru1  1 1
0.00000
HETATM   18 Ru                     1.35188   3.87253   5.37021   Ru1  1 1
0.00000
HETATM   19 Ru                     1.35179   0.75177   7.52423   Ru1  1 1
0.00000
HETATM   20 Ru                     6.75945   0.75172   7.52425   Ru1  1 1
0.00000
HETATM   21 Ru                     4.05563   0.75194   7.52422   Ru1  1 1
0.00000
HETATM   22 Ru                     8.11145   3.09335   7.52406   Ru1  1 1
0.00000
HETATM   23 Ru                     5.40761   3.09354   7.52428   Ru1  1 1
0.00000
HETATM   24 Ru                     2.70362   3.09353   7.52431   Ru1  1 1
0.00000
HETATM   25 Ru                     8.11133   1.53310   9.67959   Ru1  1 1
0.00000
HETATM   26 Ru                     5.40756   1.53303   9.67970   Ru1  1 1
0.00000
HETATM   27 Ru                     2.70349   1.53295   9.67962   Ru1  1 1
0.00000
HETATM   28 Ru                     6.75949   3.87462   9.67967   Ru1  1 1
0.00000
HETATM   29 Ru                     4.05549   3.87464   9.67956   Ru1  1 1
0.00000
HETATM   30 Ru                     1.35147   3.87460   9.67959   Ru1  1 1
0.00000
```

```
HETATM    31 Ru                  1.35144   0.75297  11.80872   Ru1  1 1
0.00000
HETATM    32 Ru                  6.75937   0.75299  11.80875   Ru1  1 1
0.00000
HETATM    33 Ru                  4.05542   0.75288  11.80858   Ru1  1 1
0.00000
HETATM    34 Ru                  8.11131   3.09448  11.80877   Ru1  1 1
0.00000
HETATM    35 Ru                  5.40736   3.09454  11.80863   Ru1  1 1
0.00000
HETATM    36 Ru                  2.70345   3.09458  11.80861   Ru1  1 1
0.00000
HETATM    37 Ru                  8.11140   1.53440  13.92754   Ru1  1 1
0.00000
HETATM    38 Ru                  5.40759   1.53396  13.92717   Ru1  1 1
0.00000
HETATM    39 Ru                  2.70370   1.53388  13.92722   Ru1  1 1
0.00000
HETATM    40 Ru                  6.75960   3.87584  13.92742   Ru1  1 1
0.00000
HETATM    41 Ru                  4.05571   3.87548  13.92716   Ru1  1 1
0.00000
HETATM    42 Ru                  1.35176   3.87588  13.92751   Ru1  1 1
0.00000
HETATM    43 H                   2.70389   3.12218  16.24199    H1  1 1
0.00000
UNIT ENERGY   kcal
ENERGY        -8795.928983255813
END

XTLGRF 200
DESCRP RuHbulk0Hbulk
RUTYPE NORMAL RUN
#refdata /rf3/training/Ru_H_bulk/0H_bulk
REMARK Created by geo_energy_extract.py EO = -148.03710103 eV; -
3442.7232797674424 kcal/mol
CRYSTX     5.40782    9.36655    4.28168   90.00000   90.00000   90.00000
HETATM     1 Ru                  0.00000   1.56114   1.07042   Ru1  1 1
0.00000
HETATM     2 Ru                  0.00000   6.24442   1.07042   Ru1  1 1
0.00000
HETATM     3 Ru                  2.70391   1.56114   1.07042   Ru1  1 1
0.00000
HETATM     4 Ru                  2.70391   6.24442   1.07042   Ru1  1 1
0.00000
HETATM     5 Ru                  1.35195   3.90278   1.07041   Ru1  1 1
0.00000
HETATM     6 Ru                  1.35195   8.58605   1.07041   Ru1  1 1
0.00000
HETATM     7 Ru                  4.05586   3.90278   1.07042   Ru1  1 1
0.00000
HETATM     8 Ru                  4.05586   8.58605   1.07042   Ru1  1 1
0.00000
HETATM     9 Ru                  1.35196   0.78050   3.21127   Ru1  1 1
0.00000
HETATM    10 Ru                  1.35196   5.46377   3.21127   Ru1  1 1
0.00000
```

```
HETATM    11 Ru                  4.05587   0.78049   3.21126   Ru1  1 1
0.00000
HETATM    12 Ru                  4.05587   5.46377   3.21126   Ru1  1 1
0.00000
HETATM    13 Ru                  0.00000   3.12213   3.21126   Ru1  1 1
0.00000
HETATM    14 Ru                  0.00000   7.80541   3.21126   Ru1  1 1
0.00000
HETATM    15 Ru                  2.70391   3.12213   3.21126   Ru1  1 1
0.00000
HETATM    16 Ru                  2.70391   7.80541   3.21126   Ru1  1 1
0.00000
UNIT ENERGY   kcal
ENERGY        -3442.7232797674424
END

XTLGRF 200
DESCRP RuHbulk1Hbulk
RUTYPE NORMAL RUN
#refdata /rf3/training/Ru_H_bulk/1H_bulk
REMARK Created by geo_energy_extract.py EO = -151.10522247 eV; -
3514.074941162791 kcal/mol
CRYSTX     5.40782    9.36655    4.28168   90.00000   90.00000   90.00000
HETATM     1 Ru                  0.00000   1.56113   1.07313   Ru1  1 1
0.00000
HETATM     2 Ru                  0.00000   6.25816   1.07556   Ru1  1 1
0.00000
HETATM     3 Ru                  2.70391   1.54792   1.07316   Ru1  1 1
0.00000
HETATM     4 Ru                  2.70391   6.28449   1.05194   Ru1  1 1
0.00000
HETATM     5 Ru                  1.32113   3.88190   1.05382   Ru1  1 1
0.00000
HETATM     6 Ru                  1.35211   8.58838   1.07134   Ru1  1 1
0.00000
HETATM     7 Ru                  4.08669   3.88190   1.05383   Ru1  1 1
0.00000
HETATM     8 Ru                  4.05571   8.58838   1.07134   Ru1  1 1
0.00000
HETATM     9 Ru                  1.35210   0.77818   3.21132   Ru1  1 1
0.00000
HETATM    10 Ru                  1.32081   5.48485   3.22968   Ru1  1 1
0.00000
HETATM    11 Ru                  4.05572   0.77818   3.21132   Ru1  1 1
0.00000
HETATM    12 Ru                  4.08701   5.48485   3.22968   Ru1  1 1
0.00000
HETATM    13 Ru                  0.00000   3.10842   3.20777   Ru1  1 1
0.00000
HETATM    14 Ru                  0.00000   7.80545   3.20962   Ru1  1 1
0.00000
HETATM    15 Ru                  2.70391   3.08168   3.23141   Ru1  1 1
0.00000
HETATM    16 Ru                  2.70391   7.81858   3.20977   Ru1  1 1
0.00000
HETATM    17 H                   2.70391   4.68322   2.14417    H1  1 1
0.00000
UNIT ENERGY   kcal
```

```
ENERGY        -3514.074941162791
END

XTLGRF 200
DESCRP RuHbulk2Hbulk
RUTYPE NORMAL RUN
#refdata /rf3/training/Ru_H_bulk/2H_bulk
REMARK Created by geo_energy_extract.py EO = -154.02754050 eV; -
3582.0358255813953 kcal/mol
CRYSTX     5.40782    9.36655    4.28168   90.00000   90.00000   90.00000
HETATM     1 Ru                  0.00000   1.55680   1.07077   Ru1  1 1
0.00000
HETATM     2 Ru                  0.00000   6.27077   1.07105   Ru1  1 1
0.00000
HETATM     3 Ru                  2.70391   1.53356   1.07086   Ru1  1 1
0.00000
HETATM     4 Ru                  2.70391   6.31490   1.07107   Ru1  1 1
0.00000
HETATM     5 Ru                  1.30047   3.86418   1.07113   Ru1  1 1
0.00000
HETATM     6 Ru                  1.35018   8.59092   1.07072   Ru1  1 1
0.00000
HETATM     7 Ru                  4.10735   3.86418   1.07113   Ru1  1 1
0.00000
HETATM     8 Ru                  4.05764   8.59092   1.07072   Ru1  1 1
0.00000
HETATM     9 Ru                  1.35017   0.77564   3.21156   Ru1  1 1
0.00000
HETATM    10 Ru                  1.30030   5.50249   3.21197   Ru1  1 1
0.00000
HETATM    11 Ru                  4.05765   0.77565   3.21156   Ru1  1 1
0.00000
HETATM    12 Ru                  4.10751   5.50249   3.21197   Ru1  1 1
0.00000
HETATM    13 Ru                  0.00000   3.09580   3.21189   Ru1  1 1
0.00000
HETATM    14 Ru                  0.00000   7.80976   3.21161   Ru1  1 1
0.00000
HETATM    15 Ru                  2.70391   3.05144   3.21190   Ru1  1 1
0.00000
HETATM    16 Ru                  2.70391   7.83297   3.21170   Ru1  1 1
0.00000
HETATM    17 H                   2.70391   4.68321   2.14475    H1  1 1
0.00000
HETATM    18 H                   2.70391   4.68325   0.00250    H1  1 1
0.00000
UNIT ENERGY   kcal
ENERGY        -3582.0358255813953
END

XTLGRF 200
DESCRP RuHbulk3Hbulk
RUTYPE NORMAL RUN
#refdata /rf3/training/Ru_H_bulk/3H_bulk
REMARK Created by geo_energy_extract.py EO = -157.01536857 eV; -
3651.5201993023256 kcal/mol
CRYSTX     5.40782    9.36655    4.28168   90.00000   90.00000   90.00000
```

```
HETATM     1 Ru                   0.00000   1.59066   1.05307   Ru1  1 1
0.00000
HETATM     2 Ru                   0.00000   6.25754   1.07333   Ru1  1 1
0.00000
HETATM     3 Ru                   2.70391   1.54703   1.07389   Ru1  1 1
0.00000
HETATM     4 Ru                   2.70391   6.30852   1.07341   Ru1  1 1
0.00000
HETATM     5 Ru                   1.30123   3.86898   1.07149   Ru1  1 1
0.00000
HETATM     6 Ru                   1.38041   8.57212   1.05349   Ru1  1 1
0.00000
HETATM     7 Ru                   4.10659   3.86898   1.07149   Ru1  1 1
0.00000
HETATM     8 Ru                   4.02741   8.57212   1.05349   Ru1  1 1
0.00000
HETATM     9 Ru                   1.38038   0.79440   3.22870   Ru1  1 1
0.00000
HETATM    10 Ru                   1.30099   5.49774   3.21158   Ru1  1 1
0.00000
HETATM    11 Ru                   4.02744   0.79440   3.22870   Ru1  1 1
0.00000
HETATM    12 Ru                   4.10683   5.49774   3.21158   Ru1  1 1
0.00000
HETATM    13 Ru                   0.00000   3.10907   3.20952   Ru1  1 1
0.00000
HETATM    14 Ru                   0.00000   7.77595   3.22925   Ru1  1 1
0.00000
HETATM    15 Ru                   2.70391   3.05770   3.20955   Ru1  1 1
0.00000
HETATM    16 Ru                   2.70391   7.81948   3.20856   Ru1  1 1
0.00000
HETATM    17 H                    2.70391   4.68326   2.14542    H1  1 1
0.00000
HETATM    18 H                    2.70391   4.68325   0.00234    H1  1 1
0.00000
HETATM    19 H                    0.00000  -0.00001   2.14086    H1  1 1
0.00000
UNIT ENERGY   kcal
ENERGY        -3651.5201993023256
END

XTLGRF 200
DESCRP RuHbulk4Hbulk
RUTYPE NORMAL RUN
#refdata /rf3/training/Ru_H_bulk/4H_bulk
REMARK Created by geo_energy_extract.py EO = -159.87394301 eV; -
3717.998674651163 kcal/mol
CRYSTX     5.40782    9.36655    4.28168   90.00000   90.00000   90.00000
HETATM     1 Ru                   0.00000   1.61854   1.07069   Ru1  1 1
0.00000
HETATM     2 Ru                   0.00000   6.24401   1.07111   Ru1  1 1
0.00000
HETATM     3 Ru                   2.70391   1.56045   1.07081   Ru1  1 1
0.00000
HETATM     4 Ru                   2.70391   6.30164   1.07116   Ru1  1 1
0.00000
```

```
HETATM     5 Ru                    1.30251   3.87367   1.07126   Ru1  1 1
0.00000
HETATM     6 Ru                    1.40133   8.55683   1.07059   Ru1  1 1
0.00000
HETATM     7 Ru                    4.10531   3.87367   1.07125   Ru1  1 1
0.00000
HETATM     8 Ru                    4.00649   8.55683   1.07059   Ru1  1 1
0.00000
HETATM     9 Ru                    1.40125   0.80952   3.21143   Ru1  1 1
0.00000
HETATM    10 Ru                    1.30273   5.49290   3.21209   Ru1  1 1
0.00000
HETATM    11 Ru                    4.00657   0.80952   3.21143   Ru1  1 1
0.00000
HETATM    12 Ru                    4.10509   5.49290   3.21210   Ru1  1 1
0.00000
HETATM    13 Ru                    0.00000   3.12282   3.21196   Ru1  1 1
0.00000
HETATM    14 Ru                    0.00000   7.74836   3.21153   Ru1  1 1
0.00000
HETATM    15 Ru                    2.70391   3.06490   3.21200   Ru1  1 1
0.00000
HETATM    16 Ru                    2.70391   7.80580   3.21165   Ru1  1 1
0.00000
HETATM    17 H                     2.70391   4.68330   2.14431    H1  1 1
0.00000
HETATM    18 H                     2.70391   4.68327   0.00285    H1  1 1
0.00000
HETATM    19 H                     0.00000  -0.00003   2.14083    H1  1 1
0.00000
HETATM    20 H                     0.00000   0.00000   0.00009    H1  1 1
0.00000
UNIT ENERGY   kcal
ENERGY        -3717.998674651163
END

XTLGRF 200
DESCRP tRuHbulkocta
RUTYPE NORMAL RUN
#refdata /rf3/training/Ru_H_bulk/octa
REMARK Created by geo_energy_extract.py EO = -151.10522247 eV; -
3514.074941162791 kcal/mol
CRYSTX     5.40782    9.36655    4.28168   90.00000   90.00000   90.00000
HETATM     1 Ru                    0.00000   1.56113   1.07313   Ru1  1 1
0.00000
HETATM     2 Ru                    0.00000   6.25816   1.07556   Ru1  1 1
0.00000
HETATM     3 Ru                    2.70391   1.54792   1.07316   Ru1  1 1
0.00000
HETATM     4 Ru                    2.70391   6.28449   1.05194   Ru1  1 1
0.00000
HETATM     5 Ru                    1.32113   3.88190   1.05382   Ru1  1 1
0.00000
HETATM     6 Ru                    1.35211   8.58838   1.07134   Ru1  1 1
0.00000
HETATM     7 Ru                    4.08669   3.88190   1.05383   Ru1  1 1
0.00000
```

```
HETATM     8 Ru                 4.05571   8.58838   1.07134   Ru1  1 1
0.00000
HETATM     9 Ru                 1.35210   0.77818   3.21132   Ru1  1 1
0.00000
HETATM    10 Ru                 1.32081   5.48485   3.22968   Ru1  1 1
0.00000
HETATM    11 Ru                 4.05572   0.77818   3.21132   Ru1  1 1
0.00000
HETATM    12 Ru                 4.08701   5.48485   3.22968   Ru1  1 1
0.00000
HETATM    13 Ru                 0.00000   3.10842   3.20777   Ru1  1 1
0.00000
HETATM    14 Ru                 0.00000   7.80545   3.20962   Ru1  1 1
0.00000
HETATM    15 Ru                 2.70391   3.08168   3.23141   Ru1  1 1
0.00000
HETATM    16 Ru                 2.70391   7.81858   3.20977   Ru1  1 1
0.00000
HETATM    17 H                  2.70391   4.68322   2.14417    H1  1 1
0.00000
UNIT ENERGY   kcal
ENERGY        -3514.074941162791
END

XTLGRF 200
DESCRP tRuHbulktetra
RUTYPE NORMAL RUN
#refdata /rf3/training/Ru_H_bulk/tetra
REMARK Created by geo_energy_extract.py EO = -150.56388705 eV; -
3501.4857453488376 kcal/mol
CRYSTX     5.40782    9.36655    4.28168   90.00000   90.00000   90.00000
HETATM     1 Ru                -0.00833   1.55797   1.08807   Ru1  1 1
0.00000
HETATM     2 Ru                -0.07153   6.29561   1.07080   Ru1  1 1
0.00000
HETATM     3 Ru                 2.71224   1.55797   1.08807   Ru1  1 1
0.00000
HETATM     4 Ru                 2.77544   6.29561   1.07080   Ru1  1 1
0.00000
HETATM     5 Ru                 1.35196   3.79639   1.06417   Ru1  1 1
0.00000
HETATM     6 Ru                 1.35196   8.59218   1.08914   Ru1  1 1
0.00000
HETATM     7 Ru                 4.05586   3.90179   1.09332   Ru1  1 1
0.00000
HETATM     8 Ru                 4.05587   8.60092   1.09088   Ru1  1 1
0.00000
HETATM     9 Ru                 1.35196   0.78213   3.22696   Ru1  1 1
0.00000
HETATM    10 Ru                 1.35195   5.46445   3.33033   Ru1  1 1
0.00000
HETATM    11 Ru                 4.05586   0.78051   3.23203   Ru1  1 1
0.00000
HETATM    12 Ru                 4.05587   5.46363   3.22530   Ru1  1 1
0.00000
HETATM    13 Ru                -0.01049   3.10944   3.22522   Ru1  1 1
0.00000
```

```
HETATM    14 Ru                    -0.00382   7.82265   3.22597   Ru1  1 1
0.00000
HETATM    15 Ru                     2.71440   3.10944   3.22522   Ru1  1 1
0.00000
HETATM    16 Ru                     2.70773   7.82265   3.22597   Ru1  1 1
0.00000
HETATM    17 H                      1.35196   5.44289   1.60793    H1  1 1
0.00000
UNIT ENERGY   kcal
ENERGY        -3501.4857453488376
END

BIOGRF 200
DESCRP H2mol
RUTYPE NORMAL RUN
#refdata /ADF/first_guess/first_guess_5
REMARK Created by geo_energy_extract.py EO = -6.76076211 eV; -
157.2270258139535 kcal/mol
HETATM     1 H                      3.75000   3.75000   3.37448    H1  1 1
0.00000
HETATM     2 H                      3.75000   3.75000   4.12552    H1  1 1
0.00000
END

BIOGRF 200
DESCRP Ru2H3
RUTYPE NORMAL RUN
#refdata C:/Surfdrive/Documents/ReaxFF/Ru2H3/Ru2H3.t21
REMARK Created by ADFinput
HETATM     1 Ru                     6.95368   6.21330   0.00000  Ru1   1 1
0.00000
HETATM     2 Ru                     9.08603   6.33087   0.00000  Ru1   1 1
0.00000
HETATM     3 H                      6.30740   6.00149   1.47541   H1   1 1
0.00000
HETATM     4 H                      6.30740   6.00149  -1.47541   H1   1 1
0.00000
HETATM     5 H                      6.70924   4.65613   0.00000   H1   1 1
0.00000
END

BIOGRF 200
DESCRP Ru2H6
RUTYPE NORMAL RUN
#refdata C:/Surfdrive/Documents/ReaxFF/Ru2H6/Ru2H6.t21
REMARK Created by ADFinput
HETATM     1 Ru                     5.25908   5.32187   5.35374  Ru1   1 1
0.00000
HETATM     2 Ru                     7.10783   5.75560   6.50033  Ru1   1 1
0.00000
HETATM     3 H                      3.98238   5.53546   6.28876   H1   1 1
0.00000
HETATM     4 H                      5.15841   6.30538   4.10004   H1   1 1
0.00000
HETATM     5 H                      5.28344   3.81180   4.83530   H1   1 1
0.00000
HETATM     6 H                      7.08293   7.26658   7.01569   H1   1 1
0.00000
```

```
HETATM     7 H                 7.21112   4.77352   7.75522   H1   1 1
0.00000
HETATM     8 H                 8.38347   5.54450   5.56347   H1   1 1
0.00000
END

BIOGRF 200
DESCRP RuH_length-1
RUTYPE NORMAL RUN
BOND RESTRAINT 1 2 1.4 2500.0 1.0
#refdata C:/Surfdrive/Documents/ReaxFF/Ru_H_length/RuH_length.t21@#lt1
REMARK Created by ADFinput
HETATM     1 Ru                2.06794   2.43138   2.70557  Ru1   1 1
0.00000
HETATM     2 H                 2.07028   3.41547   1.70980   H1   1 1
0.00000
END

BIOGRF 200
DESCRP RuH_length-2
RUTYPE NORMAL RUN
BOND RESTRAINT 1 2 1.5 2500.0 1.0
#refdata C:/Surfdrive/Documents/ReaxFF/Ru_H_length/RuH_length.t21@#lt2
REMARK Created by ADFinput
HETATM     1 Ru                2.06786   2.39623   2.74114  Ru1   1 1
0.00000
HETATM     2 H                 2.07036   3.45061   1.67424   H1   1 1
0.00000
END

BIOGRF 200
DESCRP RuH_length-3
RUTYPE NORMAL RUN
BOND RESTRAINT 1 2 1.6 2500.0 1.0
#refdata C:/Surfdrive/Documents/ReaxFF/Ru_H_length/RuH_length.t21@#lt3
REMARK Created by ADFinput
HETATM     1 Ru                2.06777   2.36109   2.77670  Ru1   1 1
0.00000
HETATM     2 H                 2.07045   3.48576   1.63867   H1   1 1
0.00000
END

BIOGRF 200
DESCRP RuH_length-4
RUTYPE NORMAL RUN
BOND RESTRAINT 1 2 1.7 2500.0 1.0
#refdata C:/Surfdrive/Documents/ReaxFF/Ru_H_length/RuH_length.t21@#lt4
REMARK Created by ADFinput
HETATM     1 Ru                2.06769   2.32594   2.81226  Ru1   1 1
0.00000
HETATM     2 H                 2.07053   3.52090   1.60311   H1   1 1
0.00000
END

BIOGRF 200
DESCRP RuH_length-5
RUTYPE NORMAL RUN
BOND RESTRAINT 1 2 1.8 2500.0 1.0
```

```
#refdata C:/Surfdrive/Documents/ReaxFF/Ru_H_length/RuH_length.t21@#lt5
REMARK Created by ADFinput
HETATM     1 Ru                  2.06761   2.29080   2.84783  Ru1   1 1
0.00000
HETATM     2 H                   2.07061   3.55605   1.56755   H1   1 1
0.00000
END

BIOGRF 200
DESCRP RuH_length-6
RUTYPE NORMAL RUN
BOND RESTRAINT 1 2 1.9 2500.0 1.0
#refdata C:/Surfdrive/Documents/ReaxFF/Ru_H_length/RuH_length.t21@#lt6
REMARK Created by ADFinput
HETATM     1 Ru                  2.06752   2.25565   2.88339  Ru1   1 1
0.00000
HETATM     2 H                   2.07070   3.59120   1.53198   H1   1 1
0.00000
END

BIOGRF 200
DESCRP RuH_length-7
RUTYPE NORMAL RUN
BOND RESTRAINT 1 2 2.0 2500.0 1.0
#refdata C:/Surfdrive/Documents/ReaxFF/Ru_H_length/RuH_length.t21@#lt7
REMARK Created by ADFinput
HETATM     1 Ru                  2.06744   2.22050   2.91895  Ru1   1 1
0.00000
HETATM     2 H                   2.07078   3.62634   1.49642   H1   1 1
0.00000
END

BIOGRF 200
DESCRP RuH_length-8
RUTYPE NORMAL RUN
BOND RESTRAINT 1 2 2.1 2500.0 1.0
#refdata C:/Surfdrive/Documents/ReaxFF/Ru_H_length/RuH_length.t21@#lt8
REMARK Created by ADFinput
HETATM     1 Ru                  2.06736   2.18536   2.95452  Ru1   1 1
0.00000
HETATM     2 H                   2.07086   3.66149   1.46086   H1   1 1
0.00000
END

BIOGRF 200
DESCRP RuH_length-9
RUTYPE NORMAL RUN
BOND RESTRAINT 1 2 2.2 2500.0 1.0
#refdata C:/Surfdrive/Documents/ReaxFF/Ru_H_length/RuH_length.t21@#lt9
REMARK Created by ADFinput
HETATM     1 Ru                  2.06727   2.15021   2.99008  Ru1   1 1
0.00000
HETATM     2 H                   2.07095   3.69663   1.42529   H1   1 1
0.00000
END

BIOGRF 200
DESCRP RuH_length-10
```

```
RUTYPE NORMAL RUN
BOND RESTRAINT 1 2 2.3 2500.0 1.0
#refdata C:/Surfdrive/Documents/ReaxFF/Ru_H_length/RuH_length.t21@#lt10
REMARK Created by ADFinput
HETATM     1 Ru                 2.06719   2.11507   3.02564  Ru1   1 1
0.00000
HETATM     2 H                  2.07103   3.73178   1.38973   H1   1 1
0.00000
END

BIOGRF 200
DESCRP RuH2_angle-1
RUTYPE NORMAL RUN
ANGLE RESTRAINT 3 1 2 75 2500.0 1.0
#refdata C:/Surfdrive/Documents/ReaxFF/RuH2_angle/RuH2_angle.t21@#lt1
REMARK Created by ADFinput
HETATM     1 Ru                 1.78399   3.67311   3.18023  Ru1   1 1
0.00000
HETATM     2 H                  3.10713   2.83060   3.05106   H1   1 1
0.00000
HETATM     3 H                  1.31197   2.23887   2.73849   H1   1 1
0.00000
END

BIOGRF 200
DESCRP RuH2_angle-2
RUTYPE NORMAL RUN
ANGLE RESTRAINT 3 1 2 80 2500.0 1.0
#refdata C:/Surfdrive/Documents/ReaxFF/RuH2_angle/RuH2_angle.t21@#lt2
REMARK Created by ADFinput
HETATM     1 Ru                 1.79332   3.64812   3.17396  Ru1   1 1
0.00000
HETATM     2 H                  3.15412   2.86011   3.06318   H1   1 1
0.00000
HETATM     3 H                  1.25566   2.23435   2.73263   H1   1 1
0.00000
END

BIOGRF 200
DESCRP RuH2_angle-3
RUTYPE NORMAL RUN
ANGLE RESTRAINT 3 1 2 85 2500.0 1.0
#refdata C:/Surfdrive/Documents/ReaxFF/RuH2_angle/RuH2_angle.t21@#lt3
REMARK Created by ADFinput
HETATM     1 Ru                 1.80372   3.62070   3.16710  Ru1   1 1
0.00000
HETATM     2 H                  3.19746   2.88990   3.07509   H1   1 1
0.00000
HETATM     3 H                  1.20191   2.23198   2.72759   H1   1 1
0.00000
END

BIOGRF 200
DESCRP RuH2_angle-4
RUTYPE NORMAL RUN
ANGLE RESTRAINT 3 1 2 90 2500.0 1.0
#refdata C:/Surfdrive/Documents/ReaxFF/RuH2_angle/RuH2_angle.t21@#lt4
REMARK Created by ADFinput
```

```
HETATM     1 Ru                 1.81520   3.59178   3.15990  Ru1   1 1
0.00000
HETATM     2 H                  3.23781   2.91998   3.08683   H1   1 1
0.00000
HETATM     3 H                  1.15008   2.23083   2.72304   H1   1 1
0.00000
END

BIOGRF 200
DESCRP RuH2_angle-5
RUTYPE NORMAL RUN
ANGLE RESTRAINT 3 1 2 95 2500.0 1.0
#refdata C:/Surfdrive/Documents/ReaxFF/RuH2_angle/RuH2_angle.t21@#lt5
REMARK Created by ADFinput
HETATM     1 Ru                 1.82570   3.56182   3.15233  Ru1   1 1
0.00000
HETATM     2 H                  3.27796   2.94959   3.09843   H1   1 1
0.00000
HETATM     3 H                  1.09944   2.23117   2.71902   H1   1 1
0.00000
END

BIOGRF 200
DESCRP RuH2_angle-6
RUTYPE NORMAL RUN
ANGLE RESTRAINT 3 1 2 100 2500.0 1.0
#refdata C:/Surfdrive/Documents/ReaxFF/RuH2_angle/RuH2_angle.t21@#lt6
REMARK Created by ADFinput
HETATM     1 Ru                 1.83708   3.53091   3.14457  Ru1   1 1
0.00000
HETATM     2 H                  3.31586   2.97938   3.10989   H1   1 1
0.00000
HETATM     3 H                  1.05016   2.23229   2.71532   H1   1 1
0.00000
END

BIOGRF 200
DESCRP RuH2_angle-7
RUTYPE NORMAL RUN
ANGLE RESTRAINT 3 1 2 105 2500.0 1.0
#refdata C:/Surfdrive/Documents/ReaxFF/RuH2_angle/RuH2_angle.t21@#lt7
REMARK Created by ADFinput
HETATM     1 Ru                 1.84946   3.49837   3.13643  Ru1   1 1
0.00000
HETATM     2 H                  3.35012   3.00908   3.12103   H1   1 1
0.00000
HETATM     3 H                  1.00352   2.23513   2.71232   H1   1 1
0.00000
END

BIOGRF 200
DESCRP RuH2_angle-8
RUTYPE NORMAL RUN
ANGLE RESTRAINT 3 1 2 110 2500.0 1.0
#refdata C:/Surfdrive/Documents/ReaxFF/RuH2_angle/RuH2_angle.t21@#lt8
REMARK Created by ADFinput
HETATM     1 Ru                 1.86194   3.46515   3.12810  Ru1   1 1
0.00000
```

```
HETATM     2 H                   3.38317   3.03867   3.13205   H1   1 1
0.00000
HETATM     3 H                   0.95799   2.23876   2.70963   H1   1 1
0.00000
END

BIOGRF 200
DESCRP RuH2_angle-9
RUTYPE NORMAL RUN
ANGLE RESTRAINT 3 1 2 115 2500.0 1.0
#refdata C:/Surfdrive/Documents/ReaxFF/RuH2_angle/RuH2_angle.t21@#lt9
REMARK Created by ADFinput
HETATM     1 Ru                  1.87490   3.43100   3.11955  Ru1   1 1
0.00000
HETATM     2 H                   3.41403   3.06828   3.14289   H1   1 1
0.00000
HETATM     3 H                   0.91417   2.24330   2.70734   H1   1 1
0.00000
END

BIOGRF 200
DESCRP RuH2_angle-10
RUTYPE NORMAL RUN
ANGLE RESTRAINT 3 1 2 120 2500.0 1.0
#refdata C:/Surfdrive/Documents/ReaxFF/RuH2_angle/RuH2_angle.t21@#lt10
REMARK Created by ADFinput
HETATM     1 Ru                  1.90083   3.39995   3.11293  Ru1   1 1
0.00000
HETATM     2 H                   3.44848   3.06263   3.14410   H1   1 1
0.00000
HETATM     3 H                   0.85378   2.28000   2.71274   H1   1 1
0.00000
END

BIOGRF 200
DESCRP RuH4_length-1
RUTYPE NORMAL RUN
BOND RESTRAINT 1 3 1.5 2500.0 1.0
#refdata C:/Surfdrive/Documents/ReaxFF/RuH4_length/RuH4_length.t21@#lt1
REMARK Created by ADFinput
HETATM     1 Ru                  0.17456  -0.06264  -0.11856  Ru1   1 1
0.00000
HETATM     2 H                   1.45933   0.35968   0.68607   H1   1 1
0.00000
HETATM     3 H                  -1.04723   0.41827   0.60666   H1   1 1
0.00000
HETATM     4 H                   0.21538   0.57705  -1.55732   H1   1 1
0.00000
HETATM     5 H                   0.12675  -1.63126  -0.23670   H1   1 1
0.00000
END

BIOGRF 200
DESCRP RuH4_length-2
RUTYPE NORMAL RUN
BOND RESTRAINT 1 3 1.6666666666666667 2500.0 1.0
#refdata C:/Surfdrive/Documents/ReaxFF/RuH4_length/RuH4_length.t21@#lt2
REMARK Created by ADFinput
```

```
HETATM     1 Ru                   0.19854  -0.07327  -0.13046  Ru1   1 1
0.00000
HETATM     2 H                    1.48771   0.35016   0.66551   H1   1 1
0.00000
HETATM     3 H                   -1.15994   0.46362   0.67207   H1   1 1
0.00000
HETATM     4 H                    0.24693   0.56133  -1.56935   H1   1 1
0.00000
HETATM     5 H                    0.15556  -1.64073  -0.25761   H1   1 1
0.00000
END

BIOGRF 200
DESCRP RuH4_length-3
RUTYPE NORMAL RUN
BOND RESTRAINT 1 3 1.8333333333333333 2500.0 1.0
#refdata C:/Surfdrive/Documents/ReaxFF/RuH4_length/RuH4_length.t21@#lt3
REMARK Created by ADFinput
HETATM     1 Ru                   0.22452  -0.08346  -0.14594  Ru1   1 1
0.00000
HETATM     2 H                    1.51476   0.33827   0.64787   H1   1 1
0.00000
HETATM     3 H                   -1.26976   0.50694   0.73705   H1   1 1
0.00000
HETATM     4 H                    0.27515   0.54958  -1.58473   H1   1 1
0.00000
HETATM     5 H                    0.18412  -1.65021  -0.27409   H1   1 1
0.00000
END

BIOGRF 200
DESCRP RuH4_length-4
RUTYPE NORMAL RUN
BOND RESTRAINT 1 3 2.0 2500.0 1.0
#refdata C:/Surfdrive/Documents/ReaxFF/RuH4_length/RuH4_length.t21@#lt4
REMARK Created by ADFinput
HETATM     1 Ru                   0.24764  -0.09340  -0.16006  Ru1   1 1
0.00000
HETATM     2 H                    1.54177   0.32326   0.62692   H1   1 1
0.00000
HETATM     3 H                   -1.38067   0.55527   0.80319   H1   1 1
0.00000
HETATM     4 H                    0.30606   0.53449  -1.59893   H1   1 1
0.00000
HETATM     5 H                    0.21399  -1.65851  -0.29096   H1   1 1
0.00000
END

BIOGRF 200
DESCRP RuH4_length-5
RUTYPE NORMAL RUN
BOND RESTRAINT 1 3 2.1666666666666667 2500.0 1.0
#refdata C:/Surfdrive/Documents/ReaxFF/RuH4_length/RuH4_length.t21@#lt5
REMARK Created by ADFinput
HETATM     1 Ru                   0.27750  -0.10051  -0.17714  Ru1   1 1
0.00000
HETATM     2 H                    1.57173   0.31225   0.61095   H1   1 1
0.00000
```

```
HETATM     3 H                  -1.49013   0.59147   0.86742   H1   1 1
0.00000
HETATM     4 H                   0.33473   0.52329  -1.61749   H1   1 1
0.00000
HETATM     5 H                   0.23497  -1.66538  -0.30358   H1   1 1
0.00000
END

BIOGRF 200
DESCRP RuH4_length-6
RUTYPE NORMAL RUN
BOND RESTRAINT 1 3 2.3333333333333333 2500.0 1.0
#refdata C:/Surfdrive/Documents/ReaxFF/RuH4_length/RuH4_length.t21@#lt6
REMARK Created by ADFinput
HETATM     1 Ru                  0.30172  -0.10716  -0.19176  Ru1   1 1
0.00000
HETATM     2 H                   1.57645   0.34365   0.60747   H1   1 1
0.00000
HETATM     3 H                  -1.63176   0.53828   0.94381   H1   1 1
0.00000
HETATM     4 H                   0.34248   0.55413  -1.61582   H1   1 1
0.00000
HETATM     5 H                   0.33990  -1.66779  -0.36354   H1   1 1
0.00000
END

BIOGRF 200
DESCRP RuH4_length-7
RUTYPE NORMAL RUN
BOND RESTRAINT 1 3 2.5 2500.0 1.0
#refdata C:/Surfdrive/Documents/ReaxFF/RuH4_length/RuH4_length.t21@#lt7
REMARK Created by ADFinput
HETATM     1 Ru                  0.32864  -0.11995  -0.20969  Ru1   1 1
0.00000
HETATM     2 H                   1.59994   0.33790   0.58786   H1   1 1
0.00000
HETATM     3 H                  -1.74054   0.57501   1.00912   H1   1 1
0.00000
HETATM     4 H                   0.37327   0.54877  -1.62799   H1   1 1
0.00000
HETATM     5 H                   0.36749  -1.68063  -0.37914   H1   1 1
0.00000
END

BIOGRF 200
DESCRP RuH4_length-8
RUTYPE NORMAL RUN
BOND RESTRAINT 1 3 2.6666666666666667 2500.0 1.0
#refdata C:/Surfdrive/Documents/ReaxFF/RuH4_length/RuH4_length.t21@#lt8
REMARK Created by ADFinput
HETATM     1 Ru                  0.35309  -0.12595  -0.22457  Ru1   1 1
0.00000
HETATM     2 H                   1.62906   0.32259   0.57169   H1   1 1
0.00000
HETATM     3 H                  -1.85362   0.61613   1.07576   H1   1 1
0.00000
HETATM     4 H                   0.40359   0.53368  -1.64775   H1   1 1
0.00000
```

```
HETATM     5 H                   0.39668  -1.68534  -0.39497   H1   1 1
0.00000
END

BIOGRF 200
DESCRP RuH4_length-9
RUTYPE NORMAL RUN
BOND RESTRAINT 1 3 2.8333333333333333 2500.0 1.0
#refdata C:/Surfdrive/Documents/ReaxFF/RuH4_length/RuH4_length.t21@#lt9
REMARK Created by ADFinput
HETATM     1 Ru                  0.37753  -0.13197  -0.23869  Ru1   1 1
0.00000
HETATM     2 H                   1.65896   0.30982   0.55258   H1   1 1
0.00000
HETATM     3 H                  -1.96838   0.65262   1.14295   H1   1 1
0.00000
HETATM     4 H                   0.43427   0.52119  -1.66495   H1   1 1
0.00000
HETATM     5 H                   0.42641  -1.69055  -0.41174   H1   1 1
0.00000
END

BIOGRF 200
DESCRP RuH4_length-10
RUTYPE NORMAL RUN
BOND RESTRAINT 1 3 3.0 2500.0 1.0
#refdata C:/Surfdrive/Documents/ReaxFF/RuH4_length/RuH4_length.t21@#lt10
REMARK Created by ADFinput
HETATM     1 Ru                  0.40187  -0.14773  -0.25280  Ru1   1 1
0.00000
HETATM     2 H                   1.68209   0.30184   0.53560   H1   1 1
0.00000
HETATM     3 H                  -2.07836   0.69906   1.20713   H1   1 1
0.00000
HETATM     4 H                   0.45779   0.51293  -1.67474   H1   1 1
0.00000
HETATM     5 H                   0.46539  -1.70500  -0.43503   H1   1 1
0.00000
END

BIOGRF 200
DESCRP H2
RUTYPE NORMAL RUN
#refdata C:/Surfdrive/Documents/ReaxFF/Train/H2/H2.t21
REMARK Created by ADFinput
HETATM     1 H                   0.00000   0.00000  -0.37527   H1   1 1
0.00000
HETATM     2 H                   0.00000   0.00000   0.37527   H1   1 1
0.00000
END


XTLGRF 200
DESCRP NEBocta2octa00
RUTYPE SINGLE POINT
#refdata /rf3/training/NEB/octa_2_octa/00
REMARK Created by geo_energy_extract.py EO = -151.14488189 eV; -
3514.997253255814 kcal/mol
```

```
CRYSTX      5.40778    5.40778    8.56336   90.00000   90.00000  120.00001
HETATM      1 Ru                  0.78055   4.05583   1.10534   Ru1  1 1
0.00000
HETATM      2 Ru                  0.78055   4.05583   5.35805   Ru1  1 1
0.00000
HETATM      3 Ru                  3.12340  -0.00211   1.09455   Ru1  1 1
0.00000
HETATM      4 Ru                  3.13997  -0.03080   5.34738   Ru1  1 1
0.00000
HETATM      5 Ru                  3.12340   2.70599   1.09455   Ru1  1 1
0.00000
HETATM      6 Ru                  3.13997   2.73469   5.34738   Ru1  1 1
0.00000
HETATM      7 Ru                  1.55978  -0.00228   3.22285   Ru1  1 1
0.00000
HETATM      8 Ru                  1.54362  -0.03025   7.53318   Ru1  1 1
0.00000
HETATM      9 Ru                  1.55978   2.70616   3.22285   Ru1  1 1
0.00000
HETATM     10 Ru                  1.54362   2.73415   7.53318   Ru1  1 1
0.00000
HETATM     11 Ru                  3.90274  -1.35194   3.21150   Ru1  1 1
0.00000
HETATM     12 Ru                  3.90273  -1.35194   7.52316   Ru1  1 1
0.00000
HETATM     13 Ru                  3.90535   1.35194   3.22285   Ru1  1 1
0.00000
HETATM     14 Ru                  3.93767   1.35194   7.53318   Ru1  1 1
0.00000
HETATM     15 Ru                  0.77811   1.35194   1.09455   Ru1  1 1
0.00000
HETATM     16 Ru                  0.74498   1.35195   5.34737   Ru1  1 1
0.00000
HETATM     17 H                   2.34164   1.35194   6.43625    H1  1 1
0.00000
UNIT ENERGY   kcal
ENERGY        -3514.997253255814
END

XTLGRF 200
DESCRP NEBocta2octa01
RUTYPE SINGLE POINT
#refdata /rf3/training/NEB/octa_2_octa/01
REMARK Created by geo_energy_extract.py EO = -150.83286319 eV; -
3507.7410044186054 kcal/mol
CRYSTX      5.40778    5.40778    8.56336   90.00000   90.00000  120.00001
HETATM      1 Ru                  0.78055   4.05583   1.06020   Ru1  1 1
0.00000
HETATM      2 Ru                  0.78055   4.05583   5.33957   Ru1  1 1
0.00000
HETATM      3 Ru                  3.12062   0.00270   1.06218   Ru1  1 1
0.00000
HETATM      4 Ru                  3.15920  -0.06411   5.31411   Ru1  1 1
0.00000
HETATM      5 Ru                  3.12063   2.70119   1.06218   Ru1  1 1
0.00000
HETATM      6 Ru                  3.15919   2.76800   5.31411   Ru1  1 1
0.00000
```

```
HETATM     7 Ru                  1.55774  -0.00582   3.19062   Ru1  1 1
0.00000
HETATM     8 Ru                  1.55312  -0.01382   7.49447   Ru1  1 1
0.00000
HETATM     9 Ru                  1.55774   2.70970   3.19061   Ru1  1 1
0.00000
HETATM    10 Ru                  1.55311   2.71771   7.49447   Ru1  1 1
0.00000
HETATM    11 Ru                  3.90274  -1.35194   3.16086   Ru1  1 1
0.00000
HETATM    12 Ru                  3.90274  -1.35194   7.51076   Ru1  1 1
0.00000
HETATM    13 Ru                  3.90944   1.35195   3.19062   Ru1  1 1
0.00000
HETATM    14 Ru                  3.91869   1.35194   7.49447   Ru1  1 1
0.00000
HETATM    15 Ru                  0.78366   1.35194   1.06218   Ru1  1 1
0.00000
HETATM    16 Ru                  0.70652   1.35195   5.31411   Ru1  1 1
0.00000
HETATM    17 H                   2.34164   1.35194   5.90420    H1  1 1
0.00000
UNIT ENERGY   kcal
ENERGY        -3507.7410044186054
END

XTLGRF 200
DESCRP NEBocta2octa02
RUTYPE SINGLE POINT
#refdata /rf3/training/NEB/octa_2_octa/02
REMARK Created by geo_energy_extract.py E0 = -150.47526710 eV; -
3499.42481627907 kcal/mol
CRYSTX     5.40778    5.40778    8.56336   90.00000   90.00000  120.00001
HETATM     1 Ru                  0.78055   4.05583   1.08807   Ru1  1 1
0.00000
HETATM     2 Ru                  0.78055   4.05583   5.36976   Ru1  1 1
0.00000
HETATM     3 Ru                  3.11992   0.00392   1.08807   Ru1  1 1
0.00000
HETATM     4 Ru                  3.17100  -0.08455   5.36978   Ru1  1 1
0.00000
HETATM     5 Ru                  3.11992   2.69996   1.08807   Ru1  1 1
0.00000
HETATM     6 Ru                  3.17099   2.78844   5.36978   Ru1  1 1
0.00000
HETATM     7 Ru                  1.55690  -0.00727   3.21996   Ru1  1 1
0.00000
HETATM     8 Ru                  1.55690  -0.00727   7.51955   Ru1  1 1
0.00000
HETATM     9 Ru                  1.55689   2.71116   3.21996   Ru1  1 1
0.00000
HETATM    10 Ru                  1.55690   2.71115   7.51955   Ru1  1 1
0.00000
HETATM    11 Ru                  3.90274  -1.35194   3.18993   Ru1  1 1
0.00000
HETATM    12 Ru                  3.90274  -1.35194   7.54959   Ru1  1 1
0.00000
```

```
HETATM    13 Ru                  3.91112   1.35194   3.21996   Ru1  1 1
0.00000
HETATM    14 Ru                  3.91111   1.35195   7.51955   Ru1  1 1
0.00000
HETATM    15 Ru                  0.78507   1.35194   1.08807   Ru1  1 1
0.00000
HETATM    16 Ru                  0.68292   1.35195   5.36978   Ru1  1 1
0.00000
HETATM    17 H                   2.34163   1.35195   5.36931    H1  1 1
0.00000
UNIT ENERGY   kcal
ENERGY        -3499.42481627907
END

XTLGRF 200
DESCRP NEBocta2octa03
RUTYPE SINGLE POINT
#refdata /rf3/training/NEB/octa_2_octa/03
REMARK Created by geo_energy_extract.py EO = -150.83736180 eV; -
3507.845623255814 kcal/mol
CRYSTX     5.40778    5.40778    8.56336   90.00000   90.00000  120.00001
HETATM     1 Ru                  0.78055   4.05583   1.11618   Ru1  1 1
0.00000
HETATM     2 Ru                  0.78055   4.05583   5.40028   Ru1  1 1
0.00000
HETATM     3 Ru                  3.12064   0.00267   1.11422   Ru1  1 1
0.00000
HETATM     4 Ru                  3.15901  -0.06379   5.42573   Ru1  1 1
0.00000
HETATM     5 Ru                  3.12065   2.70122   1.11422   Ru1  1 1
0.00000
HETATM     6 Ru                  3.15900   2.76768   5.42573   Ru1  1 1
0.00000
HETATM     7 Ru                  1.55306  -0.01392   3.24524   Ru1  1 1
0.00000
HETATM     8 Ru                  1.55775  -0.00580   7.54915   Ru1  1 1
0.00000
HETATM     9 Ru                  1.55306   2.71781   3.24524   Ru1  1 1
0.00000
HETATM    10 Ru                  1.55774   2.70969   7.54915   Ru1  1 1
0.00000
HETATM    11 Ru                  3.90274  -1.35194   3.22916   Ru1  1 1
0.00000
HETATM    12 Ru                  3.90274  -1.35194   7.57883   Ru1  1 1
0.00000
HETATM    13 Ru                  3.91880   1.35194   3.24524   Ru1  1 1
0.00000
HETATM    14 Ru                  3.90943   1.35194   7.54915   Ru1  1 1
0.00000
HETATM    15 Ru                  0.78363   1.35194   1.11422   Ru1  1 1
0.00000
HETATM    16 Ru                  0.70690   1.35195   5.42573   Ru1  1 1
0.00000
HETATM    17 H                   2.34164   1.35194   4.83057    H1  1 1
0.00000
UNIT ENERGY   kcal
ENERGY        -3507.845623255814
END
```

```
XTLGRF 200
DESCRP NEBocta2octa04
RUTYPE SINGLE POINT
#refdata /rf3/training/NEB/octa_2_octa/04
REMARK Created by geo_energy_extract.py EO = -151.14434720 eV; -
3514.9848186046515 kcal/mol
CRYSTX     5.40778    5.40778    8.56336   90.00000   90.00000  120.00001
HETATM     1 Ru                  0.78055   4.05583   1.07015   Ru1  1 1
0.00000
HETATM     2 Ru                  0.78055   4.05583   5.38094   Ru1  1 1
0.00000
HETATM     3 Ru                  3.12332  -0.00197   1.08102   Ru1  1 1
0.00000
HETATM     4 Ru                  3.13975  -0.03042   5.39185   Ru1  1 1
0.00000
HETATM     5 Ru                  3.12332   2.70585   1.08102   Ru1  1 1
0.00000
HETATM     6 Ru                  3.13975   2.73431   5.39185   Ru1  1 1
0.00000
HETATM     7 Ru                  1.54350  -0.03045   3.20564   Ru1  1 1
0.00000
HETATM     8 Ru                  1.55970  -0.00242   7.51627   Ru1  1 1
0.00000
HETATM     9 Ru                  1.54351   2.73434   3.20564   Ru1  1 1
0.00000
HETATM    10 Ru                  1.55970   2.70630   7.51627   Ru1  1 1
0.00000
HETATM    11 Ru                  3.90273  -1.35194   3.21621   Ru1  1 1
0.00000
HETATM    12 Ru                  3.90274  -1.35194   7.52648   Ru1  1 1
0.00000
HETATM    13 Ru                  3.93789   1.35195   3.20564   Ru1  1 1
0.00000
HETATM    14 Ru                  3.90551   1.35195   7.51627   Ru1  1 1
0.00000
HETATM    15 Ru                  0.77827   1.35194   1.08102   Ru1  1 1
0.00000
HETATM    16 Ru                  0.74541   1.35194   5.39185   Ru1  1 1
0.00000
HETATM    17 H                   2.34164   1.35194   4.29544    H1  1 1
0.00000
UNIT ENERGY   kcal
ENERGY        -3514.9848186046515
END

XTLGRF 200
DESCRP NEBocta2octaPREbegin
RUTYPE SINGLE POINT
#refdata /rf3/training/NEB/octa_2_octa/PRE_begin
REMARK Created by geo_energy_extract.py EO = -151.14488189 eV; -
3514.997253255814 kcal/mol
CRYSTX     5.40778    5.40778    8.56336   90.00000   90.00000  120.00001
HETATM     1 Ru                  0.78055   4.05583   1.10534   Ru1  1 1
0.00000
HETATM     2 Ru                  0.78055   4.05583   5.35805   Ru1  1 1
0.00000
```

```
HETATM     3 Ru                    3.12340  -0.00211   1.09455   Ru1  1 1
0.00000
HETATM     4 Ru                    3.13997  -0.03080   5.34738   Ru1  1 1
0.00000
HETATM     5 Ru                    3.12340   2.70599   1.09455   Ru1  1 1
0.00000
HETATM     6 Ru                    3.13997   2.73469   5.34738   Ru1  1 1
0.00000
HETATM     7 Ru                    1.55978  -0.00228   3.22285   Ru1  1 1
0.00000
HETATM     8 Ru                    1.54362  -0.03025   7.53318   Ru1  1 1
0.00000
HETATM     9 Ru                    1.55978   2.70616   3.22285   Ru1  1 1
0.00000
HETATM    10 Ru                    1.54362   2.73415   7.53318   Ru1  1 1
0.00000
HETATM    11 Ru                    3.90274  -1.35194   3.21150   Ru1  1 1
0.00000
HETATM    12 Ru                    3.90273  -1.35194   7.52316   Ru1  1 1
0.00000
HETATM    13 Ru                    3.90535   1.35194   3.22285   Ru1  1 1
0.00000
HETATM    14 Ru                    3.93767   1.35194   7.53318   Ru1  1 1
0.00000
HETATM    15 Ru                    0.77811   1.35194   1.09455   Ru1  1 1
0.00000
HETATM    16 Ru                    0.74498   1.35195   5.34737   Ru1  1 1
0.00000
HETATM    17 H                     2.34164   1.35194   6.43625    H1  1 1
0.00000
UNIT ENERGY   kcal
ENERGY        -3514.997253255814
END

XTLGRF 200
DESCRP NEBocta2octaPREend
RUTYPE SINGLE POINT
#refdata /rf3/training/NEB/octa_2_octa/PRE_end
REMARK Created by geo_energy_extract.py E0 = -151.14434720 eV; -
3514.9848186046515 kcal/mol
CRYSTX     5.40778    5.40778    8.56336   90.00000   90.00000  120.00001
HETATM     1 Ru                    0.78055   4.05583   1.07015   Ru1  1 1
0.00000
HETATM     2 Ru                    0.78055   4.05583   5.38094   Ru1  1 1
0.00000
HETATM     3 Ru                    3.12332  -0.00197   1.08102   Ru1  1 1
0.00000
HETATM     4 Ru                    3.13975  -0.03042   5.39185   Ru1  1 1
0.00000
HETATM     5 Ru                    3.12332   2.70585   1.08102   Ru1  1 1
0.00000
HETATM     6 Ru                    3.13975   2.73431   5.39185   Ru1  1 1
0.00000
HETATM     7 Ru                    1.54350  -0.03045   3.20564   Ru1  1 1
0.00000
HETATM     8 Ru                    1.55970  -0.00242   7.51627   Ru1  1 1
0.00000
```

```
HETATM     9 Ru                    1.54351   2.73434   3.20564   Ru1  1 1
0.00000
HETATM    10 Ru                    1.55970   2.70630   7.51627   Ru1  1 1
0.00000
HETATM    11 Ru                    3.90273  -1.35194   3.21621   Ru1  1 1
0.00000
HETATM    12 Ru                    3.90274  -1.35194   7.52648   Ru1  1 1
0.00000
HETATM    13 Ru                    3.93789   1.35195   3.20564   Ru1  1 1
0.00000
HETATM    14 Ru                    3.90551   1.35195   7.51627   Ru1  1 1
0.00000
HETATM    15 Ru                    0.77827   1.35194   1.08102   Ru1  1 1
0.00000
HETATM    16 Ru                    0.74541   1.35194   5.39185   Ru1  1 1
0.00000
HETATM    17 H                     2.34164   1.35194   4.29544    H1  1 1
0.00000
UNIT ENERGY   kcal
ENERGY        -3514.9848186046515
END

XTLGRF 200
DESCRP NEBfcc2octa00
RUTYPE SINGLE POINT
#refdata /rf3/training/NEB/fcc_2_octa/00
REMARK Created by geo_energy_extract.py EO = -254.75726579 eV; -
5924.587576511628 kcal/mol
CRYSTX     5.40778    5.40778   30.90350   90.00000   90.00000  120.00000
HETATM     1 Ru                    0.76926   1.35194   1.23124   Ru1  1 1
0.00000
HETATM     2 Ru                    0.78055   4.05582   1.22612   Ru1  1 1
0.00000
HETATM     3 Ru                    3.12783  -0.00976   1.23123   Ru1  1 1
0.00000
HETATM     4 Ru                    3.12786   2.71369   1.23126   Ru1  1 1
0.00000
HETATM     5 Ru                    1.56144   0.00062   3.27471   Ru1  1 1
0.00000
HETATM     6 Ru                    1.56150   2.70330   3.27488   Ru1  1 1
0.00000
HETATM     7 Ru                    3.90271  -1.35199   3.28083   Ru1  1 1
0.00000
HETATM     8 Ru                    3.90202   1.35202   3.27486   Ru1  1 1
0.00000
HETATM     9 Ru                    0.78402   1.35190   5.40060   Ru1  1 1
0.00000
HETATM    10 Ru                    0.78056   4.05586   5.39578   Ru1  1 1
0.00000
HETATM    11 Ru                    3.12041   0.00299   5.40057   Ru1  1 1
0.00000
HETATM    12 Ru                    3.12044   2.70087   5.40076   Ru1  1 1
0.00000
HETATM    13 Ru                    1.56136   0.00047   7.52859   Ru1  1 1
0.00000
HETATM    14 Ru                    1.56141   2.70334   7.52862   Ru1  1 1
0.00000
```

```
HETATM    15 Ru                  3.90274  -1.35192   7.52279   Ru1  1 1
0.00000
HETATM    16 Ru                  3.90210   1.35195   7.52862   Ru1  1 1
0.00000
HETATM    17 Ru                  0.78081   1.35191   9.65363   Ru1  1 1
0.00000
HETATM    18 Ru                  0.78056   4.05589   9.66658   Ru1  1 1
0.00000
HETATM    19 Ru                  3.12202   0.00020   9.65367   Ru1  1 1
0.00000
HETATM    20 Ru                  3.12203   2.70362   9.65348   Ru1  1 1
0.00000
HETATM    21 Ru                  1.56367   0.00448  11.78460   Ru1  1 1
0.00000
HETATM    22 Ru                  1.56374   2.69940  11.78442   Ru1  1 1
0.00000
HETATM    23 Ru                  3.90271  -1.35197  11.74918   Ru1  1 1
0.00000
HETATM    24 Ru                  3.89753   1.35201  11.78445   Ru1  1 1
0.00000
HETATM    25 Ru                  0.76417   1.35194  13.84118   Ru1  1 1
0.00000
HETATM    26 Ru                  0.78055   4.05586  13.82055   Ru1  1 1
0.00000
HETATM    27 Ru                  3.13037  -0.01416  13.84119   Ru1  1 1
0.00000
HETATM    28 Ru                  3.13040   2.71809  13.84112   Ru1  1 1
0.00000
HETATM    29 H                   2.34162   1.35192  14.90029    H1  1 1
0.00000
UNIT ENERGY   kcal
ENERGY        -5924.587576511628
END

XTLGRF 200
DESCRP NEBfcc2octa01
RUTYPE SINGLE POINT
#refdata /rf3/training/NEB/fcc_2_octa/01
REMARK Created by geo_energy_extract.py E0 = -254.59857566 eV; -
5920.8971083720935 kcal/mol
CRYSTX     5.40778    5.40778   30.90350   90.00000   90.00000  120.00000
HETATM     1 Ru                  0.77842   1.35195   1.14992   Ru1  1 1
0.00000
HETATM     2 Ru                  0.78055   4.05583   1.14829   Ru1  1 1
0.00000
HETATM     3 Ru                  3.12325  -0.00184   1.14993   Ru1  1 1
0.00000
HETATM     4 Ru                  3.12325   2.70573   1.14992   Ru1  1 1
0.00000
HETATM     5 Ru                  1.56102  -0.00014   3.20686   Ru1  1 1
0.00000
HETATM     6 Ru                  1.56102   2.70402   3.20686   Ru1  1 1
0.00000
HETATM     7 Ru                  3.90274  -1.35194   3.20973   Ru1  1 1
0.00000
HETATM     8 Ru                  3.90288   1.35195   3.20686   Ru1  1 1
0.00000
```

```
HETATM     9 Ru                   0.78175   1.35194   5.35105   Ru1  1 1
0.00000
HETATM    10 Ru                   0.78055   4.05583   5.34701   Ru1  1 1
0.00000
HETATM    11 Ru                   3.12158   0.00104   5.35105   Ru1  1 1
0.00000
HETATM    12 Ru                   3.12158   2.70284   5.35105   Ru1  1 1
0.00000
HETATM    13 Ru                   1.56097  -0.00022   7.50294   Ru1  1 1
0.00000
HETATM    14 Ru                   1.56097   2.70410   7.50294   Ru1  1 1
0.00000
HETATM    15 Ru                   3.90274  -1.35194   7.49941   Ru1  1 1
0.00000
HETATM    16 Ru                   3.90297   1.35195   7.50294   Ru1  1 1
0.00000
HETATM    17 Ru                   0.78543   1.35194   9.64681   Ru1  1 1
0.00000
HETATM    18 Ru                   0.78055   4.05583   9.65603   Ru1  1 1
0.00000
HETATM    19 Ru                   3.11974   0.00423   9.64681   Ru1  1 1
0.00000
HETATM    20 Ru                   3.11975   2.69966   9.64681   Ru1  1 1
0.00000
HETATM    21 Ru                   1.55667  -0.00764  11.78777   Ru1  1 1
0.00000
HETATM    22 Ru                   1.55668   2.71154  11.78777   Ru1  1 1
0.00000
HETATM    23 Ru                   3.90273  -1.35194  11.73989   Ru1  1 1
0.00000
HETATM    24 Ru                   3.91155   1.35195  11.78777   Ru1  1 1
0.00000
HETATM    25 Ru                   0.69906   1.35195  13.84085   Ru1  1 1
0.00000
HETATM    26 Ru                   0.78054   4.05584  13.86833   Ru1  1 1
0.00000
HETATM    27 Ru                   3.16293  -0.07057  13.84085   Ru1  1 1
0.00000
HETATM    28 Ru                   3.16292   2.77446  13.84085   Ru1  1 1
0.00000
HETATM    29 H                    2.34164   1.35194  14.54358    H1  1 1
0.00000
UNIT ENERGY   kcal
ENERGY        -5920.8971083720935
END

XTLGRF 200
DESCRP NEBfcc2octa02
RUTYPE SINGLE POINT
#refdata /rf3/training/NEB/fcc_2_octa/02
REMARK Created by geo_energy_extract.py EO = -254.03991083 eV; -
5907.904903023256 kcal/mol
CRYSTX     5.40778    5.40778   30.90350   90.00000   90.00000  120.00000
HETATM     1 Ru                   0.78034   1.35195   1.15866   Ru1  1 1
0.00000
HETATM     2 Ru                   0.78055   4.05583   1.15860   Ru1  1 1
0.00000
```

```
HETATM    3 Ru                        3.12229  -0.00018   1.15866   Ru1  1 1
0.00000
HETATM    4 Ru                        3.12229   2.70406   1.15866   Ru1  1 1
0.00000
HETATM    5 Ru                        1.56098  -0.00020   3.21599   Ru1  1 1
0.00000
HETATM    6 Ru                        1.56099   2.70409   3.21599   Ru1  1 1
0.00000
HETATM    7 Ru                        3.90274  -1.35194   3.22243   Ru1  1 1
0.00000
HETATM    8 Ru                        3.90295   1.35195   3.21599   Ru1  1 1
0.00000
HETATM    9 Ru                        0.78068   1.35194   5.36139   Ru1  1 1
0.00000
HETATM   10 Ru                        0.78055   4.05583   5.36196   Ru1  1 1
0.00000
HETATM   11 Ru                        3.12211   0.00012   5.36139   Ru1  1 1
0.00000
HETATM   12 Ru                        3.12212   2.70377   5.36139   Ru1  1 1
0.00000
HETATM   13 Ru                        1.56131   0.00037   7.51760   Ru1  1 1
0.00000
HETATM   14 Ru                        1.56131   2.70352   7.51760   Ru1  1 1
0.00000
HETATM   15 Ru                        3.90274  -1.35194   7.51638   Ru1  1 1
0.00000
HETATM   16 Ru                        3.90230   1.35195   7.51760   Ru1  1 1
0.00000
HETATM   17 Ru                        0.78528   1.35195   9.66361   Ru1  1 1
0.00000
HETATM   18 Ru                        0.78055   4.05583   9.67549   Ru1  1 1
0.00000
HETATM   19 Ru                        3.11982   0.00410   9.66361   Ru1  1 1
0.00000
HETATM   20 Ru                        3.11982   2.69979   9.66361   Ru1  1 1
0.00000
HETATM   21 Ru                        1.55373  -0.01273  11.80224   Ru1  1 1
0.00000
HETATM   22 Ru                        1.55374   2.71663  11.80224   Ru1  1 1
0.00000
HETATM   23 Ru                        3.90273  -1.35194  11.77515   Ru1  1 1
0.00000
HETATM   24 Ru                        3.91744   1.35195  11.80224   Ru1  1 1
0.00000
HETATM   25 Ru                        0.65908   1.35194  13.86512   Ru1  1 1
0.00000
HETATM   26 Ru                        0.78054   4.05584  13.89616   Ru1  1 1
0.00000
HETATM   27 Ru                        3.18291  -0.10519  13.86512   Ru1  1 1
0.00000
HETATM   28 Ru                        3.18291   2.80908  13.86512   Ru1  1 1
0.00000
HETATM   29 H                         2.34163   1.35195  14.11014    H1  1 1
0.00000
UNIT ENERGY   kcal
ENERGY        -5907.904903023256
END
```

```
XTLGRF 200
DESCRP NEBfcc2octa03
RUTYPE SINGLE POINT
#refdata /rf3/training/NEB/fcc_2_octa/03
REMARK Created by geo_energy_extract.py EO = -253.76293312 eV; -
5901.463560930233 kcal/mol
CRYSTX     5.40778    5.40778   30.90350   90.00000   90.00000  120.00000
HETATM     1 Ru                  0.78066   1.35195   1.16251   Ru1  1 1
0.00000
HETATM     2 Ru                  0.78055   4.05583   1.16367   Ru1  1 1
0.00000
HETATM     3 Ru                  3.12213   0.00010   1.16251   Ru1  1 1
0.00000
HETATM     4 Ru                  3.12213   2.70379   1.16251   Ru1  1 1
0.00000
HETATM     5 Ru                  1.56107  -0.00005   3.22032   Ru1  1 1
0.00000
HETATM     6 Ru                  1.56106   2.70394   3.22032   Ru1  1 1
0.00000
HETATM     7 Ru                  3.90274  -1.35194   3.22761   Ru1  1 1
0.00000
HETATM     8 Ru                  3.90279   1.35194   3.22032   Ru1  1 1
0.00000
HETATM     9 Ru                  0.77993   1.35194   5.36719   Ru1  1 1
0.00000
HETATM    10 Ru                  0.78055   4.05583   5.37009   Ru1  1 1
0.00000
HETATM    11 Ru                  3.12249  -0.00053   5.36719   Ru1  1 1
0.00000
HETATM    12 Ru                  3.12249   2.70442   5.36719   Ru1  1 1
0.00000
HETATM    13 Ru                  1.56152   0.00074   7.52890   Ru1  1 1
0.00000
HETATM    14 Ru                  1.56152   2.70315   7.52890   Ru1  1 1
0.00000
HETATM    15 Ru                  3.90274  -1.35194   7.52750   Ru1  1 1
0.00000
HETATM    16 Ru                  3.90188   1.35195   7.52890   Ru1  1 1
0.00000
HETATM    17 Ru                  0.78379   1.35194   9.67847   Ru1  1 1
0.00000
HETATM    18 Ru                  0.78055   4.05583   9.69188   Ru1  1 1
0.00000
HETATM    19 Ru                  3.12056   0.00281   9.67847   Ru1  1 1
0.00000
HETATM    20 Ru                  3.12057   2.70108   9.67847   Ru1  1 1
0.00000
HETATM    21 Ru                  1.55173  -0.01621  11.82036   Ru1  1 1
0.00000
HETATM    22 Ru                  1.55173   2.72010  11.82036   Ru1  1 1
0.00000
HETATM    23 Ru                  3.90273  -1.35194  11.81113   Ru1  1 1
0.00000
HETATM    24 Ru                  3.92145   1.35194  11.82036   Ru1  1 1
0.00000
HETATM    25 Ru                  0.65553   1.35194  13.91619   Ru1  1 1
0.00000
```

```
HETATM    26 Ru                    0.78054   4.05584  13.91937   Ru1  1 1
0.00000
HETATM    27 Ru                    3.18468  -0.10827  13.91619   Ru1  1 1
0.00000
HETATM    28 Ru                    3.18468   2.81216  13.91619   Ru1  1 1
0.00000
HETATM    29 H                     2.34163   1.35195  13.67678    H1  1 1
0.00000
UNIT ENERGY   kcal
ENERGY        -5901.463560930233
END

XTLGRF 200
DESCRP NEBfcc2octa04
RUTYPE SINGLE POINT
#refdata /rf3/training/NEB/fcc_2_octa/04
REMARK Created by geo_energy_extract.py EO = -253.83122274 eV; -
5903.0516916279075 kcal/mol
CRYSTX     5.40778    5.40778   30.90350   90.00000   90.00000  120.00000
HETATM     1 Ru                    0.78063   1.35194   1.16736   Ru1  1 1
0.00000
HETATM     2 Ru                    0.78055   4.05583   1.16899   Ru1  1 1
0.00000
HETATM     3 Ru                    3.12214   0.00007   1.16736   Ru1  1 1
0.00000
HETATM     4 Ru                    3.12214   2.70381   1.16736   Ru1  1 1
0.00000
HETATM     5 Ru                    1.56114   0.00007   3.22547   Ru1  1 1
0.00000
HETATM     6 Ru                    1.56113   2.70382   3.22547   Ru1  1 1
0.00000
HETATM     7 Ru                    3.90274  -1.35194   3.23251   Ru1  1 1
0.00000
HETATM     8 Ru                    3.90265   1.35194   3.22547   Ru1  1 1
0.00000
HETATM     9 Ru                    0.77980   1.35195   5.37323   Ru1  1 1
0.00000
HETATM    10 Ru                    0.78055   4.05583   5.37655   Ru1  1 1
0.00000
HETATM    11 Ru                    3.12256  -0.00065   5.37323   Ru1  1 1
0.00000
HETATM    12 Ru                    3.12256   2.70454   5.37323   Ru1  1 1
0.00000
HETATM    13 Ru                    1.56134   0.00043   7.53718   Ru1  1 1
0.00000
HETATM    14 Ru                    1.56133   2.70347   7.53718   Ru1  1 1
0.00000
HETATM    15 Ru                    3.90274  -1.35194   7.53663   Ru1  1 1
0.00000
HETATM    16 Ru                    3.90224   1.35194   7.53718   Ru1  1 1
0.00000
HETATM    17 Ru                    0.78237   1.35195   9.68883   Ru1  1 1
0.00000
HETATM    18 Ru                    0.78055   4.05583   9.69978   Ru1  1 1
0.00000
HETATM    19 Ru                    3.12127   0.00159   9.68883   Ru1  1 1
0.00000
```

```
HETATM    20 Ru                     3.12127   2.70230   9.68883   Ru1  1 1
0.00000
HETATM    21 Ru                     1.54893  -0.02106  11.82998   Ru1  1 1
0.00000
HETATM    22 Ru                     1.54893   2.72495  11.82998   Ru1  1 1
0.00000
HETATM    23 Ru                     3.90273  -1.35194  11.83509   Ru1  1 1
0.00000
HETATM    24 Ru                     3.92705   1.35194  11.82998   Ru1  1 1
0.00000
HETATM    25 Ru                     0.67098   1.35195  13.94279   Ru1  1 1
0.00000
HETATM    26 Ru                     0.78054   4.05584  13.93810   Ru1  1 1
0.00000
HETATM    27 Ru                     3.17696  -0.09490  13.94279   Ru1  1 1
0.00000
HETATM    28 Ru                     3.17696   2.79878  13.94279   Ru1  1 1
0.00000
HETATM    29 H                      2.34164   1.35194  13.39058    H1  1 1
0.00000
UNIT ENERGY   kcal
ENERGY        -5903.0516916279075
END

XTLGRF 200
DESCRP NEBfcc2octa05
RUTYPE SINGLE POINT
#refdata /rf3/training/NEB/fcc_2_octa/05
REMARK Created by geo_energy_extract.py EO = -253.89166393 eV; -
5904.457300697674 kcal/mol
CRYSTX     5.40778    5.40778   30.90350   90.00000   90.00000  120.00000
HETATM     1 Ru                     0.78134   1.35195   1.17783   Ru1  1 1
0.00000
HETATM     2 Ru                     0.78055   4.05583   1.17951   Ru1  1 1
0.00000
HETATM     3 Ru                     3.12178   0.00069   1.17783   Ru1  1 1
0.00000
HETATM     4 Ru                     3.12179   2.70320   1.17783   Ru1  1 1
0.00000
HETATM     5 Ru                     1.56111   0.00002   3.23552   Ru1  1 1
0.00000
HETATM     6 Ru                     1.56110   2.70387   3.23552   Ru1  1 1
0.00000
HETATM     7 Ru                     3.90274  -1.35194   3.24145   Ru1  1 1
0.00000
HETATM     8 Ru                     3.90271   1.35194   3.23552   Ru1  1 1
0.00000
HETATM     9 Ru                     0.77988   1.35195   5.38292   Ru1  1 1
0.00000
HETATM    10 Ru                     0.78055   4.05583   5.38662   Ru1  1 1
0.00000
HETATM    11 Ru                     3.12252  -0.00058   5.38292   Ru1  1 1
0.00000
HETATM    12 Ru                     3.12252   2.70447   5.38292   Ru1  1 1
0.00000
HETATM    13 Ru                     1.56124   0.00025   7.54538   Ru1  1 1
0.00000
```

```
HETATM    14 Ru                     1.56124   2.70364   7.54538   Ru1  1 1
0.00000
HETATM    15 Ru                     3.90274  -1.35194   7.54697   Ru1  1 1
0.00000
HETATM    16 Ru                     3.90244   1.35195   7.54538   Ru1  1 1
0.00000
HETATM    17 Ru                     0.78098   1.35194   9.69718   Ru1  1 1
0.00000
HETATM    18 Ru                     0.78055   4.05583   9.69793   Ru1  1 1
0.00000
HETATM    19 Ru                     3.12196   0.00038   9.69718   Ru1  1 1
0.00000
HETATM    20 Ru                     3.12197   2.70351   9.69718   Ru1  1 1
0.00000
HETATM    21 Ru                     1.54403  -0.02956  11.83230   Ru1  1 1
0.00000
HETATM    22 Ru                     1.54403   2.73345  11.83230   Ru1  1 1
0.00000
HETATM    23 Ru                     3.90274  -1.35194  11.85433   Ru1  1 1
0.00000
HETATM    24 Ru                     3.93686   1.35195  11.83230   Ru1  1 1
0.00000
HETATM    25 Ru                     0.69246   1.35195  13.94873   Ru1  1 1
0.00000
HETATM    26 Ru                     0.78055   4.05583  13.95506   Ru1  1 1
0.00000
HETATM    27 Ru                     3.16623  -0.07629  13.94873   Ru1  1 1
0.00000
HETATM    28 Ru                     3.16622   2.78018  13.94873   Ru1  1 1
0.00000
HETATM    29 H                      2.34164   1.35194  13.10143    H1  1 1
0.00000
UNIT ENERGY   kcal
ENERGY        -5904.457300697674
END

XTLGRF 200
DESCRP NEBfcc2octa06
RUTYPE SINGLE POINT
#refdata /rf3/training/NEB/fcc_2_octa/06
REMARK Created by geo_energy_extract.py EO = -253.82432624 eV; -
5902.891307906977 kcal/mol
CRYSTX     5.40778    5.40778   30.90350   90.00000   90.00000  120.00000
HETATM     1 Ru                     0.78007   1.35194   1.21598   Ru1  1 1
0.00000
HETATM     2 Ru                     0.78055   4.05583   1.21723   Ru1  1 1
0.00000
HETATM     3 Ru                     3.12243  -0.00042   1.21598   Ru1  1 1
0.00000
HETATM     4 Ru                     3.12243   2.70430   1.21598   Ru1  1 1
0.00000
HETATM     5 Ru                     1.56098  -0.00020   3.26428   Ru1  1 1
0.00000
HETATM     6 Ru                     1.56098   2.70409   3.26428   Ru1  1 1
0.00000
HETATM     7 Ru                     3.90274  -1.35194   3.26882   Ru1  1 1
0.00000
```

```
HETATM     8 Ru                   3.90296   1.35195   3.26428   Ru1  1 1
0.00000
HETATM     9 Ru                   0.78024   1.35194   5.38501   Ru1  1 1
0.00000
HETATM    10 Ru                   0.78055   4.05583   5.38772   Ru1  1 1
0.00000
HETATM    11 Ru                   3.12233  -0.00026   5.38501   Ru1  1 1
0.00000
HETATM    12 Ru                   3.12234   2.70415   5.38501   Ru1  1 1
0.00000
HETATM    13 Ru                   1.56135   0.00044   7.51910   Ru1  1 1
0.00000
HETATM    14 Ru                   1.56135   2.70345   7.51910   Ru1  1 1
0.00000
HETATM    15 Ru                   3.90274  -1.35194   7.52091   Ru1  1 1
0.00000
HETATM    16 Ru                   3.90222   1.35195   7.51910   Ru1  1 1
0.00000
HETATM    17 Ru                   0.78180   1.35195   9.64497   Ru1  1 1
0.00000
HETATM    18 Ru                   0.78055   4.05583   9.64317   Ru1  1 1
0.00000
HETATM    19 Ru                   3.12156   0.00108   9.64497   Ru1  1 1
0.00000
HETATM    20 Ru                   3.12156   2.70281   9.64497   Ru1  1 1
0.00000
HETATM    21 Ru                   1.54397  -0.02964  11.75520   Ru1  1 1
0.00000
HETATM    22 Ru                   1.54398   2.73353  11.75520   Ru1  1 1
0.00000
HETATM    23 Ru                   3.90272  -1.35195  11.77198   Ru1  1 1
0.00000
HETATM    24 Ru                   3.93696   1.35195  11.75520   Ru1  1 1
0.00000
HETATM    25 Ru                   0.69177   1.35195  13.85782   Ru1  1 1
0.00000
HETATM    26 Ru                   0.78054   4.05584  13.86560   Ru1  1 1
0.00000
HETATM    27 Ru                   3.16658  -0.07689  13.85782   Ru1  1 1
0.00000
HETATM    28 Ru                   3.16656   2.78077  13.85781   Ru1  1 1
0.00000
HETATM    29 H                    2.34164   1.35194  13.01425    H1  1 1
0.00000
UNIT ENERGY   kcal
ENERGY        -5902.891307906977
END

XTLGRF 200
DESCRP NEBfcc2octaPREbegin
RUTYPE NORMAL RUN
#refdata /rf3/training/NEB/fcc_2_octa/PRE_begin
REMARK Created by geo_energy_extract.py E0 = -254.75723760 eV; -
5924.586920930233 kcal/mol
CRYSTX     5.40778    5.40778   30.90350   90.00000   90.00000  120.00000
HETATM     1 Ru                   0.76926   1.35194   1.23124   Ru1  1 1
0.00000
```

```
HETATM    2 Ru                   0.78055   4.05582   1.22612   Ru1  1 1
0.00000
HETATM    3 Ru                   3.12783  -0.00976   1.23123   Ru1  1 1
0.00000
HETATM    4 Ru                   3.12786   2.71369   1.23126   Ru1  1 1
0.00000
HETATM    5 Ru                   1.56144   0.00062   3.27471   Ru1  1 1
0.00000
HETATM    6 Ru                   1.56150   2.70330   3.27488   Ru1  1 1
0.00000
HETATM    7 Ru                   3.90271  -1.35199   3.28083   Ru1  1 1
0.00000
HETATM    8 Ru                   3.90202   1.35202   3.27486   Ru1  1 1
0.00000
HETATM    9 Ru                   0.78402   1.35190   5.40060   Ru1  1 1
0.00000
HETATM   10 Ru                   0.78056   4.05586   5.39578   Ru1  1 1
0.00000
HETATM   11 Ru                   3.12041   0.00299   5.40057   Ru1  1 1
0.00000
HETATM   12 Ru                   3.12044   2.70087   5.40076   Ru1  1 1
0.00000
HETATM   13 Ru                   1.56136   0.00047   7.52859   Ru1  1 1
0.00000
HETATM   14 Ru                   1.56141   2.70334   7.52862   Ru1  1 1
0.00000
HETATM   15 Ru                   3.90274  -1.35192   7.52279   Ru1  1 1
0.00000
HETATM   16 Ru                   3.90210   1.35195   7.52862   Ru1  1 1
0.00000
HETATM   17 Ru                   0.78081   1.35191   9.65363   Ru1  1 1
0.00000
HETATM   18 Ru                   0.78056   4.05589   9.66658   Ru1  1 1
0.00000
HETATM   19 Ru                   3.12202   0.00020   9.65367   Ru1  1 1
0.00000
HETATM   20 Ru                   3.12203   2.70362   9.65348   Ru1  1 1
0.00000
HETATM   21 Ru                   1.56367   0.00448  11.78460   Ru1  1 1
0.00000
HETATM   22 Ru                   1.56374   2.69940  11.78442   Ru1  1 1
0.00000
HETATM   23 Ru                   3.90271  -1.35197  11.74918   Ru1  1 1
0.00000
HETATM   24 Ru                   3.89753   1.35201  11.78445   Ru1  1 1
0.00000
HETATM   25 Ru                   0.76417   1.35194  13.84118   Ru1  1 1
0.00000
HETATM   26 Ru                   0.78055   4.05586  13.82055   Ru1  1 1
0.00000
HETATM   27 Ru                   3.13037  -0.01416  13.84119   Ru1  1 1
0.00000
HETATM   28 Ru                   3.13040   2.71809  13.84112   Ru1  1 1
0.00000
HETATM   29 H                    2.34162   1.35192  14.90029    H1  1 1
0.00000
UNIT ENERGY   kcal
ENERGY        -5924.586920930233
```

```
END

XTLGRF 200
DESCRP NEBfcc2octaPREend
RUTYPE NORMAL RUN
#refdata /rf3/training/NEB/fcc_2_octa/PRE_end
REMARK Created by geo_energy_extract.py EO = -253.82430091 eV; -
5902.8907188372095 kcal/mol
CRYSTX     5.40778    5.40778   30.90350   90.00000   90.00000  120.00000
HETATM     1 Ru                 0.78007   1.35194   1.21598   Ru1  1 1
0.00000
HETATM     2 Ru                 0.78055   4.05583   1.21723   Ru1  1 1
0.00000
HETATM     3 Ru                 3.12243  -0.00042   1.21598   Ru1  1 1
0.00000
HETATM     4 Ru                 3.12243   2.70430   1.21598   Ru1  1 1
0.00000
HETATM     5 Ru                 1.56098  -0.00020   3.26428   Ru1  1 1
0.00000
HETATM     6 Ru                 1.56098   2.70409   3.26428   Ru1  1 1
0.00000
HETATM     7 Ru                 3.90274  -1.35194   3.26882   Ru1  1 1
0.00000
HETATM     8 Ru                 3.90296   1.35195   3.26428   Ru1  1 1
0.00000
HETATM     9 Ru                 0.78024   1.35194   5.38501   Ru1  1 1
0.00000
HETATM    10 Ru                 0.78055   4.05583   5.38772   Ru1  1 1
0.00000
HETATM    11 Ru                 3.12233  -0.00026   5.38501   Ru1  1 1
0.00000
HETATM    12 Ru                 3.12234   2.70415   5.38501   Ru1  1 1
0.00000
HETATM    13 Ru                 1.56135   0.00044   7.51910   Ru1  1 1
0.00000
HETATM    14 Ru                 1.56135   2.70345   7.51910   Ru1  1 1
0.00000
HETATM    15 Ru                 3.90274  -1.35194   7.52091   Ru1  1 1
0.00000
HETATM    16 Ru                 3.90222   1.35195   7.51910   Ru1  1 1
0.00000
HETATM    17 Ru                 0.78180   1.35195   9.64497   Ru1  1 1
0.00000
HETATM    18 Ru                 0.78055   4.05583   9.64317   Ru1  1 1
0.00000
HETATM    19 Ru                 3.12156   0.00108   9.64497   Ru1  1 1
0.00000
HETATM    20 Ru                 3.12156   2.70281   9.64497   Ru1  1 1
0.00000
HETATM    21 Ru                 1.54397  -0.02964  11.75520   Ru1  1 1
0.00000
HETATM    22 Ru                 1.54398   2.73353  11.75520   Ru1  1 1
0.00000
HETATM    23 Ru                 3.90272  -1.35195  11.77198   Ru1  1 1
0.00000
HETATM    24 Ru                 3.93696   1.35195  11.75520   Ru1  1 1
0.00000
```

```
HETATM    25 Ru                   0.69177   1.35195  13.85782   Ru1  1 1
0.00000
HETATM    26 Ru                   0.78054   4.05584  13.86560   Ru1  1 1
0.00000
HETATM    27 Ru                   3.16658  -0.07689  13.85782   Ru1  1 1
0.00000
HETATM    28 Ru                   3.16656   2.78077  13.85781   Ru1  1 1
0.00000
HETATM    29 H                    2.34164   1.35194  13.01425    H1  1 1
0.00000
UNIT ENERGY   kcal
ENERGY        -5902.8907188372095
END

XTLGRF 200
DESCRP ingNEBfcc2octaTS
RUTYPE SINGLE POINT
#refdata /rf3/training/NEB/reaxNEB_fcc2octa_TS
REMARK Created by geo_energy_extract.py EO = -252.79960484 eV; -
5879.060577674419 kcal/mol
CRYSTX     5.40778    5.40778   31.64350   90.00000   90.00000   90.00000
HETATM     1 Ru                   0.78056   1.35194   0.89412   Ru1  1 1
0.00000
HETATM     2 Ru                   0.78055   4.05583   0.89408   Ru1  1 1
0.00000
HETATM     3 Ru                   3.12218   0.00001   0.89412   Ru1  1 1
0.00000
HETATM     4 Ru                   3.12218   2.70388   0.89412   Ru1  1 1
0.00000
HETATM     5 Ru                   1.56107  -0.00004   3.09217   Ru1  1 1
0.00000
HETATM     6 Ru                   1.56107   2.70393   3.09217   Ru1  1 1
0.00000
HETATM     7 Ru                   3.90273  -1.35195   3.09206   Ru1  1 1
0.00000
HETATM     8 Ru                   3.90277   1.35194   3.09217   Ru1  1 1
0.00000
HETATM     9 Ru                   0.78051   1.35194   5.28297   Ru1  1 1
0.00000
HETATM    10 Ru                   0.78055   4.05583   5.28355   Ru1  1 1
0.00000
HETATM    11 Ru                   3.12220  -0.00003   5.28297   Ru1  1 1
0.00000
HETATM    12 Ru                   3.12220   2.70392   5.28297   Ru1  1 1
0.00000
HETATM    13 Ru                   1.56141   0.00055   7.47351   Ru1  1 1
0.00000
HETATM    14 Ru                   1.56141   2.70334   7.47351   Ru1  1 1
0.00000
HETATM    15 Ru                   3.90273  -1.35195   7.47361   Ru1  1 1
0.00000
HETATM    16 Ru                   3.90209   1.35194   7.47351   Ru1  1 1
0.00000
HETATM    17 Ru                   0.77955   1.35194   9.66607   Ru1  1 1
0.00000
HETATM    18 Ru                   0.78055   4.05583   9.65837   Ru1  1 1
0.00000
```

```
HETATM    19 Ru                   3.12268  -0.00086   9.66607   Ru1  1 1
0.00000
HETATM    20 Ru                   3.12268   2.70475   9.66607   Ru1  1 1
0.00000
HETATM    21 Ru                   1.55504  -0.01048  11.85295   Ru1  1 1
0.00000
HETATM    22 Ru                   1.55504   2.71437  11.85295   Ru1  1 1
0.00000
HETATM    23 Ru                   3.90273  -1.35195  11.87447   Ru1  1 1
0.00000
HETATM    24 Ru                   3.91484   1.35194  11.85295   Ru1  1 1
0.00000
HETATM    25 Ru                   0.74946   1.35194  14.11640   Ru1  1 1
0.00000
HETATM    26 Ru                   0.78055   4.05583  14.06084   Ru1  1 1
0.00000
HETATM    27 Ru                   3.13772  -0.02692  14.11640   Ru1  1 1
0.00000
HETATM    28 Ru                   3.13772   2.73081  14.11640   Ru1  1 1
0.00000
HETATM    29 H                    2.34163   1.35194  13.42984    H1  1 1
0.00000
UNIT ENERGY   kcal
ENERGY        -5879.060577674419
END

BIOGRF 200
DESCRP md_Ru1
REMARK Created by geo_energy_extract.py EO = -2438.58210142 eV; -
56711.21166093024 kcal/mol
RUTYPE SINGLE POINT
#refdata
C:\Surfdrive\Documents\ReaxFF\vaspsrun_files_for_py\geo_extract\md_M\md_R
u
CRYSTX    16.22334    14.04983    17.12672    90.00000    90.00000
90.00000
FORMAT ATOM
(a6,1x,i5,1x,a5,1x,a3,1x,a1,1x,a5,3f10.5,1x,a5,i3,i2,1x,f8.5)
HETATM     1 RU1   RES A   444   0.29098   1.68242   0.94803 0      1 0
0.00000
HETATM     2 RU2   RES A   444  10.99774  11.12226   1.19897 0      1 0
0.00000
HETATM     3 RU3   RES A   444  13.38198   6.42812   1.59686 0      1 0
0.00000
HETATM     4 RU4   RES A   444  11.00686   1.93810   1.38972 0      1 0
0.00000
HETATM     5 RU5   RES A   444   5.34878  10.92780   1.05026 0      1 0
0.00000
HETATM     6 RU6   RES A   444   8.34433   5.95344   0.95484 0      1 0
0.00000
HETATM     7 RU7   RES A   444   5.19446   1.80213   0.94288 0      1 0
0.00000
HETATM     8 RU8   RES A   444   0.16123  10.72368   1.12799 0      1 0
0.00000
HETATM     9 RU9   RES A   444   2.74478   6.06688   1.37469 0      1 0
0.00000
HETATM    10 RU10  RES A   444   0.06102   1.02501   5.40592 0      1 0
0.00000
```

```
HETATM   11 RU11  RES A  444  10.26884  11.00297   5.40085 0      1 0
0.00000
HETATM   12 RU12  RES A  444  13.88053   6.27035   5.36452 0      1 0
0.00000
HETATM   13 RU13  RES A  444  11.06787   0.71273   4.80331 0      1 0
0.00000
HETATM   14 RU14  RES A  444   5.62475  10.52168   4.71803 0      1 0
0.00000
HETATM   15 RU15  RES A  444   8.52133   6.26787   5.35662 0      1 0
0.00000
HETATM   16 RU16  RES A  444   5.12577   1.79756   5.74215 0      1 0
0.00000
HETATM   17 RU17  RES A  444  16.17350  10.70522   5.09932 0      1 0
0.00000
HETATM   18 RU18  RES A  444   2.52367   6.09558   5.45812 0      1 0
0.00000
HETATM   19 RU19  RES A  444  16.09848   1.42377   9.70081 0      1 0
0.00000
HETATM   20 RU20  RES A  444  10.89501  10.95585   9.65568 0      1 0
0.00000
HETATM   21 RU21  RES A  444  13.33762   6.20087  10.16144 0      1 0
0.00000
HETATM   22 RU22  RES A  444  11.03879   1.72596   9.84159 0      1 0
0.00000
HETATM   23 RU23  RES A  444   5.48712  11.10160   9.65686 0      1 0
0.00000
HETATM   24 RU24  RES A  444   8.15724   6.59822   9.48366 0      1 0
0.00000
HETATM   25 RU25  RES A  444   4.76691   1.77149   9.22724 0      1 0
0.00000
HETATM   26 RU26  RES A  444   0.03276  10.56441   9.07124 0      1 0
0.00000
HETATM   27 RU27  RES A  444   2.68461   6.43258   8.99851 0      1 0
0.00000
HETATM   28 RU28  RES A  444   0.10068   1.47434  13.94349 0      1 0
0.00000
HETATM   29 RU29  RES A  444  11.03733  11.41516  14.11227 0      1 0
0.00000
HETATM   30 RU30  RES A  444  13.68241   6.03157  14.04010 0      1 0
0.00000
HETATM   31 RU31  RES A  444  10.89254   1.67250  14.28423 0      1 0
0.00000
HETATM   32 RU32  RES A  444   5.15085  11.18503  13.66681 0      1 0
0.00000
HETATM   33 RU33  RES A  444   8.28625   6.85213  13.79788 0      1 0
0.00000
HETATM   34 RU34  RES A  444   4.98336   1.82402  13.77666 0      1 0
0.00000
HETATM   35 RU35  RES A  444   0.10210  11.27106  13.88623 0      1 0
0.00000
HETATM   36 RU36  RES A  444   2.94962   6.35368  13.70353 0      1 0
0.00000
HETATM   37 RU37  RES A  444  15.03750   3.93402   0.86055 0      1 0
0.00000
HETATM   38 RU38  RES A  444   9.82930  13.00437   1.31374 0      1 0
0.00000
HETATM   39 RU39  RES A  444  12.08860   8.80511   0.68545 0      1 0
0.00000
```

```
HETATM   40 RU40  RES A   444    8.77151   3.58866   0.64070 0       1 0
0.00000
HETATM   41 RU41  RES A   444    3.90344  13.14922   1.22670 0       1 0
0.00000
HETATM   42 RU42  RES A   444    7.24857   8.37156   0.71946 0       1 0
0.00000
HETATM   43 RU43  RES A   444    3.86936   3.86240   1.29806 0       1 0
0.00000
HETATM   44 RU44  RES A   444   15.26066  12.88588   0.49082 0       1 0
0.00000
HETATM   45 RU45  RES A   444    2.33286   8.42008   0.88951 0       1 0
0.00000
HETATM   46 RU46  RES A   444   14.52887   3.75577   5.50516 0       1 0
0.00000
HETATM   47 RU47  RES A   444    9.99285  13.23087   5.50082 0       1 0
0.00000
HETATM   48 RU48  RES A   444   11.80677   8.80369   5.35415 0       1 0
0.00000
HETATM   49 RU49  RES A   444    9.33578   3.94994   6.20448 0       1 0
0.00000
HETATM   50 RU50  RES A   444    3.96959  12.35825   5.29644 0       1 0
0.00000
HETATM   51 RU51  RES A   444    7.20879   8.37816   4.79964 0       1 0
0.00000
HETATM   52 RU52  RES A   444    3.91833   3.90983   5.13609 0       1 0
0.00000
HETATM   53 RU53  RES A   444   15.14055  13.26428   5.07205 0       1 0
0.00000
HETATM   54 RU54  RES A   444    1.09958   8.23697   5.33133 0       1 0
0.00000
HETATM   55 RU55  RES A   444   14.56881   3.70643   9.60703 0       1 0
0.00000
HETATM   56 RU56  RES A   444    9.22210  13.46324   9.74367 0       1 0
0.00000
HETATM   57 RU57  RES A   444   12.13131   8.60738   9.72910 0       1 0
0.00000
HETATM   58 RU58  RES A   444    9.24716   3.88673   9.95417 0       1 0
0.00000
HETATM   59 RU59  RES A   444    3.72077  13.34852   9.92802 0       1 0
0.00000
HETATM   60 RU60  RES A   444    6.84273   8.93886   9.58055 0       1 0
0.00000
HETATM   61 RU61  RES A   444    3.71688   4.31169   9.20454 0       1 0
0.00000
HETATM   62 RU62  RES A   444   14.99703  13.22227  10.08011 0       1 0
0.00000
HETATM   63 RU63  RES A   444    1.28017   8.44194   9.16967 0       1 0
0.00000
HETATM   64 RU64  RES A   444   14.85407   3.68833  14.40831 0       1 0
0.00000
HETATM   65 RU65  RES A   444    9.50335  13.29347  13.88348 0       1 0
0.00000
HETATM   66 RU66  RES A   444   11.95106   8.77308  14.09534 0       1 0
0.00000
HETATM   67 RU67  RES A   444    9.09962   4.13839  14.03460 0       1 0
0.00000
HETATM   68 RU68  RES A   444    4.18186  13.56283  13.98998 0       1 0
0.00000
```

```
HETATM   69 RU69  RES A   444   6.61598   8.95527  13.61403 0       1 0
0.00000
HETATM   70 RU70  RES A   444   4.21672   4.37900  13.81749 0       1 0
0.00000
HETATM   71 RU71  RES A   444  14.69773  13.75817  13.62606 0       1 0
0.00000
HETATM   72 RU72  RES A   444   1.48347   8.04950  13.70324 0       1 0
0.00000
HETATM   73 RU73  RES A   444   2.76964   1.48625   1.16373 0       1 0
0.00000
HETATM   74 RU74  RES A   444  13.70945  11.15446   0.82821 0       1 0
0.00000
HETATM   75 RU75  RES A   444   0.27579   6.30676   1.27770 0       1 0
0.00000
HETATM   76 RU76  RES A   444  13.99339   1.89532   1.53503 0       1 0
0.00000
HETATM   77 RU77  RES A   444   7.89304  10.81423   0.79871 0       1 0
0.00000
HETATM   78 RU78  RES A   444  10.78471   5.80301   0.87734 0       1 0
0.00000
HETATM   79 RU79  RES A   444   7.62795   1.33367   0.91392 0       1 0
0.00000
HETATM   80 RU80  RES A   444   2.52828  10.77569   1.41708 0       1 0
0.00000
HETATM   81 RU81  RES A   444   5.84223   6.23389   1.19435 0       1 0
0.00000
HETATM   82 RU82  RES A   444   2.60121   1.36849   5.15116 0       1 0
0.00000
HETATM   83 RU83  RES A   444  13.48034  11.01319   5.11418 0       1 0
0.00000
HETATM   84 RU84  RES A   444   0.17022   6.09418   5.83129 0       1 0
0.00000
HETATM   85 RU85  RES A   444  13.30281   1.56458   5.79705 0       1 0
0.00000
HETATM   86 RU86  RES A   444   8.12800  10.97643   5.36570 0       1 0
0.00000
HETATM   87 RU87  RES A   444  11.17873   6.46492   5.63113 0       1 0
0.00000
HETATM   88 RU88  RES A   444   8.71339   1.19097   4.98072 0       1 0
0.00000
HETATM   89 RU89  RES A   444   2.45255  10.52016   4.80293 0       1 0
0.00000
HETATM   90 RU90  RES A   444   6.17848   5.98499   5.25421 0       1 0
0.00000
HETATM   91 RU91  RES A   444   2.36140   1.52781   9.87039 0       1 0
0.00000
HETATM   92 RU92  RES A   444  14.05390  10.90125   9.46008 0       1 0
0.00000
HETATM   93 RU93  RES A   444  16.09243   5.76107   9.58255 0       1 0
0.00000
HETATM   94 RU94  RES A   444  13.30820   1.63428   9.78382 0       1 0
0.00000
HETATM   95 RU95  RES A   444   8.17989  11.23185   9.57871 0       1 0
0.00000
HETATM   96 RU96  RES A   444  10.84201   6.69806  10.11441 0       1 0
0.00000
HETATM   97 RU97  RES A   444   8.08697   0.93835  10.05377 0       1 0
0.00000
```

```
HETATM   98 RU98  RES A   444   2.71423  10.40675   9.25270 0       1 0
0.00000
HETATM   99 RU99  RES A   444   5.74967   6.40969   8.94868 0       1 0
0.00000
HETATM  100 RU100 RES A   444   2.51922   2.09924  13.73502 0       1 0
0.00000
HETATM  101 RU101 RES A   444  13.93970  10.80879  13.83670 0       1 0
0.00000
HETATM  102 RU102 RES A   444  16.08748   6.23573  14.24514 0       1 0
0.00000
HETATM  103 RU103 RES A   444  13.43086   1.78050  14.07689 0       1 0
0.00000
HETATM  104 RU104 RES A   444   8.15938  10.97921  14.00396 0       1 0
0.00000
HETATM  105 RU105 RES A   444  10.82045   6.38266  14.09297 0       1 0
0.00000
HETATM  106 RU106 RES A   444   8.09245   1.50631  13.78749 0       1 0
0.00000
HETATM  107 RU107 RES A   444   2.21943  10.52657  13.80144 0       1 0
0.00000
HETATM  108 RU108 RES A   444   5.32723   6.64229  14.02879 0       1 0
0.00000
HETATM  109 RU109 RES A   444   1.07847   4.03891   1.70523 0       1 0
0.00000
HETATM  110 RU110 RES A   444  12.46871  13.35687   1.08594 0       1 0
0.00000
HETATM  111 RU111 RES A   444  14.86190   8.51495   1.02962 0       1 0
0.00000
HETATM  112 RU112 RES A   444  12.53156   4.16881   1.65715 0       1 0
0.00000
HETATM  113 RU113 RES A   444   6.80325  13.25210   0.89197 0       1 0
0.00000
HETATM  114 RU114 RES A   444   9.80819   8.55282   0.95214 0       1 0
0.00000
HETATM  115 RU115 RES A   444   6.30315   3.99404   0.83184 0       1 0
0.00000
HETATM  116 RU116 RES A   444   1.47784  12.80283   0.34255 0       1 0
0.00000
HETATM  117 RU117 RES A   444   4.79553   8.32032   1.38533 0       1 0
0.00000
HETATM  118 RU118 RES A   444   1.19454   3.85462   5.36821 0       1 0
0.00000
HETATM  119 RU119 RES A   444  12.46042  13.62369   5.46034 0       1 0
0.00000
HETATM  120 RU120 RES A   444  14.60246   8.59654   5.36166 0       1 0
0.00000
HETATM  121 RU121 RES A   444  11.58620   3.47764   5.76643 0       1 0
0.00000
HETATM  122 RU122 RES A   444   6.15119  12.80780   5.50878 0       1 0
0.00000
HETATM  123 RU123 RES A   444   9.51922   8.56514   5.77225 0       1 0
0.00000
HETATM  124 RU124 RES A   444   7.18224   3.07648   5.34954 0       1 0
0.00000
HETATM  125 RU125 RES A   444   1.51425  12.89779   5.33810 0       1 0
0.00000
HETATM  126 RU126 RES A   444   4.24430   8.60741   5.29143 0       1 0
0.00000
```

HETATM 127 RU127 RES A 444 1.21047 3.53890 9.60801 0 1 0
0.00000
HETATM 128 RU128 RES A 444 12.38782 13.27196 9.16765 0 1 0
0.00000
HETATM 129 RU129 RES A 444 14.60378 8.24110 9.81470 0 1 0
0.00000
HETATM 130 RU130 RES A 444 11.52781 4.28769 9.93801 0 1 0
0.00000
HETATM 131 RU131 RES A 444 6.64866 13.13343 10.07225 0 1 0
0.00000
HETATM 132 RU132 RES A 444 9.40732 8.96023 10.07929 0 1 0
0.00000
HETATM 133 RU133 RES A 444 6.99672 4.47439 9.82822 0 1 0
0.00000
HETATM 134 RU134 RES A 444 1.31928 12.80540 9.95324 0 1 0
0.00000
HETATM 135 RU135 RES A 444 3.99044 8.59258 9.18918 0 1 0
0.00000
HETATM 136 RU136 RES A 444 1.01335 4.27639 13.62313 0 1 0
0.00000
HETATM 137 RU137 RES A 444 11.87028 13.77270 14.07013 0 1 0
0.00000
HETATM 138 RU138 RES A 444 15.77836 8.74915 14.28647 0 1 0
0.00000
HETATM 139 RU139 RES A 444 11.89059 4.10124 14.16684 0 1 0
0.00000
HETATM 140 RU140 RES A 444 6.71517 13.30090 14.02543 0 1 0
0.00000
HETATM 141 RU141 RES A 444 9.60983 9.20863 14.29075 0 1 0
0.00000
HETATM 142 RU142 RES A 444 6.56713 3.81554 13.81292 0 1 0
0.00000
HETATM 143 RU143 RES A 444 1.49918 13.71861 13.69858 0 1 0
0.00000
HETATM 144 RU144 RES A 444 3.97757 8.71923 13.64629 0 1 0
0.00000
HETATM 145 RU145 RES A 444 1.50722 0.58977 3.09862 0 1 0
0.00000
HETATM 146 RU146 RES A 444 12.22985 10.24522 2.99163 0 1 0
0.00000
HETATM 147 RU147 RES A 444 15.17081 4.89460 3.09021 0 1 0
0.00000
HETATM 148 RU148 RES A 444 12.49436 0.73566 3.02334 0 1 0
0.00000
HETATM 149 RU149 RES A 444 6.69962 9.30822 2.63025 0 1 0
0.00000
HETATM 150 RU150 RES A 444 9.71871 5.09871 3.25118 0 1 0
0.00000
HETATM 151 RU151 RES A 444 6.52601 0.81930 3.54594 0 1 0
0.00000
HETATM 152 RU152 RES A 444 1.15248 9.00644 2.82408 0 1 0
0.00000
HETATM 153 RU153 RES A 444 4.00592 5.44511 3.22437 0 1 0
0.00000
HETATM 154 RU154 RES A 444 1.30107 0.80005 7.65458 0 1 0
0.00000
HETATM 155 RU155 RES A 444 12.12797 10.51411 7.37466 0 1 0
0.00000

HETATM   156 RU156 RES A   444 14.83077   5.17810   7.53037 0   1 0
0.00000
HETATM   157 RU157 RES A   444 12.06745   0.55723   7.62207 0   1 0
0.00000
HETATM   158 RU158 RES A   444   6.70953 10.25692   7.52219 0   1 0
0.00000
HETATM   159 RU159 RES A   444   9.97472   5.46681   7.99091 0   1 0
0.00000
HETATM   160 RU160 RES A   444   6.30044   0.60876   7.63188 0   1 0
0.00000
HETATM   161 RU161 RES A   444   1.33743   9.94543   6.96525 0   1 0
0.00000
HETATM   162 RU162 RES A   444   4.24406   5.62613   7.35942 0   1 0
0.00000
HETATM   163 RU163 RES A   444   1.15595   0.70826 11.90610 0   1 0
0.00000
HETATM   164 RU164 RES A   444 12.24149 10.54744 12.00467 0   1 0
0.00000
HETATM   165 RU165 RES A   444 15.04212   5.55589 12.01943 0   1 0
0.00000
HETATM   166 RU166 RES A   444 12.17944   1.09378 11.79633 0   1 0
0.00000
HETATM   167 RU167 RES A   444   6.92205 10.57621 11.54241 0   1 0
0.00000
HETATM   168 RU168 RES A   444   9.23529   5.96387 12.03275 0   1 0
0.00000
HETATM   169 RU169 RES A   444   6.20564   1.30004 11.33517 0   1 0
0.00000
HETATM   170 RU170 RES A   444   1.38330 10.40506 11.44855 0   1 0
0.00000
HETATM   171 RU171 RES A   444   4.41445   5.85074 11.21930 0   1 0
0.00000
HETATM   172 RU172 RES A   444   1.30480   0.58668 16.17529 0   1 0
0.00000
HETATM   173 RU173 RES A   444 12.27097 10.25423 15.95887 0   1 0
0.00000
HETATM   174 RU174 RES A   444 14.99014   5.12990 16.33158 0   1 0
0.00000
HETATM   175 RU175 RES A   444 12.19551   1.02822 16.53042 0   1 0
0.00000
HETATM   176 RU176 RES A   444   6.59579 10.20551 15.73904 0   1 0
0.00000
HETATM   177 RU177 RES A   444   9.42484   5.42508 15.77052 0   1 0
0.00000
HETATM   178 RU178 RES A   444   6.40403   0.71147 15.94852 0   1 0
0.00000
HETATM   179 RU179 RES A   444   1.31681   9.95356 16.08109 0   1 0
0.00000
HETATM   180 RU180 RES A   444   4.40503   5.42976 16.66542 0   1 0
0.00000
HETATM   181 RU181 RES A   444 15.71882   2.67244   3.60425 0   1 0
0.00000
HETATM   182 RU182 RES A   444 11.21667 12.23417   3.51600 0   1 0
0.00000
HETATM   183 RU183 RES A   444 13.06043   7.94624   3.58671 0   1 0
0.00000
HETATM   184 RU184 RES A   444 10.53912   3.01351   3.35594 0   1 0
0.00000

HETATM   185 RU185 RES A   444   5.39435 12.33838   2.75888 0   1 0
0.00000
HETATM   186 RU186 RES A   444   8.36821   7.31607   3.08992 0   1 0
0.00000
HETATM   187 RU187 RES A   444   5.37116   3.01513   3.18497 0   1 0
0.00000
HETATM   188 RU188 RES A   444   0.33303 12.18579   2.88138 0   1 0
0.00000
HETATM   189 RU189 RES A   444   3.02634   7.85008   3.29256 0   1 0
0.00000
HETATM   190 RU190 RES A   444 16.19252   2.78407   7.29259 0   1 0
0.00000
HETATM   191 RU191 RES A   444 10.72590 12.52843   7.75210 0   1 0
0.00000
HETATM   192 RU192 RES A   444 13.32407   7.94986   7.09812 0   1 0
0.00000
HETATM   193 RU193 RES A   444 10.24616   2.29502   7.43300 0   1 0
0.00000
HETATM   194 RU194 RES A   444   5.41319 12.54621   7.80035 0   1 0
0.00000
HETATM   195 RU195 RES A   444   7.99686   7.77202   7.36983 0   1 0
0.00000
HETATM   196 RU196 RES A   444   5.72407   3.72640   7.64321 0   1 0
0.00000
HETATM   197 RU197 RES A   444 16.11824 12.12973   7.06820 0   1 0
0.00000
HETATM   198 RU198 RES A   444   2.73653   7.74257   7.05682 0   1 0
0.00000
HETATM   199 RU199 RES A   444 15.90859   3.01086 11.91618 0   1 0
0.00000
HETATM   200 RU200 RES A   444 11.08320 12.67362 11.33416 0   1 0
0.00000
HETATM   201 RU201 RES A   444 13.72042   8.21003 12.50897 0   1 0
0.00000
HETATM   202 RU202 RES A   444 10.62918   3.44764 12.06207 0   1 0
0.00000
HETATM   203 RU203 RES A   444   5.14154 12.74701 11.87722 0   1 0
0.00000
HETATM   204 RU204 RES A   444   7.85501   8.22515 11.77087 0   1 0
0.00000
HETATM   205 RU205 RES A   444   5.24492   3.60155 11.83403 0   1 0
0.00000
HETATM   206 RU206 RES A   444 16.07252 12.84744 12.02652 0   1 0
0.00000
HETATM   207 RU207 RES A   444   2.86855   7.76370 11.66037 0   1 0
0.00000
HETATM   208 RU208 RES A   444   0.56148   2.96321 15.78664 0   1 0
0.00000
HETATM   209 RU209 RES A   444 10.94451 12.72183 16.31574 0   1 0
0.00000
HETATM   210 RU210 RES A   444 13.75048   8.02624 15.93339 0   1 0
0.00000
HETATM   211 RU211 RES A   444 10.90513   3.64470 16.43677 0   1 0
0.00000
HETATM   212 RU212 RES A   444   4.96258 12.18629 15.95275 0   1 0
0.00000
HETATM   213 RU213 RES A   444   8.25104   8.11131 15.73630 0   1 0
0.00000

```
HETATM   214 RU214 RES A   444   5.09597   3.06258  15.93376 0       1 0
0.00000
HETATM   215 RU215 RES A   444   0.36143  13.40308  15.75811 0       1 0
0.00000
HETATM   216 RU216 RES A   444   2.70021   7.51217  15.72647 0       1 0
0.00000
HETATM   217 RU217 RES A   444   4.02542   0.56189   3.24913 0       1 0
0.00000
HETATM   218 RU218 RES A   444  14.76097  10.05354   3.21004 0       1 0
0.00000
HETATM   219 RU219 RES A   444   1.31476   5.96374   3.49035 0       1 0
0.00000
HETATM   220 RU220 RES A   444  15.17743   0.31348   2.96589 0       1 0
0.00000
HETATM   221 RU221 RES A   444   9.14618   9.76300   3.05320 0       1 0
0.00000
HETATM   222 RU222 RES A   444  12.45241   5.47553   3.85693 0       1 0
0.00000
HETATM   223 RU223 RES A   444   9.01399   0.45265   2.43164 0       1 0
0.00000
HETATM   224 RU224 RES A   444   4.10146  10.39577   3.12264 0       1 0
0.00000
HETATM   225 RU225 RES A   444   7.06450   5.11567   3.20344 0       1 0
0.00000
HETATM   226 RU226 RES A   444   3.69689   0.51286   7.35271 0       1 0
0.00000
HETATM   227 RU227 RES A   444  14.76214   9.98410   7.11119 0       1 0
0.00000
HETATM   228 RU228 RES A   444   1.42469   5.11411   7.74990 0       1 0
0.00000
HETATM   229 RU229 RES A   444  14.67725   0.88604   7.60778 0       1 0
0.00000
HETATM   230 RU230 RES A   444   9.32962  10.10219   7.60797 0       1 0
0.00000
HETATM   231 RU231 RES A   444  12.51836   5.49246   7.27953 0       1 0
0.00000
HETATM   232 RU232 RES A   444   9.43049   0.31043   7.86407 0       1 0
0.00000
HETATM   233 RU233 RES A   444   4.17779  10.40288   7.02309 0       1 0
0.00000
HETATM   234 RU234 RES A   444   7.68024   5.13532   7.37746 0       1 0
0.00000
HETATM   235 RU235 RES A   444   3.87080   0.85490  11.46790 0       1 0
0.00000
HETATM   236 RU236 RES A   444  15.31885  10.10423  11.76848 0       1 0
0.00000
HETATM   237 RU237 RES A   444   1.43966   5.75684  11.18062 0       1 0
0.00000
HETATM   238 RU238 RES A   444  14.71116   0.92587  12.15401 0       1 0
0.00000
HETATM   239 RU239 RES A   444   9.50071  10.67097  11.91112 0       1 0
0.00000
HETATM   240 RU240 RES A   444  12.07132   5.65926  12.22170 0       1 0
0.00000
HETATM   241 RU241 RES A   444   9.53445   0.89729  11.62970 0       1 0
0.00000
HETATM   242 RU242 RES A   444   3.91341  10.26279  11.41701 0       1 0
0.00000
```

```
HETATM   243 RU243 RES A   444   6.81912   5.75590  12.29595 0       1 0
0.00000
HETATM   244 RU244 RES A   444   4.07124   0.94693  16.14221 0       1 0
0.00000
HETATM   245 RU245 RES A   444  14.91795  10.42479  15.95466 0       1 0
0.00000
HETATM   246 RU246 RES A   444   1.11748   5.47270  16.06423 0       1 0
0.00000
HETATM   247 RU247 RES A   444  14.66078   0.71303  15.96080 0       1 0
0.00000
HETATM   248 RU248 RES A   444   9.82137  10.42344  16.21842 0       1 0
0.00000
HETATM   249 RU249 RES A   444  12.72776   5.39706  16.06212 0       1 0
0.00000
HETATM   250 RU250 RES A   444   9.58981   1.32804  16.50994 0       1 0
0.00000
HETATM   251 RU251 RES A   444   3.56089   9.83094  16.47474 0       1 0
0.00000
HETATM   252 RU252 RES A   444   6.95273   5.37005  15.84853 0       1 0
0.00000
HETATM   253 RU253 RES A   444   2.49628   2.85337   3.32775 0       1 0
0.00000
HETATM   254 RU254 RES A   444  13.71190  12.47968   2.79214 0       1 0
0.00000
HETATM   255 RU255 RES A   444  15.32939   7.54787   3.25843 0       1 0
0.00000
HETATM   256 RU256 RES A   444  13.27428   2.93698   3.75719 0       1 0
0.00000
HETATM   257 RU257 RES A   444   8.11191  12.22503   3.01621 0       1 0
0.00000
HETATM   258 RU258 RES A   444  10.70795   7.69248   3.19397 0       1 0
0.00000
HETATM   259 RU259 RES A   444   7.97018   2.83419   2.88684 0       1 0
0.00000
HETATM   260 RU260 RES A   444   2.67746  13.00062   3.17215 0       1 0
0.00000
HETATM   261 RU261 RES A   444   5.40234   7.15599   3.55494 0       1 0
0.00000
HETATM   262 RU262 RES A   444   2.72477   3.00025   7.30355 0       1 0
0.00000
HETATM   263 RU263 RES A   444  13.75616  12.30254   7.35779 0       1 0
0.00000
HETATM   264 RU264 RES A   444  15.61400   7.66134   7.59787 0       1 0
0.00000
HETATM   265 RU265 RES A   444  12.91229   3.07849   7.97664 0       1 0
0.00000
HETATM   266 RU266 RES A   444   7.95298  12.67229   7.17823 0       1 0
0.00000
HETATM   267 RU267 RES A   444  10.83132   7.84284   7.83531 0       1 0
0.00000
HETATM   268 RU268 RES A   444   7.81010   2.52979   7.98352 0       1 0
0.00000
HETATM   269 RU269 RES A   444   2.90569  12.14502   7.47257 0       1 0
0.00000
HETATM   270 RU270 RES A   444   5.48721   8.06929   7.30596 0       1 0
0.00000
HETATM   271 RU271 RES A   444   2.74936   3.89135  11.67155 0       1 0
0.00000
```

```
HETATM   272 RU272 RES A   444  13.68140  12.66836  12.07652 0      1 0
0.00000
HETATM   273 RU273 RES A   444   0.04553   7.77574  11.32433 0      1 0
0.00000
HETATM   274 RU274 RES A   444  13.27583   3.49301  11.81493 0      1 0
0.00000
HETATM   275 RU275 RES A   444   8.01302  12.73770  12.07778 0      1 0
0.00000
HETATM   276 RU276 RES A   444  11.13476   8.36433  12.00547 0      1 0
0.00000
HETATM   277 RU277 RES A   444   7.94504   3.33492  11.60840 0      1 0
0.00000
HETATM   278 RU278 RES A   444   2.77019  12.58676  11.93200 0      1 0
0.00000
HETATM   279 RU279 RES A   444   5.36514   8.05554  11.39995 0      1 0
0.00000
HETATM   280 RU280 RES A   444   2.83050   3.24187  16.28849 0      1 0
0.00000
HETATM   281 RU281 RES A   444  13.40766  12.64966  15.49457 0      1 0
0.00000
HETATM   282 RU282 RES A   444   0.07208   8.02073  16.55059 0      1 0
0.00000
HETATM   283 RU283 RES A   444  13.60942   3.05049  16.44483 0      1 0
0.00000
HETATM   284 RU284 RES A   444   8.36607  12.37084  16.03873 0      1 0
0.00000
HETATM   285 RU285 RES A   444  11.08751   7.59416  16.23787 0      1 0
0.00000
HETATM   286 RU286 RES A   444   7.49886   3.04383  16.01673 0      1 0
0.00000
HETATM   287 RU287 RES A   444   2.70493  11.96776  15.33286 0      1 0
0.00000
HETATM   288 RU288 RES A   444   5.41368   8.18163  15.93699 0      1 0
0.00000
UNIT ENERGY   kcal
ENERGY        -56711.21166093024
END

BIOGRF 200
DESCRP md_Ru2
REMARK Created by geo_energy_extract.py EO = -2554.28555745 eV; -
59401.98970813954 kcal/mol
RUTYPE SINGLE POINT
#refdata
C:\Surfdrive\Documents\ReaxFF\vaspsrun_files_for_py\geo_extract\md_M\md_R
u
CRYSTX    16.22334    14.04983    17.12672    90.00000    90.00000
90.00000
FORMAT ATOM
(a6,1x,i5,1x,a5,1x,a3,1x,a1,1x,a5,3f10.5,1x,a5,i3,i2,1x,f8.5)
HETATM     1 RU1   RES A   444   0.17299   1.57448   1.15005 0      1 0
0.00000
HETATM     2 RU2   RES A   444  10.87630  10.69128   0.94800 0      1 0
0.00000
HETATM     3 RU3   RES A   444  13.78151   6.18251   1.17647 0      1 0
0.00000
HETATM     4 RU4   RES A   444  10.69417   1.52475   0.95420 0      1 0
0.00000
```

```
HETATM    5 RU5   RES A   444    5.58095  10.62129   0.94863 0       1 0
0.00000
HETATM    6 RU6   RES A   444    8.12456   6.24692   1.49591 0       1 0
0.00000
HETATM    7 RU7   RES A   444    5.60414   1.35710   0.99262 0       1 0
0.00000
HETATM    8 RU8   RES A   444   16.17179  10.78511   0.78288 0       1 0
0.00000
HETATM    9 RU9   RES A   444    2.67948   6.08292   0.74806 0       1 0
0.00000
HETATM   10 RU10  RES A   444    0.06829   1.30651   5.23257 0       1 0
0.00000
HETATM   11 RU11  RES A   444   10.65670  10.73992   5.26049 0       1 0
0.00000
HETATM   12 RU12  RES A   444   13.45247   5.74052   5.48287 0       1 0
0.00000
HETATM   13 RU13  RES A   444   10.69843   1.00939   5.65043 0       1 0
0.00000
HETATM   14 RU14  RES A   444    5.32413  10.63543   5.28142 0       1 0
0.00000
HETATM   15 RU15  RES A   444    8.10781   6.19444   5.44817 0       1 0
0.00000
HETATM   16 RU16  RES A   444    5.30609   1.39451   5.28311 0       1 0
0.00000
HETATM   17 RU17  RES A   444   15.93589  10.56051   5.38185 0       1 0
0.00000
HETATM   18 RU18  RES A   444    2.74890   6.11226   5.16168 0       1 0
0.00000
HETATM   19 RU19  RES A   444    0.01514   1.66963   9.77080 0       1 0
0.00000
HETATM   20 RU20  RES A   444   10.80729  11.07503   9.68115 0       1 0
0.00000
HETATM   21 RU21  RES A   444   13.51251   6.15328   9.59516 0       1 0
0.00000
HETATM   22 RU22  RES A   444   10.65943   1.58682   9.97606 0       1 0
0.00000
HETATM   23 RU23  RES A   444    5.24279  10.88577   9.60482 0       1 0
0.00000
HETATM   24 RU24  RES A   444    8.28584   6.37887   9.57337 0       1 0
0.00000
HETATM   25 RU25  RES A   444    5.27639   1.54846   9.72415 0       1 0
0.00000
HETATM   26 RU26  RES A   444    0.00867  11.18870   9.47957 0       1 0
0.00000
HETATM   27 RU27  RES A   444    2.52783   6.31373   9.79672 0       1 0
0.00000
HETATM   28 RU28  RES A   444   16.20290   1.77651  13.95013 0       1 0
0.00000
HETATM   29 RU29  RES A   444   10.59269  11.37496  13.83518 0       1 0
0.00000
HETATM   30 RU30  RES A   444   13.62111   6.48567  14.11182 0       1 0
0.00000
HETATM   31 RU31  RES A   444   10.96955   1.53149  13.84776 0       1 0
0.00000
HETATM   32 RU32  RES A   444    5.83239  10.97771  14.09351 0       1 0
0.00000
HETATM   33 RU33  RES A   444    8.11933   6.20979  13.85694 0       1 0
0.00000
```

```
HETATM   34 RU34  RES A  444   5.24394   1.58340  14.08572 0       1 0
0.00000
HETATM   35 RU35  RES A  444  16.20578  11.07702  13.70078 0       1 0
0.00000
HETATM   36 RU36  RES A  444   2.72357   6.48602  13.83743 0       1 0
0.00000
HETATM   37 RU37  RES A  444  14.70010   3.71246   1.33918 0       1 0
0.00000
HETATM   38 RU38  RES A  444   9.34309  13.33185   1.06337 0       1 0
0.00000
HETATM   39 RU39  RES A  444  12.08256   8.32867   1.17540 0       1 0
0.00000
HETATM   40 RU40  RES A  444   9.36165   3.82622   1.28242 0       1 0
0.00000
HETATM   41 RU41  RES A  444   4.25277  13.09848   1.28288 0       1 0
0.00000
HETATM   42 RU42  RES A  444   6.86006   8.48081   1.17688 0       1 0
0.00000
HETATM   43 RU43  RES A  444   4.12104   3.90758   1.20505 0       1 0
0.00000
HETATM   44 RU44  RES A  444  14.84443  13.19403   1.40462 0       1 0
0.00000
HETATM   45 RU45  RES A  444   1.19867   8.56299   0.70193 0       1 0
0.00000
HETATM   46 RU46  RES A  444  14.70655   3.52267   5.32117 0       1 0
0.00000
HETATM   47 RU47  RES A  444   9.54348  13.13197   5.26675 0       1 0
0.00000
HETATM   48 RU48  RES A  444  12.22281   8.56292   5.38632 0       1 0
0.00000
HETATM   49 RU49  RES A  444   9.26484   3.71787   5.51013 0       1 0
0.00000
HETATM   50 RU50  RES A  444   3.73908  12.57958   5.18816 0       1 0
0.00000
HETATM   51 RU51  RES A  444   6.65988   8.41840   5.34220 0       1 0
0.00000
HETATM   52 RU52  RES A  444   3.92953   3.81383   5.22133 0       1 0
0.00000
HETATM   53 RU53  RES A  444  14.57099  12.98488   5.21943 0       1 0
0.00000
HETATM   54 RU54  RES A  444   1.30860   8.37333   5.28951 0       1 0
0.00000
HETATM   55 RU55  RES A  444  15.04787   4.02753   9.69854 0       1 0
0.00000
HETATM   56 RU56  RES A  444   9.21168  13.33549   9.81674 0       1 0
0.00000
HETATM   57 RU57  RES A  444  12.15112   8.87646   9.57379 0       1 0
0.00000
HETATM   58 RU58  RES A  444   9.52188   4.05719   9.69719 0       1 0
0.00000
HETATM   59 RU59  RES A  444   4.13886  13.17540   9.58043 0       1 0
0.00000
HETATM   60 RU60  RES A  444   6.93583   8.34233   9.69558 0       1 0
0.00000
HETATM   61 RU61  RES A  444   4.06716   3.83631   9.69964 0       1 0
0.00000
HETATM   62 RU62  RES A  444  14.95278  13.42487   9.76783 0       1 0
0.00000
```

HETATM 63 RU63 RES A 444 1.52330 8.78225 9.69501 0 1 0
0.00000
HETATM 64 RU64 RES A 444 14.89188 4.28141 13.82991 0 1 0
0.00000
HETATM 65 RU65 RES A 444 9.32393 13.48321 13.90019 0 1 0
0.00000
HETATM 66 RU66 RES A 444 12.19370 8.88049 13.55606 0 1 0
0.00000
HETATM 67 RU67 RES A 444 9.43864 3.99514 14.10680 0 1 0
0.00000
HETATM 68 RU68 RES A 444 4.11036 13.51321 13.93155 0 1 0
0.00000
HETATM 69 RU69 RES A 444 6.94877 8.61743 13.71401 0 1 0
0.00000
HETATM 70 RU70 RES A 444 4.27511 3.94901 13.72131 0 1 0
0.00000
HETATM 71 RU71 RES A 444 15.05736 13.57155 14.24870 0 1 0
0.00000
HETATM 72 RU72 RES A 444 1.42000 8.94269 13.78470 0 1 0
0.00000
HETATM 73 RU73 RES A 444 2.74982 1.57336 0.97794 0 1 0
0.00000
HETATM 74 RU74 RES A 444 13.75911 10.59075 0.90786 0 1 0
0.00000
HETATM 75 RU75 RES A 444 0.21657 6.24590 0.97797 0 1 0
0.00000
HETATM 76 RU76 RES A 444 13.52505 1.45945 1.11848 0 1 0
0.00000
HETATM 77 RU77 RES A 444 8.06462 11.02335 1.03185 0 1 0
0.00000
HETATM 78 RU78 RES A 444 10.80644 6.16816 1.36312 0 1 0
0.00000
HETATM 79 RU79 RES A 444 8.33206 1.49417 1.34205 0 1 0
0.00000
HETATM 80 RU80 RES A 444 2.93759 10.81039 0.66632 0 1 0
0.00000
HETATM 81 RU81 RES A 444 5.54641 6.25454 0.93326 0 1 0
0.00000
HETATM 82 RU82 RES A 444 2.82990 1.45658 5.32869 0 1 0
0.00000
HETATM 83 RU83 RES A 444 13.38578 10.84188 5.49255 0 1 0
0.00000
HETATM 84 RU84 RES A 444 0.06533 5.97272 5.10526 0 1 0
0.00000
HETATM 85 RU85 RES A 444 13.14442 0.93557 5.65667 0 1 0
0.00000
HETATM 86 RU86 RES A 444 7.92960 10.94288 5.44181 0 1 0
0.00000
HETATM 87 RU87 RES A 444 10.86083 6.06907 5.15584 0 1 0
0.00000
HETATM 88 RU88 RES A 444 7.88698 1.27477 5.31789 0 1 0
0.00000
HETATM 89 RU89 RES A 444 2.56105 10.61235 4.88739 0 1 0
0.00000
HETATM 90 RU90 RES A 444 5.39075 6.02247 5.29491 0 1 0
0.00000
HETATM 91 RU91 RES A 444 2.48741 1.89650 9.91149 0 1 0
0.00000

```
HETATM   92 RU92  RES A   444  13.76673  10.92687   9.70580 0       1 0
0.00000
HETATM   93 RU93  RES A   444   0.01098   6.34034   9.53971 0       1 0
0.00000
HETATM   94 RU94  RES A   444  13.71516   1.65408   9.59567 0       1 0
0.00000
HETATM   95 RU95  RES A   444   8.21633  11.09743   9.58056 0       1 0
0.00000
HETATM   96 RU96  RES A   444  10.87913   6.12250   9.71545 0       1 0
0.00000
HETATM   97 RU97  RES A   444   7.97606   1.65565   9.50723 0       1 0
0.00000
HETATM   98 RU98  RES A   444   2.48955  11.47252   9.79895 0       1 0
0.00000
HETATM   99 RU99  RES A   444   5.48888   6.05076   9.60635 0       1 0
0.00000
HETATM  100 RU100 RES A   444   2.81075   1.64441  13.60557 0       1 0
0.00000
HETATM  101 RU101 RES A   444  13.48699  11.35600  13.85556 0       1 0
0.00000
HETATM  102 RU102 RES A   444  16.09434   6.69107  13.93923 0       1 0
0.00000
HETATM  103 RU103 RES A   444  13.49135   1.83027  13.85594 0       1 0
0.00000
HETATM  104 RU104 RES A   444   8.18431  10.76870  13.86918 0       1 0
0.00000
HETATM  105 RU105 RES A   444  11.05441   6.47799  14.15428 0       1 0
0.00000
HETATM  106 RU106 RES A   444   8.38556   1.80022  14.09949 0       1 0
0.00000
HETATM  107 RU107 RES A   444   2.82928  11.37858  13.88731 0       1 0
0.00000
HETATM  108 RU108 RES A   444   5.53650   6.33117  13.77888 0       1 0
0.00000
HETATM  109 RU109 RES A   444   1.53011   3.82559   1.02087 0       1 0
0.00000
HETATM  110 RU110 RES A   444  11.99991  13.33377   1.03148 0       1 0
0.00000
HETATM  111 RU111 RES A   444  14.93633   8.48226   1.23057 0       1 0
0.00000
HETATM  112 RU112 RES A   444  12.01625   3.79834   1.10145 0       1 0
0.00000
HETATM  113 RU113 RES A   444   6.95461  13.32035   0.93689 0       1 0
0.00000
HETATM  114 RU114 RES A   444   9.47123   8.41914   1.07790 0       1 0
0.00000
HETATM  115 RU115 RES A   444   6.89130   3.64639   1.32360 0       1 0
0.00000
HETATM  116 RU116 RES A   444   1.44180  13.24610   1.24029 0       1 0
0.00000
HETATM  117 RU117 RES A   444   3.95300   8.46925   0.77675 0       1 0
0.00000
HETATM  118 RU118 RES A   444   1.47787   3.84966   5.64748 0       1 0
0.00000
HETATM  119 RU119 RES A   444  11.95798  12.96109   5.23655 0       1 0
0.00000
HETATM  120 RU120 RES A   444  15.04128   8.15237   5.00697 0       1 0
0.00000
```

HETATM 121 RU121 RES A 444 12.09204 3.43224 5.36747 0 1 0
0.00000
HETATM 122 RU122 RES A 444 6.69754 13.06404 5.28894 0 1 0
0.00000
HETATM 123 RU123 RES A 444 9.59903 8.59425 5.49593 0 1 0
0.00000
HETATM 124 RU124 RES A 444 6.52658 3.72340 5.11590 0 1 0
0.00000
HETATM 125 RU125 RES A 444 1.20274 12.61275 5.29497 0 1 0
0.00000
HETATM 126 RU126 RES A 444 3.77834 8.58272 5.28238 0 1 0
0.00000
HETATM 127 RU127 RES A 444 1.39604 4.17183 9.65924 0 1 0
0.00000
HETATM 128 RU128 RES A 444 12.18264 13.32919 9.55545 0 1 0
0.00000
HETATM 129 RU129 RES A 444 14.89328 8.71920 9.61724 0 1 0
0.00000
HETATM 130 RU130 RES A 444 12.05011 3.76469 9.74691 0 1 0
0.00000
HETATM 131 RU131 RES A 444 6.68983 13.51356 9.82606 0 1 0
0.00000
HETATM 132 RU132 RES A 444 9.71438 8.48805 9.63973 0 1 0
0.00000
HETATM 133 RU133 RES A 444 6.89091 3.68101 9.66226 0 1 0
0.00000
HETATM 134 RU134 RES A 444 1.26930 13.46707 9.37967 0 1 0
0.00000
HETATM 135 RU135 RES A 444 4.04431 8.46454 9.49848 0 1 0
0.00000
HETATM 136 RU136 RES A 444 1.37853 3.94641 14.06425 0 1 0
0.00000
HETATM 137 RU137 RES A 444 12.06912 13.40908 13.99944 0 1 0
0.00000
HETATM 138 RU138 RES A 444 14.81888 8.89939 13.69854 0 1 0
0.00000
HETATM 139 RU139 RES A 444 12.21365 4.01603 13.91520 0 1 0
0.00000
HETATM 140 RU140 RES A 444 6.58122 13.57076 13.83033 0 1 0
0.00000
HETATM 141 RU141 RES A 444 9.57496 8.59938 13.97345 0 1 0
0.00000
HETATM 142 RU142 RES A 444 6.99294 3.93708 14.01796 0 1 0
0.00000
HETATM 143 RU143 RES A 444 1.22498 13.34338 13.79885 0 1 0
0.00000
HETATM 144 RU144 RES A 444 4.02402 9.07075 13.66791 0 1 0
0.00000
HETATM 145 RU145 RES A 444 1.45772 0.46045 3.19733 0 1 0
0.00000
HETATM 146 RU146 RES A 444 12.14584 9.83571 3.12002 0 1 0
0.00000
HETATM 147 RU147 RES A 444 14.71681 5.18112 3.22644 0 1 0
0.00000
HETATM 148 RU148 RES A 444 11.96994 0.32017 3.22732 0 1 0
0.00000
HETATM 149 RU149 RES A 444 6.91994 10.10746 3.38206 0 1 0
0.00000

HETATM 150 RU150 RES A 444 9.45737 5.39884 3.37508 0 1 0
0.00000
HETATM 151 RU151 RES A 444 6.75576 0.51330 3.10323 0 1 0
0.00000
HETATM 152 RU152 RES A 444 0.84858 9.71462 2.92659 0 1 0
0.00000
HETATM 153 RU153 RES A 444 4.03366 5.49752 3.14307 0 1 0
0.00000
HETATM 154 RU154 RES A 444 1.23926 1.10787 7.58902 0 1 0
0.00000
HETATM 155 RU155 RES A 444 12.20595 10.28056 7.39462 0 1 0
0.00000
HETATM 156 RU156 RES A 444 14.82165 5.44501 7.50659 0 1 0
0.00000
HETATM 157 RU157 RES A 444 12.28180 0.85712 7.74318 0 1 0
0.00000
HETATM 158 RU158 RES A 444 6.81779 10.03954 7.78207 0 1 0
0.00000
HETATM 159 RU159 RES A 444 9.46525 5.31256 7.36923 0 1 0
0.00000
HETATM 160 RU160 RES A 444 6.76466 0.84360 7.41254 0 1 0
0.00000
HETATM 161 RU161 RES A 444 1.57521 10.22726 7.29353 0 1 0
0.00000
HETATM 162 RU162 RES A 444 4.05990 5.01877 7.61715 0 1 0
0.00000
HETATM 163 RU163 RES A 444 1.08528 0.78070 11.82524 0 1 0
0.00000
HETATM 164 RU164 RES A 444 12.15652 10.62886 11.51975 0 1 0
0.00000
HETATM 165 RU165 RES A 444 14.92151 5.75726 11.84103 0 1 0
0.00000
HETATM 166 RU166 RES A 444 12.24376 0.90589 11.67975 0 1 0
0.00000
HETATM 167 RU167 RES A 444 6.71680 10.19640 11.45525 0 1 0
0.00000
HETATM 168 RU168 RES A 444 9.46468 5.57752 11.77775 0 1 0
0.00000
HETATM 169 RU169 RES A 444 6.65959 1.07207 12.17448 0 1 0
0.00000
HETATM 170 RU170 RES A 444 1.42500 10.54515 11.79025 0 1 0
0.00000
HETATM 171 RU171 RES A 444 4.02946 5.59762 11.63727 0 1 0
0.00000
HETATM 172 RU172 RES A 444 1.17925 0.74890 16.19702 0 1 0
0.00000
HETATM 173 RU173 RES A 444 12.17030 10.49742 15.64401 0 1 0
0.00000
HETATM 174 RU174 RES A 444 14.94230 5.95003 16.18254 0 1 0
0.00000
HETATM 175 RU175 RES A 444 12.23200 0.81436 16.12026 0 1 0
0.00000
HETATM 176 RU176 RES A 444 6.96261 9.79759 16.08024 0 1 0
0.00000
HETATM 177 RU177 RES A 444 9.81705 5.87827 16.37902 0 1 0
0.00000
HETATM 178 RU178 RES A 444 7.03223 0.75821 16.24235 0 1 0
0.00000

```
HETATM   179 RU179 RES A   444   1.24134  10.44671  15.83661 0       1 0
0.00000
HETATM   180 RU180 RES A   444   4.24671   5.56068  15.85401 0       1 0
0.00000
HETATM   181 RU181 RES A   444   0.11496   2.94724   3.35691 0       1 0
0.00000
HETATM   182 RU182 RES A   444  10.91293  12.21290   3.23868 0       1 0
0.00000
HETATM   183 RU183 RES A   444  13.35700   7.43535   3.39902 0       1 0
0.00000
HETATM   184 RU184 RES A   444  10.71168   2.61592   3.50436 0       1 0
0.00000
HETATM   185 RU185 RES A   444   5.46302  12.27763   3.26146 0       1 0
0.00000
HETATM   186 RU186 RES A   444   8.33281   7.70989   3.61450 0       1 0
0.00000
HETATM   187 RU187 RES A   444   5.27819   2.60622   3.03889 0       1 0
0.00000
HETATM   188 RU188 RES A   444  16.00856  11.90592   3.22229 0       1 0
0.00000
HETATM   189 RU189 RES A   444   2.64323   7.64862   2.98093 0       1 0
0.00000
HETATM   190 RU190 RES A   444  16.12758   3.04174   7.55770 0       1 0
0.00000
HETATM   191 RU191 RES A   444  10.50034  12.61341   7.68455 0       1 0
0.00000
HETATM   192 RU192 RES A   444  13.62399   7.82438   7.34209 0       1 0
0.00000
HETATM   193 RU193 RES A   444  10.80832   3.09257   7.89302 0       1 0
0.00000
HETATM   194 RU194 RES A   444   5.26479  12.52148   7.45771 0       1 0
0.00000
HETATM   195 RU195 RES A   444   8.27607   7.96624   7.53829 0       1 0
0.00000
HETATM   196 RU196 RES A   444   5.46887   2.99681   7.57131 0       1 0
0.00000
HETATM   197 RU197 RES A   444   0.04147  12.80128   7.44691 0       1 0
0.00000
HETATM   198 RU198 RES A   444   2.75342   7.93501   7.31498 0       1 0
0.00000
HETATM   199 RU199 RES A   444   0.04899   3.50645  11.84299 0       1 0
0.00000
HETATM   200 RU200 RES A   444  10.91485  12.79550  11.62273 0       1 0
0.00000
HETATM   201 RU201 RES A   444  13.34535   8.04567  11.76302 0       1 0
0.00000
HETATM   202 RU202 RES A   444  10.58035   3.30631  12.11911 0       1 0
0.00000
HETATM   203 RU203 RES A   444   5.31667  12.53621  11.81001 0       1 0
0.00000
HETATM   204 RU204 RES A   444   8.24988   7.75357  11.85155 0       1 0
0.00000
HETATM   205 RU205 RES A   444   5.57346   3.10934  11.80309 0       1 0
0.00000
HETATM   206 RU206 RES A   444  16.17601  12.56964  11.68454 0       1 0
0.00000
HETATM   207 RU207 RES A   444   2.74879   8.00152  11.75258 0       1 0
0.00000
```

```
HETATM   208 RU208 RES A   444   0.09497   3.24316  16.00886 0       1 0
0.00000
HETATM   209 RU209 RES A   444  10.77087  12.77427  16.02291 0       1 0
0.00000
HETATM   210 RU210 RES A   444  13.49118   8.36378  16.11029 0       1 0
0.00000
HETATM   211 RU211 RES A   444  10.75023   3.34948  16.14006 0       1 0
0.00000
HETATM   212 RU212 RES A   444   5.24671  12.59322  16.23212 0       1 0
0.00000
HETATM   213 RU213 RES A   444   8.08974   7.55841  16.00078 0       1 0
0.00000
HETATM   214 RU214 RES A   444   5.46547   3.12981  16.22486 0       1 0
0.00000
HETATM   215 RU215 RES A   444   0.16367  12.48155  16.18386 0       1 0
0.00000
HETATM   216 RU216 RES A   444   2.78738   8.06792  15.70643 0       1 0
0.00000
HETATM   217 RU217 RES A   444   4.02238   0.56269   3.00655 0       1 0
0.00000
HETATM   218 RU218 RES A   444  14.58718   9.86673   3.42173 0       1 0
0.00000
HETATM   219 RU219 RES A   444   1.65225   5.34046   2.90258 0       1 0
0.00000
HETATM   220 RU220 RES A   444  14.86751   0.55537   3.46845 0       1 0
0.00000
HETATM   221 RU221 RES A   444   9.53339   9.86127   2.98889 0       1 0
0.00000
HETATM   222 RU222 RES A   444  12.08300   5.13852   3.19033 0       1 0
0.00000
HETATM   223 RU223 RES A   444   9.25298   0.53100   3.62712 0       1 0
0.00000
HETATM   224 RU224 RES A   444   4.14783   9.76608   2.95365 0       1 0
0.00000
HETATM   225 RU225 RES A   444   6.74094   5.30818   3.18152 0       1 0
0.00000
HETATM   226 RU226 RES A   444   4.07355   0.75803   7.42749 0       1 0
0.00000
HETATM   227 RU227 RES A   444  14.86401  10.29980   7.60689 0       1 0
0.00000
HETATM   228 RU228 RES A   444   1.35658   5.61107   7.59885 0       1 0
0.00000
HETATM   229 RU229 RES A   444  14.94360   0.67847   7.46498 0       1 0
0.00000
HETATM   230 RU230 RES A   444   9.30658  10.22971   7.45065 0       1 0
0.00000
HETATM   231 RU231 RES A   444  12.21609   5.37124   7.64947 0       1 0
0.00000
HETATM   232 RU232 RES A   444   9.63083   0.97961   7.57661 0       1 0
0.00000
HETATM   233 RU233 RES A   444   3.92450  10.21599   7.23465 0       1 0
0.00000
HETATM   234 RU234 RES A   444   6.69736   5.56454   7.65330 0       1 0
0.00000
HETATM   235 RU235 RES A   444   4.05350   0.86382  12.00104 0       1 0
0.00000
HETATM   236 RU236 RES A   444  14.74440  10.29194  11.81344 0       1 0
0.00000
```

```
HETATM   237 RU237 RES A   444   1.32025   5.76008  11.83448 0       1 0
0.00000
HETATM   238 RU238 RES A   444  14.75561   1.15489  11.77445 0       1 0
0.00000
HETATM   239 RU239 RES A   444   9.50489  10.04255  11.74194 0       1 0
0.00000
HETATM   240 RU240 RES A   444  12.08941   5.69340  11.94164 0       1 0
0.00000
HETATM   241 RU241 RES A   444   9.34572   0.89165  11.90208 0       1 0
0.00000
HETATM   242 RU242 RES A   444   3.93603  10.49769  11.55741 0       1 0
0.00000
HETATM   243 RU243 RES A   444   6.72203   5.41664  11.70056 0       1 0
0.00000
HETATM   244 RU244 RES A   444   4.08291   0.87940  16.22711 0       1 0
0.00000
HETATM   245 RU245 RES A   444  14.73575  10.55029  15.80678 0       1 0
0.00000
HETATM   246 RU246 RES A   444   1.18987   5.88778  15.73164 0       1 0
0.00000
HETATM   247 RU247 RES A   444  14.92087   0.99941  15.88840 0       1 0
0.00000
HETATM   248 RU248 RES A   444   9.37859  10.23577  16.22557 0       1 0
0.00000
HETATM   249 RU249 RES A   444  12.22718   5.64713  16.27155 0       1 0
0.00000
HETATM   250 RU250 RES A   444   9.50414   0.93848  16.05364 0       1 0
0.00000
HETATM   251 RU251 RES A   444   4.44210  10.32639  15.68809 0       1 0
0.00000
HETATM   252 RU252 RES A   444   6.96949   5.20772  16.31149 0       1 0
0.00000
HETATM   253 RU253 RES A   444   2.63019   2.80376   3.17449 0       1 0
0.00000
HETATM   254 RU254 RES A   444  13.19075  11.95170   3.31644 0       1 0
0.00000
HETATM   255 RU255 RES A   444   0.14753   7.15727   3.15765 0       1 0
0.00000
HETATM   256 RU256 RES A   444  13.21005   2.87281   3.23802 0       1 0
0.00000
HETATM   257 RU257 RES A   444   8.13745  12.32365   3.44845 0       1 0
0.00000
HETATM   258 RU258 RES A   444  10.88238   7.64689   3.36142 0       1 0
0.00000
HETATM   259 RU259 RES A   444   8.14229   2.83171   3.36816 0       1 0
0.00000
HETATM   260 RU260 RES A   444   2.42405  11.89863   2.86301 0       1 0
0.00000
HETATM   261 RU261 RES A   444   5.46379   7.76958   2.82995 0       1 0
0.00000
HETATM   262 RU262 RES A   444   2.87709   2.87012   7.63580 0       1 0
0.00000
HETATM   263 RU263 RES A   444  13.52635  12.61676   7.44223 0       1 0
0.00000
HETATM   264 RU264 RES A   444   0.20968   8.08028   7.59092 0       1 0
0.00000
HETATM   265 RU265 RES A   444  13.61133   3.22997   7.68739 0       1 0
0.00000
```

```
HETATM   266 RU266 RES A   444   8.12586  12.36638   7.41050 0      1 0
0.00000
HETATM   267 RU267 RES A   444  11.07320   7.55428   7.57998 0      1 0
0.00000
HETATM   268 RU268 RES A   444   8.16352   2.96558   7.44641 0      1 0
0.00000
HETATM   269 RU269 RES A   444   2.69289  12.53434   7.54647 0      1 0
0.00000
HETATM   270 RU270 RES A   444   5.43514   7.81884   7.52609 0      1 0
0.00000
HETATM   271 RU271 RES A   444   2.79251   3.56764  11.81664 0      1 0
0.00000
HETATM   272 RU272 RES A   444  13.33765  12.82198  11.68945 0      1 0
0.00000
HETATM   273 RU273 RES A   444   0.05753   8.11518  11.72654 0      1 0
0.00000
HETATM   274 RU274 RES A   444  13.48520   3.49874  11.90750 0      1 0
0.00000
HETATM   275 RU275 RES A   444   8.20784  12.36014  11.91442 0      1 0
0.00000
HETATM   276 RU276 RES A   444  10.73192   7.84791  11.79696 0      1 0
0.00000
HETATM   277 RU277 RES A   444   8.07182   3.12965  11.72148 0      1 0
0.00000
HETATM   278 RU278 RES A   444   2.50251  12.95172  11.56280 0      1 0
0.00000
HETATM   279 RU279 RES A   444   5.21838   8.02240  11.52561 0      1 0
0.00000
HETATM   280 RU280 RES A   444   2.96733   3.26010  15.85835 0      1 0
0.00000
HETATM   281 RU281 RES A   444  13.43324  12.73768  16.16297 0      1 0
0.00000
HETATM   282 RU282 RES A   444  16.02992   8.15134  15.77796 0      1 0
0.00000
HETATM   283 RU283 RES A   444  13.41397   3.08306  16.19224 0      1 0
0.00000
HETATM   284 RU284 RES A   444   7.93628  12.59623  16.02290 0      1 0
0.00000
HETATM   285 RU285 RES A   444  11.09808   8.26363  15.79020 0      1 0
0.00000
HETATM   286 RU286 RES A   444   8.29907   3.00772  16.19821 0      1 0
0.00000
HETATM   287 RU287 RES A   444   2.69700  12.92203  15.92092 0      1 0
0.00000
HETATM   288 RU288 RES A   444   5.65371   7.75608  15.88590 0      1 0
0.00000
UNIT ENERGY   kcal
ENERGY        -59401.98970813954
END

BIOGRF 200
DESCRP md_Ru3
REMARK Created by geo_energy_extract.py EO = -2536.56893032 eV; -
58989.97512372094 kcal/mol
RUTYPE SINGLE POINT
#refdata
C:\Surfdrive\Documents\ReaxFF\vaspsrun_files_for_py\geo_extract\md_M\md_R
u
```

```
CRYSTX    16.22334    14.04983    17.12672    90.00000    90.00000
90.00000
FORMAT ATOM
(a6,1x,i5,1x,a5,1x,a3,1x,a1,1x,a5,3f10.5,1x,a5,i3,i2,1x,f8.5)
HETATM     1 RU1   RES A   444  16.08133   1.74449   1.23096 0      1 0
0.00000
HETATM     2 RU2   RES A   444  11.07048  11.02652   1.00292 0      1 0
0.00000
HETATM     3 RU3   RES A   444  13.82276   6.33750   0.86306 0      1 0
0.00000
HETATM     4 RU4   RES A   444  10.77449   1.58107   1.10350 0      1 0
0.00000
HETATM     5 RU5   RES A   444   5.07977  10.92799   1.16150 0      1 0
0.00000
HETATM     6 RU6   RES A   444   7.58189   6.26377   1.12554 0      1 0
0.00000
HETATM     7 RU7   RES A   444   5.40330   1.66356   0.91283 0      1 0
0.00000
HETATM     8 RU8   RES A   444   0.04385  11.40290   1.06962 0      1 0
0.00000
HETATM     9 RU9   RES A   444   2.62259   6.24539   1.10399 0      1 0
0.00000
HETATM    10 RU10  RES A   444  16.05231   1.84147   5.13815 0      1 0
0.00000
HETATM    11 RU11  RES A   444  10.73230  11.18278   5.11546 0      1 0
0.00000
HETATM    12 RU12  RES A   444  13.36531   6.75064   5.44503 0      1 0
0.00000
HETATM    13 RU13  RES A   444  10.76632   1.92731   5.50173 0      1 0
0.00000
HETATM    14 RU14  RES A   444   5.42276  11.20348   5.66071 0      1 0
0.00000
HETATM    15 RU15  RES A   444   8.10907   6.38380   5.47321 0      1 0
0.00000
HETATM    16 RU16  RES A   444   5.62123   1.68457   5.39456 0      1 0
0.00000
HETATM    17 RU17  RES A   444  16.16196  11.31419   5.75921 0      1 0
0.00000
HETATM    18 RU18  RES A   444   2.76700   6.48877   5.53852 0      1 0
0.00000
HETATM    19 RU19  RES A   444   0.21335   1.36219   9.50205 0      1 0
0.00000
HETATM    20 RU20  RES A   444  11.06637  10.86859   9.62136 0      1 0
0.00000
HETATM    21 RU21  RES A   444  13.66277   6.07555   9.75240 0      1 0
0.00000
HETATM    22 RU22  RES A   444  10.88889   1.52961   9.84235 0      1 0
0.00000
HETATM    23 RU23  RES A   444   4.97075  10.83114   9.80163 0      1 0
0.00000
HETATM    24 RU24  RES A   444   8.13976   6.39175   9.69224 0      1 0
0.00000
HETATM    25 RU25  RES A   444   5.38442   1.55201   9.78974 0      1 0
0.00000
HETATM    26 RU26  RES A   444  16.10765  10.55455  10.01121 0      1 0
0.00000
HETATM    27 RU27  RES A   444   2.95224   6.20463   9.28237 0      1 0
0.00000
```

```
HETATM   28 RU28  RES A  444   0.18641   1.25267  13.64467 0      1 0
0.00000
HETATM   29 RU29  RES A  444  11.04247  10.85919  14.17326 0      1 0
0.00000
HETATM   30 RU30  RES A  444  13.73484   5.95810  13.86176 0      1 0
0.00000
HETATM   31 RU31  RES A  444  10.91990   1.50991  13.90985 0      1 0
0.00000
HETATM   32 RU32  RES A  444   5.19146  10.82773  14.23342 0      1 0
0.00000
HETATM   33 RU33  RES A  444   8.07931   5.90898  13.82477 0      1 0
0.00000
HETATM   34 RU34  RES A  444   5.58742   1.53205  14.05896 0      1 0
0.00000
HETATM   35 RU35  RES A  444  16.06727  10.63970  13.97493 0      1 0
0.00000
HETATM   36 RU36  RES A  444   2.67862   5.93718  13.80612 0      1 0
0.00000
HETATM   37 RU37  RES A  444  14.93240   3.88663   1.00505 0      1 0
0.00000
HETATM   38 RU38  RES A  444   9.37247  13.30321   1.08803 0      1 0
0.00000
HETATM   39 RU39  RES A  444  12.01020   8.20857   1.01276 0      1 0
0.00000
HETATM   40 RU40  RES A  444   9.43336   3.88685   0.79100 0      1 0
0.00000
HETATM   41 RU41  RES A  444   4.34447  13.50974   1.30493 0      1 0
0.00000
HETATM   42 RU42  RES A  444   6.63084   8.74352   1.52869 0      1 0
0.00000
HETATM   43 RU43  RES A  444   4.11740   4.23560   1.35115 0      1 0
0.00000
HETATM   44 RU44  RES A  444  14.75133  13.36439   1.19577 0      1 0
0.00000
HETATM   45 RU45  RES A  444   1.01984   8.43631   1.14935 0      1 0
0.00000
HETATM   46 RU46  RES A  444  14.71134   4.02176   5.38169 0      1 0
0.00000
HETATM   47 RU47  RES A  444   9.75584  13.44942   5.20473 0      1 0
0.00000
HETATM   48 RU48  RES A  444  12.08084   8.73192   5.41262 0      1 0
0.00000
HETATM   49 RU49  RES A  444   9.28686   3.92496   5.20641 0      1 0
0.00000
HETATM   50 RU50  RES A  444   4.11675  13.37133   5.26819 0      1 0
0.00000
HETATM   51 RU51  RES A  444   6.61215   8.86278   5.38952 0      1 0
0.00000
HETATM   52 RU52  RES A  444   3.85885   3.78939   5.30523 0      1 0
0.00000
HETATM   53 RU53  RES A  444  14.88997  13.54050   5.48952 0      1 0
0.00000
HETATM   54 RU54  RES A  444   1.08179   8.81821   5.46385 0      1 0
0.00000
HETATM   55 RU55  RES A  444  14.74975   3.58604   9.77469 0      1 0
0.00000
HETATM   56 RU56  RES A  444   9.61512  13.31139   9.39994 0      1 0
0.00000
```

```
HETATM    57 RU57  RES A   444  12.20710   8.52040   9.51980 0       1 0
0.00000
HETATM    58 RU58  RES A   444   9.40586   3.83747   9.39383 0       1 0
0.00000
HETATM    59 RU59  RES A   444   4.06150  12.99374   9.66782 0       1 0
0.00000
HETATM    60 RU60  RES A   444   6.67171   8.50219   9.75988 0       1 0
0.00000
HETATM    61 RU61  RES A   444   4.02543   3.48126   9.55868 0       1 0
0.00000
HETATM    62 RU62  RES A   444  14.96501  13.06374   9.82877 0       1 0
0.00000
HETATM    63 RU63  RES A   444   1.37136   8.42497   9.86658 0       1 0
0.00000
HETATM    64 RU64  RES A   444  14.94434   3.62186  13.76189 0       1 0
0.00000
HETATM    65 RU65  RES A   444   9.47059  13.00563  14.04482 0       1 0
0.00000
HETATM    66 RU66  RES A   444  12.24488   8.26378  13.96600 0       1 0
0.00000
HETATM    67 RU67  RES A   444   9.42183   3.76736  13.61032 0       1 0
0.00000
HETATM    68 RU68  RES A   444   4.18281  13.05667  13.90056 0       1 0
0.00000
HETATM    69 RU69  RES A   444   6.69070   8.40445  13.79764 0       1 0
0.00000
HETATM    70 RU70  RES A   444   4.08253   3.88500  13.90564 0       1 0
0.00000
HETATM    71 RU71  RES A   444  14.88585  13.19189  13.80676 0       1 0
0.00000
HETATM    72 RU72  RES A   444   1.64725   8.32087  14.17139 0       1 0
0.00000
HETATM    73 RU73  RES A   444   2.74613   1.54680   1.00996 0       1 0
0.00000
HETATM    74 RU74  RES A   444  13.64256  11.08093   1.25206 0       1 0
0.00000
HETATM    75 RU75  RES A   444   0.05943   6.23633   1.38768 0       1 0
0.00000
HETATM    76 RU76  RES A   444  13.50867   1.62384   0.89962 0       1 0
0.00000
HETATM    77 RU77  RES A   444   8.25286  11.01436   1.07438 0       1 0
0.00000
HETATM    78 RU78  RES A   444  10.56448   6.13963   0.79601 0       1 0
0.00000
HETATM    79 RU79  RES A   444   8.06164   1.60626   0.92616 0       1 0
0.00000
HETATM    80 RU80  RES A   444   2.80413  11.30271   1.30858 0       1 0
0.00000
HETATM    81 RU81  RES A   444   5.01501   6.38831   1.11524 0       1 0
0.00000
HETATM    82 RU82  RES A   444   2.95148   1.56130   5.55844 0       1 0
0.00000
HETATM    83 RU83  RES A   444  13.25978  11.33065   5.54735 0       1 0
0.00000
HETATM    84 RU84  RES A   444  16.10184   6.41493   5.66235 0       1 0
0.00000
HETATM    85 RU85  RES A   444  13.29290   1.88106   5.26269 0       1 0
0.00000
```

```
HETATM   86 RU86  RES A   444   8.01948  10.98094   5.32745 0       1 0
0.00000
HETATM   87 RU87  RES A   444  10.72341   6.39808   5.12845 0       1 0
0.00000
HETATM   88 RU88  RES A   444   8.02393   1.98235   5.10148 0       1 0
0.00000
HETATM   89 RU89  RES A   444   2.59323  11.33002   5.11226 0       1 0
0.00000
HETATM   90 RU90  RES A   444   5.49432   6.31276   5.89502 0       1 0
0.00000
HETATM   91 RU91  RES A   444   2.79463   1.51682   9.68961 0       1 0
0.00000
HETATM   92 RU92  RES A   444  13.46839  10.62283   9.60761 0       1 0
0.00000
HETATM   93 RU93  RES A   444   0.07925   6.17920   9.41932 0       1 0
0.00000
HETATM   94 RU94  RES A   444  13.47208   1.16737   9.56930 0       1 0
0.00000
HETATM   95 RU95  RES A   444   8.18061  10.78883   9.75897 0       1 0
0.00000
HETATM   96 RU96  RES A   444  11.08749   6.01426   9.50635 0       1 0
0.00000
HETATM   97 RU97  RES A   444   8.30717   1.41346   9.51693 0       1 0
0.00000
HETATM   98 RU98  RES A   444   2.58840  10.57197   9.59180 0       1 0
0.00000
HETATM   99 RU99  RES A   444   5.37377   6.01550   9.55553 0       1 0
0.00000
HETATM  100 RU100 RES A   444   2.76465   1.13624  13.78153 0       1 0
0.00000
HETATM  101 RU101 RES A   444  13.36797  10.45831  14.31213 0       1 0
0.00000
HETATM  102 RU102 RES A   444  16.08470   6.03030  13.79887 0       1 0
0.00000
HETATM  103 RU103 RES A   444  13.62990   1.46796  13.50282 0       1 0
0.00000
HETATM  104 RU104 RES A   444   8.32927  10.87473  14.09408 0       1 0
0.00000
HETATM  105 RU105 RES A   444  10.81000   5.84970  13.96791 0       1 0
0.00000
HETATM  106 RU106 RES A   444   8.28228   1.52288  13.55097 0       1 0
0.00000
HETATM  107 RU107 RES A   444   2.44738  11.04430  14.22375 0       1 0
0.00000
HETATM  108 RU108 RES A   444   5.26152   6.15725  13.87009 0       1 0
0.00000
HETATM  109 RU109 RES A   444   1.49595   3.76566   1.45118 0       1 0
0.00000
HETATM  110 RU110 RES A   444  12.32377  13.12901   0.99935 0       1 0
0.00000
HETATM  111 RU111 RES A   444  14.71810   8.78037   1.43194 0       1 0
0.00000
HETATM  112 RU112 RES A   444  12.18466   3.83334   0.77084 0       1 0
0.00000
HETATM  113 RU113 RES A   444   6.89233  13.60073   0.90415 0       1 0
0.00000
HETATM  114 RU114 RES A   444   9.26563   8.45575   0.76425 0       1 0
0.00000
```

```
HETATM   115 RU115 RES A   444   6.65006   3.70737   0.96615 0       1 0
0.00000
HETATM   116 RU116 RES A   444   1.10695  13.37709   0.79499 0       1 0
0.00000
HETATM   117 RU117 RES A   444   3.93402   8.75890   1.50509 0       1 0
0.00000
HETATM   118 RU118 RES A   444   1.31673   3.97408   5.43569 0       1 0
0.00000
HETATM   119 RU119 RES A   444  12.03190  13.38815   5.43697 0       1 0
0.00000
HETATM   120 RU120 RES A   444  14.95054   8.92338   5.24337 0       1 0
0.00000
HETATM   121 RU121 RES A   444  12.25239   4.29017   5.27034 0       1 0
0.00000
HETATM   122 RU122 RES A   444   6.93948  13.48967   5.23358 0       1 0
0.00000
HETATM   123 RU123 RES A   444   9.40400   8.69160   5.41696 0       1 0
0.00000
HETATM   124 RU124 RES A   444   6.51813   3.95530   5.31236 0       1 0
0.00000
HETATM   125 RU125 RES A   444   1.64631  13.70917   5.46566 0       1 0
0.00000
HETATM   126 RU126 RES A   444   3.97947   9.00561   5.40288 0       1 0
0.00000
HETATM   127 RU127 RES A   444   1.39325   4.07075   9.51495 0       1 0
0.00000
HETATM   128 RU128 RES A   444  12.52288  13.15725   9.29426 0       1 0
0.00000
HETATM   129 RU129 RES A   444  14.72584   8.43822   9.76092 0       1 0
0.00000
HETATM   130 RU130 RES A   444  12.12398   3.92002   9.44398 0       1 0
0.00000
HETATM   131 RU131 RES A   444   6.87803  13.19662   9.49083 0       1 0
0.00000
HETATM   132 RU132 RES A   444   9.64915   8.57588   9.55542 0       1 0
0.00000
HETATM   133 RU133 RES A   444   6.95862   3.65492   9.58524 0       1 0
0.00000
HETATM   134 RU134 RES A   444   1.21290  13.26101   9.68209 0       1 0
0.00000
HETATM   135 RU135 RES A   444   4.14080   8.46891   9.47284 0       1 0
0.00000
HETATM   136 RU136 RES A   444   1.44254   3.38939  13.86610 0       1 0
0.00000
HETATM   137 RU137 RES A   444  12.48121  13.02629  13.81601 0       1 0
0.00000
HETATM   138 RU138 RES A   444  14.93430   8.32367  14.03986 0       1 0
0.00000
HETATM   139 RU139 RES A   444  12.35278   3.80938  13.65063 0       1 0
0.00000
HETATM   140 RU140 RES A   444   6.84741  13.10726  13.60976 0       1 0
0.00000
HETATM   141 RU141 RES A   444   9.40918   8.42825  13.99719 0       1 0
0.00000
HETATM   142 RU142 RES A   444   6.62236   3.93900  13.91243 0       1 0
0.00000
HETATM   143 RU143 RES A   444   1.40380  13.35249  13.93535 0       1 0
0.00000
```

```
HETATM   144 RU144 RES A   444   4.16480   8.55687  13.97410 0      1 0
0.00000
HETATM   145 RU145 RES A   444   1.41144   0.83838   3.29065 0      1 0
0.00000
HETATM   146 RU146 RES A   444  12.14867  10.07749   3.01471 0      1 0
0.00000
HETATM   147 RU147 RES A   444  14.70040   5.66095   3.33557 0      1 0
0.00000
HETATM   148 RU148 RES A   444  12.34025   1.13656   3.11204 0      1 0
0.00000
HETATM   149 RU149 RES A   444   6.74200  10.48153   3.09959 0      1 0
0.00000
HETATM   150 RU150 RES A   444   9.28550   5.47630   2.65179 0      1 0
0.00000
HETATM   151 RU151 RES A   444   6.85627   0.95472   3.17689 0      1 0
0.00000
HETATM   152 RU152 RES A   444   1.33448  10.31010   3.18022 0      1 0
0.00000
HETATM   153 RU153 RES A   444   4.12906   5.42376   3.80430 0      1 0
0.00000
HETATM   154 RU154 RES A   444   1.30136   0.86871   7.25072 0      1 0
0.00000
HETATM   155 RU155 RES A   444  11.96314  10.36810   7.35739 0      1 0
0.00000
HETATM   156 RU156 RES A   444  14.85111   5.62230   7.59631 0      1 0
0.00000
HETATM   157 RU157 RES A   444  12.14680   0.92394   7.25673 0      1 0
0.00000
HETATM   158 RU158 RES A   444   6.62888  10.43315   7.84745 0      1 0
0.00000
HETATM   159 RU159 RES A   444   9.37015   5.77558   7.34304 0      1 0
0.00000
HETATM   160 RU160 RES A   444   6.87692   0.92500   7.65421 0      1 0
0.00000
HETATM   161 RU161 RES A   444   1.09010  10.22851   7.60195 0      1 0
0.00000
HETATM   162 RU162 RES A   444   4.18116   5.43602   7.43553 0      1 0
0.00000
HETATM   163 RU163 RES A   444   1.44487   0.50331  11.42830 0      1 0
0.00000
HETATM   164 RU164 RES A   444  12.03150  10.00520  12.00215 0      1 0
0.00000
HETATM   165 RU165 RES A   444  14.97522   5.19887  11.77990 0      1 0
0.00000
HETATM   166 RU166 RES A   444  12.27783   0.50217  11.39803 0      1 0
0.00000
HETATM   167 RU167 RES A   444   6.63854  10.18112  11.70373 0      1 0
0.00000
HETATM   168 RU168 RES A   444   9.33149   5.28562  11.74503 0      1 0
0.00000
HETATM   169 RU169 RES A   444   6.89675   0.68074  11.81375 0      1 0
0.00000
HETATM   170 RU170 RES A   444   1.27078   9.88986  11.94755 0      1 0
0.00000
HETATM   171 RU171 RES A   444   3.91157   5.28531  11.89672 0      1 0
0.00000
HETATM   172 RU172 RES A   444   1.52118   0.82379  15.78364 0      1 0
0.00000
```

```
HETATM   173 RU173 RES A   444  12.07188  10.01292  16.34934 0       1 0
0.00000
HETATM   174 RU174 RES A   444  15.05967   5.26944  16.08241 0       1 0
0.00000
HETATM   175 RU175 RES A   444  12.11635   0.71929  15.96587 0       1 0
0.00000
HETATM   176 RU176 RES A   444   6.74925   9.99226  16.20186 0       1 0
0.00000
HETATM   177 RU177 RES A   444   9.38997   5.47805  15.88430 0       1 0
0.00000
HETATM   178 RU178 RES A   444   6.94355   0.46550  15.90333 0       1 0
0.00000
HETATM   179 RU179 RES A   444   1.44993  10.00329  16.33594 0       1 0
0.00000
HETATM   180 RU180 RES A   444   3.93968   5.11928  16.18302 0       1 0
0.00000
HETATM   181 RU181 RES A   444  15.99646   3.34306   3.27814 0       1 0
0.00000
HETATM   182 RU182 RES A   444  11.00528  12.38121   2.95364 0       1 0
0.00000
HETATM   183 RU183 RES A   444  13.53936   8.10373   3.25674 0       1 0
0.00000
HETATM   184 RU184 RES A   444  10.90821   3.44191   3.27807 0       1 0
0.00000
HETATM   185 RU185 RES A   444   5.77769  12.59251   3.20258 0       1 0
0.00000
HETATM   186 RU186 RES A   444   7.83453   7.99504   3.50872 0       1 0
0.00000
HETATM   187 RU187 RES A   444   5.22990   3.04939   3.17769 0       1 0
0.00000
HETATM   188 RU188 RES A   444   0.22476  12.72657   3.35636 0       1 0
0.00000
HETATM   189 RU189 RES A   444   2.44087   7.94884   3.30609 0       1 0
0.00000
HETATM   190 RU190 RES A   444  15.99309   3.23778   7.46821 0       1 0
0.00000
HETATM   191 RU191 RES A   444  10.89603  12.82434   7.56729 0       1 0
0.00000
HETATM   192 RU192 RES A   444  13.61734   7.83714   7.56026 0       1 0
0.00000
HETATM   193 RU193 RES A   444  10.65751   3.56594   7.30293 0       1 0
0.00000
HETATM   194 RU194 RES A   444   5.64673  12.59850   7.43645 0       1 0
0.00000
HETATM   195 RU195 RES A   444   8.16331   8.06039   7.49781 0       1 0
0.00000
HETATM   196 RU196 RES A   444   5.44753   3.12767   7.48427 0       1 0
0.00000
HETATM   197 RU197 RES A   444   0.06392  12.82835   7.53746 0       1 0
0.00000
HETATM   198 RU198 RES A   444   2.82703   8.13072   7.45431 0       1 0
0.00000
HETATM   199 RU199 RES A   444   0.16798   2.82689  11.40375 0       1 0
0.00000
HETATM   200 RU200 RES A   444  10.74036  12.20309  12.03111 0       1 0
0.00000
HETATM   201 RU201 RES A   444  13.54477   7.51395  11.78312 0       1 0
0.00000
```

```
HETATM   202 RU202 RES A   444  10.66499   3.08105  11.73545 0       1 0
0.00000
HETATM   203 RU203 RES A   444   5.57545  12.28226  11.88563 0       1 0
0.00000
HETATM   204 RU204 RES A   444   8.08098   8.01575  11.94837 0       1 0
0.00000
HETATM   205 RU205 RES A   444   5.35549   3.10637  11.85343 0       1 0
0.00000
HETATM   206 RU206 RES A   444  16.10469  12.26439  11.79167 0       1 0
0.00000
HETATM   207 RU207 RES A   444   2.78084   7.69136  11.80085 0       1 0
0.00000
HETATM   208 RU208 RES A   444  16.12635   2.97517  15.96791 0       1 0
0.00000
HETATM   209 RU209 RES A   444  10.72430  12.38260  16.33426 0       1 0
0.00000
HETATM   210 RU210 RES A   444  13.33448   7.79410  16.16761 0       1 0
0.00000
HETATM   211 RU211 RES A   444  10.82030   3.29536  15.80544 0       1 0
0.00000
HETATM   212 RU212 RES A   444   5.25306  12.40330  16.44356 0       1 0
0.00000
HETATM   213 RU213 RES A   444   7.98733   7.52837  15.99855 0       1 0
0.00000
HETATM   214 RU214 RES A   444   5.44026   3.12670  15.94496 0       1 0
0.00000
HETATM   215 RU215 RES A   444  16.11793  12.40793  15.90475 0       1 0
0.00000
HETATM   216 RU216 RES A   444   2.64063   7.90591  16.49192 0       1 0
0.00000
HETATM   217 RU217 RES A   444   4.27438   0.68983   3.24742 0       1 0
0.00000
HETATM   218 RU218 RES A   444  15.33129  10.47880   3.29296 0       1 0
0.00000
HETATM   219 RU219 RES A   444   1.24114   5.61178   3.65894 0       1 0
0.00000
HETATM   220 RU220 RES A   444  14.77305   0.96452   3.23060 0       1 0
0.00000
HETATM   221 RU221 RES A   444   9.32818  10.23117   3.18860 0       1 0
0.00000
HETATM   222 RU222 RES A   444  12.36519   5.70978   2.69697 0       1 0
0.00000
HETATM   223 RU223 RES A   444   9.62056   0.98374   3.14912 0       1 0
0.00000
HETATM   224 RU224 RES A   444   4.09172  10.50520   3.28048 0       1 0
0.00000
HETATM   225 RU225 RES A   444   6.73434   5.43855   3.32467 0       1 0
0.00000
HETATM   226 RU226 RES A   444   4.33513   0.94214   7.50945 0       1 0
0.00000
HETATM   227 RU227 RES A   444  14.86238  10.33047   7.68325 0       1 0
0.00000
HETATM   228 RU228 RES A   444   1.39592   5.73022   7.40054 0       1 0
0.00000
HETATM   229 RU229 RES A   444  14.85940   1.05736   7.14678 0       1 0
0.00000
HETATM   230 RU230 RES A   444   9.61494  10.30111   7.48811 0       1 0
0.00000
```

HETATM   231 RU231 RES A   444 12.06734   5.39768   7.20282 0   1 0
0.00000
HETATM   232 RU232 RES A   444   9.77845   0.84871   7.56470 0   1 0
0.00000
HETATM   233 RU233 RES A   444   4.05476 10.50072   7.74646 0   1 0
0.00000
HETATM   234 RU234 RES A   444   7.11530   5.28679   7.75227 0   1 0
0.00000
HETATM   235 RU235 RES A   444   3.89227   0.41114 11.41196 0   1 0
0.00000
HETATM   236 RU236 RES A   444 14.79074   9.81558 12.10454 0   1 0
0.00000
HETATM   237 RU237 RES A   444   1.46783   5.12014 11.87096 0   1 0
0.00000
HETATM   238 RU238 RES A   444 14.96589   0.80785 11.65090 0   1 0
0.00000
HETATM   239 RU239 RES A   444   9.63468 10.03511 11.63814 0   1 0
0.00000
HETATM   240 RU240 RES A   444 12.12159   5.30235 11.75727 0   1 0
0.00000
HETATM   241 RU241 RES A   444   9.18805   0.54713 11.55297 0   1 0
0.00000
HETATM   242 RU242 RES A   444   3.91026 10.19043 11.83435 0   1 0
0.00000
HETATM   243 RU243 RES A   444   6.74071   5.37363 11.59243 0   1 0
0.00000
HETATM   244 RU244 RES A   444   3.97452   0.61714 15.94090 0   1 0
0.00000
HETATM   245 RU245 RES A   444 14.83880 10.23383 16.11819 0   1 0
0.00000
HETATM   246 RU246 RES A   444   1.23991   5.13071 16.05502 0   1 0
0.00000
HETATM   247 RU247 RES A   444 15.31992   0.66467 15.90642 0   1 0
0.00000
HETATM   248 RU248 RES A   444   9.31753 10.10724 16.11968 0   1 0
0.00000
HETATM   249 RU249 RES A   444 12.49471   5.25981 15.87304 0   1 0
0.00000
HETATM   250 RU250 RES A   444   9.33555   1.01009 15.99667 0   1 0
0.00000
HETATM   251 RU251 RES A   444   3.91856 10.07672 16.09458 0   1 0
0.00000
HETATM   252 RU252 RES A   444   6.82945   5.45994 16.13936 0   1 0
0.00000
HETATM   253 RU253 RES A   444   2.65373   2.79243   3.42667 0   1 0
0.00000
HETATM   254 RU254 RES A   444 13.71345 12.66554   3.47826 0   1 0
0.00000
HETATM   255 RU255 RES A   444 16.10476   7.97463   3.15206 0   1 0
0.00000
HETATM   256 RU256 RES A   444 13.32261   3.36163   3.21520 0   1 0
0.00000
HETATM   257 RU257 RES A   444   8.45339 12.50485   3.11542 0   1 0
0.00000
HETATM   258 RU258 RES A   444 10.47824   8.15557   3.20343 0   1 0
0.00000
HETATM   259 RU259 RES A   444   8.09988   3.43794   3.16035 0   1 0
0.00000

HETATM 260 RU260 RES A 444 2.86949 12.87141 3.20053 0 1 0
0.00000
HETATM 261 RU261 RES A 444 5.02572 7.54259 3.57384 0 1 0
0.00000
HETATM 262 RU262 RES A 444 2.63700 3.10943 7.30284 0 1 0
0.00000
HETATM 263 RU263 RES A 444 13.51503 12.55298 7.60996 0 1 0
0.00000
HETATM 264 RU264 RES A 444 16.16983 7.99669 7.70563 0 1 0
0.00000
HETATM 265 RU265 RES A 444 13.40547 3.18398 7.51414 0 1 0
0.00000
HETATM 266 RU266 RES A 444 8.22987 12.86881 7.30299 0 1 0
0.00000
HETATM 267 RU267 RES A 444 10.59501 7.89532 7.34575 0 1 0
0.00000
HETATM 268 RU268 RES A 444 8.10136 2.98430 7.16817 0 1 0
0.00000
HETATM 269 RU269 RES A 444 2.82143 12.69031 7.48356 0 1 0
0.00000
HETATM 270 RU270 RES A 444 5.67952 7.94835 7.50355 0 1 0
0.00000
HETATM 271 RU271 RES A 444 2.86163 2.75964 11.77667 0 1 0
0.00000
HETATM 272 RU272 RES A 444 13.60103 12.20309 11.76549 0 1 0
0.00000
HETATM 273 RU273 RES A 444 16.14987 7.44667 11.68081 0 1 0
0.00000
HETATM 274 RU274 RES A 444 13.38095 2.84238 11.44974 0 1 0
0.00000
HETATM 275 RU275 RES A 444 8.17693 12.32277 11.72966 0 1 0
0.00000
HETATM 276 RU276 RES A 444 10.80794 7.61863 11.65695 0 1 0
0.00000
HETATM 277 RU277 RES A 444 8.23085 3.03403 11.54781 0 1 0
0.00000
HETATM 278 RU278 RES A 444 2.73679 12.60912 11.86485 0 1 0
0.00000
HETATM 279 RU279 RES A 444 5.25974 7.85057 11.87165 0 1 0
0.00000
HETATM 280 RU280 RES A 444 2.89462 2.79426 16.23003 0 1 0
0.00000
HETATM 281 RU281 RES A 444 13.70786 12.31639 16.29454 0 1 0
0.00000
HETATM 282 RU282 RES A 444 16.02131 7.70208 16.02980 0 1 0
0.00000
HETATM 283 RU283 RES A 444 13.60506 3.01118 15.63755 0 1 0
0.00000
HETATM 284 RU284 RES A 444 8.01958 12.36958 15.89439 0 1 0
0.00000
HETATM 285 RU285 RES A 444 10.67554 7.69710 16.12189 0 1 0
0.00000
HETATM 286 RU286 RES A 444 7.96482 3.14569 15.74663 0 1 0
0.00000
HETATM 287 RU287 RES A 444 2.62980 12.68255 16.26228 0 1 0
0.00000
HETATM 288 RU288 RES A 444 5.20068 7.54128 15.91235 0 1 0
0.00000

```
UNIT ENERGY   kcal
ENERGY        -58989.97512372094
END

XTLGRF 200
DESCRP ingaultsRu111hcp001H
RUTYPE NORMAL RUN
#refdata /rf3/training/stack_faults_Ru/fcc111hcp001_H
REMARK Created by geo_energy_extract.py EO = -149.68154762 eV; -
3480.9662237209304 kcal/mol
CRYSTX     2.70391    4.68327   17.12640   90.00000   90.00000   90.00000
HETATM     1 Ru                  2.06733   2.85379   1.02805   Ru1  1 1
0.00000
HETATM     2 Ru                  0.71524   2.05893   3.19981   Ru1  1 1
0.00000
HETATM     3 Ru                  2.06755   4.41612   3.19300   Ru1  1 1
0.00000
HETATM     4 Ru                  0.71577   3.63005   5.33624   Ru1  1 1
0.00000
HETATM     5 Ru                  2.06758   1.28764   5.34806   Ru1  1 1
0.00000
HETATM     6 Ru                  0.71613   0.46779   7.45723   Ru1  1 1
0.00000
HETATM     7 Ru                  2.06866   2.87557   7.44478   Ru1  1 1
0.00000
HETATM     8 Ru                  0.71654   3.67794   9.65331   Ru1  1 1
0.00000
HETATM     9 Ru                  2.06893   1.22316   9.66443   Ru1  1 1
0.00000
HETATM    10 Ru                  0.71508   2.08203  11.80039   Ru1  1 1
0.00000
HETATM    11 Ru                  2.06682   4.37607  11.78765   Ru1  1 1
0.00000
HETATM    12 Ru                  0.71489   0.53056  13.96342   Ru1  1 1
0.00000
HETATM    13 Ru                  2.06749   2.81785  13.96473   Ru1  1 1
0.00000
HETATM    14 Ru                  0.71531   3.63824  16.05969   Ru1  1 1
0.00000
HETATM    15 Ru                  2.06704   1.29346  16.06187   Ru1  1 1
0.00000
HETATM    16 Ru                  0.71552   0.50642   1.03270   Ru1  1 1
0.00000
HETATM    17 H                   0.71734   2.07218   8.57905    H1  1 1
0.00000
UNIT ENERGY   kcal
ENERGY        -3480.9662237209304
END

XTLGRF 200
DESCRP stackfaultsRuhcpslip
RUTYPE NORMAL RUN
#refdata /rf3/training/stack_faults_Ru/hcpslip
REMARK Created by geo_energy_extract.py EO = -143.76766897 eV; -
3343.434162093023 kcal/mol
CRYSTX     2.70391   18.73310    4.28570   90.00000   90.00000   90.00000
HETATM     1 Ru                  2.02791   2.75740   1.07155   Ru1  1 1
0.00000
```

```
HETATM     2 Ru                 0.67607   5.05869   1.07147   Ru1  1 1
0.00000
HETATM     3 Ru                 2.02779   7.30310   1.07139   Ru1  1 1
0.00000
HETATM     4 Ru                 2.02648   9.88312   1.07133   Ru1  1 1
0.00000
HETATM     5 Ru                 0.67590  12.12391   1.07133   Ru1  1 1
0.00000
HETATM     6 Ru                 2.02791  14.42518   1.07139   Ru1  1 1
0.00000
HETATM     7 Ru                 0.67611  16.66959   1.07144   Ru1  1 1
0.00000
HETATM     8 Ru                 0.67589   2.06350   3.21441   Ru1  1 1
0.00000
HETATM     9 Ru                 2.02804   4.30794   3.21435   Ru1  1 1
0.00000
HETATM    10 Ru                 0.67614   6.60923   3.21427   Ru1  1 1
0.00000
HETATM    11 Ru                 2.02943   8.84998   3.21418   Ru1  1 1
0.00000
HETATM    12 Ru                 2.02817  11.43000   3.21417   Ru1  1 1
0.00000
HETATM    13 Ru                 0.67596  13.67444   3.21422   Ru1  1 1
0.00000
HETATM    14 Ru                 2.02788  15.97571   3.21426   Ru1  1 1
0.00000
HETATM    15 Ru                 0.67517  18.21650   3.21436   Ru1  1 1
0.00000
HETATM    16 Ru                 0.67643   0.51659   1.07153   Ru1  1 1
0.00000
UNIT ENERGY   kcal
ENERGY        -3343.434162093023
END

XTLGRF 200
DESCRP tackfaultsRuhcpslipH
RUTYPE NORMAL RUN
#refdata /rf3/training/stack_faults_Ru/hcpslip_H
REMARK Created by geo_energy_extract.py EO = -151.42506100 eV; -
3521.513046511628 kcal/mol
CRYSTX     2.70391   18.73310    4.28570   90.00000   90.00000   90.00000
HETATM     1 Ru                 0.08024   2.67389   1.10080   Ru1  1 1
0.00000
HETATM     2 Ru                 1.43273   5.00732   1.09746   Ru1  1 1
0.00000
HETATM     3 Ru                 0.07977   7.33790   1.10017   Ru1  1 1
0.00000
HETATM     4 Ru                 1.43174   9.66746   1.06966   Ru1  1 1
0.00000
HETATM     5 Ru                 0.07828  12.12166   1.09458   Ru1  1 1
0.00000
HETATM     6 Ru                 1.43095  14.42660   1.10809   Ru1  1 1
0.00000
HETATM     7 Ru                 0.07908  16.75522   1.10401   Ru1  1 1
0.00000
HETATM     8 Ru                 1.43237   1.90650   3.24393   Ru1  1 1
0.00000
```

```
HETATM     9 Ru                   0.08079   4.23417   3.24258   Ru1  1 1
0.00000
HETATM    10 Ru                   1.43202   6.56296   3.24018   Ru1  1 1
0.00000
HETATM    11 Ru                   0.07971   8.86830   3.25474   Ru1  1 1
0.00000
HETATM    12 Ru                   1.43078  11.32205   3.27973   Ru1  1 1
0.00000
HETATM    13 Ru                   0.07834  13.65180   3.24853   Ru1  1 1
0.00000
HETATM    14 Ru                   1.43094  15.98248   3.24950   Ru1  1 1
0.00000
HETATM    15 Ru                   0.07994  18.31573   3.24515   Ru1  1 1
0.00000
HETATM    16 Ru                   1.43202   0.35078   1.10159   Ru1  1 1
0.00000
HETATM    17 H                    0.07972  10.49440   2.17265    H1  1 1
0.00000
UNIT ENERGY   kcal
ENERGY        -3521.513046511628
END

XTLGRF 200
DESCRP ingaultsRuigma7twist
RUTYPE NORMAL RUN
#refdata /rf3/training/stack_faults_Ru/sigma7twist
REMARK Created by geo_energy_extract.py EO = -1029.51740955 eV; -
23942.265338372094 kcal/mol
CRYSTX     7.22044    7.22044   34.78901   90.00000   90.00000   60.00000
HETATM     1 Ru                   4.16872   7.22043   1.01502   Ru1  1 1
0.00000
HETATM     2 Ru                   4.16872   7.22043   5.25257   Ru1  1 1
0.00000
HETATM     3 Ru                   4.16872   7.22044   9.56288   Ru1  1 1
0.00000
HETATM     4 Ru                   4.16872   7.22043  13.89652   Ru1  1 1
0.00000
HETATM     5 Ru                   3.28334   4.62815   0.97742   Ru1  1 1
0.00000
HETATM     6 Ru                   3.27795   4.63696   5.23727   Ru1  1 1
0.00000
HETATM     7 Ru                   3.27530   4.64313   9.56195   Ru1  1 1
0.00000
HETATM     8 Ru                   3.27921   4.64938  13.88084   Ru1  1 1
0.00000
HETATM     9 Ru                   2.36642   2.06291   0.97742   Ru1  1 1
0.00000
HETATM    10 Ru                   2.37675   2.06315   5.23727   Ru1  1 1
0.00000
HETATM    11 Ru                   2.38342   2.06238   9.56195   Ru1  1 1
0.00000
HETATM    12 Ru                   2.38687   2.05587  13.88084   Ru1  1 1
0.00000
HETATM    13 Ru                   1.48319   6.63717   0.94487   Ru1  1 1
0.00000
HETATM    14 Ru                   1.48924   6.69887   5.24209   Ru1  1 1
0.00000
```

HETATM 15 Ru 1.49111 6.70399 9.55214 Ru1 1 1
0.00000
HETATM 16 Ru 1.47120 6.69619 13.87163 Ru1 1 1
0.00000
HETATM 17 Ru 0.60332 4.13960 0.97742 Ru1 1 1
0.00000
HETATM 18 Ru 0.59837 4.13053 5.23727 Ru1 1 1
0.00000
HETATM 19 Ru 0.59437 4.12515 9.56195 Ru1 1 1
0.00000
HETATM 20 Ru 0.58700 4.12541 13.88084 Ru1 1 1
0.00000
HETATM 21 Ru 6.01661 5.18632 0.94487 Ru1 1 1
0.00000
HETATM 22 Ru 5.96015 5.16072 5.24209 Ru1 1 1
0.00000
HETATM 23 Ru 5.95478 5.15977 9.55213 Ru1 1 1
0.00000
HETATM 24 Ru 5.97149 5.14644 13.87163 Ru1 1 1
0.00000
HETATM 25 Ru 5.00636 9.83781 0.94487 Ru1 1 1
0.00000
HETATM 26 Ru 5.05677 9.80172 5.24209 Ru1 1 1
0.00000
HETATM 27 Ru 5.06027 9.79755 9.55214 Ru1 1 1
0.00000
HETATM 28 Ru 5.06348 9.81868 13.87163 Ru1 1 1
0.00000
HETATM 29 Ru 3.86619 8.77477 3.08937 Ru1 1 1
0.00000
HETATM 30 Ru 3.86967 8.76828 7.40830 Ru1 1 1
0.00000
HETATM 31 Ru 3.87634 8.76750 11.73301 Ru1 1 1
0.00000
HETATM 32 Ru 3.88666 8.76775 15.99277 Ru1 1 1
0.00000
HETATM 33 Ru 2.97388 6.18127 3.08937 Ru1 1 1
0.00000
HETATM 34 Ru 2.97778 6.18752 7.40830 Ru1 1 1
0.00000
HETATM 35 Ru 2.97512 6.19369 11.73301 Ru1 1 1
0.00000
HETATM 36 Ru 2.96974 6.20251 15.99277 Ru1 1 1
0.00000
HETATM 37 Ru 2.08436 3.61022 3.07369 Ru1 1 1
0.00000
HETATM 38 Ru 2.08436 3.61022 7.40739 Ru1 1 1
0.00000
HETATM 39 Ru 2.08436 3.61022 11.71771 Ru1 1 1
0.00000
HETATM 40 Ru 2.08436 3.61022 15.95522 Ru1 1 1
0.00000
HETATM 41 Ru 1.18960 1.01198 3.09860 Ru1 1 1
0.00000
HETATM 42 Ru 1.19282 1.03311 7.41809 Ru1 1 1
0.00000
HETATM 43 Ru 1.19631 1.02893 11.72820 Ru1 1 1
0.00000

```
HETATM    44 Ru                   1.24672   0.99282  16.02537   Ru1  1 1
0.00000
HETATM    45 Ru                   0.28160   5.68422   3.09860   Ru1  1 1
0.00000
HETATM    46 Ru                   0.29829   5.67087   7.41809   Ru1  1 1
0.00000
HETATM    47 Ru                   0.29293   5.66993  11.72820   Ru1  1 1
0.00000
HETATM    48 Ru                   0.23645   5.64433  16.02537   Ru1  1 1
0.00000
HETATM    49 Ru                   5.66608   6.70526   3.08937   Ru1  1 1
0.00000
HETATM    50 Ru                   5.65872   6.70551   7.40830   Ru1  1 1
0.00000
HETATM    51 Ru                   5.65472   6.70012  11.73301   Ru1  1 1
0.00000
HETATM    52 Ru                   5.64977   6.69106  15.99277   Ru1  1 1
0.00000
HETATM    53 Ru                   4.78188   4.13445   3.09860   Ru1  1 1
0.00000
HETATM    54 Ru                   4.76197   4.12667   7.41809   Ru1  1 1
0.00000
HETATM    55 Ru                   4.76384   4.13179  11.72820   Ru1  1 1
0.00000
HETATM    56 Ru                   4.76991   4.19350  16.02537   Ru1  1 1
0.00000
HETATM    57 Ru                   3.88666   5.67313  18.37193   Ru1  1 1
0.00000
HETATM    58 Ru                   3.87633   5.67338  22.63178   Ru1  1 1
0.00000
HETATM    59 Ru                   3.86966   5.67259  26.95645   Ru1  1 1
0.00000
HETATM    60 Ru                   3.86621   5.66610  31.27535   Ru1  1 1
0.00000
HETATM    61 Ru                   2.08436   3.61022  18.40952   Ru1  1 1
0.00000
HETATM    62 Ru                   2.08436   3.61022  22.64708   Ru1  1 1
0.00000
HETATM    63 Ru                   2.08436   3.61022  26.95737   Ru1  1 1
0.00000
HETATM    64 Ru                   2.08436   3.61022  31.29104   Ru1  1 1
0.00000
HETATM    65 Ru                   0.23646   1.57611  18.33938   Ru1  1 1
0.00000
HETATM    66 Ru                   0.29293   1.55050  22.63660   Ru1  1 1
0.00000
HETATM    67 Ru                   0.29829   1.54956  26.94663   Ru1  1 1
0.00000
HETATM    68 Ru                   0.28159   1.53622  31.26614   Ru1  1 1
0.00000
HETATM    69 Ru                   4.76990   3.02695  18.33938   Ru1  1 1
0.00000
HETATM    70 Ru                   4.76384   3.08865  22.63660   Ru1  1 1
0.00000
HETATM    71 Ru                   4.76198   3.09377  26.94663   Ru1  1 1
0.00000
HETATM    72 Ru                   4.78188   3.08597  31.26614   Ru1  1 1
0.00000
```

HETATM   73 Ru   2.96974   8.23836 18.37193   Ru1 1 1
0.00000
HETATM   74 Ru   2.97513   8.24719 22.63178   Ru1 1 1
0.00000
HETATM   75 Ru   2.97778   8.25335 26.95645   Ru1 1 1
0.00000
HETATM   76 Ru   2.97388   8.25959 31.27535   Ru1 1 1
0.00000
HETATM   77 Ru   1.24671   6.22759 18.33939   Ru1 1 1
0.00000
HETATM   78 Ru   1.19631   6.19150 22.63660   Ru1 1 1
0.00000
HETATM   79 Ru   1.19281   6.18733 26.94663   Ru1 1 1
0.00000
HETATM   80 Ru   1.18961   6.20846 31.26614   Ru1 1 1
0.00000
HETATM   81 Ru   5.64976   7.74982 18.37193   Ru1 1 1
0.00000
HETATM   82 Ru   5.65471   7.74075 22.63178   Ru1 1 1
0.00000
HETATM   83 Ru   5.65872   7.73536 26.95645   Ru1 1 1
0.00000
HETATM   84 Ru   5.66608   7.73562 31.27535   Ru1 1 1
0.00000
HETATM   85 Ru   4.16872   7.22044 20.46820   Ru1 1 1
0.00000
HETATM   86 Ru   4.16872   7.22044 24.80189   Ru1 1 1
0.00000
HETATM   87 Ru   4.16872   7.22043 29.11222   Ru1 1 1
0.00000
HETATM   88 Ru   4.16872   7.22044 33.34973   Ru1 1 1
0.00000
HETATM   89 Ru   2.38688   5.16456 20.48388   Ru1 1 1
0.00000
HETATM   90 Ru   2.38342   5.15806 24.80280   Ru1 1 1
0.00000
HETATM   91 Ru   2.37674   5.15729 29.12752   Ru1 1 1
0.00000
HETATM   92 Ru   2.36642   5.15753 33.38728   Ru1 1 1
0.00000
HETATM   93 Ru   0.58700   3.09503 20.48388   Ru1 1 1
0.00000
HETATM   94 Ru   0.59436   3.09529 24.80280   Ru1 1 1
0.00000
HETATM   95 Ru   0.59837   3.08989 29.12752   Ru1 1 1
0.00000
HETATM   96 Ru   0.60332   3.08083 33.38728   Ru1 1 1
0.00000
HETATM   97 Ru   5.06348   4.62220 20.49311   Ru1 1 1
0.00000
HETATM   98 Ru   5.06026   4.64333 24.81259   Ru1 1 1
0.00000
HETATM   99 Ru   5.05676   4.63915 29.12271   Ru1 1 1
0.00000
HETATM   100 Ru   5.00636   4.60304 33.41988   Ru1 1 1
0.00000
HETATM   101 Ru   3.27920   2.57106 20.48388   Ru1 1 1
0.00000

```
HETATM   102 Ru                  3.27530   2.57731  24.80280   Ru1  1 1
0.00000
HETATM   103 Ru                  3.27797   2.58347  29.12752   Ru1  1 1
0.00000
HETATM   104 Ru                  3.28334   2.59229  33.38728   Ru1  1 1
0.00000
HETATM   105 Ru                  1.47119   7.74468  20.49311   Ru1  1 1
0.00000
HETATM   106 Ru                  1.49111   7.73689  24.81259   Ru1  1 1
0.00000
HETATM   107 Ru                  1.48924   7.74200  29.12271   Ru1  1 1
0.00000
HETATM   108 Ru                  1.48317   7.80371  33.41988   Ru1  1 1
0.00000
HETATM   109 Ru                  5.97149   9.29444  20.49311   Ru1  1 1
0.00000
HETATM   110 Ru                  5.95479   9.28108  24.81259   Ru1  1 1
0.00000
HETATM   111 Ru                  5.96015   9.28015  29.12271   Ru1  1 1
0.00000
HETATM   112 Ru                  6.01663   9.25455  33.41988   Ru1  1 1
0.00000
UNIT ENERGY   kcal
ENERGY        -23942.265338372094
END

XTLGRF 200
DESCRP ingaultsRugma7twistH
RUTYPE NORMAL RUN
#refdata /rf3/training/stack_faults_Ru/sigma7twist_H
REMARK Created by geo_energy_extract.py EO = -1032.88674635 eV; -
24020.622008139537 kcal/mol
CRYSTX     7.22044    7.22044   34.78901   90.00000   90.00000   60.00000
HETATM     1 Ru                  4.02017   7.32108   1.01005   Ru1  1 1
0.00000
HETATM     2 Ru                  4.04799   7.30015   5.24567   Ru1  1 1
0.00000
HETATM     3 Ru                  4.05846   7.29354   9.55175   Ru1  1 1
0.00000
HETATM     4 Ru                  4.06502   7.29604  13.88726   Ru1  1 1
0.00000
HETATM     5 Ru                  3.18203   4.70044   0.99439   Ru1  1 1
0.00000
HETATM     6 Ru                  3.16475   4.71457   5.23961   Ru1  1 1
0.00000
HETATM     7 Ru                  3.16585   4.71763   9.55190   Ru1  1 1
0.00000
HETATM     8 Ru                  3.17815   4.72563  13.86408   Ru1  1 1
0.00000
HETATM     9 Ru                  2.22175   2.15650   0.95121   Ru1  1 1
0.00000
HETATM    10 Ru                  2.26134   2.14033   5.22760   Ru1  1 1
0.00000
HETATM    11 Ru                  2.27283   2.13635   9.55225   Ru1  1 1
0.00000
HETATM    12 Ru                  2.28477   2.12709  13.85790   Ru1  1 1
0.00000
```

```
HETATM    13 Ru                    1.36472   6.71641   0.94768   Ru1  1 1
0.00000
HETATM    14 Ru                    1.37108   6.77679   5.23674   Ru1  1 1
0.00000
HETATM    15 Ru                    1.38109   6.77910   9.54218   Ru1  1 1
0.00000
HETATM    16 Ru                    1.37130   6.76279  13.86165   Ru1  1 1
0.00000
HETATM    17 Ru                    0.46591   4.18956   0.97006   Ru1  1 1
0.00000
HETATM    18 Ru                    0.48257   4.20437   5.22818   Ru1  1 1
0.00000
HETATM    19 Ru                    0.48595   4.19897   9.54879   Ru1  1 1
0.00000
HETATM    20 Ru                    0.46754   4.20026  13.86092   Ru1  1 1
0.00000
HETATM    21 Ru                    5.88679   5.27476   0.98052   Ru1  1 1
0.00000
HETATM    22 Ru                    5.84531   5.23980   5.23725   Ru1  1 1
0.00000
HETATM    23 Ru                    5.84512   5.23375   9.54835   Ru1  1 1
0.00000
HETATM    24 Ru                    5.86363   5.21085  13.87030   Ru1  1 1
0.00000
HETATM    25 Ru                    4.91787   9.91606   0.90784   Ru1  1 1
0.00000
HETATM    26 Ru                    4.94323   9.87748   5.23472   Ru1  1 1
0.00000
HETATM    27 Ru                    4.95084   9.87298   9.53396   Ru1  1 1
0.00000
HETATM    28 Ru                    4.96115   9.88259  13.84536   Ru1  1 1
0.00000
HETATM    29 Ru                    3.75212   8.86332   3.09137   Ru1  1 1
0.00000
HETATM    30 Ru                    3.75678   8.84370   7.40376   Ru1  1 1
0.00000
HETATM    31 Ru                    3.77038   8.83981  11.71676   Ru1  1 1
0.00000
HETATM    32 Ru                    3.76396   8.86242  15.98064   Ru1  1 1
0.00000
HETATM    33 Ru                    2.85233   6.25856   3.08429   Ru1  1 1
0.00000
HETATM    34 Ru                    2.86510   6.26439   7.39872   Ru1  1 1
0.00000
HETATM    35 Ru                    2.87072   6.26607  11.72388   Ru1  1 1
0.00000
HETATM    36 Ru                    2.89540   6.28418  16.00042   Ru1  1 1
0.00000
HETATM    37 Ru                    1.96869   3.68820   3.07131   Ru1  1 1
0.00000
HETATM    38 Ru                    1.97062   3.68524   7.39789   Ru1  1 1
0.00000
HETATM    39 Ru                    1.97377   3.68484  11.70643   Ru1  1 1
0.00000
HETATM    40 Ru                    1.98512   3.67737  15.89995   Ru1  1 1
0.00000
HETATM    41 Ru                    1.07597   1.09013   3.07833   Ru1  1 1
0.00000
```

HETATM 42 Ru 1.07998 1.10934 7.40281 Ru1 1 1
0.00000
HETATM 43 Ru 1.08850 1.09975 11.71119 Ru1 1 1
0.00000
HETATM 44 Ru 1.14186 1.07268 15.98053 Ru1 1 1
0.00000
HETATM 45 Ru 0.16460 5.75652 3.10684 Ru1 1 1
0.00000
HETATM 46 Ru 0.18610 5.74672 7.41556 Ru1 1 1
0.00000
HETATM 47 Ru 0.18766 5.74365 11.71712 Ru1 1 1
0.00000
HETATM 48 Ru 0.13301 5.72982 16.02380 Ru1 1 1
0.00000
HETATM 49 Ru 5.53453 6.79527 3.08282 Ru1 1 1
0.00000
HETATM 50 Ru 5.54638 6.78045 7.39800 Ru1 1 1
0.00000
HETATM 51 Ru 5.54634 6.77086 11.71702 Ru1 1 1
0.00000
HETATM 52 Ru 5.51734 6.72734 15.97783 Ru1 1 1
0.00000
HETATM 53 Ru 4.66560 4.20220 3.09997 Ru1 1 1
0.00000
HETATM 54 Ru 4.65031 4.20307 7.40789 Ru1 1 1
0.00000
HETATM 55 Ru 4.65591 4.20358 11.71827 Ru1 1 1
0.00000
HETATM 56 Ru 4.66137 4.24885 16.01270 Ru1 1 1
0.00000
HETATM 57 Ru 3.99428 5.62099 18.35702 Ru1 1 1
0.00000
HETATM 58 Ru 3.98981 5.60125 22.63907 Ru1 1 1
0.00000
HETATM 59 Ru 3.99326 5.59628 26.96542 Ru1 1 1
0.00000
HETATM 60 Ru 3.99450 5.58107 31.28021 Ru1 1 1
0.00000
HETATM 61 Ru 2.23412 3.51293 18.43617 Ru1 1 1
0.00000
HETATM 62 Ru 2.20583 3.53406 22.65871 Ru1 1 1
0.00000
HETATM 63 Ru 2.20690 3.53211 26.96694 Ru1 1 1
0.00000
HETATM 64 Ru 2.21076 3.53057 31.29371 Ru1 1 1
0.00000
HETATM 65 Ru 0.35174 1.50473 18.38213 Ru1 1 1
0.00000
HETATM 66 Ru 0.40880 1.47786 22.65365 Ru1 1 1
0.00000
HETATM 67 Ru 0.42123 1.47239 26.96190 Ru1 1 1
0.00000
HETATM 68 Ru 0.40660 1.46047 31.28629 Ru1 1 1
0.00000
HETATM 69 Ru 4.87430 2.97940 18.31451 Ru1 1 1
0.00000
HETATM 70 Ru 4.88143 3.01966 22.64802 Ru1 1 1
0.00000

HETATM 71 Ru 4.88531 3.01741 26.94938 Ru1 1 1
0.00000
HETATM 72 Ru 4.90327 3.00455 31.25810 Ru1 1 1
0.00000
HETATM 73 Ru 3.07331 8.13552 18.38496 Ru1 1 1
0.00000
HETATM 74 Ru 3.09088 8.17316 22.64927 Ru1 1 1
0.00000
HETATM 75 Ru 3.09943 8.17563 26.96750 Ru1 1 1
0.00000
HETATM 76 Ru 3.11867 8.17334 31.28203 Ru1 1 1
0.00000
HETATM 77 Ru 1.35372 6.18083 18.37646 Ru1 1 1
0.00000
HETATM 78 Ru 1.31409 6.11693 22.64706 Ru1 1 1
0.00000
HETATM 79 Ru 1.31522 6.11124 26.95877 Ru1 1 1
0.00000
HETATM 80 Ru 1.30689 6.12500 31.26717 Ru1 1 1
0.00000
HETATM 81 Ru 5.74401 7.67366 18.41015 Ru1 1 1
0.00000
HETATM 82 Ru 5.77318 7.66855 22.64859 Ru1 1 1
0.00000
HETATM 83 Ru 5.78114 7.65819 26.96174 Ru1 1 1
0.00000
HETATM 84 Ru 5.79968 7.66360 31.27547 Ru1 1 1
0.00000
HETATM 85 Ru 4.28128 7.14893 20.48883 Ru1 1 1
0.00000
HETATM 86 Ru 4.28814 7.14446 24.81344 Ru1 1 1
0.00000
HETATM 87 Ru 4.29810 7.13825 29.11987 Ru1 1 1
0.00000
HETATM 88 Ru 4.32973 7.12161 33.35560 Ru1 1 1
0.00000
HETATM 89 Ru 2.50708 5.08888 20.49272 Ru1 1 1
0.00000
HETATM 90 Ru 2.50297 5.08343 24.81242 Ru1 1 1
0.00000
HETATM 91 Ru 2.50098 5.08062 29.12561 Ru1 1 1
0.00000
HETATM 92 Ru 2.47819 5.08559 33.37025 Ru1 1 1
0.00000
HETATM 93 Ru 0.70470 3.02447 20.49914 Ru1 1 1
0.00000
HETATM 94 Ru 0.71516 3.01975 24.81278 Ru1 1 1
0.00000
HETATM 95 Ru 0.72294 3.01208 29.13826 Ru1 1 1
0.00000
HETATM 96 Ru 0.75615 2.98730 33.41571 Ru1 1 1
0.00000
HETATM 97 Ru 5.16953 4.55208 20.50527 Ru1 1 1
0.00000
HETATM 98 Ru 5.18063 4.56790 24.82382 Ru1 1 1
0.00000
HETATM 99 Ru 5.18435 4.55897 29.12958 Ru1 1 1
0.00000

```
HETATM   100 Ru                   5.13426   4.52328  33.42256   Ru1  1 1
0.00000
HETATM   101 Ru                   3.40865   2.49309  20.50559   Ru1  1 1
0.00000
HETATM   102 Ru                   3.39463   2.50275  24.81856   Ru1  1 1
0.00000
HETATM   103 Ru                   3.40098   2.50279  29.13584   Ru1  1 1
0.00000
HETATM   104 Ru                   3.39927   2.47949  33.39432   Ru1  1 1
0.00000
HETATM   105 Ru                   1.58302   7.67463  20.49879   Ru1  1 1
0.00000
HETATM   106 Ru                   1.61096   7.66098  24.81620   Ru1  1 1
0.00000
HETATM   107 Ru                   1.61561   7.66566  29.12765   Ru1  1 1
0.00000
HETATM   108 Ru                   1.62585   7.71868  33.38305   Ru1  1 1
0.00000
HETATM   109 Ru                   6.07369   9.22824  20.52073   Ru1  1 1
0.00000
HETATM   110 Ru                   6.07598   9.20672  24.82951   Ru1  1 1
0.00000
HETATM   111 Ru                   6.08282   9.20284  29.13062   Ru1  1 1
0.00000
HETATM   112 Ru                   6.12952   9.20044  33.45449   Ru1  1 1
0.00000
HETATM   113 H                    1.05249   4.58335  17.34005    H1  1 1
0.00000
UNIT ENERGY   kcal
ENERGY        -24020.622008139537
END

XTLGRF 200
DESCRP Ruslabrect142rect010
RUTYPE NORMAL RUN
#refdata /rf3/training/Ru_slab/rect/142_rect_010
REMARK Created by geo_energy_extract.py EO = -287.61958658 eV; -
6688.827594883721 kcal/mol
CRYSTX     2.70391   33.73310    8.56336   90.00000   90.00000   90.00000
HETATM     1 Ru                  -0.00001   1.50046   1.07056   Ru1  1 1
0.00000
HETATM     2 Ru                  -0.00001   1.50046   5.35217   Ru1  1 1
0.00000
HETATM     3 Ru                  -0.00001   6.20146   1.07042   Ru1  1 1
0.00000
HETATM     4 Ru                  -0.00001   6.20147   5.35205   Ru1  1 1
0.00000
HETATM     5 Ru                  -0.00002  10.89772   1.07039   Ru1  1 1
0.00000
HETATM     6 Ru                  -0.00002  10.89768   5.35208   Ru1  1 1
0.00000
HETATM     7 Ru                  -0.00003  15.61813   1.07054   Ru1  1 1
0.00000
HETATM     8 Ru                  -0.00003  15.61809   5.35222   Ru1  1 1
0.00000
HETATM     9 Ru                   1.35195   3.83256   1.07042   Ru1  1 1
0.00000
```

```
HETATM    10 Ru                 1.35195   3.83256   5.35214   Ru1  1 1
0.00000
HETATM    11 Ru                 1.35194   8.55511   1.07037   Ru1  1 1
0.00000
HETATM    12 Ru                 1.35194   8.55509   5.35204   Ru1  1 1
0.00000
HETATM    13 Ru                 1.35194  13.24827   1.07047   Ru1  1 1
0.00000
HETATM    14 Ru                 1.35194  13.24831   5.35215   Ru1  1 1
0.00000
HETATM    15 Ru                 1.35194  17.85164   1.07055   Ru1  1 1
0.00000
HETATM    16 Ru                 1.35194  17.85158   5.35223   Ru1  1 1
0.00000
HETATM    17 Ru                 1.35194   0.82035   3.21127   Ru1  1 1
0.00000
HETATM    18 Ru                 1.35194   0.82039   7.49295   Ru1  1 1
0.00000
HETATM    19 Ru                 1.35194   5.42376   3.21118   Ru1  1 1
0.00000
HETATM    20 Ru                 1.35194   5.42371   7.49286   Ru1  1 1
0.00000
HETATM    21 Ru                 1.35193  10.11693   3.21111   Ru1  1 1
0.00000
HETATM    22 Ru                 1.35193  10.11695   7.49280   Ru1  1 1
0.00000
HETATM    23 Ru                 1.35192  14.83950   3.21130   Ru1  1 1
0.00000
HETATM    24 Ru                 1.35192  14.83950   7.49295   Ru1  1 1
0.00000
HETATM    25 Ru                 0.00000   3.05391   3.21127   Ru1  1 1
0.00000
HETATM    26 Ru                 0.00000   3.05396   7.49295   Ru1  1 1
0.00000
HETATM    27 Ru                -0.00001   7.77435   3.21112   Ru1  1 1
0.00000
HETATM    28 Ru                -0.00001   7.77438   7.49279   Ru1  1 1
0.00000
HETATM    29 Ru                -0.00002  12.47057   3.21117   Ru1  1 1
0.00000
HETATM    30 Ru                -0.00002  12.47056   7.49290   Ru1  1 1
0.00000
HETATM    31 Ru                -0.00003  17.17162   3.21130   Ru1  1 1
0.00000
HETATM    32 Ru                -0.00003  17.17162   7.49305   Ru1  1 1
0.00000
UNIT ENERGY   kcal
ENERGY        -6688.827594883721
END

XTLGRF 200
DESCRP Ruslabrect412rect100
RUTYPE NORMAL RUN
#refdata /rf3/training/Ru_slab/rect/412_rect_100
REMARK Created by geo_energy_extract.py EO = -279.16727062 eV; -
6492.262107441861 kcal/mol
CRYSTX    25.81564    4.68327    8.56336   90.00000   90.00000   90.00000
```

```
HETATM    1 Ru                   2.68005   1.52777   1.07040   Ru1  1 1
0.00000
HETATM    2 Ru                   2.68005   1.52777   5.35209   Ru1  1 1
0.00000
HETATM    3 Ru                   5.41040   1.53185   1.07042   Ru1  1 1
0.00000
HETATM    4 Ru                   5.41039   1.53184   5.35210   Ru1  1 1
0.00000
HETATM    5 Ru                   8.13232   1.56410   1.07052   Ru1  1 1
0.00000
HETATM    6 Ru                   8.13232   1.56410   5.35220   Ru1  1 1
0.00000
HETATM    7 Ru                   0.06308   1.52873   1.07049   Ru1  1 1
0.00000
HETATM    8 Ru                   0.06308   1.52873   5.35217   Ru1  1 1
0.00000
HETATM    9 Ru                   1.33137   3.90572   1.07052   Ru1  1 1
0.00000
HETATM   10 Ru                   1.33137   3.90572   5.35220   Ru1  1 1
0.00000
HETATM   11 Ru                   4.05326   3.87348   1.07042   Ru1  1 1
0.00000
HETATM   12 Ru                   4.05326   3.87348   5.35210   Ru1  1 1
0.00000
HETATM   13 Ru                   6.78358   3.86942   1.07041   Ru1  1 1
0.00000
HETATM   14 Ru                   6.78358   3.86941   5.35209   Ru1  1 1
0.00000
HETATM   15 Ru                   9.40056   3.87043   1.07048   Ru1  1 1
0.00000
HETATM   16 Ru                   9.40056   3.87044   5.35217   Ru1  1 1
0.00000
HETATM   17 Ru                   1.33124   0.71650   3.21128   Ru1  1 1
0.00000
HETATM   18 Ru                   1.33123   0.71650   7.49296   Ru1  1 1
0.00000
HETATM   19 Ru                   4.05322   0.74878   3.21118   Ru1  1 1
0.00000
HETATM   20 Ru                   4.05322   0.74878   7.49285   Ru1  1 1
0.00000
HETATM   21 Ru                   6.78350   0.75281   3.21122   Ru1  1 1
0.00000
HETATM   22 Ru                   6.78350   0.75281   7.49289   Ru1  1 1
0.00000
HETATM   23 Ru                   9.40050   0.75173   3.21122   Ru1  1 1
0.00000
HETATM   24 Ru                   9.40050   0.75173   7.49289   Ru1  1 1
0.00000
HETATM   25 Ru                   2.68016   3.09444   3.21121   Ru1  1 1
0.00000
HETATM   26 Ru                   2.68016   3.09444   7.49289   Ru1  1 1
0.00000
HETATM   27 Ru                   5.41042   3.09043   3.21118   Ru1  1 1
0.00000
HETATM   28 Ru                   5.41042   3.09043   7.49286   Ru1  1 1
0.00000
HETATM   29 Ru                   8.13239   3.05817   3.21128   Ru1  1 1
0.00000
```

```
HETATM    30 Ru                    8.13239   3.05817   7.49297   Ru1  1 1
0.00000
HETATM    31 Ru                    0.06316   3.09335   3.21122   Ru1  1 1
0.00000
HETATM    32 Ru                    0.06316   3.09335   7.49290   Ru1  1 1
0.00000
UNIT ENERGY   kcal
ENERGY        -6492.262107441861
END

XTLGRF 200
DESCRP RuadsmultiH12H
RUTYPE NORMAL RUN
#refdata /rf3/training/Ru_ads/multi_H/12H
REMARK Created by geo_energy_extract.py EO = -418.87061634 eV; -
9741.177124186048 kcal/mol
CRYSTX     8.11167    4.68327   28.85554   90.00000   90.00000   90.00000
HETATM     1 Ru                   -0.00120   1.35977   1.14808   Ru1  1 1
0.00000
HETATM     2 Ru                    5.40644   1.35978   1.14811   Ru1  1 1
0.00000
HETATM     3 Ru                    2.70260   1.35975   1.14813   Ru1  1 1
0.00000
HETATM     4 Ru                    6.75844   3.70130   1.14815   Ru1  1 1
0.00000
HETATM     5 Ru                    4.05451   3.70139   1.14819   Ru1  1 1
0.00000
HETATM     6 Ru                    1.35073   3.70147   1.14825   Ru1  1 1
0.00000
HETATM     7 Ru                    1.35078   0.58025   3.20381   Ru1  1 1
0.00000
HETATM     8 Ru                    6.75843   0.58034   3.20372   Ru1  1 1
0.00000
HETATM     9 Ru                    4.05470   0.58037   3.20381   Ru1  1 1
0.00000
HETATM    10 Ru                   -0.00121   2.92199   3.20379   Ru1  1 1
0.00000
HETATM    11 Ru                    5.40658   2.92200   3.20385   Ru1  1 1
0.00000
HETATM    12 Ru                    2.70278   2.92189   3.20379   Ru1  1 1
0.00000
HETATM    13 Ru                   -0.00069   1.36255   5.34512   Ru1  1 1
0.00000
HETATM    14 Ru                    5.40709   1.36237   5.34514   Ru1  1 1
0.00000
HETATM    15 Ru                    2.70311   1.36235   5.34503   Ru1  1 1
0.00000
HETATM    16 Ru                    6.75888   3.70413   5.34521   Ru1  1 1
0.00000
HETATM    17 Ru                    4.05516   3.70394   5.34518   Ru1  1 1
0.00000
HETATM    18 Ru                    1.35118   3.70402   5.34507   Ru1  1 1
0.00000
HETATM    19 Ru                    1.35178   0.58307   7.50620   Ru1  1 1
0.00000
HETATM    20 Ru                    6.75960   0.58303   7.50613   Ru1  1 1
0.00000
```

HETATM	21	Ru	4.05563	0.58291	7.50629	Ru1	1	1	0.00000
HETATM	22	Ru	-0.00011	2.92477	7.50603	Ru1	1	1	0.00000
HETATM	23	Ru	5.40745	2.92456	7.50616	Ru1	1	1	0.00000
HETATM	24	Ru	2.70369	2.92463	7.50618	Ru1	1	1	0.00000
HETATM	25	Ru	-0.00021	1.36394	9.65844	Ru1	1	1	0.00000
HETATM	26	Ru	5.40735	1.36361	9.65868	Ru1	1	1	0.00000
HETATM	27	Ru	2.70364	1.36364	9.65853	Ru1	1	1	0.00000
HETATM	28	Ru	6.75934	3.70543	9.65856	Ru1	1	1	0.00000
HETATM	29	Ru	4.05550	3.70525	9.65866	Ru1	1	1	0.00000
HETATM	30	Ru	1.35163	3.70548	9.65848	Ru1	1	1	0.00000
HETATM	31	Ru	1.35107	0.58192	11.77092	Ru1	1	1	0.00000
HETATM	32	Ru	6.75902	0.58177	11.77085	Ru1	1	1	0.00000
HETATM	33	Ru	4.05514	0.58186	11.77089	Ru1	1	1	0.00000
HETATM	34	Ru	-0.00079	2.92371	11.77103	Ru1	1	1	0.00000
HETATM	35	Ru	5.40714	2.92355	11.77096	Ru1	1	1	0.00000
HETATM	36	Ru	2.70303	2.92344	11.77098	Ru1	1	1	0.00000
HETATM	37	Ru	-0.00070	1.36156	13.91537	Ru1	1	1	0.00000
HETATM	38	Ru	5.40693	1.36114	13.91507	Ru1	1	1	0.00000
HETATM	39	Ru	2.70288	1.36151	13.91531	Ru1	1	1	0.00000
HETATM	40	Ru	6.75900	3.70310	13.91553	Ru1	1	1	0.00000
HETATM	41	Ru	4.05488	3.70274	13.91531	Ru1	1	1	0.00000
HETATM	42	Ru	1.35095	3.70315	13.91546	Ru1	1	1	0.00000
HETATM	43	H	4.05517	3.70155	15.53214	H1	1	1	0.00000
HETATM	44	H	1.35095	3.70176	15.53248	H1	1	1	0.00000
HETATM	45	H	6.75896	3.70092	15.53276	H1	1	1	0.00000
HETATM	46	H	-0.00089	1.36027	15.53222	H1	1	1	0.00000
HETATM	47	H	2.70334	1.35937	15.53203	H1	1	1	0.00000
HETATM	48	H	5.40692	1.35976	15.53177	H1	1	1	0.00000
HETATM	49	H	1.35139	0.58050	14.88979	H1	1	1	0.00000

```
HETATM    50 H                    4.05481   0.57674  14.88827    H1   1 1
0.00000
HETATM    51 H                    6.75981   0.58046  14.88947    H1   1 1
0.00000
HETATM    52 H                    2.70265   2.92211  14.88951    H1   1 1
0.00000
HETATM    53 H                    5.40729   2.91948  14.88794    H1   1 1
0.00000
HETATM    54 H                    0.00000   2.92229  14.88936    H1   1 1
0.00000
UNIT ENERGY   kcal
ENERGY        -9741.177124186048
END

XTLGRF 200
DESCRP RuadsmultiH4H
RUTYPE NORMAL RUN
#refdata /rf3/training/Ru_ads/multi_H/4H
REMARK Created by geo_energy_extract.py EO = -392.11496268 eV; -
9118.952620465117 kcal/mol
CRYSTX     8.11167    4.68327   28.85554   90.00000   90.00000   90.00000
HETATM     1 Ru                   0.00000   1.53380   1.12663   Ru1  1 1
0.00000
HETATM     2 Ru                   5.40841   1.53200   1.12413   Ru1  1 1
0.00000
HETATM     3 Ru                   2.70327   1.53198   1.12417   Ru1  1 1
0.00000
HETATM     4 Ru                   6.75908   3.87271   1.12405   Ru1  1 1
0.00000
HETATM     5 Ru                   4.05585   3.87239   1.12201   Ru1  1 1
0.00000
HETATM     6 Ru                   1.35266   3.87270   1.12406   Ru1  1 1
0.00000
HETATM     7 Ru                   1.35224   0.75026   3.18362   Ru1  1 1
0.00000
HETATM     8 Ru                   6.75949   0.75027   3.18359   Ru1  1 1
0.00000
HETATM     9 Ru                   4.05584   0.74818   3.18190   Ru1  1 1
0.00000
HETATM    10 Ru                  -0.00003   3.09166   3.18281   Ru1  1 1
0.00000
HETATM    11 Ru                   5.40848   3.09138   3.18220   Ru1  1 1
0.00000
HETATM    12 Ru                   2.70321   3.09139   3.18222   Ru1  1 1
0.00000
HETATM    13 Ru                   8.11164   1.52957   5.32968   Ru1  1 1
0.00000
HETATM    14 Ru                   5.40656   1.53188   5.32695   Ru1  1 1
0.00000
HETATM    15 Ru                   2.70510   1.53187   5.32695   Ru1  1 1
0.00000
HETATM    16 Ru                   6.75983   3.86900   5.32629   Ru1  1 1
0.00000
HETATM    17 Ru                   4.05583   3.86918   5.32343   Ru1  1 1
0.00000
HETATM    18 Ru                   1.35180   3.86899   5.32629   Ru1  1 1
0.00000
```

```
HETATM    19 Ru                  1.35131   0.74867   7.48011   Ru1  1 1
0.00000
HETATM    20 Ru                  6.76037   0.74872   7.48011   Ru1  1 1
0.00000
HETATM    21 Ru                  4.05583   0.74796   7.47679   Ru1  1 1
0.00000
HETATM    22 Ru                 -0.00002   3.08859   7.48454   Ru1  1 1
0.00000
HETATM    23 Ru                  5.40875   3.08757   7.48294   Ru1  1 1
0.00000
HETATM    24 Ru                  2.70290   3.08758   7.48293   Ru1  1 1
0.00000
HETATM    25 Ru                 -0.00003   1.53290   9.64163   Ru1  1 1
0.00000
HETATM    26 Ru                  5.40815   1.52895   9.63566   Ru1  1 1
0.00000
HETATM    27 Ru                  2.70349   1.52894   9.63565   Ru1  1 1
0.00000
HETATM    28 Ru                  6.75752   3.86902   9.64247   Ru1  1 1
0.00000
HETATM    29 Ru                  4.05581   3.87043   9.63628   Ru1  1 1
0.00000
HETATM    30 Ru                  1.35407   3.86903   9.64244   Ru1  1 1
0.00000
HETATM    31 Ru                  1.35085   0.74873  11.76485   Ru1  1 1
0.00000
HETATM    32 Ru                  6.76072   0.74875  11.76489   Ru1  1 1
0.00000
HETATM    33 Ru                  4.05578   0.74979  11.75740   Ru1  1 1
0.00000
HETATM    34 Ru                  8.11160   3.09586  11.78146   Ru1  1 1
0.00000
HETATM    35 Ru                  5.40391   3.09055  11.78172   Ru1  1 1
0.00000
HETATM    36 Ru                  2.70774   3.09058  11.78162   Ru1  1 1
0.00000
HETATM    37 Ru                  8.11165   1.51744  13.89740   Ru1  1 1
0.00000
HETATM    38 Ru                  5.39937   1.52353  13.86776   Ru1  1 1
0.00000
HETATM    39 Ru                  2.71213   1.52354  13.86775   Ru1  1 1
0.00000
HETATM    40 Ru                  6.73959   3.87632  13.86871   Ru1  1 1
0.00000
HETATM    41 Ru                  4.05574   3.89574  13.84718   Ru1  1 1
0.00000
HETATM    42 Ru                  1.37197   3.87635  13.86869   Ru1  1 1
0.00000
HETATM    43 H                   4.05564   2.29183  14.92521    H1  1 1
0.00000
HETATM    44 H                   1.36758   2.29887  14.92549    H1  1 1
0.00000
HETATM    45 H                   6.74373   2.29889  14.92553    H1  1 1
0.00000
HETATM    46 H                   8.11147   4.67133  14.93409    H1  1 1
0.00000
UNIT ENERGY   kcal
ENERGY        -9118.952620465117
```

```
END

XTLGRF 200
DESCRP RuadsmultiH6H
RUTYPE NORMAL RUN
#refdata /rf3/training/Ru_ads/multi_H/6H
REMARK Created by geo_energy_extract.py E0 = -399.88317327 eV; -
9299.608680697675 kcal/mol
CRYSTX     8.11167    4.68327   28.85554   90.00000   90.00000   90.00000
HETATM     1 Ru                  0.00001    1.53078    1.16765   Ru1  1 1
0.00000
HETATM     2 Ru                  5.40780    1.53063    1.16776   Ru1  1 1
0.00000
HETATM     3 Ru                  2.70377    1.53065    1.16778   Ru1  1 1
0.00000
HETATM     4 Ru                  6.75965    3.87238    1.16767   Ru1  1 1
0.00000
HETATM     5 Ru                  4.05585    3.87230    1.16770   Ru1  1 1
0.00000
HETATM     6 Ru                  1.35193    3.87240    1.16769   Ru1  1 1
0.00000
HETATM     7 Ru                  1.35188    0.74928    3.22561   Ru1  1 1
0.00000
HETATM     8 Ru                  6.75970    0.74925    3.22561   Ru1  1 1
0.00000
HETATM     9 Ru                  4.05578    0.74927    3.22565   Ru1  1 1
0.00000
HETATM    10 Ru                  8.11158    3.09079    3.22563   Ru1  1 1
0.00000
HETATM    11 Ru                  5.40774    3.09088    3.22561   Ru1  1 1
0.00000
HETATM    12 Ru                  2.70386    3.09086    3.22562   Ru1  1 1
0.00000
HETATM    13 Ru                  8.11157    1.53090    5.37021   Ru1  1 1
0.00000
HETATM    14 Ru                  5.40770    1.53100    5.37022   Ru1  1 1
0.00000
HETATM    15 Ru                  2.70374    1.53096    5.37025   Ru1  1 1
0.00000
HETATM    16 Ru                  6.75960    3.87259    5.37017   Ru1  1 1
0.00000
HETATM    17 Ru                  4.05577    3.87276    5.37033   Ru1  1 1
0.00000
HETATM    18 Ru                  1.35188    3.87253    5.37021   Ru1  1 1
0.00000
HETATM    19 Ru                  1.35179    0.75177    7.52423   Ru1  1 1
0.00000
HETATM    20 Ru                  6.75945    0.75172    7.52425   Ru1  1 1
0.00000
HETATM    21 Ru                  4.05563    0.75194    7.52422   Ru1  1 1
0.00000
HETATM    22 Ru                  8.11145    3.09335    7.52406   Ru1  1 1
0.00000
HETATM    23 Ru                  5.40761    3.09354    7.52428   Ru1  1 1
0.00000
HETATM    24 Ru                  2.70362    3.09353    7.52431   Ru1  1 1
0.00000
```

```
HETATM    25 Ru                     8.11133   1.53310   9.67959   Ru1  1 1
0.00000
HETATM    26 Ru                     5.40756   1.53303   9.67970   Ru1  1 1
0.00000
HETATM    27 Ru                     2.70349   1.53295   9.67962   Ru1  1 1
0.00000
HETATM    28 Ru                     6.75949   3.87462   9.67967   Ru1  1 1
0.00000
HETATM    29 Ru                     4.05549   3.87464   9.67956   Ru1  1 1
0.00000
HETATM    30 Ru                     1.35147   3.87460   9.67959   Ru1  1 1
0.00000
HETATM    31 Ru                     1.35144   0.75297  11.80872   Ru1  1 1
0.00000
HETATM    32 Ru                     6.75937   0.75299  11.80875   Ru1  1 1
0.00000
HETATM    33 Ru                     4.05542   0.75288  11.80858   Ru1  1 1
0.00000
HETATM    34 Ru                     8.11131   3.09448  11.80877   Ru1  1 1
0.00000
HETATM    35 Ru                     5.40736   3.09454  11.80863   Ru1  1 1
0.00000
HETATM    36 Ru                     2.70345   3.09458  11.80861   Ru1  1 1
0.00000
HETATM    37 Ru                     8.11140   1.53440  13.92754   Ru1  1 1
0.00000
HETATM    38 Ru                     5.40759   1.53396  13.92717   Ru1  1 1
0.00000
HETATM    39 Ru                     2.70370   1.53388  13.92722   Ru1  1 1
0.00000
HETATM    40 Ru                     6.75960   3.87584  13.92742   Ru1  1 1
0.00000
HETATM    41 Ru                     4.05571   3.87548  13.92716   Ru1  1 1
0.00000
HETATM    42 Ru                     1.35176   3.87588  13.92751   Ru1  1 1
0.00000
HETATM    43 H                      4.05536   2.31309  14.97234    H1  1 1
0.00000
HETATM    44 H                      1.35200   2.31323  14.97262    H1  1 1
0.00000
HETATM    45 H                      6.75914   2.31329  14.97260    H1  1 1
0.00000
HETATM    46 H                      8.11118   4.65492  14.97280    H1  1 1
0.00000
HETATM    47 H                      2.70346   4.65477  14.97235    H1  1 1
0.00000
HETATM    48 H                      5.40734   4.65482  14.97233    H1  1 1
0.00000
UNIT ENERGY   kcal
ENERGY        -9299.608680697675
END

XTLGRF 200
DESCRP RuadsmultiH8H
RUTYPE NORMAL RUN
#refdata /rf3/training/Ru_ads/multi_H/8H
REMARK Created by geo_energy_extract.py EO = -405.80916451 eV; -
9437.422430465118 kcal/mol
```

```
CRYSTX      8.11167    4.68327   28.85554   90.00000   90.00000   90.00000
HETATM     1 Ru                  0.00062    1.53208    1.12504    Ru1  1 1
0.00000
HETATM     2 Ru                  5.40750    1.53184    1.12721    Ru1  1 1
0.00000
HETATM     3 Ru                  2.70430    1.53205    1.12260    Ru1  1 1
0.00000
HETATM     4 Ru                  6.75946    3.87207    1.12455    Ru1  1 1
0.00000
HETATM     5 Ru                  4.05475    3.87308    1.12312    Ru1  1 1
0.00000
HETATM     6 Ru                  1.35289    3.87385    1.12840    Ru1  1 1
0.00000
HETATM     7 Ru                  1.35147    0.74886    3.18307    Ru1  1 1
0.00000
HETATM     8 Ru                  6.75956    0.74964    3.18314    Ru1  1 1
0.00000
HETATM     9 Ru                  4.05622    0.74991    3.18190    Ru1  1 1
0.00000
HETATM    10 Ru                  0.00026    3.09131    3.18323    Ru1  1 1
0.00000
HETATM    11 Ru                  5.40839    3.09050    3.18238    Ru1  1 1
0.00000
HETATM    12 Ru                  2.70364    3.09191    3.18251    Ru1  1 1
0.00000
HETATM    13 Ru                  0.00050    1.52851    5.32560    Ru1  1 1
0.00000
HETATM    14 Ru                  5.40780    1.52926    5.32970    Ru1  1 1
0.00000
HETATM    15 Ru                  2.70431    1.52921    5.32080    Ru1  1 1
0.00000
HETATM    16 Ru                  6.75903    3.87004    5.32519    Ru1  1 1
0.00000
HETATM    17 Ru                  4.05557    3.87093    5.32196    Ru1  1 1
0.00000
HETATM    18 Ru                  1.35201    3.87122    5.33064    Ru1  1 1
0.00000
HETATM    19 Ru                  1.35383    0.74551    7.48490    Ru1  1 1
0.00000
HETATM    20 Ru                  6.75771    0.74948    7.48852    Ru1  1 1
0.00000
HETATM    21 Ru                  4.05615    0.74727    7.48425    Ru1  1 1
0.00000
HETATM    22 Ru                  0.00232    3.09033    7.48626    Ru1  1 1
0.00000
HETATM    23 Ru                  5.40584    3.08713    7.48512    Ru1  1 1
0.00000
HETATM    24 Ru                  2.70380    3.08975    7.48364    Ru1  1 1
0.00000
HETATM    25 Ru                  0.00060    1.52830    9.63206    Ru1  1 1
0.00000
HETATM    26 Ru                  5.40719    1.52923    9.64223    Ru1  1 1
0.00000
HETATM    27 Ru                  2.70422    1.53208    9.63475    Ru1  1 1
0.00000
HETATM    28 Ru                  6.75823    3.86923    9.63203    Ru1  1 1
0.00000
```

```
HETATM    29 Ru                  4.05598   3.87277   9.63612   Ru1  1 1
0.00000
HETATM    30 Ru                  1.35186   3.87053   9.64172   Ru1  1 1
0.00000
HETATM    31 Ru                  1.35767   0.74571  11.75781   Ru1  1 1
0.00000
HETATM    32 Ru                  6.75710   0.73907  11.75581   Ru1  1 1
0.00000
HETATM    33 Ru                  4.05172   0.75187  11.76345   Ru1  1 1
0.00000
HETATM    34 Ru                  0.00608   3.08181  11.75406   Ru1  1 1
0.00000
HETATM    35 Ru                  5.40170   3.08697  11.76341   Ru1  1 1
0.00000
HETATM    36 Ru                  2.70601   3.09663  11.76269   Ru1  1 1
0.00000
HETATM    37 Ru                  8.10448   1.53490  13.86422   Ru1  1 1
0.00000
HETATM    38 Ru                  5.41280   1.53185  13.95604   Ru1  1 1
0.00000
HETATM    39 Ru                  2.67370   1.50667  13.89093   Ru1  1 1
0.00000
HETATM    40 Ru                  6.77197   3.87246  13.85604   Ru1  1 1
0.00000
HETATM    41 Ru                  4.09223   3.85000  13.91132   Ru1  1 1
0.00000
HETATM    42 Ru                  1.34380   3.87074  13.97163   Ru1  1 1
0.00000
HETATM    43 H                   4.78271   2.70814  15.11055    H1  1 1
0.00000
HETATM    44 H                   0.94877   2.50613  15.05163    H1  1 1
0.00000
HETATM    45 H                   6.78875   2.23078  14.93818    H1  1 1
0.00000
HETATM    46 H                   8.07355   4.62068  14.93394    H1  1 1
0.00000
HETATM    47 H                   2.74143   3.15418  14.89810    H1  1 1
0.00000
HETATM    48 H                   5.63778   0.04407  14.98132    H1  1 1
0.00000
HETATM    49 H                   1.94938   0.40153  15.10540    H1  1 1
0.00000
HETATM    50 H                   3.98237   0.81853  14.89388    H1  1 1
0.00000
UNIT ENERGY   kcal
ENERGY        -9437.422430465118
END

XTLGRF 200
DESCRP Ruadsrect142rect010
RUTYPE NORMAL RUN
#refdata /rf3/training/Ru_ads/rect/142_rect_010
REMARK Created by geo_energy_extract.py EO = -291.46940564 eV; -
6778.358270697675 kcal/mol
CRYSTX     2.70391   33.73310    8.56336   90.00000   90.00000   90.00000
HETATM     1 Ru                 -0.00005   1.48455   1.08248   Ru1  1 1
0.00000
```

```
HETATM    2 Ru                 -0.00005   1.48456   5.36379   Ru1  1 1
0.00000
HETATM    3 Ru                 -0.00008   6.18795   1.08699   Ru1  1 1
0.00000
HETATM    4 Ru                 -0.00008   6.18761   5.36743   Ru1  1 1
0.00000
HETATM    5 Ru                 -0.00009  10.88857   1.08918   Ru1  1 1
0.00000
HETATM    6 Ru                 -0.00009  10.88908   5.36713   Ru1  1 1
0.00000
HETATM    7 Ru                 -0.00008  15.60449   1.08159   Ru1  1 1
0.00000
HETATM    8 Ru                 -0.00008  15.61530   5.37904   Ru1  1 1
0.00000
HETATM    9 Ru                  1.35189   3.81763   1.08458   Ru1  1 1
0.00000
HETATM   10 Ru                  1.35189   3.81753   5.36607   Ru1  1 1
0.00000
HETATM   11 Ru                  1.35187   8.54275   1.08634   Ru1  1 1
0.00000
HETATM   12 Ru                  1.35187   8.54220   5.36935   Ru1  1 1
0.00000
HETATM   13 Ru                  1.35187  13.24104   1.09308   Ru1  1 1
0.00000
HETATM   14 Ru                  1.35187  13.23960   5.36707   Ru1  1 1
0.00000
HETATM   15 Ru                  1.35188  17.84488   1.06372   Ru1  1 1
0.00000
HETATM   16 Ru                  1.35188  17.83194   5.43176   Ru1  1 1
0.00000
HETATM   17 Ru                  1.35191   0.80407   3.22227   Ru1  1 1
0.00000
HETATM   18 Ru                  1.35191   0.80588   7.50445   Ru1  1 1
0.00000
HETATM   19 Ru                  1.35188   5.40932   3.22692   Ru1  1 1
0.00000
HETATM   20 Ru                  1.35188   5.41009   7.50880   Ru1  1 1
0.00000
HETATM   21 Ru                  1.35187  10.10674   3.22773   Ru1  1 1
0.00000
HETATM   22 Ru                  1.35186  10.10547   7.51051   Ru1  1 1
0.00000
HETATM   23 Ru                  1.35188  14.85145   3.22720   Ru1  1 1
0.00000
HETATM   24 Ru                  1.35187  14.82287   7.51013   Ru1  1 1
0.00000
HETATM   25 Ru                 -0.00006   3.03839   3.22458   Ru1  1 1
0.00000
HETATM   26 Ru                 -0.00006   3.03936   7.50628   Ru1  1 1
0.00000
HETATM   27 Ru                 -0.00009   7.76453   3.22744   Ru1  1 1
0.00000
HETATM   28 Ru                 -0.00009   7.76299   7.50892   Ru1  1 1
0.00000
HETATM   29 Ru                 -0.00008  12.46727   3.23176   Ru1  1 1
0.00000
HETATM   30 Ru                 -0.00009  12.46141   7.51105   Ru1  1 1
0.00000
```

```
HETATM    31 Ru                 -0.00007  17.19558   3.22010   Ru1  1 1
0.00000
HETATM    32 Ru                 -0.00009  17.15695   7.53004   Ru1  1 1
0.00000
HETATM    33 H                   1.35186  18.47057   3.68602    H1  1 1
0.00000
UNIT ENERGY   kcal
ENERGY        -6778.358270697675
END

XTLGRF 200
DESCRP Ruadsrect412rect100
RUTYPE NORMAL RUN
#refdata /rf3/training/Ru_ads/rect/412_rect_100
REMARK Created by geo_energy_extract.py EO = -282.95194673 eV; -
6580.277830930232 kcal/mol
CRYSTX    25.81564    4.68327    8.56336   90.00000   90.00000   90.00000
HETATM     1 Ru                  2.69882   1.52829   1.06997   Ru1  1 1
0.00000
HETATM     2 Ru                  2.69938   1.52804   5.35137   Ru1  1 1
0.00000
HETATM     3 Ru                  5.42814   1.53098   1.07232   Ru1  1 1
0.00000
HETATM     4 Ru                  5.43634   1.52590   5.35031   Ru1  1 1
0.00000
HETATM     5 Ru                  8.13621   1.56713   1.07028   Ru1  1 1
0.00000
HETATM     6 Ru                  8.14106   1.54913   5.36578   Ru1  1 1
0.00000
HETATM     7 Ru                  0.08371   1.53325   1.07034   Ru1  1 1
0.00000
HETATM     8 Ru                  0.08354   1.52800   5.35108   Ru1  1 1
0.00000
HETATM     9 Ru                  1.34993   3.90694   1.07146   Ru1  1 1
0.00000
HETATM    10 Ru                  1.35274   3.90502   5.35076   Ru1  1 1
0.00000
HETATM    11 Ru                  4.07148   3.87403   1.07001   Ru1  1 1
0.00000
HETATM    12 Ru                  4.07286   3.87279   5.35103   Ru1  1 1
0.00000
HETATM    13 Ru                  6.80378   3.87270   1.06481   Ru1  1 1
0.00000
HETATM    14 Ru                  6.80685   3.86945   5.34749   Ru1  1 1
0.00000
HETATM    15 Ru                  9.42600   3.86801   1.06334   Ru1  1 1
0.00000
HETATM    16 Ru                  9.42721   3.84237   5.39354   Ru1  1 1
0.00000
HETATM    17 Ru                  1.35278   0.71798   3.21172   Ru1  1 1
0.00000
HETATM    18 Ru                  1.34993   0.71614   7.49101   Ru1  1 1
0.00000
HETATM    19 Ru                  4.07302   0.75040   3.21131   Ru1  1 1
0.00000
HETATM    20 Ru                  4.07157   0.74921   7.49241   Ru1  1 1
0.00000
```

```
HETATM    21 Ru                    6.80699   0.75371   3.21440   Ru1  1 1
0.00000
HETATM    22 Ru                    6.80417   0.75089   7.49710   Ru1  1 1
0.00000
HETATM    23 Ru                    9.42724   0.78060   3.16854   Ru1  1 1
0.00000
HETATM    24 Ru                    9.42659   0.75711   7.49817   Ru1  1 1
0.00000
HETATM    25 Ru                    2.69939   3.09504   3.21113   Ru1  1 1
0.00000
HETATM    26 Ru                    2.69891   3.09484   7.49254   Ru1  1 1
0.00000
HETATM    27 Ru                    5.43658   3.09738   3.21199   Ru1  1 1
0.00000
HETATM    28 Ru                    5.42807   3.09255   7.48992   Ru1  1 1
0.00000
HETATM    29 Ru                    8.14151   3.07353   3.19688   Ru1  1 1
0.00000
HETATM    30 Ru                    8.13623   3.05691   7.49156   Ru1  1 1
0.00000
HETATM    31 Ru                    0.08354   3.09491   3.21139   Ru1  1 1
0.00000
HETATM    32 Ru                    0.08372   3.08981   7.49222   Ru1  1 1
0.00000
HETATM    33 H                     9.78008   2.32240   4.26758    H1  1 1
0.00000
UNIT ENERGY   kcal
ENERGY         -6580.277830930232
END

XTLGRF 200
DESCRP uadsrectRu142rect010
RUTYPE NORMAL RUN
#refdata /rf3/training/Ru_ads/rect_Ru/142_rect_010
REMARK Created by geo_energy_extract.py EO = -296.45557323 eV; -
6894.315656511629 kcal/mol
CRYSTX     2.70391   33.73310    8.56336   90.00000   90.00000   90.00000
HETATM     1 Ru                   -0.00010   1.45561   1.10291   Ru1  1 1
0.00000
HETATM     2 Ru                   -0.00010   1.45567   5.38477   Ru1  1 1
0.00000
HETATM     3 Ru                   -0.00010   6.15906   1.10110   Ru1  1 1
0.00000
HETATM     4 Ru                   -0.00005   6.15892   5.38276   Ru1  1 1
0.00000
HETATM     5 Ru                   -0.00008  10.86282   1.10105   Ru1  1 1
0.00000
HETATM     6 Ru                   -0.00009  10.86247   5.38397   Ru1  1 1
0.00000
HETATM     7 Ru                   -0.00009  15.58159   1.10802   Ru1  1 1
0.00000
HETATM     8 Ru                   -0.00005  15.58182   5.37777   Ru1  1 1
0.00000
HETATM     9 Ru                    1.35188   3.78924   1.10181   Ru1  1 1
0.00000
HETATM    10 Ru                    1.35185   3.78921   5.38437   Ru1  1 1
0.00000
```

```
HETATM    11 Ru                   1.35190   8.51178   1.09939   Ru1  1 1
0.00000
HETATM    12 Ru                   1.35188   8.51168   5.38419   Ru1  1 1
0.00000
HETATM    13 Ru                   1.35185  13.21224   1.09422   Ru1  1 1
0.00000
HETATM    14 Ru                   1.35189  13.21188   5.39101   Ru1  1 1
0.00000
HETATM    15 Ru                   1.35189  17.86976   1.12758   Ru1  1 1
0.00000
HETATM    16 Ru                   1.35190  17.86973   5.35903   Ru1  1 1
0.00000
HETATM    17 Ru                   1.35186   0.77818   3.24395   Ru1  1 1
0.00000
HETATM    18 Ru                   1.35186   0.77910   7.52556   Ru1  1 1
0.00000
HETATM    19 Ru                   1.35187   5.38305   3.24201   Ru1  1 1
0.00000
HETATM    20 Ru                   1.35189   5.38371   7.52372   Ru1  1 1
0.00000
HETATM    21 Ru                   1.35187  10.07324   3.24198   Ru1  1 1
0.00000
HETATM    22 Ru                   1.35188  10.08146   7.52411   Ru1  1 1
0.00000
HETATM    23 Ru                   1.35189  14.76809   3.24295   Ru1  1 1
0.00000
HETATM    24 Ru                   1.35186  14.82367   7.52439   Ru1  1 1
0.00000
HETATM    25 Ru                  -0.00009   3.00976   3.24360   Ru1  1 1
0.00000
HETATM    26 Ru                  -0.00010   3.01118   7.52512   Ru1  1 1
0.00000
HETATM    27 Ru                  -0.00007   7.73277   3.24174   Ru1  1 1
0.00000
HETATM    28 Ru                  -0.00006   7.73355   7.52335   Ru1  1 1
0.00000
HETATM    29 Ru                  -0.00008  12.42993   3.24277   Ru1  1 1
0.00000
HETATM    30 Ru                  -0.00008  12.43935   7.52435   Ru1  1 1
0.00000
HETATM    31 Ru                  -0.00004  17.11472   3.24303   Ru1  1 1
0.00000
HETATM    32 Ru                   2.70383  17.14667   7.52486   Ru1  1 1
0.00000
HETATM    33 Ru                   1.35193  19.32069   3.24230   Ru1  1 1
0.00000
UNIT ENERGY   kcal
ENERGY        -6894.315656511629
END

XTLGRF 200
DESCRP uadsrectRu412rect100
RUTYPE NORMAL RUN
#refdata /rf3/training/Ru_ads/rect_Ru/412_rect_100
REMARK Created by geo_energy_extract.py EO = -288.38677354 eV; -
6706.6691520930235 kcal/mol
CRYSTX    25.81564    4.68327    8.56336   90.00000   90.00000   90.00000
```

```
HETATM     1 Ru                    2.66976   1.50104   1.10353   Ru1  1 1
0.00000
HETATM     2 Ru                    2.66974   1.50103   5.38231   Ru1  1 1
0.00000
HETATM     3 Ru                    5.41400   1.50844   1.09872   Ru1  1 1
0.00000
HETATM     4 Ru                    5.41419   1.50839   5.38630   Ru1  1 1
0.00000
HETATM     5 Ru                    8.14400   1.53347   1.11262   Ru1  1 1
0.00000
HETATM     6 Ru                    8.14447   1.53409   5.37252   Ru1  1 1
0.00000
HETATM     7 Ru                    0.05556   1.50976   1.10296   Ru1  1 1
0.00000
HETATM     8 Ru                    0.05560   1.51023   5.38269   Ru1  1 1
0.00000
HETATM     9 Ru                    1.32181   3.88348   1.10245   Ru1  1 1
0.00000
HETATM    10 Ru                    1.32186   3.88355   5.38315   Ru1  1 1
0.00000
HETATM    11 Ru                    4.04206   3.84791   1.09312   Ru1  1 1
0.00000
HETATM    12 Ru                    4.04188   3.84786   5.39261   Ru1  1 1
0.00000
HETATM    13 Ru                    6.74812   3.84749   1.09778   Ru1  1 1
0.00000
HETATM    14 Ru                    6.74830   3.84761   5.38722   Ru1  1 1
0.00000
HETATM    15 Ru                    9.40404   3.87007   1.16682   Ru1  1 1
0.00000
HETATM    16 Ru                    9.40349   3.87143   5.31894   Ru1  1 1
0.00000
HETATM    17 Ru                    1.32205   0.69332   3.24281   Ru1  1 1
0.00000
HETATM    18 Ru                    1.32456   0.69019   7.52460   Ru1  1 1
0.00000
HETATM    19 Ru                    4.04948   0.72910   3.24291   Ru1  1 1
0.00000
HETATM    20 Ru                    4.05329   0.73020   7.52453   Ru1  1 1
0.00000
HETATM    21 Ru                    6.74059   0.72341   3.24243   Ru1  1 1
0.00000
HETATM    22 Ru                    6.79204   0.73749   7.52414   Ru1  1 1
0.00000
HETATM    23 Ru                    9.50674   0.84981   3.24230   Ru1  1 1
0.00000
HETATM    24 Ru                    9.39966   0.67968   7.52473   Ru1  1 1
0.00000
HETATM    25 Ru                    2.65912   3.06873   3.24280   Ru1  1 1
0.00000
HETATM    26 Ru                    2.67094   3.06668   7.52467   Ru1  1 1
0.00000
HETATM    27 Ru                    5.37432   3.06789   3.24266   Ru1  1 1
0.00000
HETATM    28 Ru                    5.41080   3.05631   7.52413   Ru1  1 1
0.00000
HETATM    29 Ru                    8.04847   3.05000   3.24243   Ru1  1 1
0.00000
```

```
HETATM    30 Ru                 8.16365   3.04211   7.52459   Ru1  1 1
0.00000
HETATM    31 Ru                 0.05108   3.07372   3.24267   Ru1  1 1
0.00000
HETATM    32 Ru                 0.05658   3.06393   7.52446   Ru1  1 1
0.00000
HETATM    33 Ru                10.59616   2.96699   3.24315   Ru1  1 1
0.00000
UNIT ENERGY   kcal
ENERGY        -6706.6691520930235
END

BIOGRF 200
DESCRP Ru_cluster_01
REMARK DataFrom
/home/phys/conwudin/ADF_jobs/training/Ru_cluster/01/Ru_cluster_01
RUTYPE SINGLE POINT
MOLCHARGE 1 1 0
HETATM     1 Ru                 0.00000   0.00000   0.00000   Ru   1 1
-0.00000
UNIT ENERGY   kcalmol
ENERGY -6.901349921740462
END

BIOGRF 200
DESCRP Ru_cluster_02
REMARK DataFrom
/home/phys/conwudin/ADF_jobs/training/Ru_cluster/02/Ru_cluster_02
RUTYPE NORMAL RUN
MOLCHARGE 1 2 0
HETATM     1 Ru                 0.00000   0.00000   0.99767   Ru   1 1
0.00000
HETATM     2 Ru                 0.00000   0.00000  -0.99767   Ru   1 1
-0.00000
UNIT ENERGY   kcalmol
ENERGY -184.4357214901065
END

BIOGRF 200
DESCRP Ru_cluster_03
REMARK DataFrom
/home/phys/conwudin/ADF_jobs/training/Ru_cluster/03/Ru_cluster_03
RUTYPE NORMAL RUN
MOLCHARGE 1 3 0
HETATM     1 Ru                 0.00000   1.11089   0.02699   Ru   1 1
-0.00700
HETATM     2 Ru                 0.00000   0.00000  -2.09617   Ru   1 1
0.01500
HETATM     3 Ru                 0.00000  -1.11089   0.02699   Ru   1 1
-0.00700
UNIT ENERGY   kcalmol
ENERGY -332.0855787219597
END

BIOGRF 200
DESCRP Ru_cluster_04
REMARK DataFrom
/home/phys/conwudin/ADF_jobs/training/Ru_cluster/04/Ru_cluster_04
```

```
RUTYPE NORMAL RUN
MOLCHARGE 1 4 0
HETATM     1 Ru                  0.00000   1.57246   0.00000   Ru   1 1
0.00000
HETATM     2 Ru                  0.00000  -1.57246   0.00000   Ru   1 1
0.00000
HETATM     3 Ru                 -1.57246   0.00000   0.00000   Ru   1 1
0.00000
HETATM     4 Ru                  1.57246   0.00000   0.00000   Ru   1 1
0.00000
UNIT ENERGY   kcalmol
ENERGY -509.5239413306944
END

BIOGRF 200
DESCRP Ru_cluster_05
REMARK DataFrom
/home/phys/conwudin/ADF_jobs/training/Ru_cluster/05/Ru_cluster_05
RUTYPE NORMAL RUN
MOLCHARGE 1 5 0
HETATM     1 Ru                 -0.03619   2.21741  -0.07540   Ru   1 1
-0.01500
HETATM     2 Ru                 -2.38933   0.01681   0.17400   Ru   1 1
-0.01300
HETATM     3 Ru                 -0.11277  -0.06548  -0.00257   Ru   1 1
-0.01300
HETATM     4 Ru                 -2.31289   2.29977   0.10131   Ru   1 1
-0.01400
HETATM     5 Ru                 -1.06723   1.17179   1.90688   Ru   1 1
0.05500
UNIT ENERGY   kcalmol
ENERGY -680.3772188258861
END

BIOGRF 200
DESCRP Ru_cluster_06
REMARK DataFrom
/home/phys/conwudin/ADF_jobs/training/Ru_cluster/06/Ru_cluster_06
RUTYPE NORMAL RUN
MOLCHARGE 1 6 0
HETATM     1 Ru                  0.01018   2.31439  -0.08289   Ru   1 1
-0.02400
HETATM     2 Ru                 -2.46903   0.03445   0.23112   Ru   1 1
-0.02300
HETATM     3 Ru                 -0.06559  -0.05206   0.00082   Ru   1 1
-0.02200
HETATM     4 Ru                 -2.39139   2.40081   0.15031   Ru   1 1
-0.02300
HETATM     5 Ru                 -1.05996   1.04935   1.88571   Ru   1 1
0.04600
HETATM     6 Ru                 -1.39972   1.29881  -1.73455   Ru   1 1
0.04600
UNIT ENERGY   kcalmol
ENERGY -831.3258937099115
END

BIOGRF 200
DESCRP Ru_cluster_07
```

```
REMARK DataFrom
/home/phys/conwudin/ADF_jobs/training/Ru_cluster/07/Ru_cluster_07
RUTYPE NORMAL RUN
MOLCHARGE 1 7 0
HETATM     1 Ru                  -0.01705   2.52982  -0.23770   Ru   1 1
-0.07500
HETATM     2 Ru                  -2.37283  -0.02205   0.25094   Ru   1 1
0.04300
HETATM     3 Ru                   0.11311   0.10530  -0.17133   Ru   1 1
-0.09000
HETATM     4 Ru                  -2.51476   2.35623   0.18095   Ru   1 1
0.02700
HETATM     5 Ru                  -0.79523   1.31993   1.72791   Ru   1 1
-0.07600
HETATM     6 Ru                  -1.61235   1.16965  -1.67775   Ru   1 1
0.04200
HETATM     7 Ru                   1.53748   1.43929   1.21322   Ru   1 1
0.12900
UNIT ENERGY   kcalmol
ENERGY -1002.174778638323
END

BIOGRF 200
DESCRP Ru_cluster_08
REMARK DataFrom
/home/phys/conwudin/ADF_jobs/training/Ru_cluster/08/Ru_cluster_08
RUTYPE NORMAL RUN
MOLCHARGE 1 8 0
HETATM     1 Ru                  -0.08662   2.46420   0.07999   Ru   1 1
-0.24900
HETATM     2 Ru                  -2.27376  -0.02886   0.50589   Ru   1 1
-0.01300
HETATM     3 Ru                  -0.23398  -0.89086  -0.35704   Ru   1 1
0.07400
HETATM     4 Ru                  -2.63472   2.43473   0.37271   Ru   1 1
0.03500
HETATM     5 Ru                  -1.03995   1.34981   2.00290   Ru   1 1
0.11700
HETATM     6 Ru                  -1.19129   1.05127  -1.37606   Ru   1 1
0.07800
HETATM     7 Ru                   0.94074   0.46989   1.11926   Ru   1 1
-0.12100
HETATM     8 Ru                   2.17731   2.47237   0.55862   Ru   1 1
0.07900
UNIT ENERGY   kcalmol
ENERGY -1166.778061114371
END

BIOGRF 200
DESCRP trainingdimerRu01
RUTYPE SINGLE POINT
#refdata /rf3/training/dimer_Ru/01
REMARK Created by geo_energy_extract.py EO = 108.28146890 eV;
2518.173695348837 kcal/mol
HETATM     1 Ru                   6.75000   6.75000   6.34500   Ru1  1 1
0.00000
HETATM     2 Ru                   6.75000   6.75000   7.15500   Ru1  1 1
0.00000
```

```
UNIT ENERGY   kcal
ENERGY        2518.173695348837
END

BIOGRF 200
DESCRP trainingdimerRu02
RUTYPE SINGLE POINT
#refdata /rf3/training/dimer_Ru/02
REMARK Created by geo_energy_extract.py EO = 15.03588873 eV;
349.6718309302326 kcal/mol
HETATM     1 Ru                  6.75000   6.75000   6.21000   Ru1  1 1
0.00000
HETATM     2 Ru                  6.75000   6.75000   7.29000   Ru1  1 1
0.00000
UNIT ENERGY   kcal
ENERGY        349.6718309302326
END

BIOGRF 200
DESCRP trainingdimerRu03
RUTYPE SINGLE POINT
#refdata /rf3/training/dimer_Ru/03
REMARK Created by geo_energy_extract.py EO = -4.44839031 eV; -
103.45093744186046 kcal/mol
HETATM     1 Ru                  6.75000   6.75000   6.07500   Ru1  1 1
0.00000
HETATM     2 Ru                  6.75000   6.75000   7.42500   Ru1  1 1
0.00000
UNIT ENERGY   kcal
ENERGY        -103.45093744186046
END

BIOGRF 200
DESCRP trainingdimerRu04
RUTYPE SINGLE POINT
#refdata /rf3/training/dimer_Ru/04
REMARK Created by geo_energy_extract.py EO = -7.18061935 eV; -
166.9911476744186 kcal/mol
HETATM     1 Ru                  6.75000   6.75000   5.94000   Ru1  1 1
0.00000
HETATM     2 Ru                  6.75000   6.75000   7.56000   Ru1  1 1
0.00000
UNIT ENERGY   kcal
ENERGY        -166.9911476744186
END

BIOGRF 200
DESCRP trainingdimerRu05
RUTYPE SINGLE POINT
#refdata /rf3/training/dimer_Ru/05
REMARK Created by geo_energy_extract.py EO = -8.55603805 eV; -
198.97762906976746 kcal/mol
HETATM     1 Ru                  6.75000   6.75000   5.80500   Ru1  1 1
0.00000
HETATM     2 Ru                  6.75000   6.75000   7.69500   Ru1  1 1
0.00000
UNIT ENERGY   kcal
ENERGY        -198.97762906976746
```

```
END

BIOGRF 200
DESCRP trainingdimerRu06
RUTYPE SINGLE POINT
#refdata /rf3/training/dimer_Ru/06
REMARK Created by geo_energy_extract.py EO = -8.76571117 eV; -
203.8537481395349 kcal/mol
HETATM     1 Ru                  6.75000   6.75000   5.67000   Ru1  1 1
0.00000
HETATM     2 Ru                  6.75000   6.75000   7.83000   Ru1  1 1
0.00000
UNIT ENERGY   kcal
ENERGY        -203.8537481395349
END

BIOGRF 200
DESCRP trainingdimerRu07
RUTYPE SINGLE POINT
#refdata /rf3/training/dimer_Ru/07
REMARK Created by geo_energy_extract.py EO = -8.22015431 eV; -
191.1663793023256 kcal/mol
HETATM     1 Ru                  6.75000   6.75000   5.53500   Ru1  1 1
0.00000
HETATM     2 Ru                  6.75000   6.75000   7.96500   Ru1  1 1
0.00000
UNIT ENERGY   kcal
ENERGY        -191.1663793023256
END

BIOGRF 200
DESCRP trainingdimerRu08
RUTYPE SINGLE POINT
#refdata /rf3/training/dimer_Ru/08
REMARK Created by geo_energy_extract.py EO = -7.30823101 eV; -
169.95886069767442 kcal/mol
HETATM     1 Ru                  6.75000   6.75000   5.40000   Ru1  1 1
0.00000
HETATM     2 Ru                  6.75000   6.75000   8.10000   Ru1  1 1
0.00000
UNIT ENERGY   kcal
ENERGY        -169.95886069767442
END

BIOGRF 200
DESCRP trainingdimerRu09
RUTYPE SINGLE POINT
#refdata /rf3/training/dimer_Ru/09
REMARK Created by geo_energy_extract.py EO = -6.42399632 eV; -
149.39526325581394 kcal/mol
HETATM     1 Ru                  6.75000   6.75000   5.26500   Ru1  1 1
0.00000
HETATM     2 Ru                  6.75000   6.75000   8.23500   Ru1  1 1
0.00000
UNIT ENERGY   kcal
ENERGY        -149.39526325581394
END
```

```
BIOGRF 200
DESCRP trainingdimerRu10
RUTYPE SINGLE POINT
#refdata /rf3/training/dimer_Ru/10
REMARK Created by geo_energy_extract.py EO = -5.69443254 eV; -
132.42866372093025 kcal/mol
HETATM     1 Ru                  6.75000   6.75000   5.13000   Ru1  1 1
0.00000
HETATM     2 Ru                  6.75000   6.75000   8.37000   Ru1  1 1
0.00000
UNIT ENERGY   kcal
ENERGY        -132.42866372093025
END

BIOGRF 200
DESCRP trainingdimerRu11
RUTYPE SINGLE POINT
#refdata /rf3/training/dimer_Ru/11
REMARK Created by geo_energy_extract.py EO = -5.45580317 eV; -
126.87914348837211 kcal/mol
HETATM     1 Ru                  6.75000   6.75000   4.99500   Ru1  1 1
0.00000
HETATM     2 Ru                  6.75000   6.75000   8.50500   Ru1  1 1
0.00000
UNIT ENERGY   kcal
ENERGY        -126.87914348837211
END

BIOGRF 200
DESCRP trainingdimerRu12
RUTYPE SINGLE POINT
#refdata /rf3/training/dimer_Ru/12
REMARK Created by geo_energy_extract.py EO = -5.05612989 eV; -
117.58441604651165 kcal/mol
HETATM     1 Ru                  6.75000   6.75000   4.86000   Ru1  1 1
0.00000
HETATM     2 Ru                  6.75000   6.75000   8.64000   Ru1  1 1
0.00000
UNIT ENERGY   kcal
ENERGY        -117.58441604651165
END

BIOGRF 200
DESCRP trainingdimerRu13
RUTYPE SINGLE POINT
#refdata /rf3/training/dimer_Ru/13
REMARK Created by geo_energy_extract.py EO = -4.90363353 eV; -
114.03798906976743 kcal/mol
HETATM     1 Ru                  6.75000   6.75000   4.72500   Ru1  1 1
0.00000
HETATM     2 Ru                  6.75000   6.75000   8.77500   Ru1  1 1
0.00000
UNIT ENERGY   kcal
ENERGY        -114.03798906976743
END

BIOGRF 200
DESCRP trainingdimerRurelax
```

```
RUTYPE NORMAL RUN
#refdata /rf3/training/dimer_Ru/relax
REMARK Created by geo_energy_extract.py EO = -8.93076466 eV; -
207.69220139534883 kcal/mol
HETATM     1 Ru                 6.75000   6.75000   5.73009   Ru1  1 1
0.00000
HETATM     2 Ru                 6.75000   6.75000   7.76991   Ru1  1 1
0.00000
UNIT ENERGY   kcal
ENERGY        -207.69220139534883
END

XTLGRF 200
DESCRP Ruslaboddsurfbcc100
RUTYPE NORMAL RUN
#refdata /rf3/training/Ru_slab/odd_surf/bcc100
REMARK Created by geo_energy_extract.py EO = -64.57114818 eV; -
1501.6546088372093 kcal/mol
CRYSTX     3.83093    3.83093   30.64741   90.00000   90.00000   90.00000
HETATM     1 Ru                 0.28732   0.75498   3.11920   Ru1  1 1
0.00000
HETATM     2 Ru                 2.08528   0.78374   4.57797   Ru1  1 1
0.00000
HETATM     3 Ru                 3.73868   2.69306   4.79185   Ru1  1 1
0.00000
HETATM     4 Ru                 1.86466   2.67214   6.51673   Ru1  1 1
0.00000
HETATM     5 Ru                 0.03120   0.77111   6.59919   Ru1  1 1
0.00000
HETATM     6 Ru                 2.04105   0.79144   8.30864   Ru1  1 1
0.00000
HETATM     7 Ru                 3.77675   0.47171   9.83752   Ru1  1 1
0.00000
HETATM     8 Ru                 1.49875   2.55461   9.88186   Ru1  1 1
0.00000
UNIT ENERGY   kcal
ENERGY        -1501.6546088372093
END

XTLGRF 200
DESCRP Ruslaboddsurfbcc110
RUTYPE NORMAL RUN
#refdata /rf3/training/Ru_slab/odd_surf/bcc110
REMARK Created by geo_energy_extract.py EO = -48.27812339 eV; -
1122.7470555813954 kcal/mol
CRYSTX     3.50000    3.49629   30.00000   90.00000   90.00000  109.29005
HETATM     1 Ru                 1.88577   0.83937  25.87815   Ru1  1 1
0.00000
HETATM     2 Ru                 3.17900  -0.98032  26.95490   Ru1  1 1
0.00000
HETATM     3 Ru                 1.88292   0.84421  23.07731   Ru1  1 1
0.00000
HETATM     4 Ru                 3.07155  -0.82930  24.42283   Ru1  1 1
0.00000
HETATM     5 Ru                 1.77723   0.99274  20.54518   Ru1  1 1
0.00000
HETATM     6 Ru                 3.06930  -0.82529  21.62164   Ru1  1 1
0.00000
```

```
UNIT ENERGY   kcal
ENERGY        -1122.7470555813954
END

XTLGRF 200
DESCRP Ruslaboddsurffcc100
RUTYPE NORMAL RUN
#refdata /rf3/training/Ru_slab/odd_surf/fcc100
REMARK Created by geo_energy_extract.py EO = -67.88256308 eV; -
1578.6642576744186 kcal/mol
CRYSTX     3.80000    3.80000   35.00000   90.00000   90.00000   90.00000
HETATM     1 Ru                  3.79976   0.94973  20.24391   Ru1  1 1
0.00000
HETATM     2 Ru                  1.90079   2.84998  20.24398   Ru1  1 1
0.00000
HETATM     3 Ru                  0.00100   2.84999  18.51314   Ru1  1 1
0.00000
HETATM     4 Ru                  1.90016   0.95020  18.51344   Ru1  1 1
0.00000
HETATM     5 Ru                  3.79906   0.95025  16.48668   Ru1  1 1
0.00000
HETATM     6 Ru                  1.89990   2.84967  16.48690   Ru1  1 1
0.00000
HETATM     7 Ru                  0.00011   2.85003  14.75613   Ru1  1 1
0.00000
HETATM     8 Ru                  1.89922   0.95015  14.75581   Ru1  1 1
0.00000
UNIT ENERGY   kcal
ENERGY        -1578.6642576744186
END

XTLGRF 200
DESCRP Ruslaboddsurffcc111
RUTYPE NORMAL RUN
#refdata /rf3/training/Ru_slab/odd_surf/fcc111
REMARK Created by geo_energy_extract.py EO = -70.72869846 eV; -
1644.8534525581397 kcal/mol
CRYSTX     3.80000    3.80211   40.00000   90.00000   90.00000  119.98166
HETATM     1 Ru                  2.19596  -0.00284  17.20942   Ru1  1 1
0.00000
HETATM     2 Ru                  1.09875   1.89555  15.58344   Ru1  1 1
0.00000
HETATM     3 Ru                  0.00117   3.79725  16.93965   Ru1  1 1
0.00000
HETATM     4 Ru                  2.19430   0.00149  20.00015   Ru1  1 1
0.00000
HETATM     5 Ru                  1.09824   1.90123  18.83965   Ru1  1 1
0.00000
HETATM     6 Ru                  3.29036  -1.89826  21.15870   Ru1  1 1
0.00000
HETATM     7 Ru                  2.19276   0.00558  22.79009   Ru1  1 1
0.00000
HETATM     8 Ru                  1.09591   1.90658  23.06160   Ru1  1 1
0.00000
HETATM     9 Ru                  3.29007  -1.89287  24.41731   Ru1  1 1
0.00000
UNIT ENERGY   kcal
ENERGY        -1644.8534525581397
```

```
END

XTLGRF 200
DESCRP laboddsurfHadsbcc100
RUTYPE NORMAL RUN
#refdata /rf3/training/Ru_slab/odd_surf_Hads/bcc100
REMARK Created by geo_energy_extract.py EO = -68.52802275 eV; -
1593.674947674419 kcal/mol
CRYSTX     3.83093    3.83093   30.64741   90.00000   90.00000   90.00000
HETATM     1 Ru                  0.07382   0.68261   3.11279   Ru1  1 1
0.00000
HETATM     2 Ru                  1.87030   0.73457   4.58596   Ru1  1 1
0.00000
HETATM     3 Ru                  3.53164   2.63278   4.78662   Ru1  1 1
0.00000
HETATM     4 Ru                  1.66598   2.61893   6.50925   Ru1  1 1
0.00000
HETATM     5 Ru                  3.66412   0.70876   6.60640   Ru1  1 1
0.00000
HETATM     6 Ru                  1.80923   0.77722   8.31438   Ru1  1 1
0.00000
HETATM     7 Ru                  3.57734   0.44626   9.85262   Ru1  1 1
0.00000
HETATM     8 Ru                  1.50319   2.52863   9.88110   Ru1  1 1
0.00000
HETATM     9 H                   3.37916   2.30468  10.47867    H1  1 1
0.00000
UNIT ENERGY   kcal
ENERGY        -1593.674947674419
END

XTLGRF 200
DESCRP laboddsurfHadsbcc110
RUTYPE NORMAL RUN
#refdata /rf3/training/Ru_slab/odd_surf_Hads/bcc110
REMARK Created by geo_energy_extract.py EO = -52.15459335 eV; -
1212.897519767442 kcal/mol
CRYSTX     3.50000    3.49629   30.00000   90.00000   90.00000  109.29005
HETATM     1 Ru                  1.78352   0.97666  25.74148   Ru1  1 1
0.00000
HETATM     2 Ru                  3.11841  -0.89992  27.05108   Ru1  1 1
0.00000
HETATM     3 Ru                  1.80861   0.94449  23.09811   Ru1  1 1
0.00000
HETATM     4 Ru                  2.98251  -0.70987  24.50084   Ru1  1 1
0.00000
HETATM     5 Ru                  1.69705   1.10084  20.55582   Ru1  1 1
0.00000
HETATM     6 Ru                  2.97954  -0.70299  21.61083   Ru1  1 1
0.00000
HETATM     7 H                   2.14788   0.50224  27.56349    H1  1 1
0.00000
UNIT ENERGY   kcal
ENERGY        -1212.897519767442
END

XTLGRF 200
DESCRP laboddsurfHadsfcc100
```

```
RUTYPE NORMAL RUN
#refdata /rf3/training/Ru_slab/odd_surf_Hads/fcc100
REMARK Created by geo_energy_extract.py EO = -71.84569175 eV; -
1670.8300406976746 kcal/mol
CRYSTX     3.80000    3.80000   35.00000   90.00000   90.00000   90.00000
HETATM     1 Ru                   0.00077   1.05517  20.30114   Ru1  1 1
0.00000
HETATM     2 Ru                   1.90066   2.95518  20.30156   Ru1  1 1
0.00000
HETATM     3 Ru                   0.00073   2.95500  18.56887   Ru1  1 1
0.00000
HETATM     4 Ru                   1.90035   1.05451  18.54184   Ru1  1 1
0.00000
HETATM     5 Ru                   0.00055   1.05922  16.52081   Ru1  1 1
0.00000
HETATM     6 Ru                   1.90078   2.95890  16.52106   Ru1  1 1
0.00000
HETATM     7 Ru                   0.00077   2.95924  14.79371   Ru1  1 1
0.00000
HETATM     8 Ru                   1.90081   1.05922  14.78328   Ru1  1 1
0.00000
HETATM     9 H                    1.89381   1.04373  21.07353    H1  1 1
0.00000
UNIT ENERGY   kcal
ENERGY        -1670.8300406976746
END

XTLGRF 200
DESCRP laboddsurfHadsfcc111
RUTYPE NORMAL RUN
#refdata /rf3/training/Ru_slab/odd_surf_Hads/fcc111
REMARK Created by geo_energy_extract.py EO = -74.34528222 eV; -
1728.9600516279072 kcal/mol
CRYSTX     3.80000    3.80211   40.00000   90.00000   90.00000  119.98166
HETATM     1 Ru                   2.21870  -0.04047  17.25290   Ru1  1 1
0.00000
HETATM     2 Ru                   1.12049   1.85938  15.64065   Ru1  1 1
0.00000
HETATM     3 Ru                   0.02371   3.76011  16.99492   Ru1  1 1
0.00000
HETATM     4 Ru                   2.21882  -0.03914  20.07956   Ru1  1 1
0.00000
HETATM     5 Ru                   1.12280   1.86010  18.86661   Ru1  1 1
0.00000
HETATM     6 Ru                   0.02728   3.75640  21.17420   Ru1  1 1
0.00000
HETATM     7 Ru                   2.21876  -0.03563  22.81205   Ru1  1 1
0.00000
HETATM     8 Ru                   1.11346   1.88159  23.17664   Ru1  1 1
0.00000
HETATM     9 Ru                   0.02472   3.77525  24.62512   Ru1  1 1
0.00000
HETATM    10 H                    1.43174   1.29493  24.99464    H1  1 1
0.00000
UNIT ENERGY   kcal
ENERGY        -1728.9600516279072
END
```

```
XTLGRF 200
DESCRP aboddsurfRuadsbcc100
RUTYPE NORMAL RUN
#refdata /rf3/training/Ru_slab/odd_surf_Ruads/bcc100
REMARK Created by geo_energy_extract.py EO = -72.84215340 eV; -
1694.0035674418607 kcal/mol
CRYSTX     3.83093    3.83093   30.64741   90.00000   90.00000   90.00000
HETATM     1 Ru                  0.32848   0.85418   3.06461   Ru1  1 1
0.00000
HETATM     2 Ru                  2.13562   0.85432   4.50554   Ru1  1 1
0.00000
HETATM     3 Ru                  3.75346   2.76983   4.73326   Ru1  1 1
0.00000
HETATM     4 Ru                  1.87495   2.77020   6.47598   Ru1  1 1
0.00000
HETATM     5 Ru                  0.06918   0.85444   6.58003   Ru1  1 1
0.00000
HETATM     6 Ru                  2.05772   0.85477   8.19864   Ru1  1 1
0.00000
HETATM     7 Ru                  3.67819   0.85581  10.08008   Ru1  1 1
0.00000
HETATM     8 Ru                  1.39457   2.76996   9.87944   Ru1  1 1
0.00000
HETATM     9 Ru                  1.95169   0.85092  11.52960   Ru1  1 1
0.00000
UNIT ENERGY   kcal
ENERGY        -1694.0035674418607
END

XTLGRF 200
DESCRP aboddsurfRuadsbcc110
RUTYPE NORMAL RUN
#refdata /rf3/training/Ru_slab/odd_surf_Ruads/bcc110
REMARK Created by geo_energy_extract.py EO = -56.95564635 eV; -
1324.5499151162792 kcal/mol
CRYSTX     3.50000    3.49629   30.00000   90.00000   90.00000  109.29005
HETATM     1 Ru                  1.88725   0.83909  25.70527   Ru1  1 1
0.00000
HETATM     2 Ru                  3.05555  -0.80650  27.21799   Ru1  1 1
0.00000
HETATM     3 Ru                  1.88748   0.83878  23.07095   Ru1  1 1
0.00000
HETATM     4 Ru                  3.08809  -0.85144  24.38797   Ru1  1 1
0.00000
HETATM     5 Ru                  1.76996   1.00165  20.50300   Ru1  1 1
0.00000
HETATM     6 Ru                  3.05504  -0.80575  21.56215   Ru1  1 1
0.00000
HETATM     7 Ru                  1.77417   0.99564  28.27429   Ru1  1 1
0.00000
UNIT ENERGY   kcal
ENERGY        -1324.5499151162792
END

XTLGRF 200
DESCRP aboddsurfRuadsfcc100
RUTYPE NORMAL RUN
#refdata /rf3/training/Ru_slab/odd_surf_Ruads/fcc100
```

```
REMARK Created by geo_energy_extract.py EO = -76.04498671 eV; -
1768.488063023256 kcal/mol
CRYSTX     3.80000    3.80000   35.00000   90.00000   90.00000   90.00000
HETATM     1 Ru                -0.00016   0.93938  20.28823   Ru1  1 1
0.00000
HETATM     2 Ru                 1.90007   3.01780  20.32307   Ru1  1 1
0.00000
HETATM     3 Ru                -0.00004   2.91434  18.49440   Ru1  1 1
0.00000
HETATM     4 Ru                 1.89990   1.05719  18.39240   Ru1  1 1
0.00000
HETATM     5 Ru                -0.00002   1.03082  16.45007   Ru1  1 1
0.00000
HETATM     6 Ru                 1.90000   2.93534  16.46800   Ru1  1 1
0.00000
HETATM     7 Ru                 0.00000   2.92895  14.70674   Ru1  1 1
0.00000
HETATM     8 Ru                 1.89999   1.03172  14.70155   Ru1  1 1
0.00000
HETATM     9 Ru                 1.89947   1.24461  21.93134   Ru1  1 1
0.00000
UNIT ENERGY   kcal
ENERGY        -1768.488063023256
END

XTLGRF 200
DESCRP aboddsurfRuadsfcc111
RUTYPE NORMAL RUN
#refdata /rf3/training/Ru_slab/odd_surf_Ruads/fcc111
REMARK Created by geo_energy_extract.py EO = -80.31783849 eV; -
1867.8567090697675 kcal/mol
CRYSTX     3.80000    3.80211   40.00000   90.00000   90.00000  119.98166
HETATM     1 Ru                 2.24757  -0.09071  17.10187   Ru1  1 1
0.00000
HETATM     2 Ru                 1.15011   1.80861  15.52244   Ru1  1 1
0.00000
HETATM     3 Ru                 0.05289  -0.09251  16.87496   Ru1  1 1
0.00000
HETATM     4 Ru                 2.24791  -0.09205  20.01655   Ru1  1 1
0.00000
HETATM     5 Ru                 1.15060   1.81063  18.66486   Ru1  1 1
0.00000
HETATM     6 Ru                 0.05352  -0.09445  20.84866   Ru1  1 1
0.00000
HETATM     7 Ru                 2.25057  -0.09521  22.57703   Ru1  1 1
0.00000
HETATM     8 Ru                 1.15419   1.80495  23.40213   Ru1  1 1
0.00000
HETATM     9 Ru                 0.05806  -0.09697  25.11034   Ru1  1 1
0.00000
HETATM    10 Ru                 1.15505   1.80391  25.89847   Ru1  1 1
0.00000
UNIT ENERGY   kcal
ENERGY        -1867.8567090697675
END

XTLGRF 200
DESCRP eosRuhcpRufinal
```

```
RUTYPE NORMAL RUN
#refdata /ortho_train/Ru/eos_Ru/hcp_Ru/final
REMARK Created by geo_energy_extract.py EO = -37.01587679 eV; -
860.8343439534884 kcal/mol
CRYSTX     2.71507    4.70567    4.27725   89.98371   89.99535   90.00166
HETATM     1 Ru                -0.00046   1.56592   1.06935   Ru1  1 1
0.00000
HETATM     2 Ru                 1.35707   3.91868   1.06951   Ru1  1 1
0.00000
HETATM     3 Ru                 1.35837   0.78691   3.20805   Ru1  1 1
0.00000
HETATM     4 Ru                 0.00083   3.13983   3.20798   Ru1  1 1
0.00000
UNIT ENERGY   kcal
ENERGY        -860.8343439534884
END

XTLGRF 200
DESCRP eosRufccRufinal
RUTYPE NORMAL RUN
#refdata /ortho_train/Ru/eos_Ru/fcc_Ru/final
REMARK Created by geo_energy_extract.py EO = -36.55863526 eV; -
850.2008200000001 kcal/mol
CRYSTX     3.80427    3.80032    3.79819   90.02960   90.03104   89.99023
HETATM     1 Ru                 2.85307   2.85108   0.94876   Ru1  1 1
0.00000
HETATM     2 Ru                 2.85282   0.95065   2.84812   Ru1  1 1
0.00000
HETATM     3 Ru                 0.95067   2.85016   2.84810   Ru1  1 1
0.00000
HETATM     4 Ru                 0.95094   0.95027   0.94927   Ru1  1 1
0.00000
UNIT ENERGY   kcal
ENERGY        -850.2008200000001
END

XTLGRF 200
DESCRP eosRubccRufinal
RUTYPE NORMAL RUN
#refdata /rf3/training/eos_Ru/bcc_Ru/final
REMARK Created by geo_energy_extract.py EO = -18.26831001 eV; -
424.84441883720933 kcal/mol
CRYSTX     2.69241    2.69228    3.78567   90.00826   90.00859   90.08439
HETATM     1 Ru                -0.00000  -0.00001   0.00000   Ru1  1 1
0.00000
HETATM     2 Ru                 1.36795   1.36090   1.86522   Ru1  1 1
0.00000
UNIT ENERGY   kcal
ENERGY        -424.84441883720933
END
```
