# Supplementary material for: A ReaxFF Molecular Dynamics Study of Hydrogen Diffusion in Ruthenium–The Role of Grain Boundaries
Source: J Phys Chem C Nanomater Interfaces. 2022 Mar 23;126(13):5950–9. doi: 10.1021/acs.jpcc.1c08776 (PMC8996245; doi:10.1021/acs.jpcc.1c08776)
Supplement: Supplementary file 3 — jp1c08776_si_003.pdf [file jp1c08776_si_003.pdf]

A ReaxFF molecular dynamics study of hydrogen diffusion in ruthenium – the role of grain boundaries

Chidozie Onwudinanti,[†,‡,¶] Mike Pols,[‡] Geert Brocks,[‡,¶,§] Vianney Koelman,[¶,†,‖] Adri C.T. van Duin,[⊥] Thomas Morgan,[†] and Shuxia Tao[*,‡,¶]

†*Dutch Institute for Fundamental Energy Research, P.O. Box 6336, 5600 HH Eindhoven, The Netherlands*

‡*Materials Simulation and Modelling, Department of Applied Physics, Eindhoven University of Technology, 5600 MB Eindhoven, The Netherlands*

¶*Center for Computational Energy Research, P.O. Box 6336, 5600 HH Eindhoven, The Netherlands*

§*Computational Materials Science, Faculty of Science and Technology, MESA+ Institute for Nanotechnology, University of Twente, P.O. Box 217, 7500 AE Enschede, The Netherlands*

‖*Department of Applied Physics, Eindhoven University of Technology, 5600 MB Eindhoven, The Netherlands*

⊥*Department of Mechanical Engineering, The Pennsylvania State University, University Park, PA 16802, USA*

E-mail: s.x.tao@tue.nl

ReaxFF training set

The fitting of parameters for the force field was done with a training set which includes Ru equations of state for multiple crystal structures (hcp, fcc, bcc, sc); Ru clusters; amorphous Ru; surface formation energies (slabs of hcp Ru, fcc Ru, bcc Ru); H adsorption energies on the same slabs; interstitial hydride formation energies (up to 0.25 H/Ru concentration); and bond length scans(Ru-Ru and Ru-H in RuH_4). Figures S1 and S2 show samples of the training set.

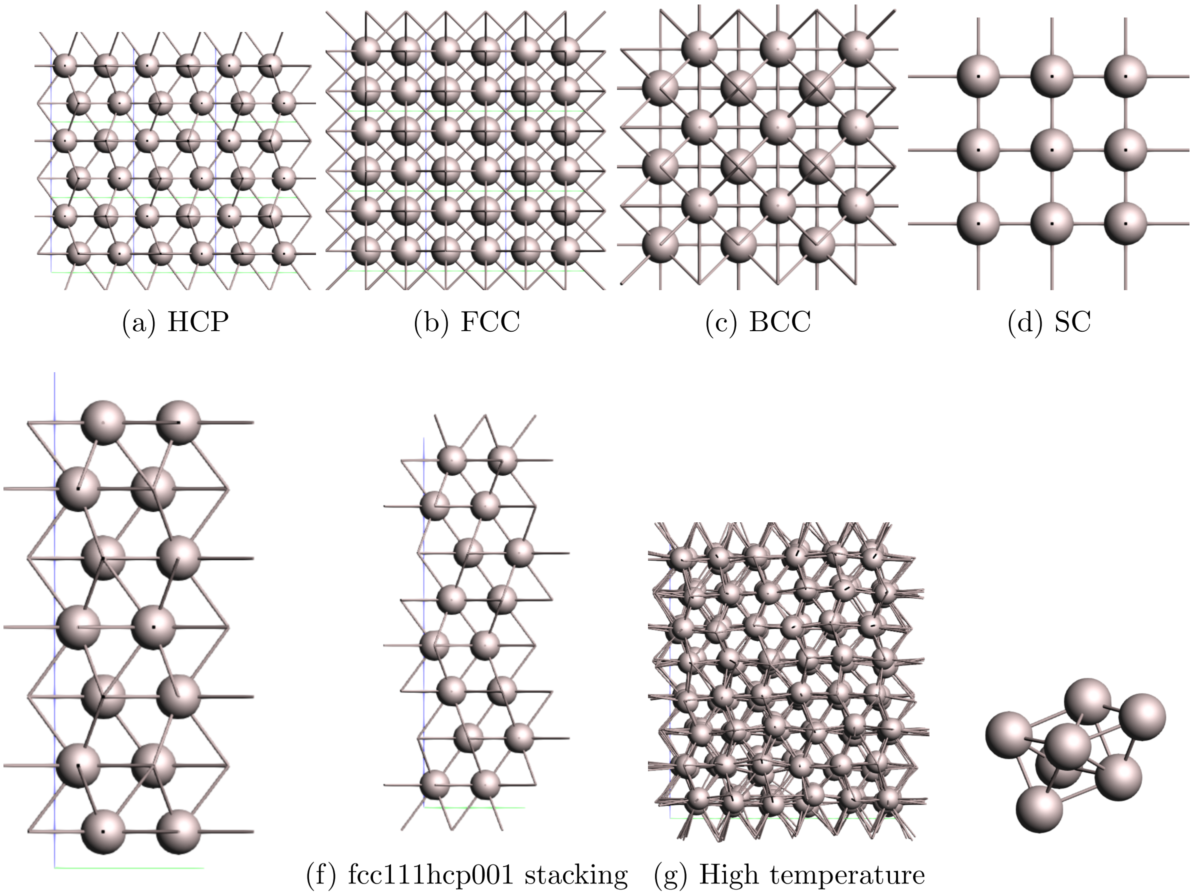

(a) HCP (b) FCC (c) BCC (d) SC

(e) Ru(0001) slab (f) fcc111hcp001 stacking fault (g) High temperature Ru (h) Ru cluster

Figure S1: Examples of structures included in the Ru training set.

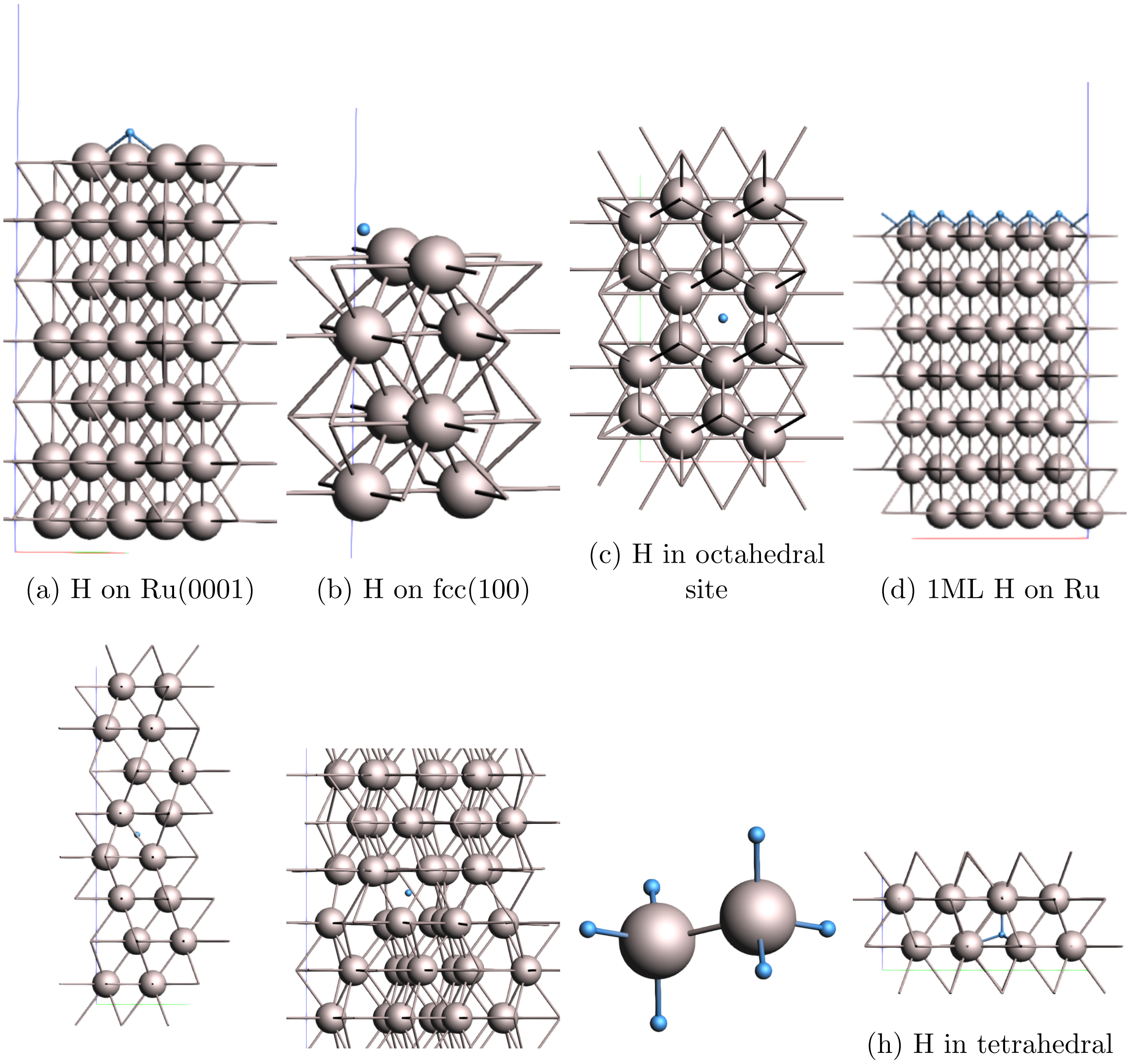

(a) H on Ru(0001) (b) H on fcc(100) (c) H in octahedral site (d) 1ML H on Ru

(e) H in fcc111hc001 (f) H in twist GB (g) Ru_2H_6 (h) H in tetrahedral site

Figure S2: Examples of structures included in the Ru/H training set.

Ru-H force field parameters

```
Reactive MD force field: RuH
 39       ! Number of general parameters
   50.0000 !p_boc1 Eq(4c): Overcoordination parameter
    9.5469 !p_boc2 Eq(4d): Overcoordination parameter
    1.6725 !p_coa2 Eq(15): Valency angle conjugation
    1.7224 !p_trip4 Eq(20): Triple bond stabilisation
    6.8702 !p_trip3 Eq(20): Triple bond stabilisation
   60.4850 !k_c2 Eq(19): C2-correction
    1.0588 !p_ovun6 Eq(12): Undercoordination
    4.6000 !p_trip2 Eq(20): Triple bond stabilisation
   12.1176 !p_ovun7 Eq(12): Undercoordination
   13.3056 !p_ovun8 Eq(12): Undercoordination
  -70.5044 !p_trip1 Eq(20): Triple bond stabilization
    0.0000 !Lower Taper-radius (must be 0)
   10.0000 !R_cut Eq(21): Upper Taper-radius
    2.8793 !p_fe1 Eq(6a): Fe dimer correction
   33.8667 !p_val6 Eq(13c): Valency undercoordination
    6.0891 !p_lp1 Eq(8): Lone pair param
    1.0563 !p_val9 Eq(13f): Valency angle exponent
    2.0384 !p_val10 Eq(13g): Valency angle parameter
    6.1431 !p_fe2 Eq(6a): Fe dimer correction
    6.9290 !p_pen2 Eq(14a): Double bond/angle param
    0.3989 !p_pen3 Eq(14a): Double bond/angle param
    3.9954 !p_pen4 Eq(14a): Double bond/angle param
   -2.4837 !p_fe3 Eq(6a): Fe dimer correction
    5.7796 !p_tor2 Eq(16b): Torsion/BO parameter
   10.0000 !p_tor3 Eq(16c): Torsion overcoordination
    1.9487 !p_tor4 Eq(16c): Torsion overcoordination
   -1.2327 !p_elho Eq(26a): electron-hole interaction
    2.1645 !p_cot2 Eq(17b): Conjugation if tors13=0
    1.5591 !p_vdW1 Eq(23b): vdWaals shielding
    0.1000 !Cutoff for bond order (*100)
    1.7602 !p_coa4 Eq(15): Valency angle conjugation
    0.6991 !p_ovun4 Eq(11b): Over/Undercoordination
   50.0000 !p_ovun3 Eq(11b): Over/Undercoordination
    1.8512 !p_val8 Eq(13d): Valency/lone pair param
    0.5000 !X_soft Eq(25): ACKS2 softness for X_ij
   20.0000 !d Eq(23d): Scale factor in lg-dispersion
    5.0000 !p_val Eq(27): Gauss exponent for electrons
    0.0000 !1 Eq(13e): disable undecoord in val angle
    0.7903 !p_coa3 Eq(15): Valency angle conjugation
  2    ! Nr of atoms; cov.r; valency;a.m;Rvdw;Evdw;gammaEEM;cov.r2;#
            alfa;gammavdW;valency;Eunder;Eover;chiEEM;etaEEM;n.u.
            cov r3;Elp;Heat inc.;bo131;bo132;bo133;softcut;n.u.
            ov/un;val1;n.u.;val3,vval4
 H    0.8930   1.0000   1.0080   1.3550   0.0930   0.8203  -0.1000   1.0000
      8.2230  33.2894   1.0000   0.0000 121.1250   3.7248   9.6093   1.0000
     -0.1000   0.0000  55.1878   3.0408   2.4197   0.0003   1.0698   0.0000
    -19.4571   4.2733   1.0338   1.0000   2.8793   0.0000   0.0000   0.0000
 Ru   2.6757   4.0000 101.0700   3.7443   0.0394   0.8412  -1.0000   4.0000
     11.0901  41.5093   4.0000   0.0000   0.0000   3.0356   6.7556   0.0000
     -1.0000   0.0000  10.1626 109.5355  76.2738   0.0009   0.8563   0.0000
     -1.0840   0.8894   1.0338   8.0000   2.5791   0.0000   0.0000   0.0000
  3    ! Nr of bonds; Edis1;LPpen;n.u.;pbe1;pbo5;13corr;pbo6
                         pbe2;pbo3;pbo4;n.u.;pbo1;pbo2;ovcorr
  1  1 153.3934   0.0000   0.0000  -0.4600   0.0000   1.0000   6.0000   0.7300
         6.2500   1.0000   0.0000   1.0000  -0.0790   6.0552   0.0000   0.0000
  1  2 110.8446   0.0000   0.0000  -0.6157   0.0000   1.0000  16.0000   0.5137
         0.1307   1.0000   0.0000   1.0000  -0.4436   6.1985   0.0000   0.0000
  2  2 117.1478   0.0000   0.0000  -0.6450  -0.2000   0.0000  16.0000   0.1685
        15.8671  -0.2000  15.0000   1.0000  -0.0221   9.5113   0.0000   0.0000
  1    ! Nr of off-diagonal terms; Ediss;Ro;gamma;rsigma;rpi;rpi2
  1  2   0.2510   1.2762  10.8237   2.2667  -1.0000  -1.0000
  5    ! Nr of angles;at1;at2;at3;Thetao,o;ka;kb;pv1;pv2
  1  1  1   0.0000   7.9213   0.8635   0.0000   0.0000   0.0000   1.0400
  1  2  1  -0.2670   0.2116   1.7042   0.0000   1.4791   0.0000   3.1294
  2  1  2   0.0000   1.7341   2.1648   0.0000   0.8397   0.0000   2.7364
  1  1  2   0.0000  25.3724   7.3597   0.0000   2.5136   0.0000   3.1412
  1  2  2  64.6157  27.9453   0.0199   0.0000   0.0948   0.0000   3.5155
  0    ! Nr of torsions;at1;at2;at3;at4;;V1;V2;V3;V2(BO);vconj;n.u;n
  0    ! Nr of hydrogen bonds;at1;at2;at3;Rhb;Dehb;vhb1
```

MSD plots

In this section are MSD plots for each of the simulated structures at temperatures 500, 600, 800, and 900K. Note that the scales on the vertical axis differ significantly.

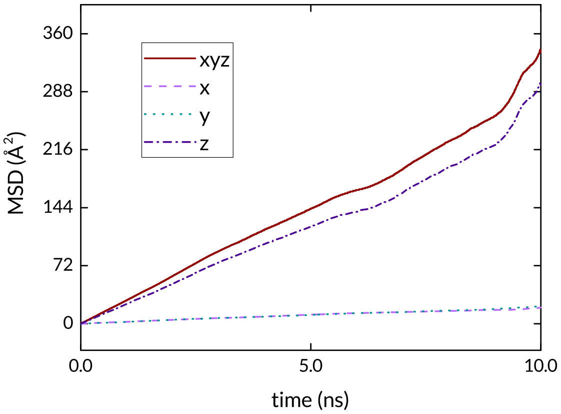

(a) 500K

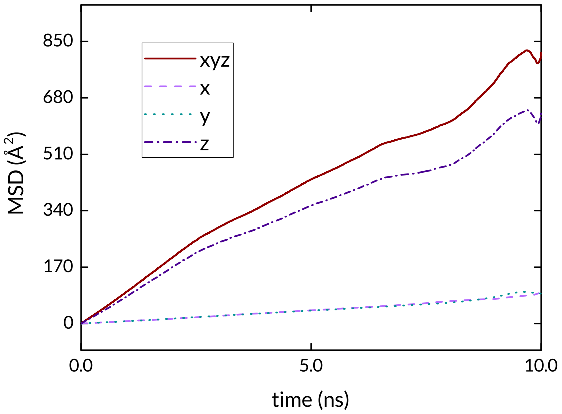

(b) 600K

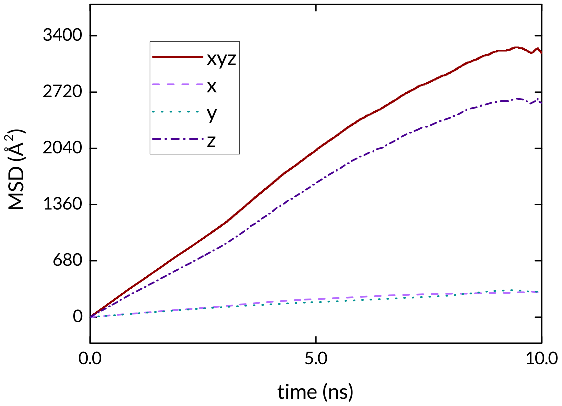

(c) 800K

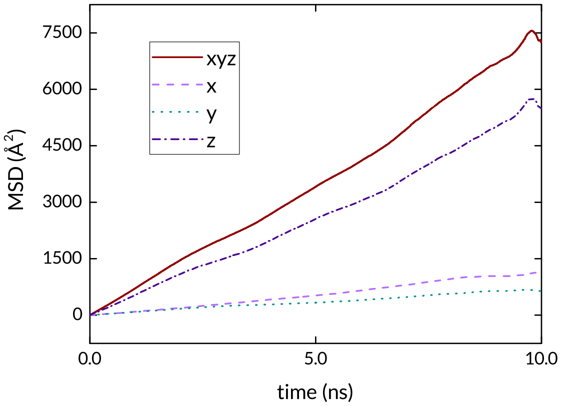

(d) 900K

Figure S3: MSD plots for NVT simulations of H in pristine Ru.

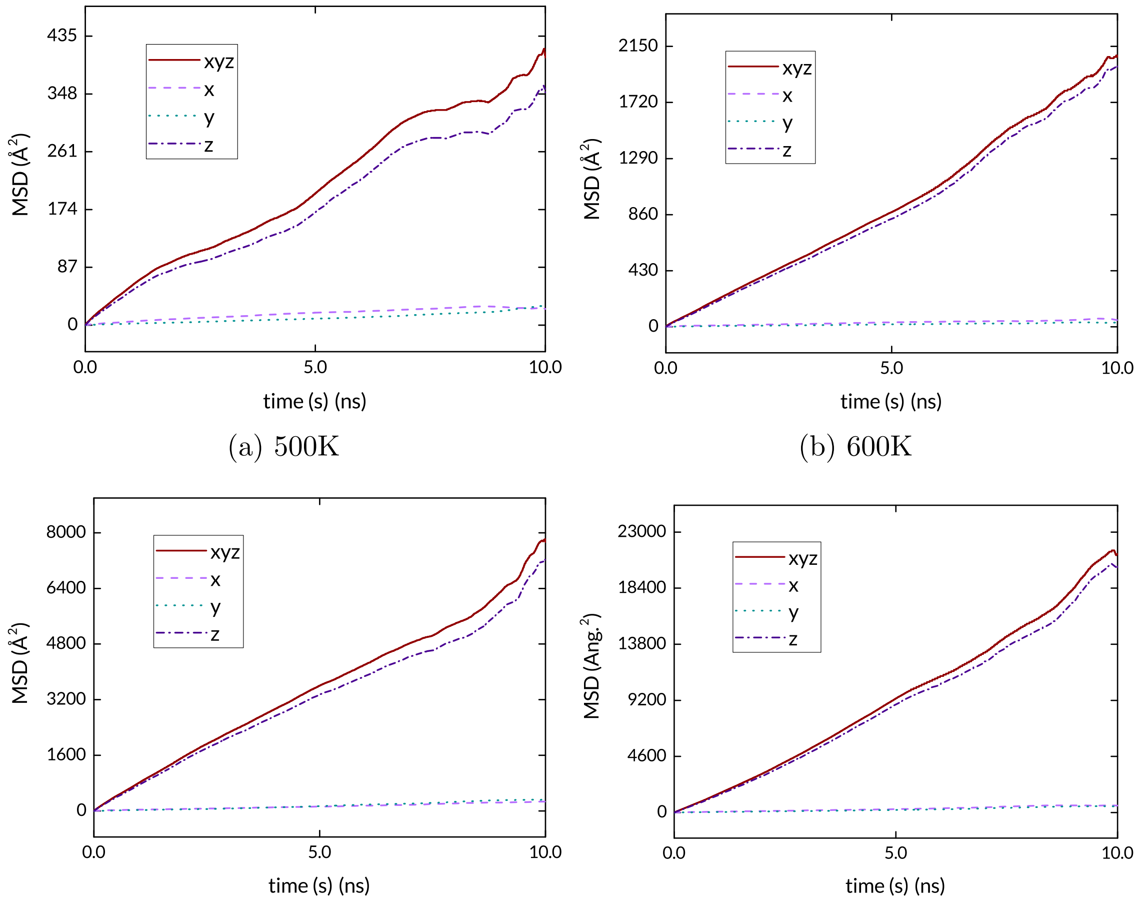

(a) 500K

(b) 600K

(c) 800K

(d) 900K

Figure S4: MSD plots for NVT simulations of H in Ru tilt GB.

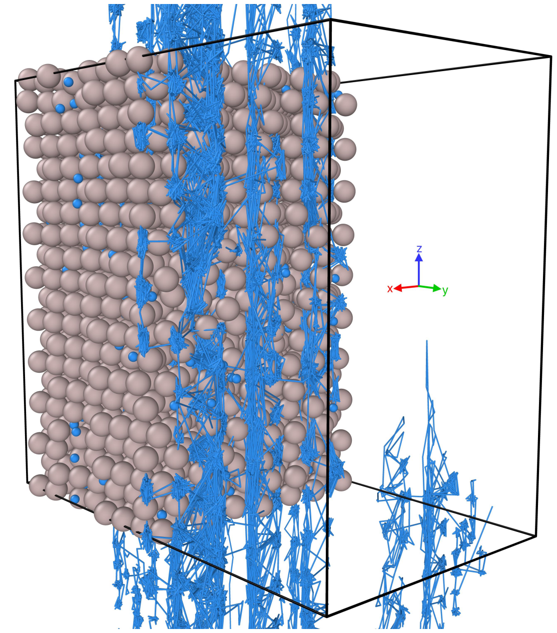

(a)

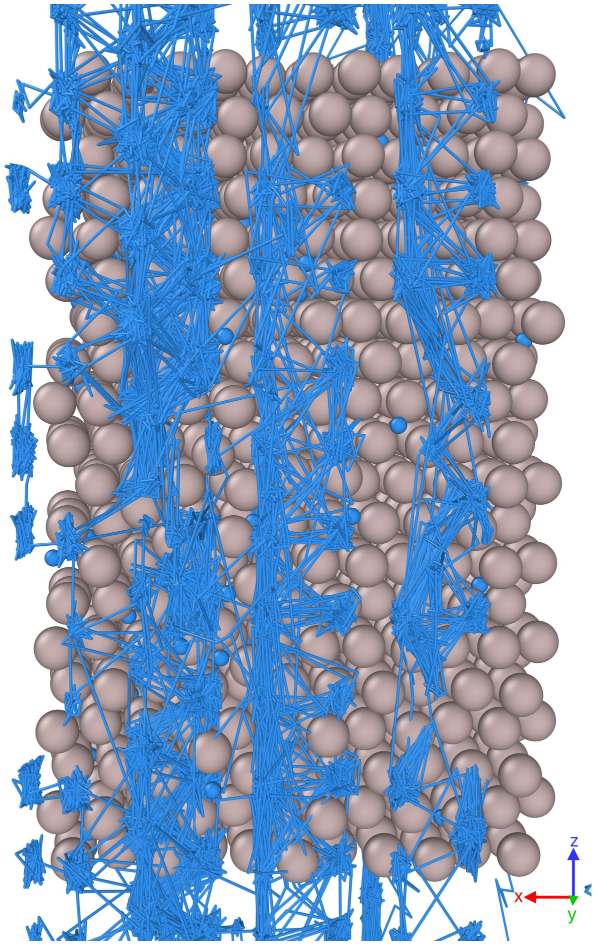

(b)

Figure S5: H diffusion channels in tilt GB structure.

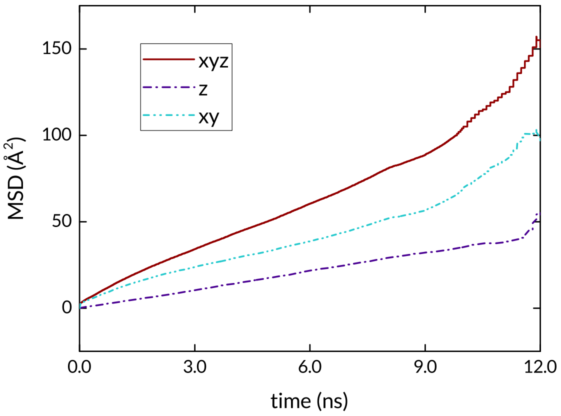

(a) 500K

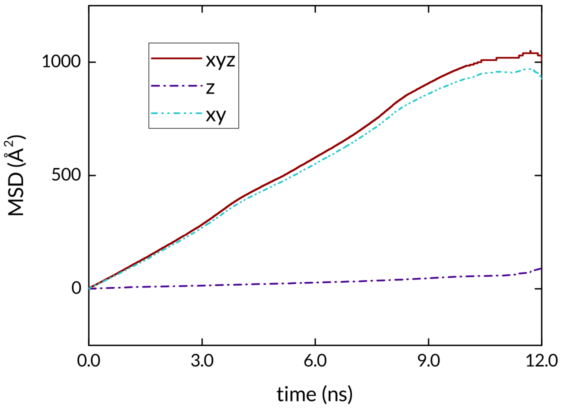

(b) 600K

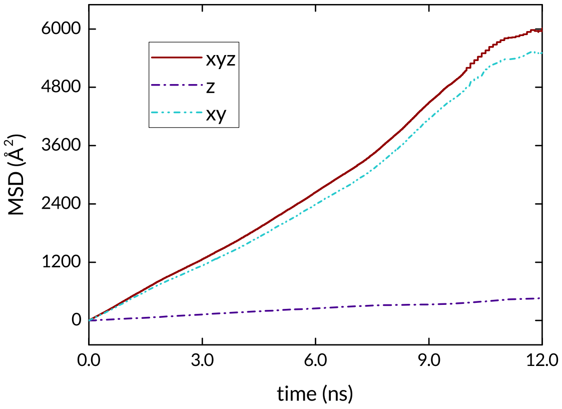

(c) 800K

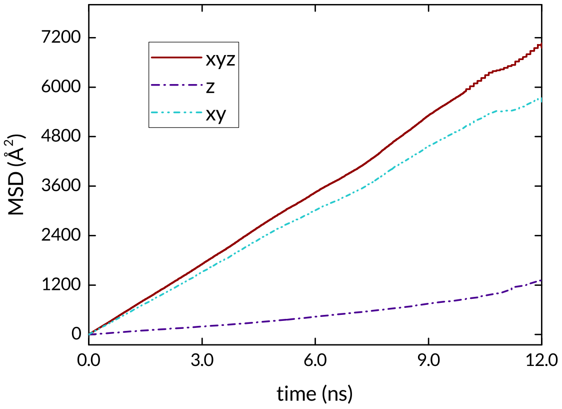

(d) 900K

Figure S6: MSD plots for NVT simulations of H in Ru twist GB. For the twist GB, at the lower temperature of 500K, the square root of the MSD is less than half the dimension of the simulation box. This heuristic implies that longer simulations are necessary to properly sample diffusion at this temperature.
